# Supplementary material for: Single cell functional genomics reveals the importance of mitochondria in cell-to-cell phenotypic variation
Source: eLife. 2019 Jan 14;8:e38904. doi: 10.7554/eLife.38904 (PMC6366901; doi:10.7554/eLife.38904)
Supplement: Supplementary file 4. — Multiple lines in each plot represent reproducible replicate measurements. x-axis represents microcolony growth rate (h−1) and y-axis represents density. [file elife-38904-supp4.pdf]

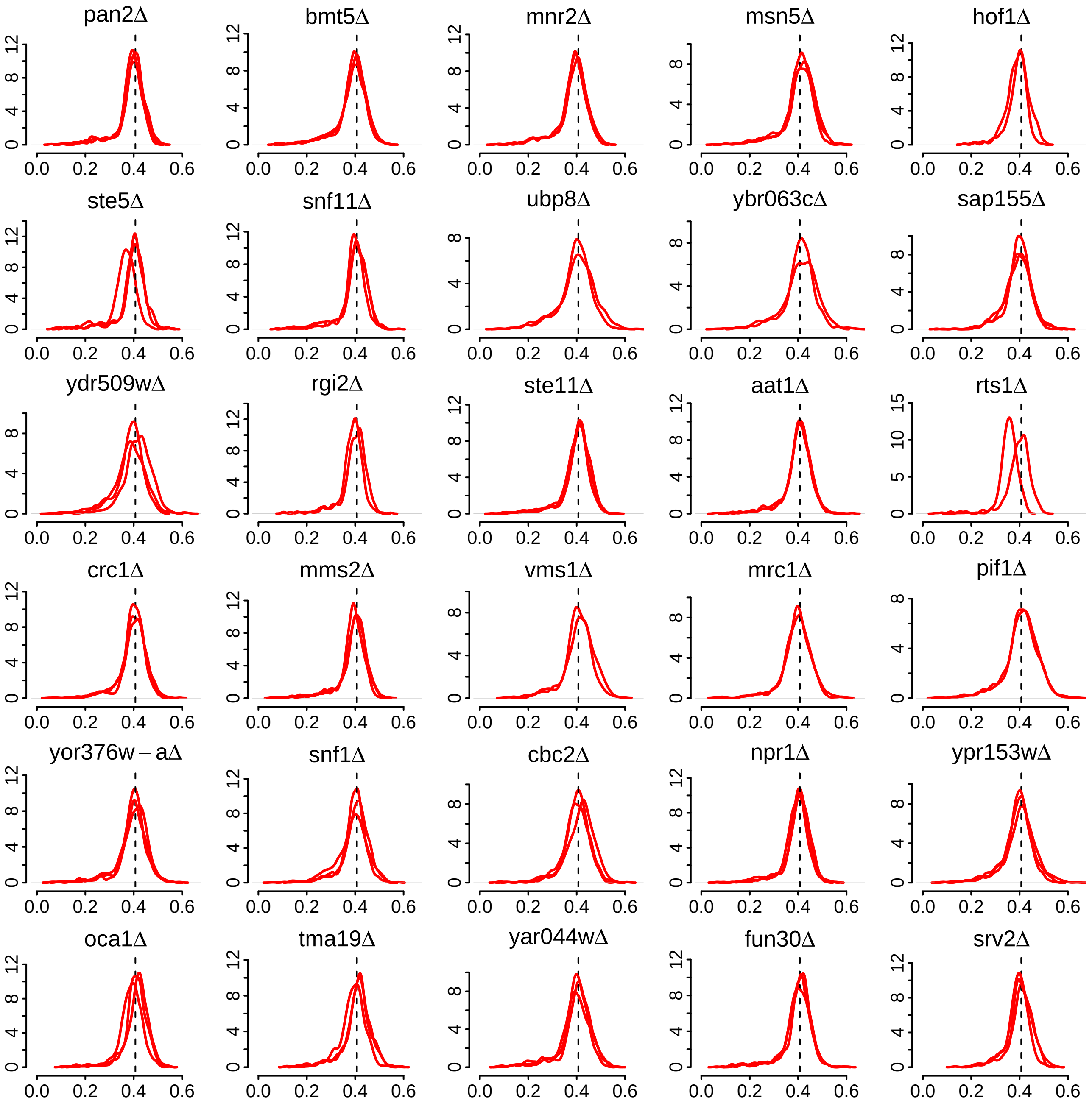
pan2Δ
bmt5Δ
mnr2Δ
msn5Δ
hof1Δ
ste5Δ
snf11Δ
ubp8Δ
ybr063cΔ
sap155Δ
ydr509wΔ
rgi2Δ
ste11Δ
aat1Δ
rts1Δ
crc1Δ
mms2Δ
vms1Δ
mrc1Δ
pif1Δ
yor376w – aΔ
snf1Δ
cbc2Δ
npr1Δ
ypr153wΔ
oca1Δ
tma19Δ
yar044wΔ
fun30Δ
srv2Δ

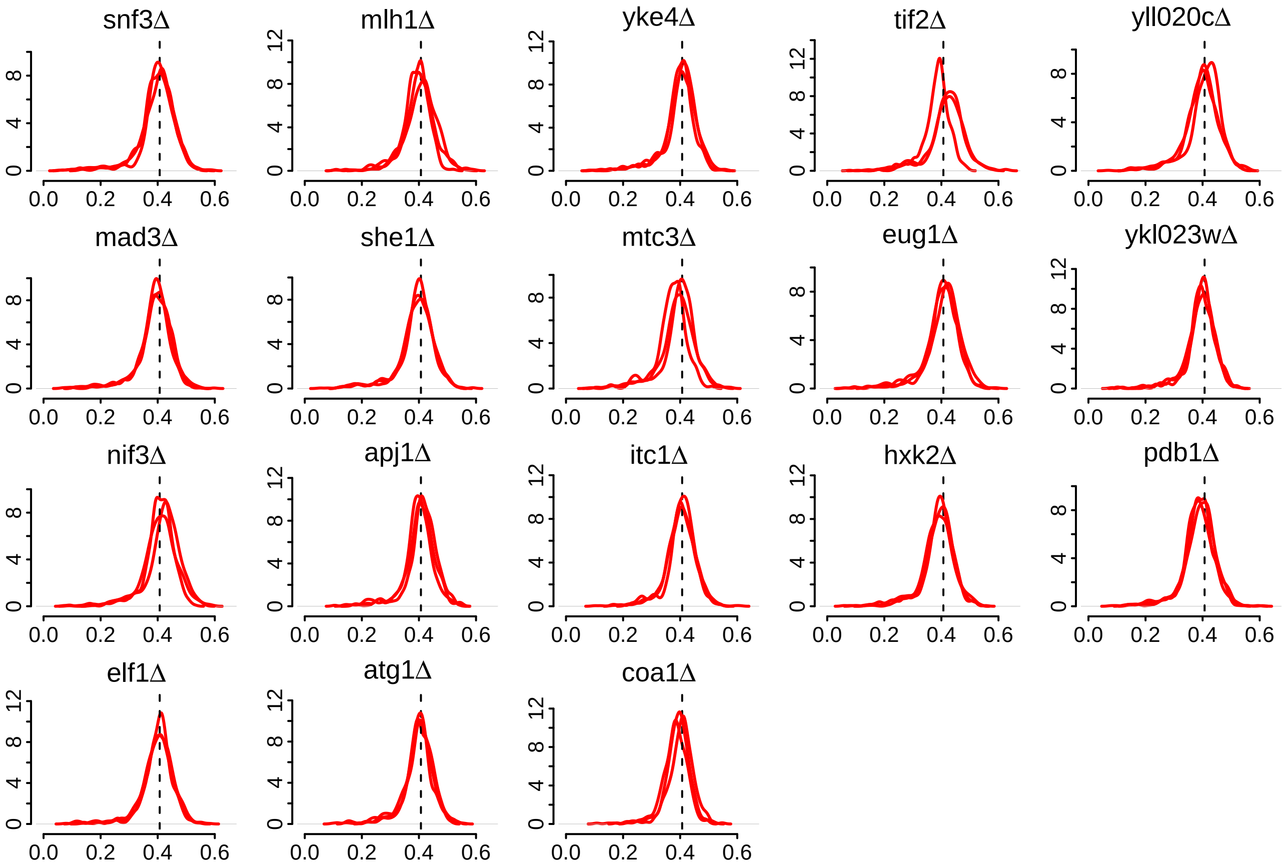

snf3Δ
mlh1Δ
yke4Δ
tif2Δ
yll020cΔ
mad3Δ
she1Δ
mtc3Δ
eug1Δ
ykl023wΔ
nif3Δ
apj1Δ
itc1Δ
hxk2Δ
pdb1Δ
elf1Δ
atg1Δ
coa1Δ
0.0 0.2 0.4 0.6
0 4 8 12

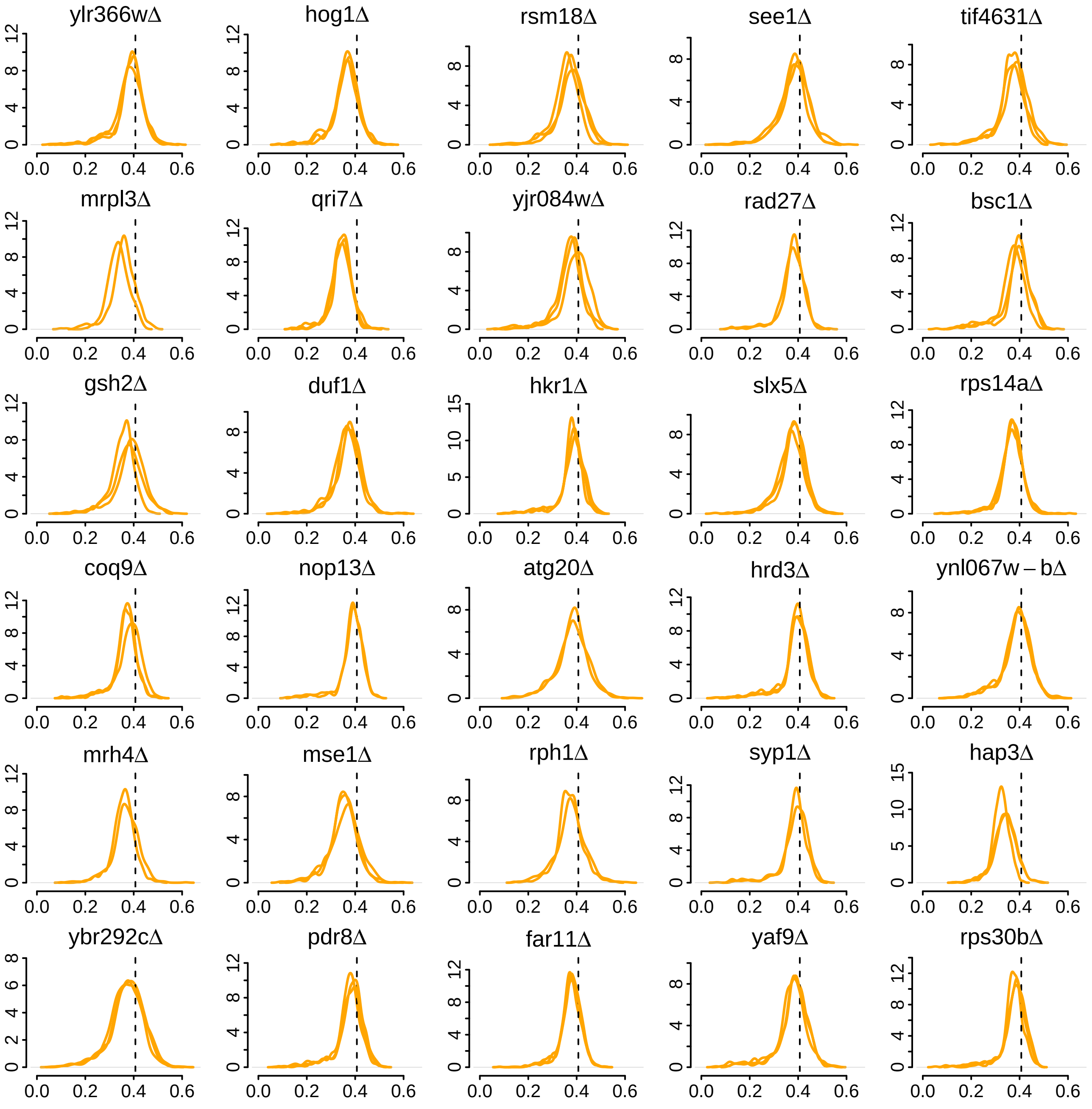
ylr366wΔ
hog1Δ
rsm18Δ
see1Δ
tif4631Δ
mrpl3Δ
qri7Δ
yjr084wΔ
rad27Δ
bsc1Δ
gsh2Δ
duf1Δ
hkr1Δ
slx5Δ
rps14aΔ
coq9Δ
nop13Δ
atg20Δ
hrd3Δ
ynl067w – bΔ
mrh4Δ
mse1Δ
rph1Δ
syp1Δ
hap3Δ
ybr292cΔ
pdr8Δ
far11Δ
yaf9Δ
rps30bΔ
0.0
0.2
0.4
0.6

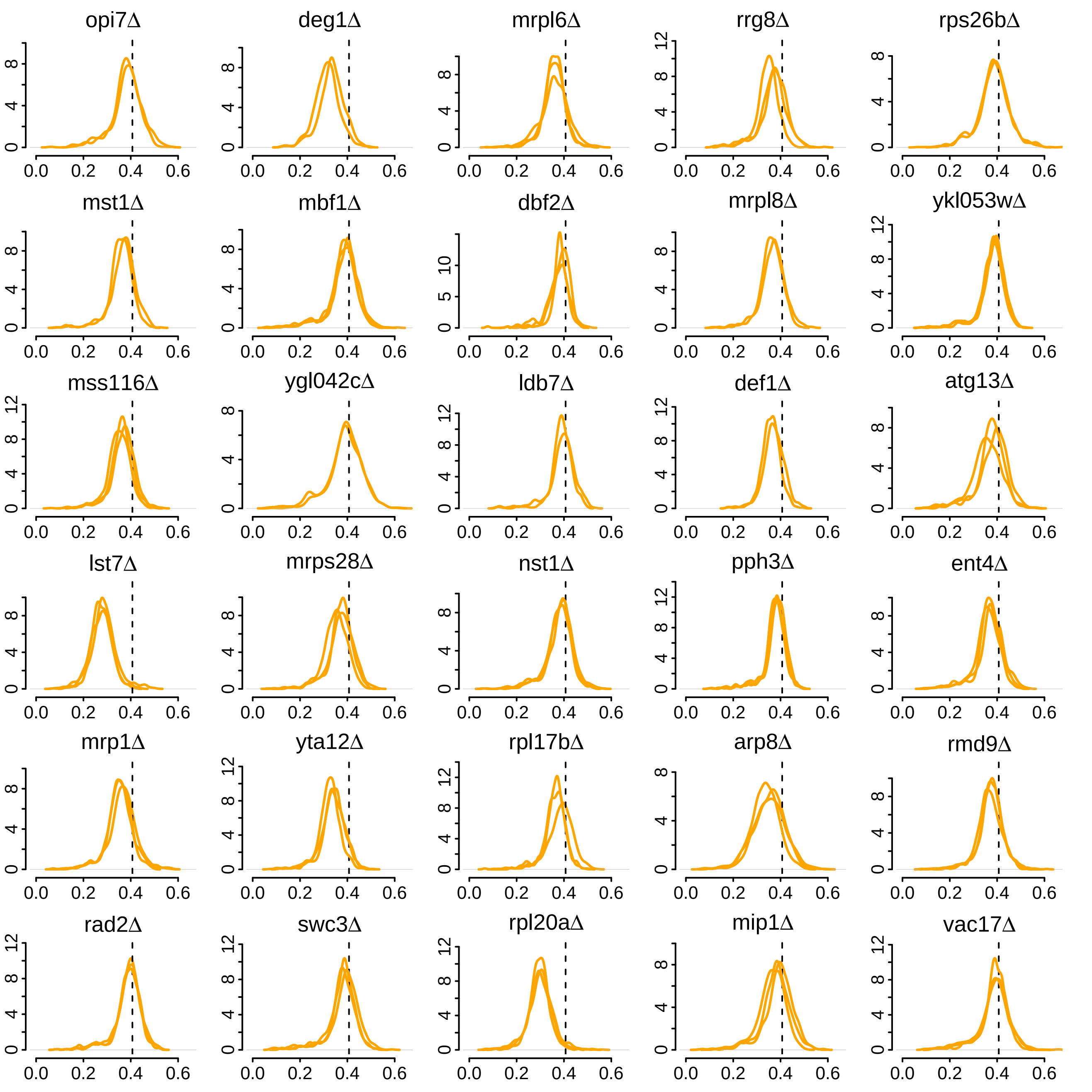
opi7Δ
deg1Δ
mrpl6Δ
rrg8Δ
rps26bΔ
mst1Δ
mbf1Δ
dbf2Δ
mrpl8Δ
ykl053wΔ
mss116Δ
ygl042cΔ
ldb7Δ
def1Δ
atg13Δ
lst7Δ
mrps28Δ
nst1Δ
pph3Δ
ent4Δ
mrp1Δ
yta12Δ
rpl17bΔ
arp8Δ
rmd9Δ
rad2Δ
swc3Δ
rpl20aΔ
mip1Δ
vac17Δ

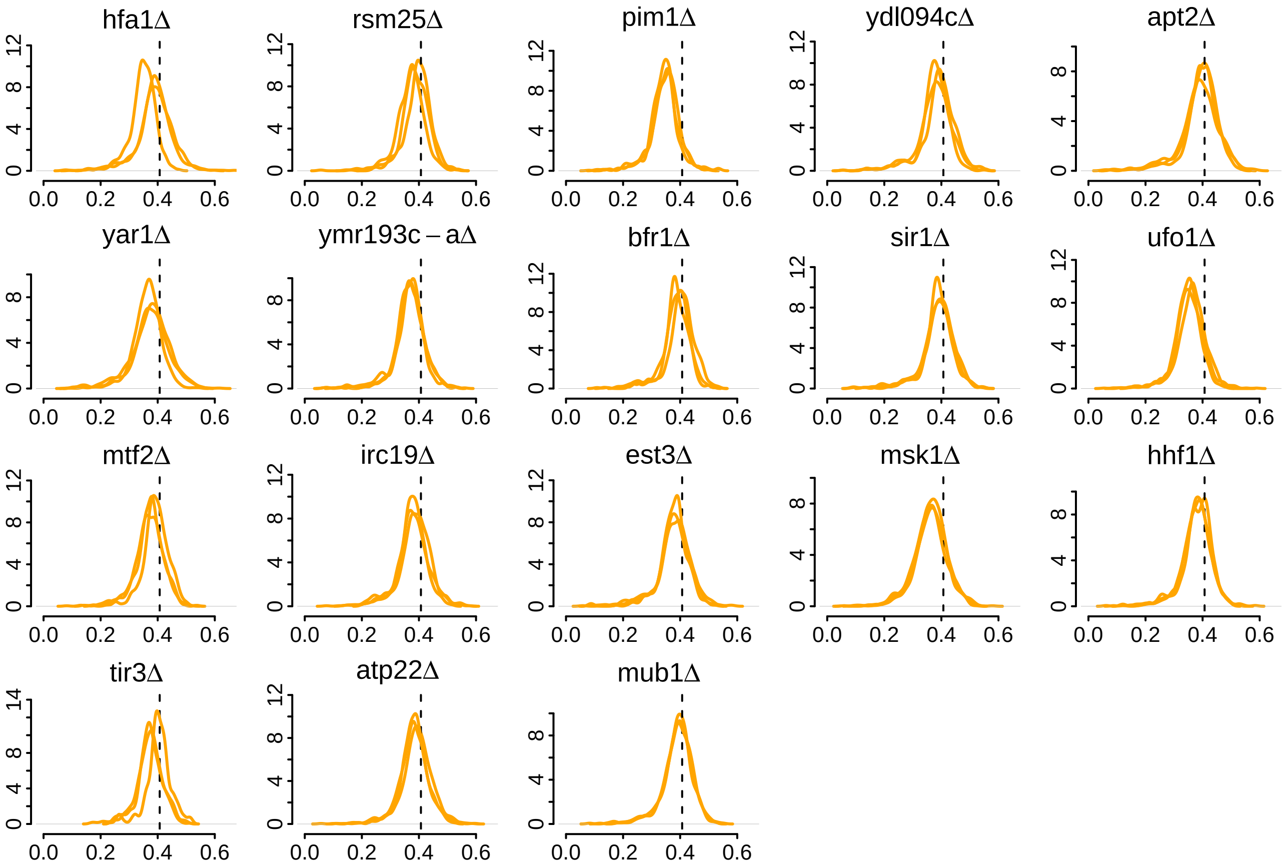

hfa1Δ
rsm25Δ
pim1Δ
ydl094cΔ
apt2Δ
yar1Δ
ymr193c – aΔ
bfr1Δ
sir1Δ
ufo1Δ
mtf2Δ
irc19Δ
est3Δ
msk1Δ
hhf1Δ
tir3Δ
atp22Δ
mub1Δ
0.0 0.2 0.4 0.6
0 4 8 12
0 4 8 12 14

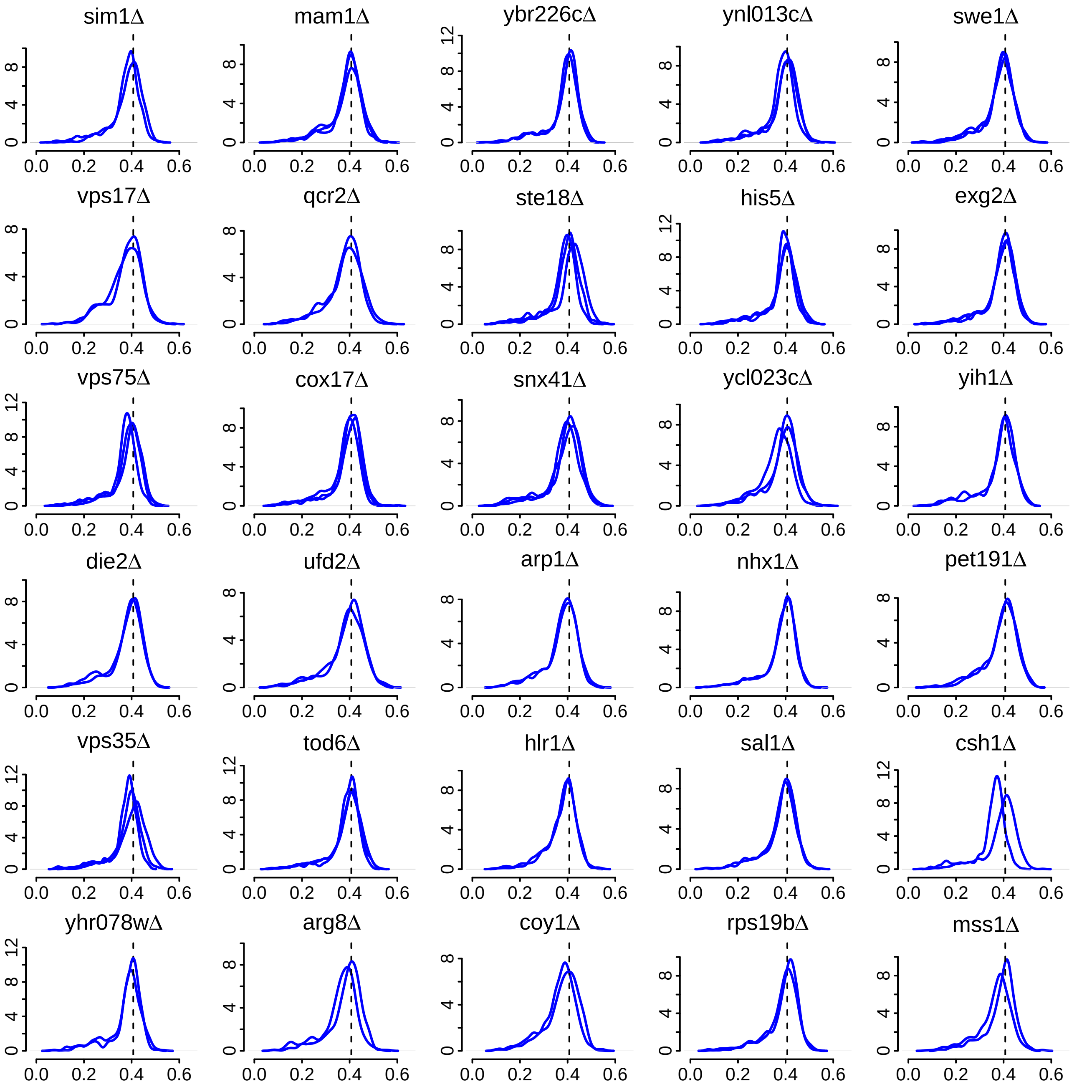
sim1Δ
mam1Δ
ybr226cΔ
ynl013cΔ
swe1Δ
vps17Δ
qcr2Δ
ste18Δ
his5Δ
exg2Δ
vps75Δ
cox17Δ
snx41Δ
ycl023cΔ
yih1Δ
die2Δ
ufd2Δ
arp1Δ
nhx1Δ
pet191Δ
vps35Δ
tod6Δ
hlr1Δ
sal1Δ
csh1Δ
yhr078wΔ
arg8Δ
coy1Δ
rps19bΔ
mss1Δ
0.0 0.2 0.4 0.6
0 4 8 12

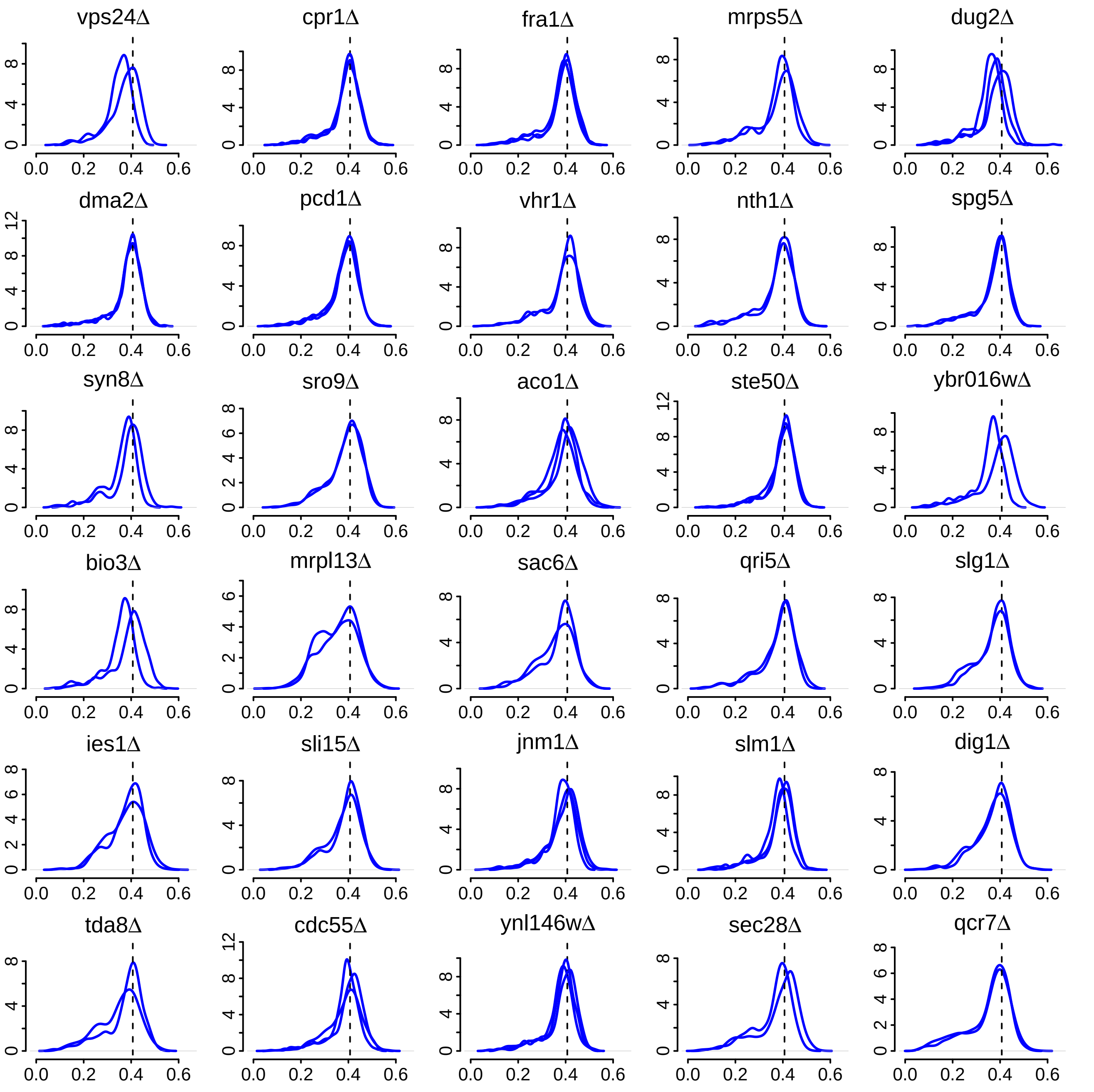

vps24Δ
0 4 8
0.0 0.2 0.4 0.6
cpr1Δ
0 4 8
0.0 0.2 0.4 0.6
fra1Δ
0 4 8
0.0 0.2 0.4 0.6
mrps5Δ
0 4 8
0.0 0.2 0.4 0.6
dug2Δ
0 4 8
0.0 0.2 0.4 0.6
dma2Δ
0 4 8 12
0.0 0.2 0.4 0.6
pcd1Δ
0 4 8
0.0 0.2 0.4 0.6
vhr1Δ
0 4 8
0.0 0.2 0.4 0.6
nth1Δ
0 4 8
0.0 0.2 0.4 0.6
spg5Δ
0 4 8
0.0 0.2 0.4 0.6
syn8Δ
0 4 8
0.0 0.2 0.4 0.6
sro9Δ
0 2 4 6 8
0.0 0.2 0.4 0.6
aco1Δ
0 4 8
0.0 0.2 0.4 0.6
ste50Δ
0 4 8 12
0.0 0.2 0.4 0.6
ybr016wΔ
0 4 8
0.0 0.2 0.4 0.6
bio3Δ
0 4 8
0.0 0.2 0.4 0.6
mrpl13Δ
0 2 4 6
0.0 0.2 0.4 0.6
sac6Δ
0 4 8
0.0 0.2 0.4 0.6
qri5Δ
0 4 8
0.0 0.2 0.4 0.6
slg1Δ
0 4 8
0.0 0.2 0.4 0.6
ies1Δ
0 2 4 6 8
0.0 0.2 0.4 0.6
sli15Δ
0 4 8
0.0 0.2 0.4 0.6
jnm1Δ
0 4 8
0.0 0.2 0.4 0.6
slm1Δ
0 4 8
0.0 0.2 0.4 0.6
dig1Δ
0 4 8
0.0 0.2 0.4 0.6
tda8Δ
0 4 8
0.0 0.2 0.4 0.6
cdc55Δ
0 4 8 12
0.0 0.2 0.4 0.6
ynl146wΔ
0 4 8
0.0 0.2 0.4 0.6
sec28Δ
0 4 8
0.0 0.2 0.4 0.6
qcr7Δ
0 2 4 6 8
0.0 0.2 0.4 0.6

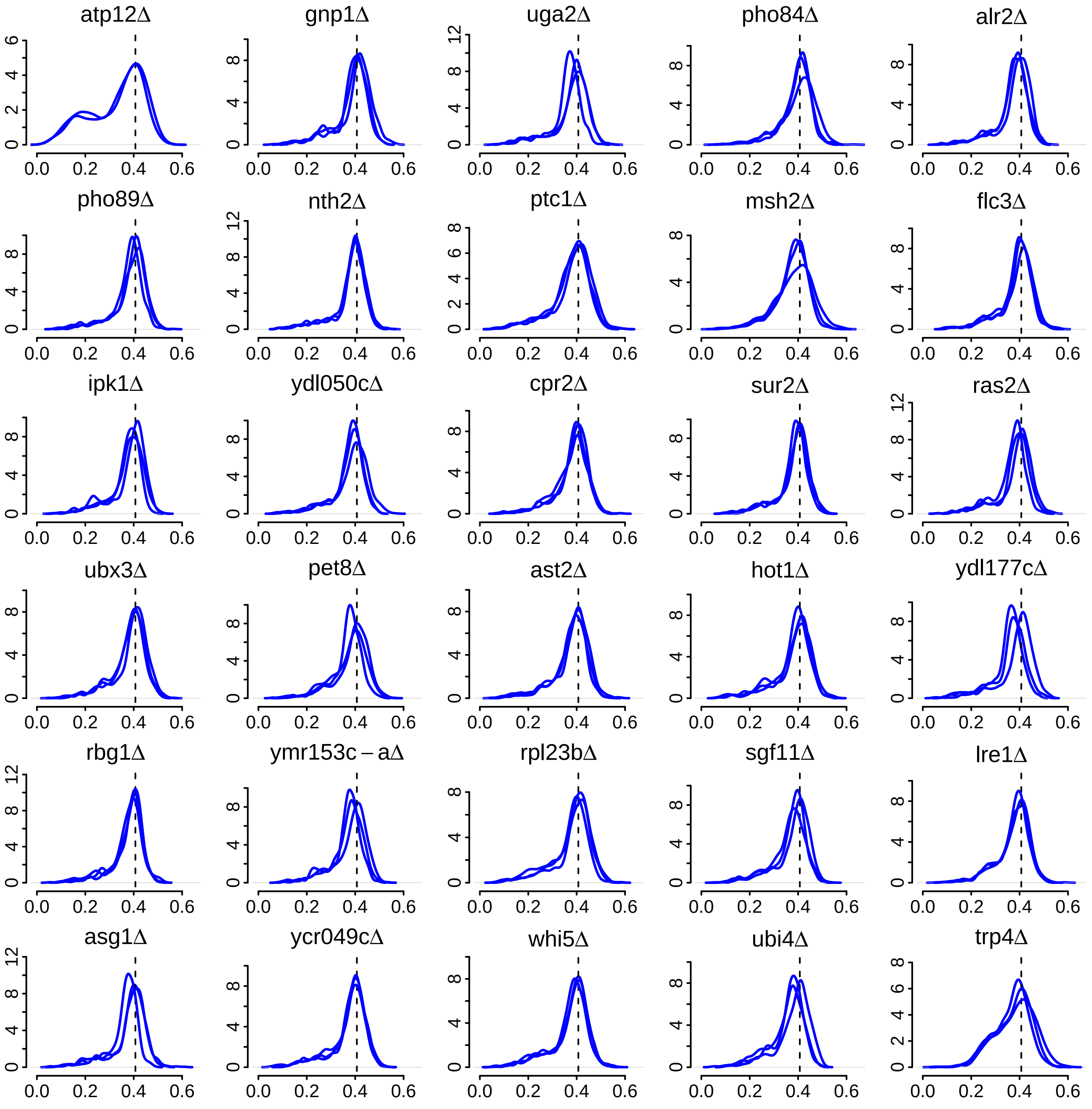
atp12Δ
gnp1Δ
uga2Δ
pho84Δ
alr2Δ
pho89Δ
nth2Δ
ptc1Δ
msh2Δ
flc3Δ
ipk1Δ
ydl050cΔ
cpr2Δ
sur2Δ
ras2Δ
ubx3Δ
pet8Δ
ast2Δ
hot1Δ
ydl177cΔ
rbg1Δ
ymr153c – aΔ
rpl23bΔ
sgf11Δ
lre1Δ
asg1Δ
ycr049cΔ
whi5Δ
ubi4Δ
trp4Δ

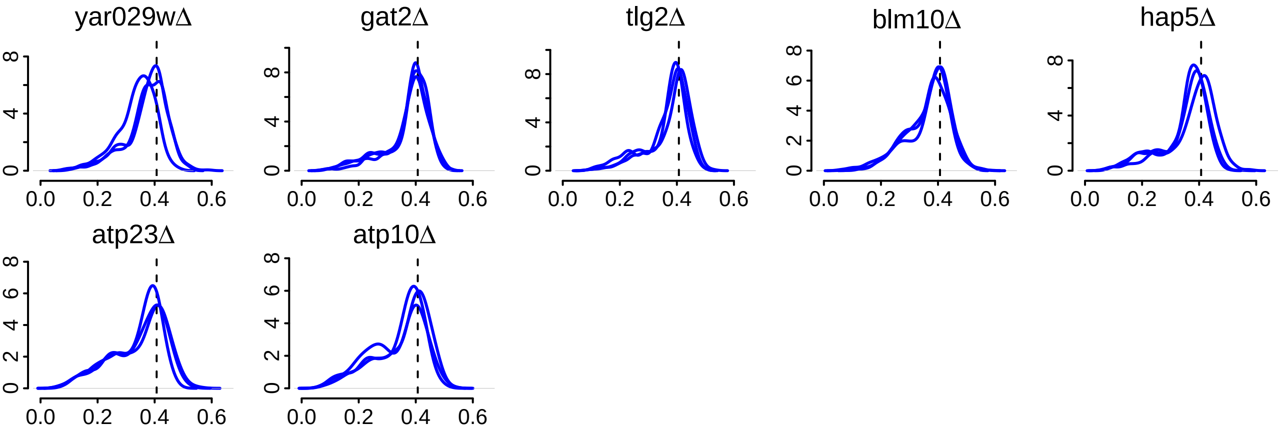

yar029wΔ
gat2Δ
tlg2Δ
blm10Δ
hap5Δ
atp23Δ
atp10Δ
0 2 4 6 8
0.0 0.2 0.4 0.6

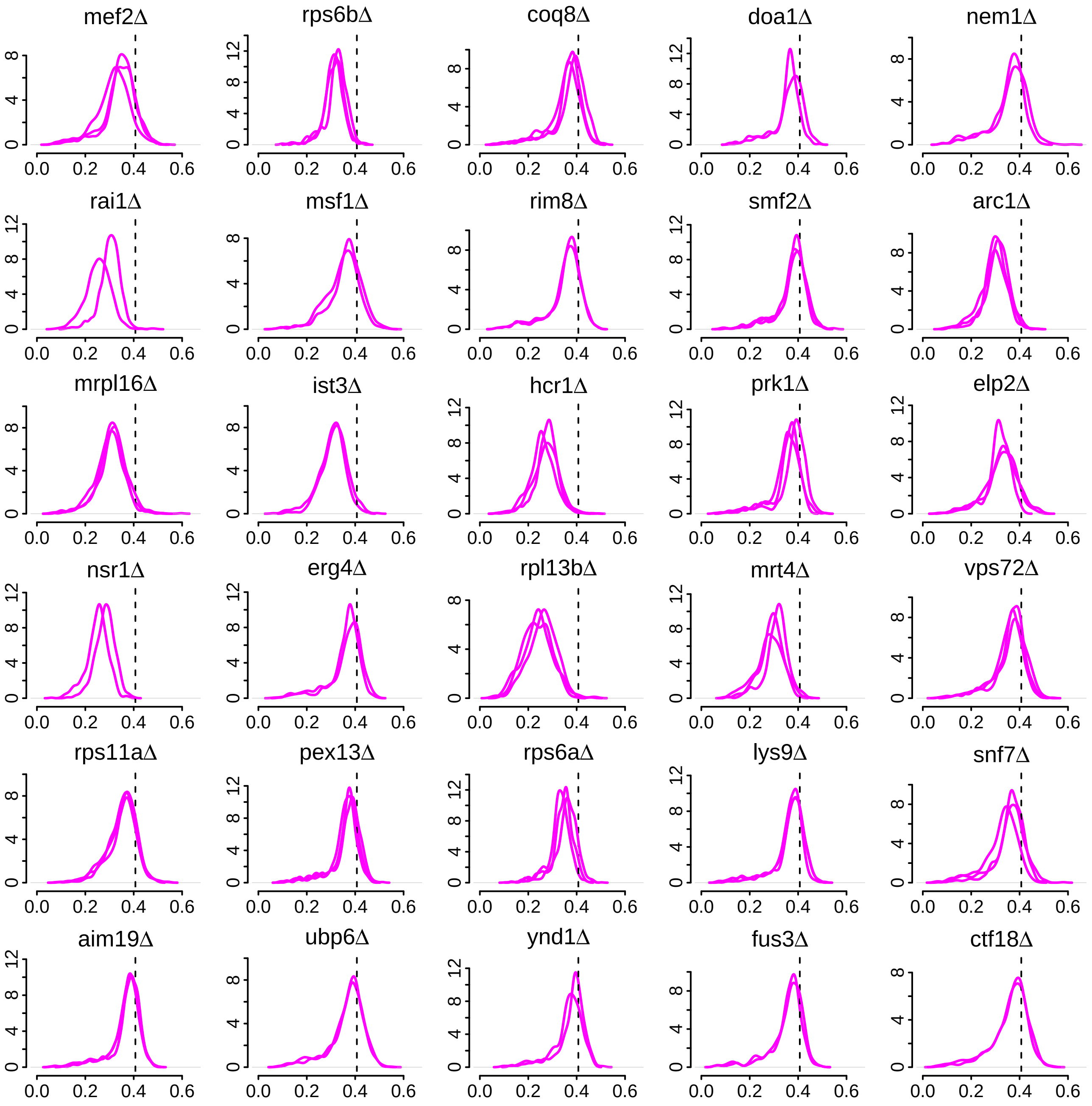
mef2Δ
rps6bΔ
coq8Δ
doa1Δ
nem1Δ
rai1Δ
msf1Δ
rim8Δ
smf2Δ
arc1Δ
mrpl16Δ
ist3Δ
hcr1Δ
prk1Δ
elp2Δ
nsr1Δ
erg4Δ
rpl13bΔ
mrt4Δ
vps72Δ
rps11aΔ
pex13Δ
rps6aΔ
lys9Δ
snf7Δ
aim19Δ
ubp6Δ
ynd1Δ
fus3Δ
ctf18Δ
0.0
0.2
0.4
0.6

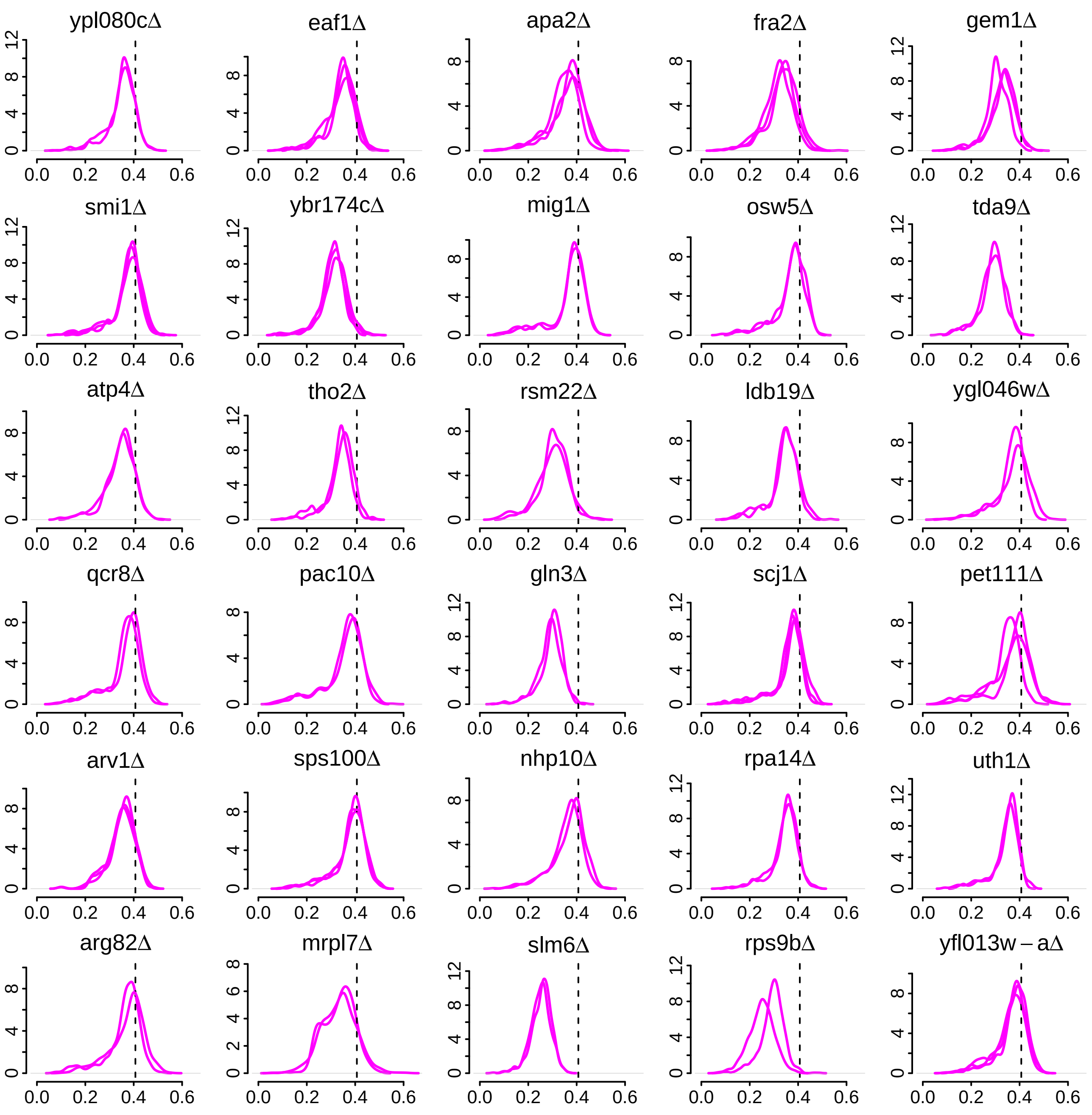
ypl080cΔ
eaf1Δ
apa2Δ
fra2Δ
gem1Δ
smi1Δ
ybr174cΔ
mig1Δ
osw5Δ
tda9Δ
atp4Δ
tho2Δ
rsm22Δ
ldb19Δ
ygl046wΔ
qcr8Δ
pac10Δ
gln3Δ
scj1Δ
pet111Δ
arv1Δ
sps100Δ
nhp10Δ
rpa14Δ
uth1Δ
arg82Δ
mrpl7Δ
slm6Δ
rps9bΔ
yfl013w – aΔ
0.0
0.2
0.4
0.6

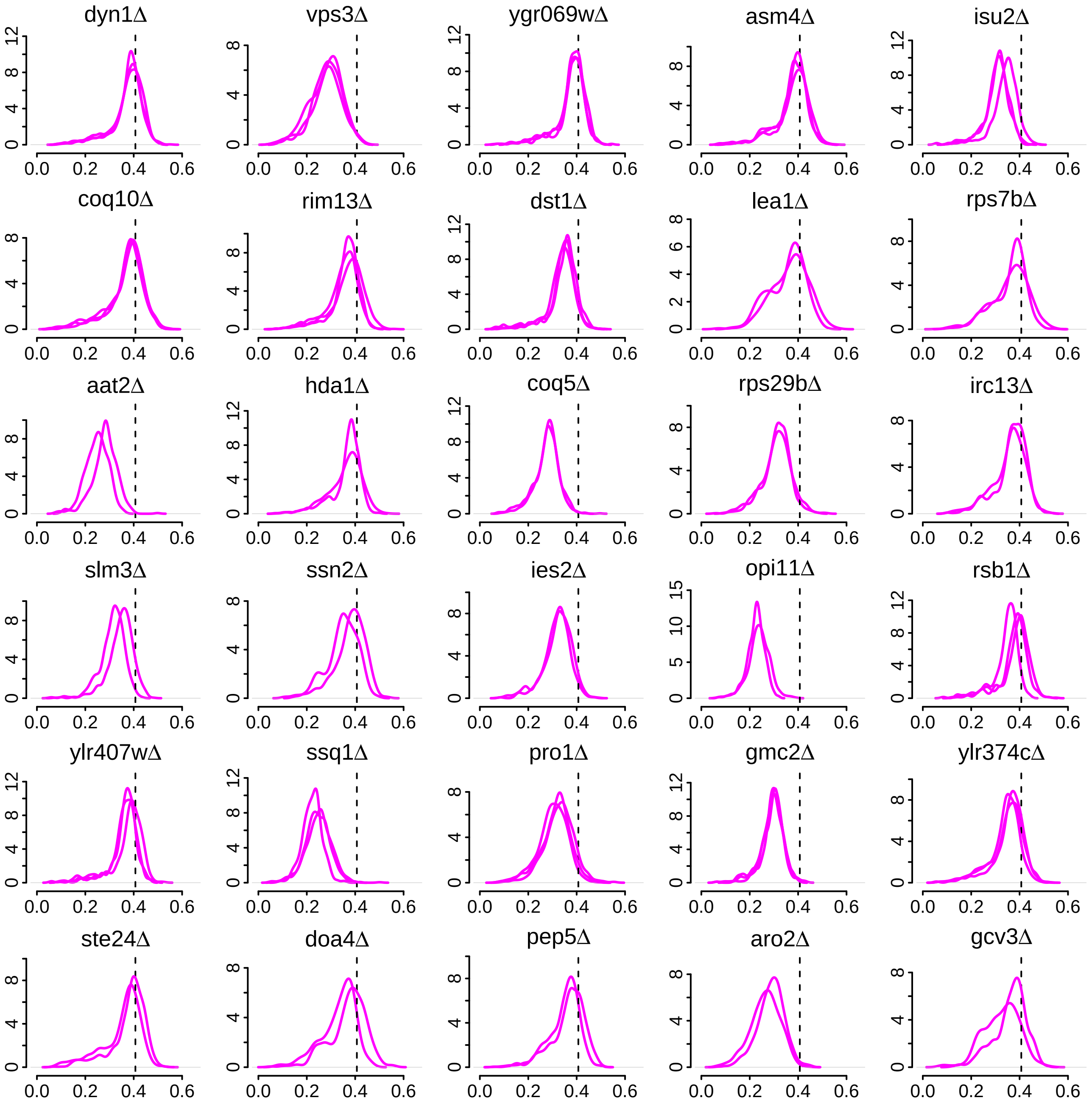

dyn1Δ
vps3Δ
ygr069wΔ
asm4Δ
isu2Δ
coq10Δ
rim13Δ
dst1Δ
lea1Δ
rps7bΔ
aat2Δ
hda1Δ
coq5Δ
rps29bΔ
irc13Δ
slm3Δ
ssn2Δ
ies2Δ
opi11Δ
rsb1Δ
ylr407wΔ
ssq1Δ
pro1Δ
gmc2Δ
ylr374cΔ
ste24Δ
doa4Δ
pep5Δ
aro2Δ
gcv3Δ
0.0 0.2 0.4 0.6

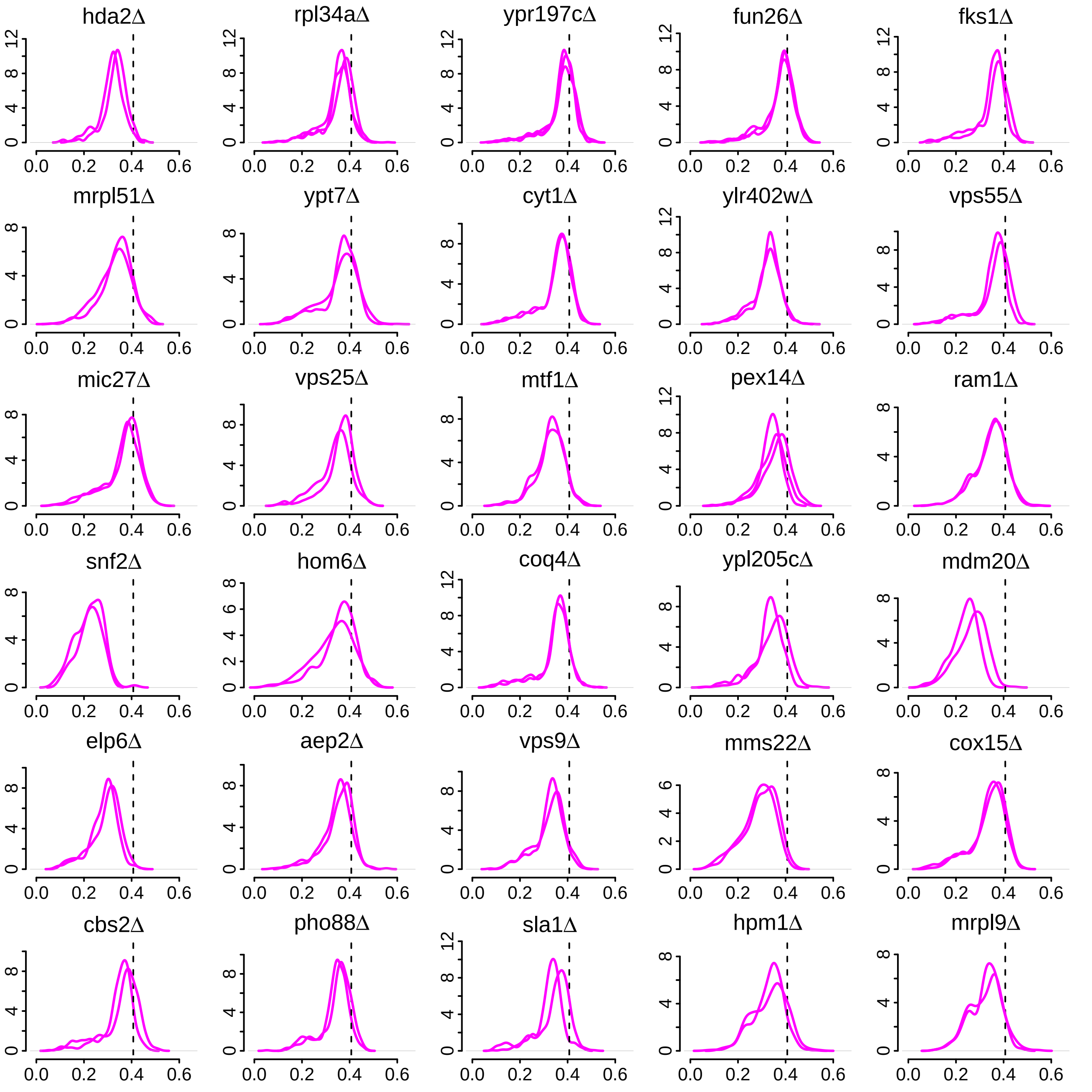

hda2Δ
rpl34aΔ
ypr197cΔ
fun26Δ
fks1Δ
mrpl51Δ
ypt7Δ
cyt1Δ
ylr402wΔ
vps55Δ
mic27Δ
vps25Δ
mtf1Δ
pex14Δ
ram1Δ
snf2Δ
hom6Δ
coq4Δ
ypl205cΔ
mdm20Δ
elp6Δ
aep2Δ
vps9Δ
mms22Δ
cox15Δ
cbs2Δ
pho88Δ
sla1Δ
hpm1Δ
mrpl9Δ
0.0
0.2
0.4
0.6

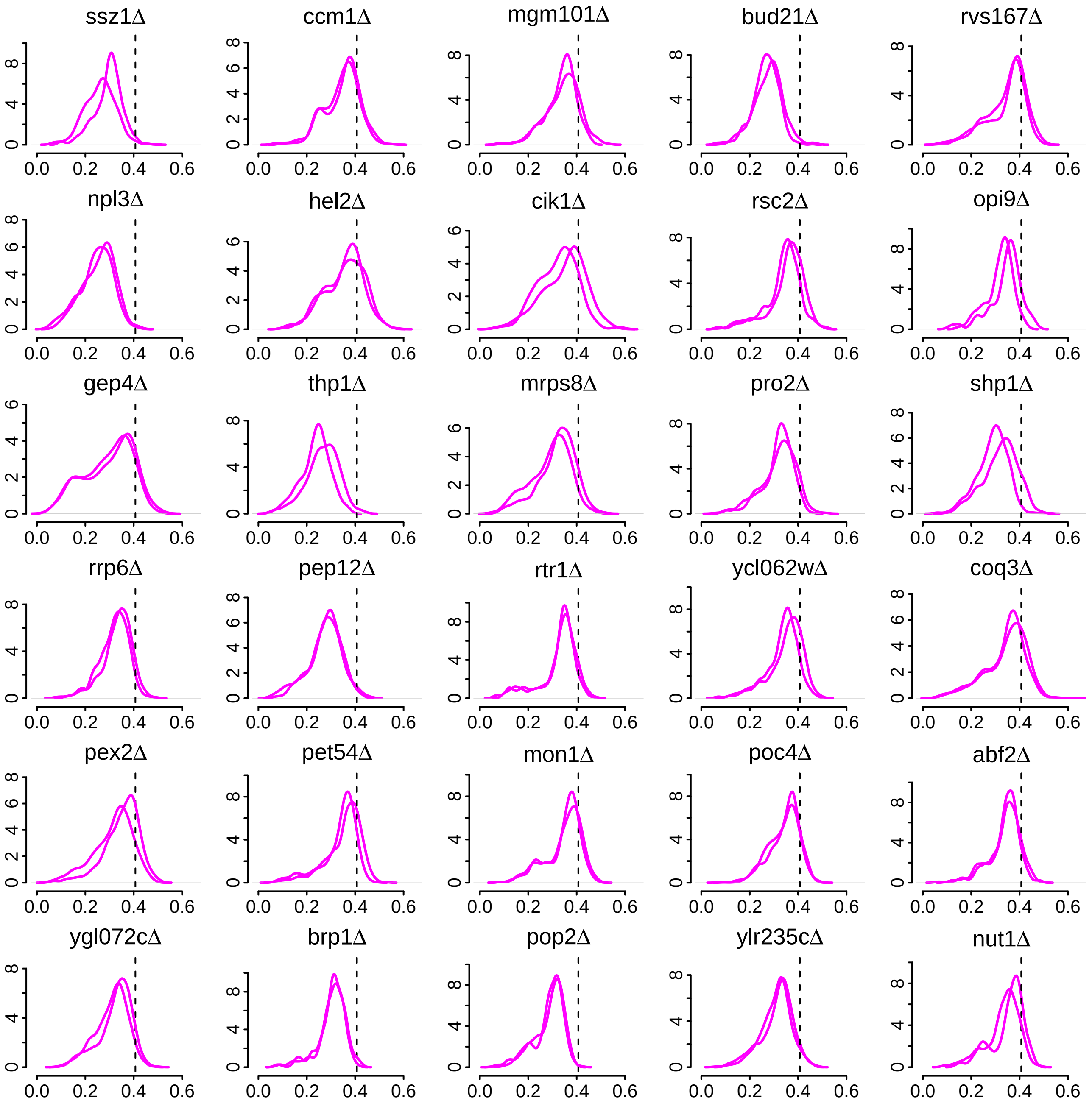

ssz1Δ
ccm1Δ
mgm101Δ
bud21Δ
rvs167Δ
npl3Δ
hel2Δ
cik1Δ
rsc2Δ
opi9Δ
gep4Δ
thp1Δ
mrps8Δ
pro2Δ
shp1Δ
rrp6Δ
pep12Δ
rtr1Δ
ycl062wΔ
coq3Δ
pex2Δ
pet54Δ
mon1Δ
poc4Δ
abf2Δ
ygl072cΔ
brp1Δ
pop2Δ
ylr235cΔ
nut1Δ
0.0
0.2
0.4
0.6

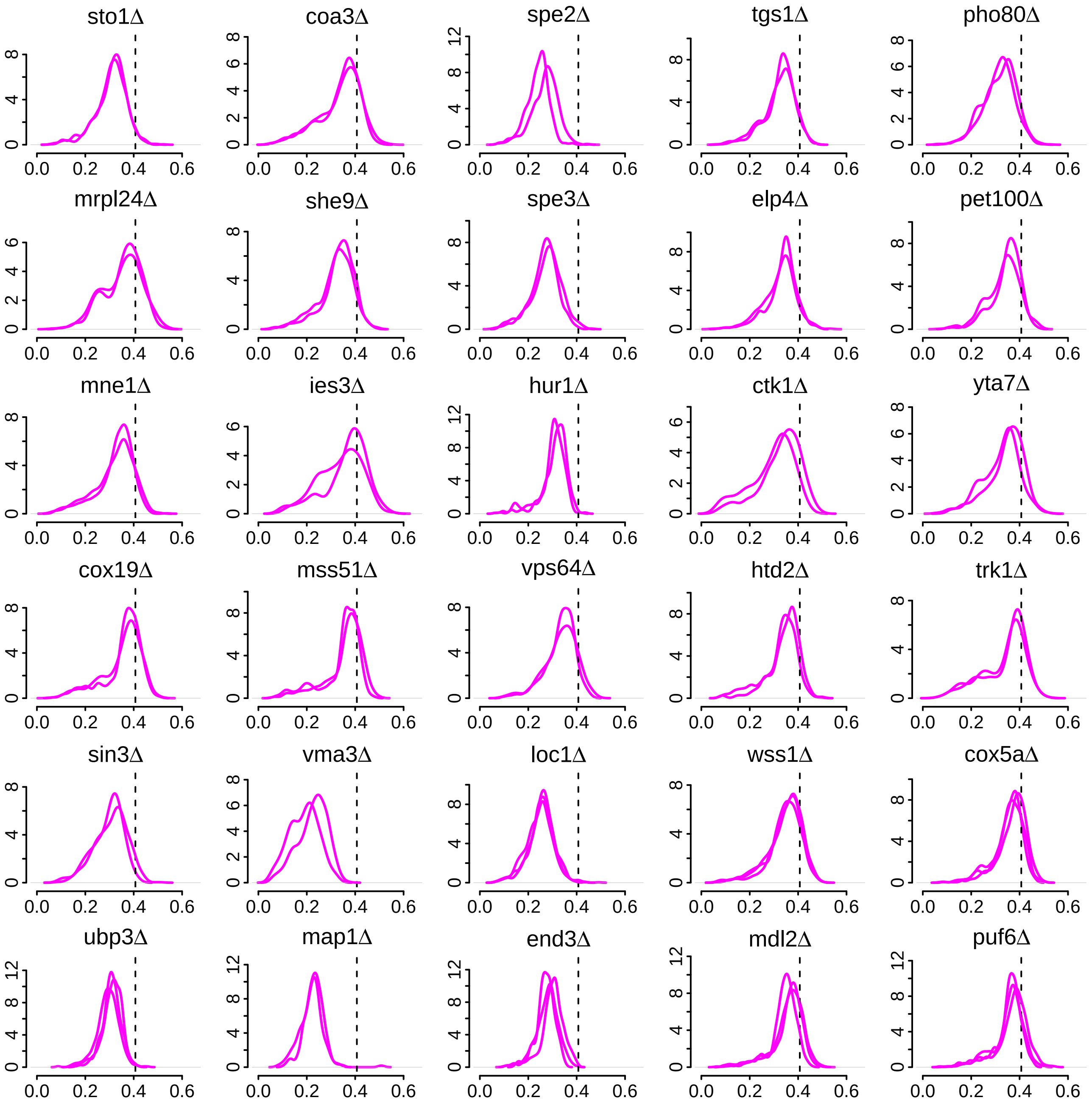
sto1Δ
coa3Δ
spe2Δ
tgs1Δ
pho80Δ
mrpl24Δ
she9Δ
spe3Δ
elp4Δ
pet100Δ
mne1Δ
ies3Δ
hur1Δ
ctk1Δ
yta7Δ
cox19Δ
mss51Δ
vps64Δ
htd2Δ
trk1Δ
sin3Δ
vma3Δ
loc1Δ
wss1Δ
cox5aΔ
ubp3Δ
map1Δ
end3Δ
mdl2Δ
puf6Δ
0.0 0.2 0.4 0.6

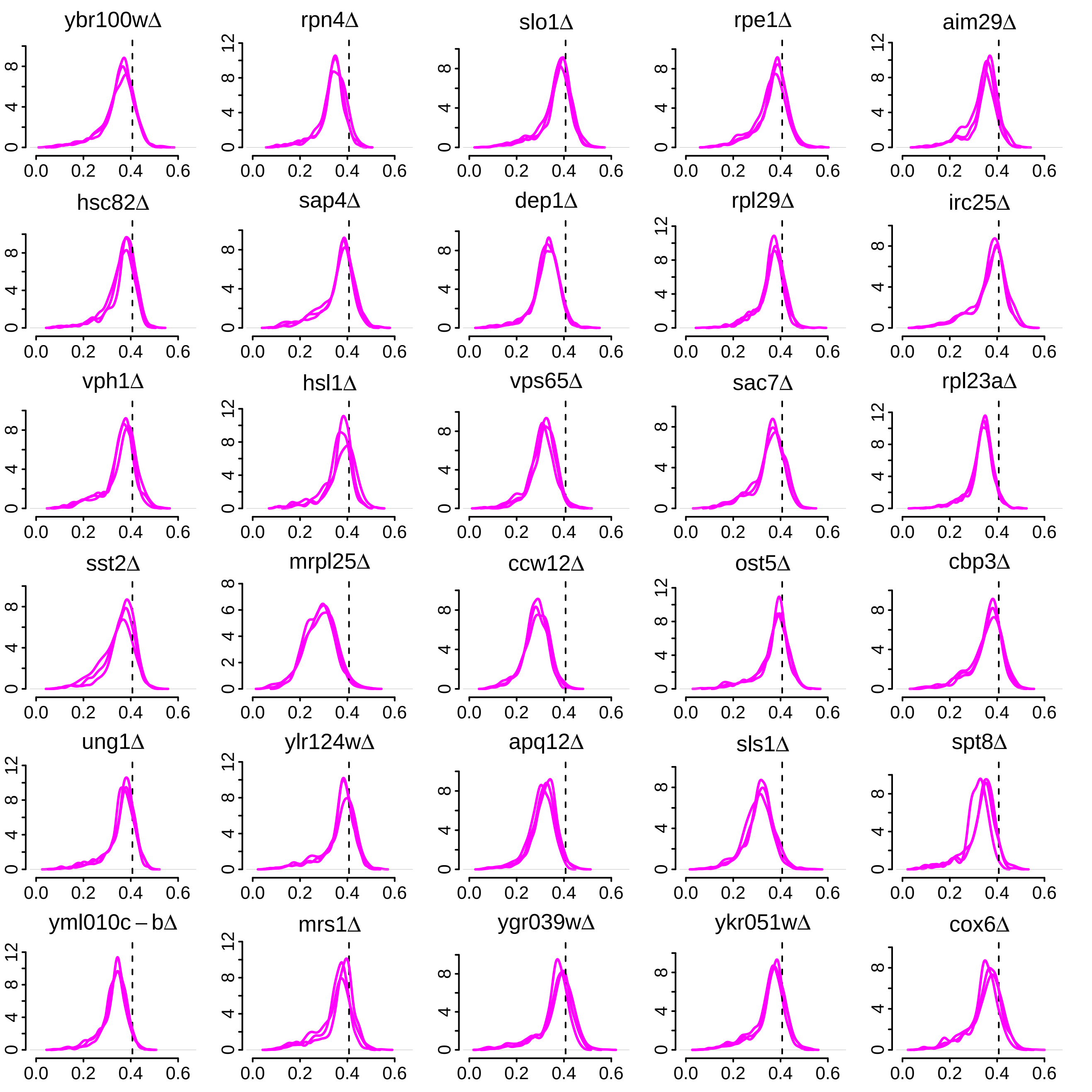
ybr100wΔ
rpn4Δ
slo1Δ
rpe1Δ
aim29Δ
hsc82Δ
sap4Δ
dep1Δ
rpl29Δ
irc25Δ
vph1Δ
hsl1Δ
vps65Δ
sac7Δ
rpl23aΔ
sst2Δ
mrpl25Δ
ccw12Δ
ost5Δ
cbp3Δ
ung1Δ
ylr124wΔ
apq12Δ
sls1Δ
spt8Δ
yml010c – bΔ
mrs1Δ
ygr039wΔ
ykr051wΔ
cox6Δ

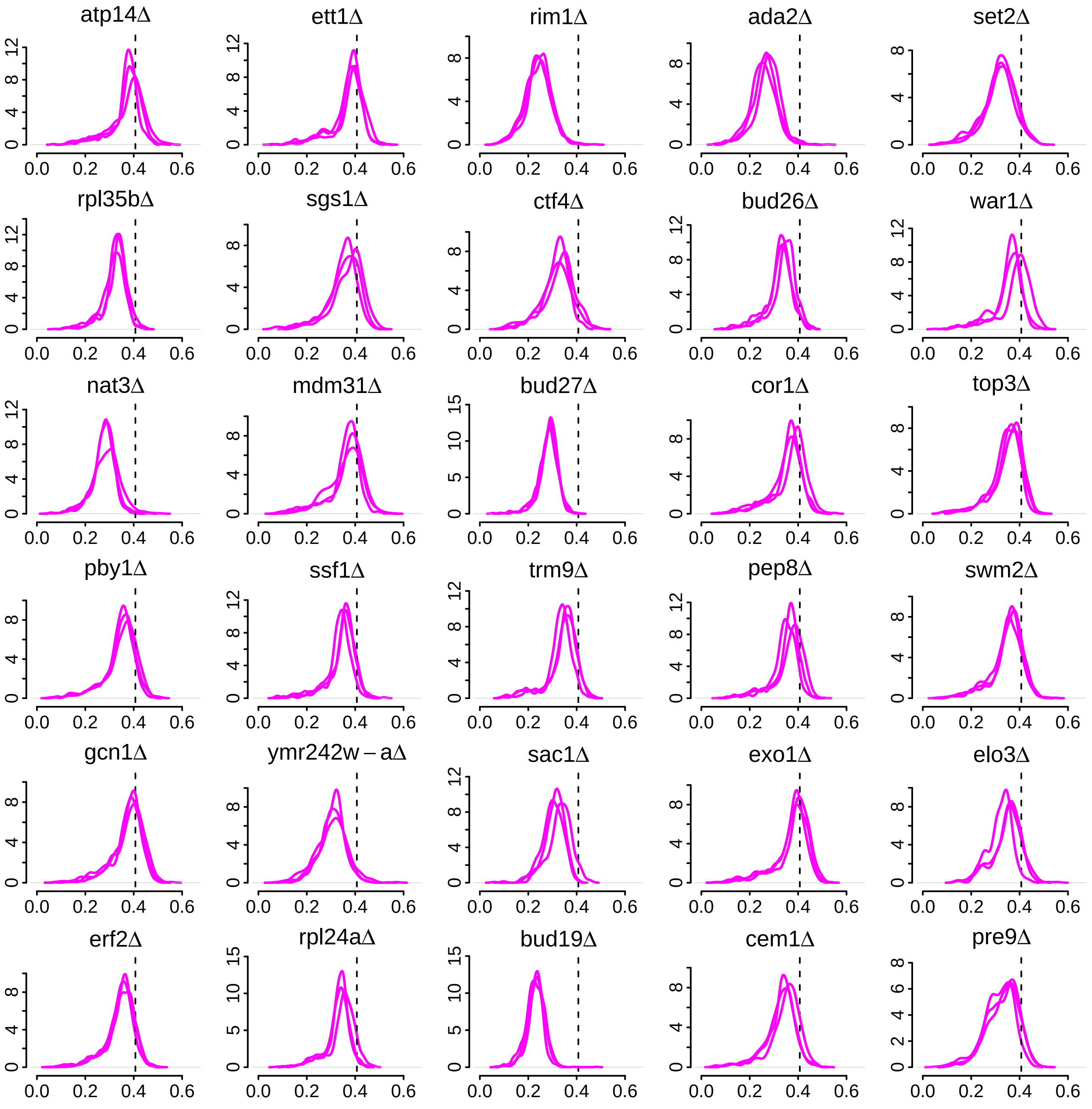
atp14Δ
ett1Δ
rim1Δ
ada2Δ
set2Δ
rpl35bΔ
sgs1Δ
ctf4Δ
bud26Δ
war1Δ
nat3Δ
mdm31Δ
bud27Δ
cor1Δ
top3Δ
pby1Δ
ssf1Δ
trm9Δ
pep8Δ
swm2Δ
gcn1Δ
ymr242w – aΔ
sac1Δ
exo1Δ
elo3Δ
erf2Δ
rpl24aΔ
bud19Δ
cem1Δ
pre9Δ

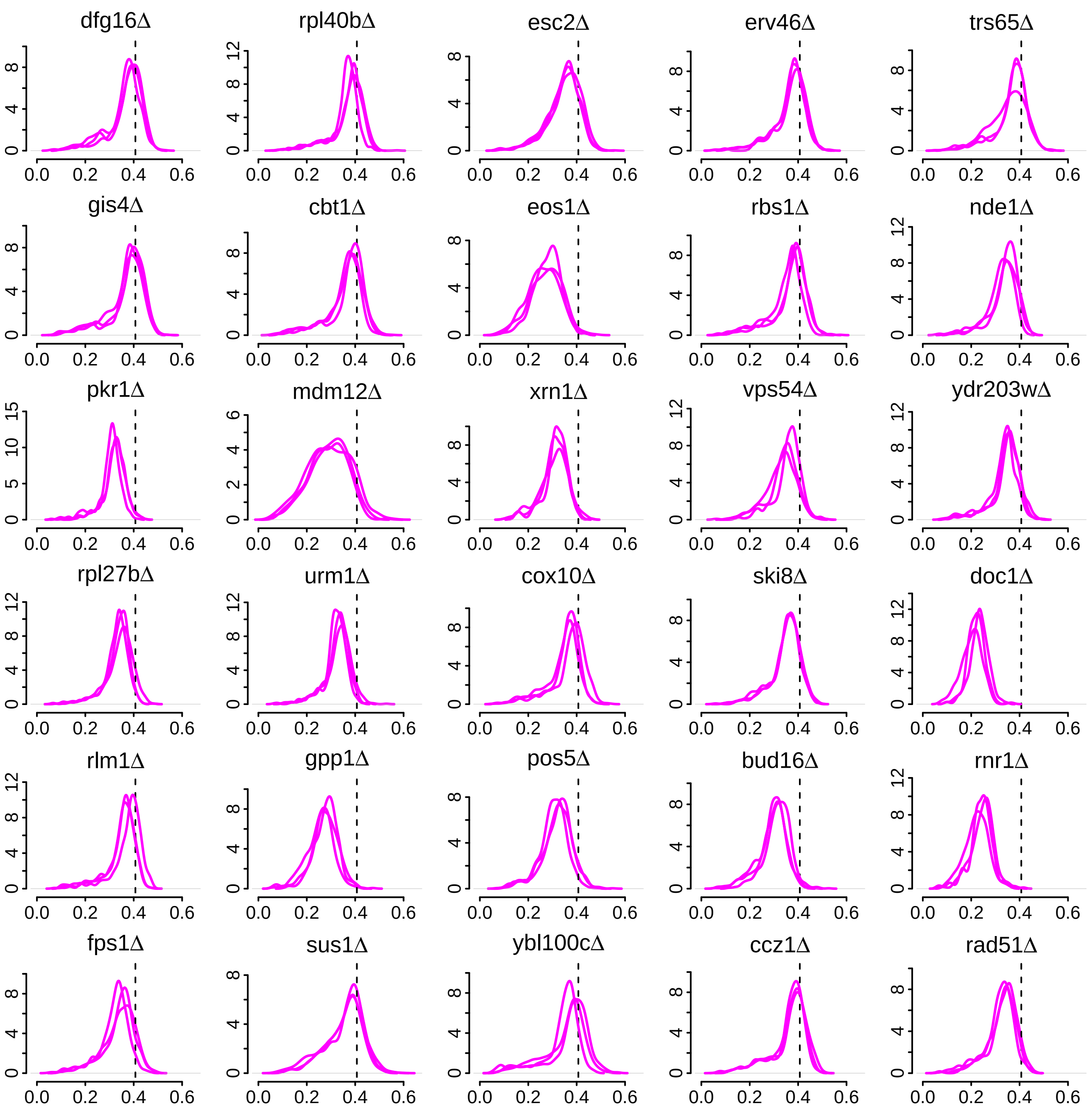
dfg16Δ
rpl40bΔ
esc2Δ
erv46Δ
trs65Δ
gis4Δ
cbt1Δ
eos1Δ
rbs1Δ
nde1Δ
pkr1Δ
mdm12Δ
xrn1Δ
vps54Δ
ydr203wΔ
rpl27bΔ
urm1Δ
cox10Δ
ski8Δ
doc1Δ
rlm1Δ
gpp1Δ
pos5Δ
bud16Δ
rnr1Δ
fps1Δ
sus1Δ
ybl100cΔ
ccz1Δ
rad51Δ
0.0 0.2 0.4 0.6

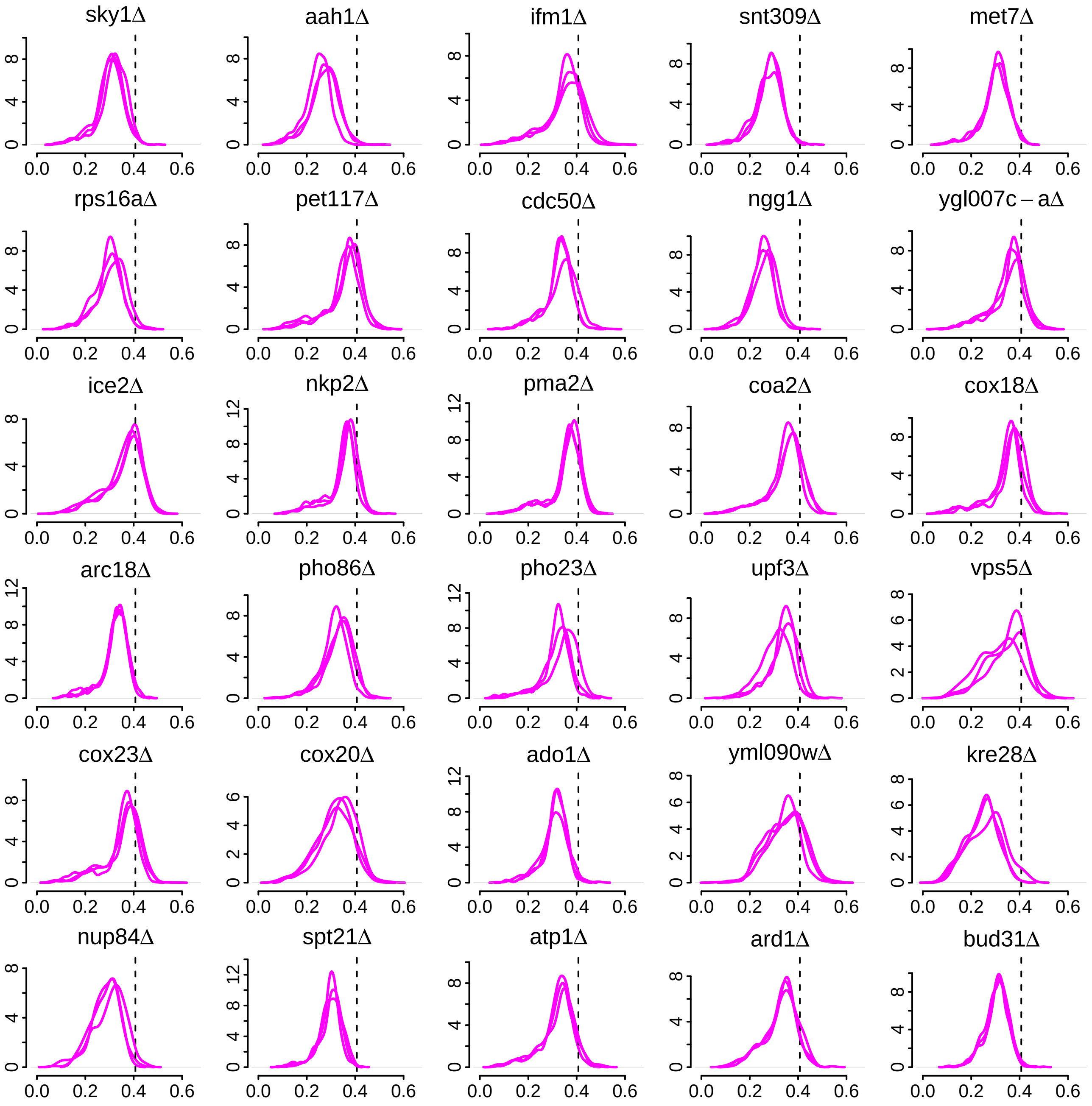
sky1Δ
aah1Δ
ifm1Δ
snt309Δ
met7Δ
rps16aΔ
pet117Δ
cdc50Δ
ngg1Δ
ygl007c – aΔ
ice2Δ
nkp2Δ
pma2Δ
coa2Δ
cox18Δ
arc18Δ
pho86Δ
pho23Δ
upf3Δ
vps5Δ
cox23Δ
cox20Δ
ado1Δ
yml090wΔ
kre28Δ
nup84Δ
spt21Δ
atp1Δ
ard1Δ
bud31Δ
0.0
0.2
0.4
0.6

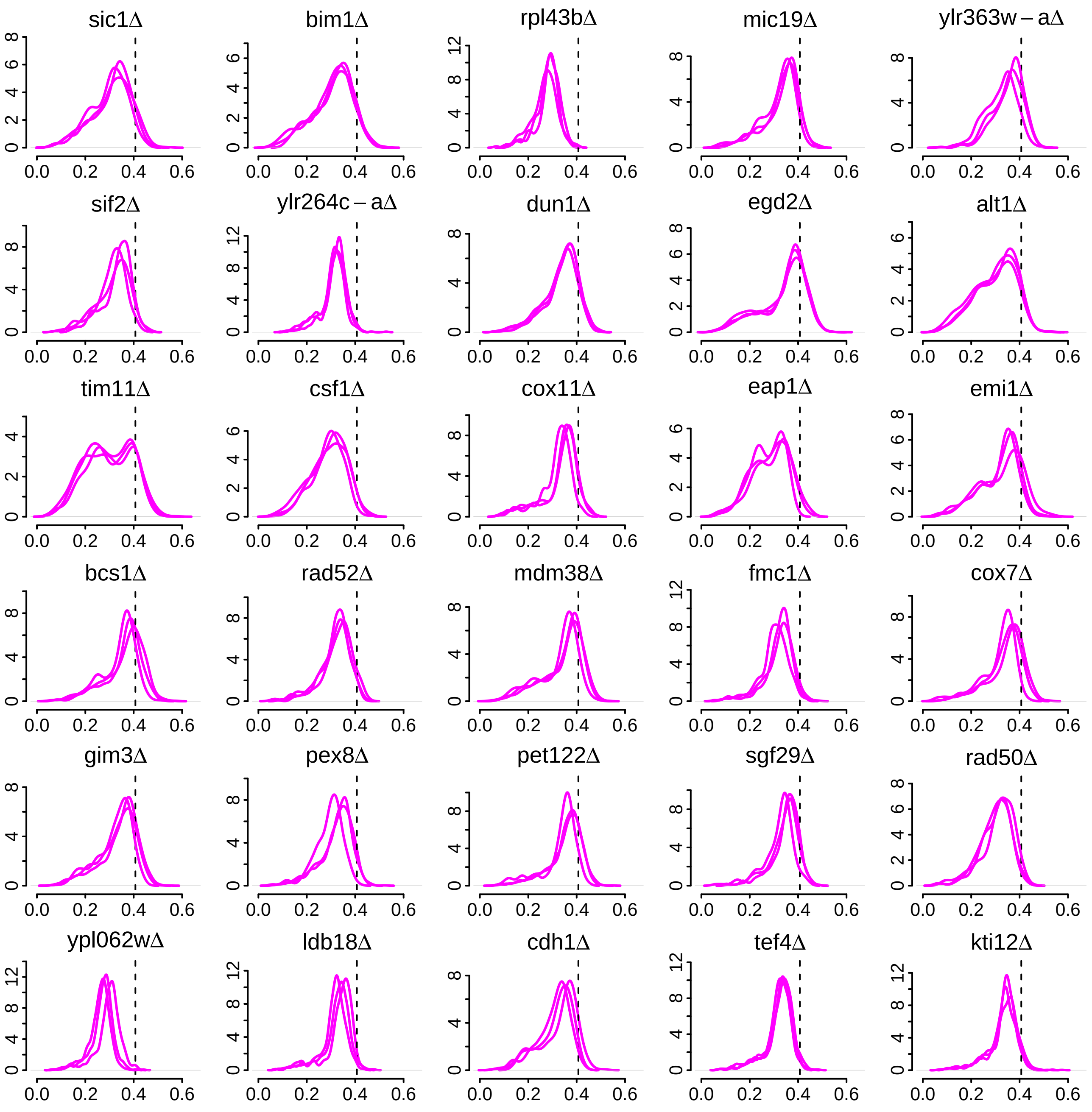
sic1Δ
bim1Δ
rpl43bΔ
mic19Δ
ylr363w – aΔ
sif2Δ
ylr264c – aΔ
dun1Δ
egd2Δ
alt1Δ
tim11Δ
csf1Δ
cox11Δ
eap1Δ
emi1Δ
bcs1Δ
rad52Δ
mdm38Δ
fmc1Δ
cox7Δ
gim3Δ
pex8Δ
pet122Δ
sgf29Δ
rad50Δ
ypl062wΔ
ldb18Δ
cdh1Δ
tef4Δ
kti12Δ
0.0 0.2 0.4 0.6

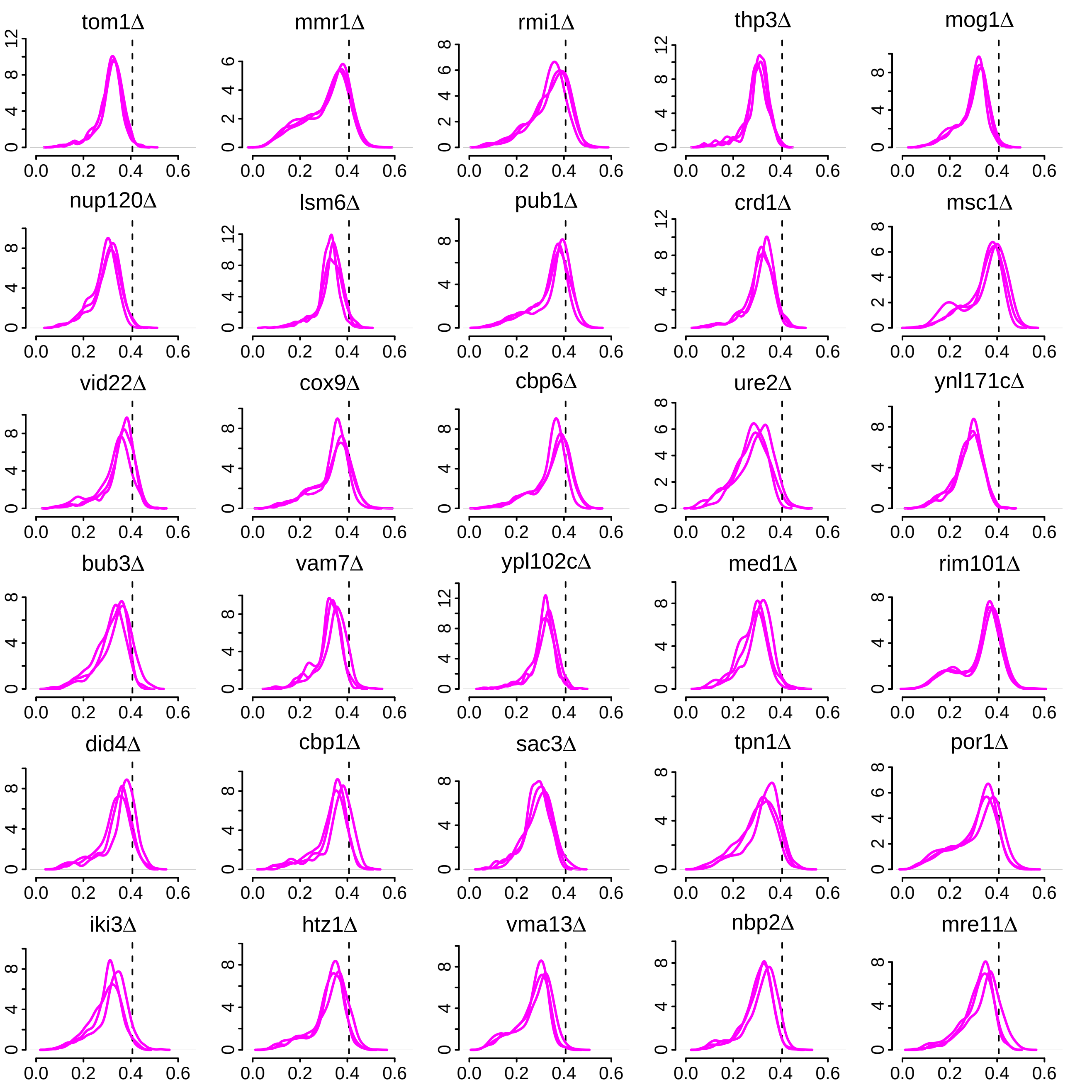
tom1Δ
mmr1Δ
rmi1Δ
thp3Δ
mog1Δ
nup120Δ
lsm6Δ
pub1Δ
crd1Δ
msc1Δ
vid22Δ
cox9Δ
cbp6Δ
ure2Δ
ynl171cΔ
bub3Δ
vam7Δ
ypl102cΔ
med1Δ
rim101Δ
did4Δ
cbp1Δ
sac3Δ
tpn1Δ
por1Δ
iki3Δ
htz1Δ
vma13Δ
nbp2Δ
mre11Δ

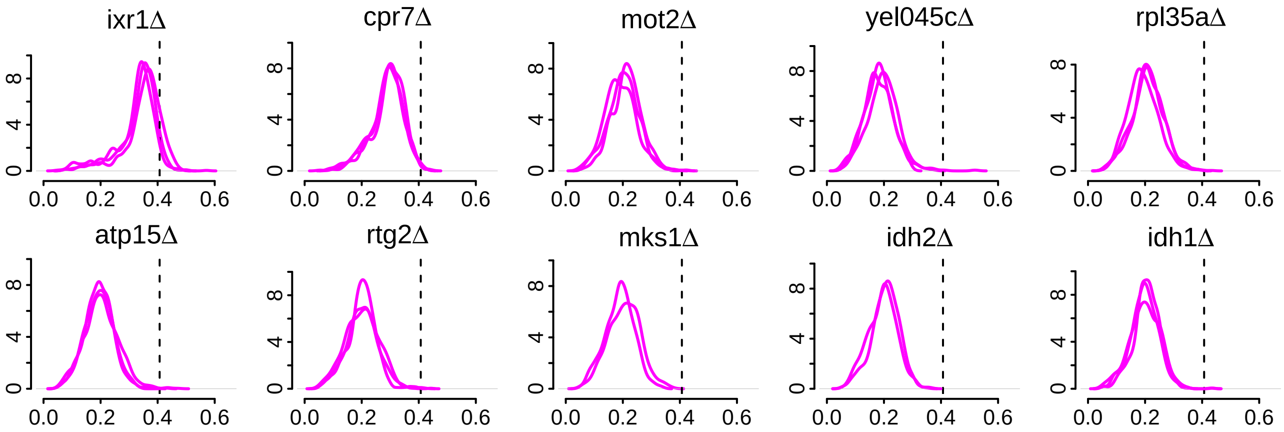

ixr1Δ
cpr7Δ
mot2Δ
yel045cΔ
rpl35aΔ
atp15Δ
rtg2Δ
mks1Δ
idh2Δ
idh1Δ
0
4
8
0.0
0.2
0.4
0.6

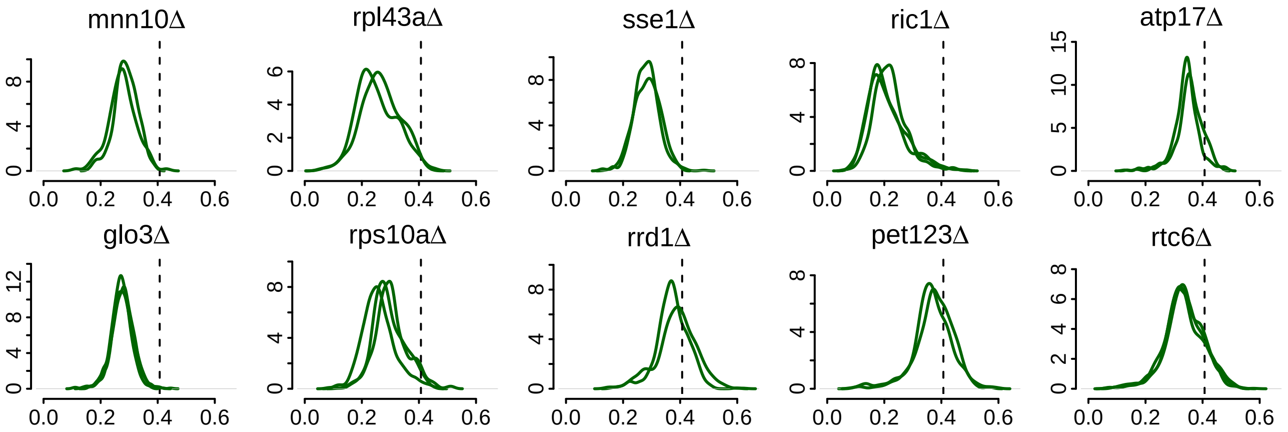

mnn10Δ
rpl43aΔ
sse1Δ
ric1Δ
atp17Δ
glo3Δ
rps10aΔ
rrd1Δ
pet123Δ
rtc6Δ
0.0
0.2
0.4
0.6

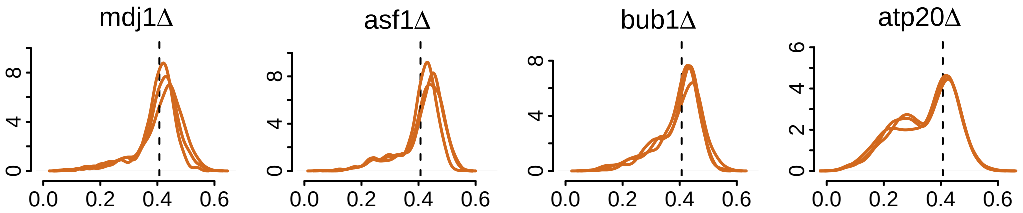
mdj1Δ
asf1Δ
bub1Δ
atp20Δ
0
4
8
2
6
0.0
0.2
0.4
0.6

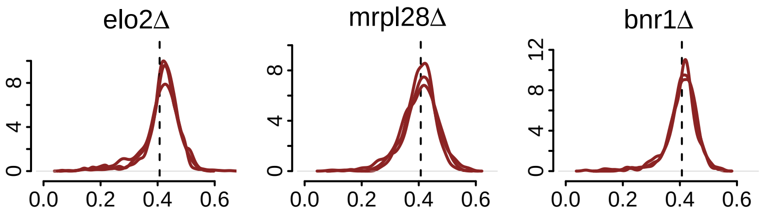

elo2Δ
mrpl28Δ
bnr1Δ
0
4
8
12
0.0
0.2
0.4
0.6

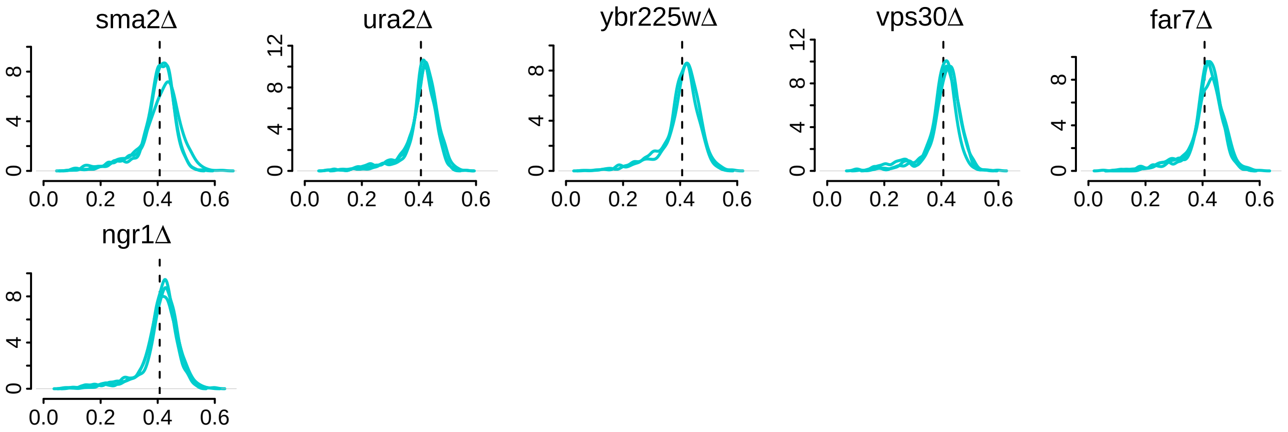

sma2Δ
ura2Δ
ybr225wΔ
vps30Δ
far7Δ
ngr1Δ
0
4
8
12
0.0
0.2
0.4
0.6

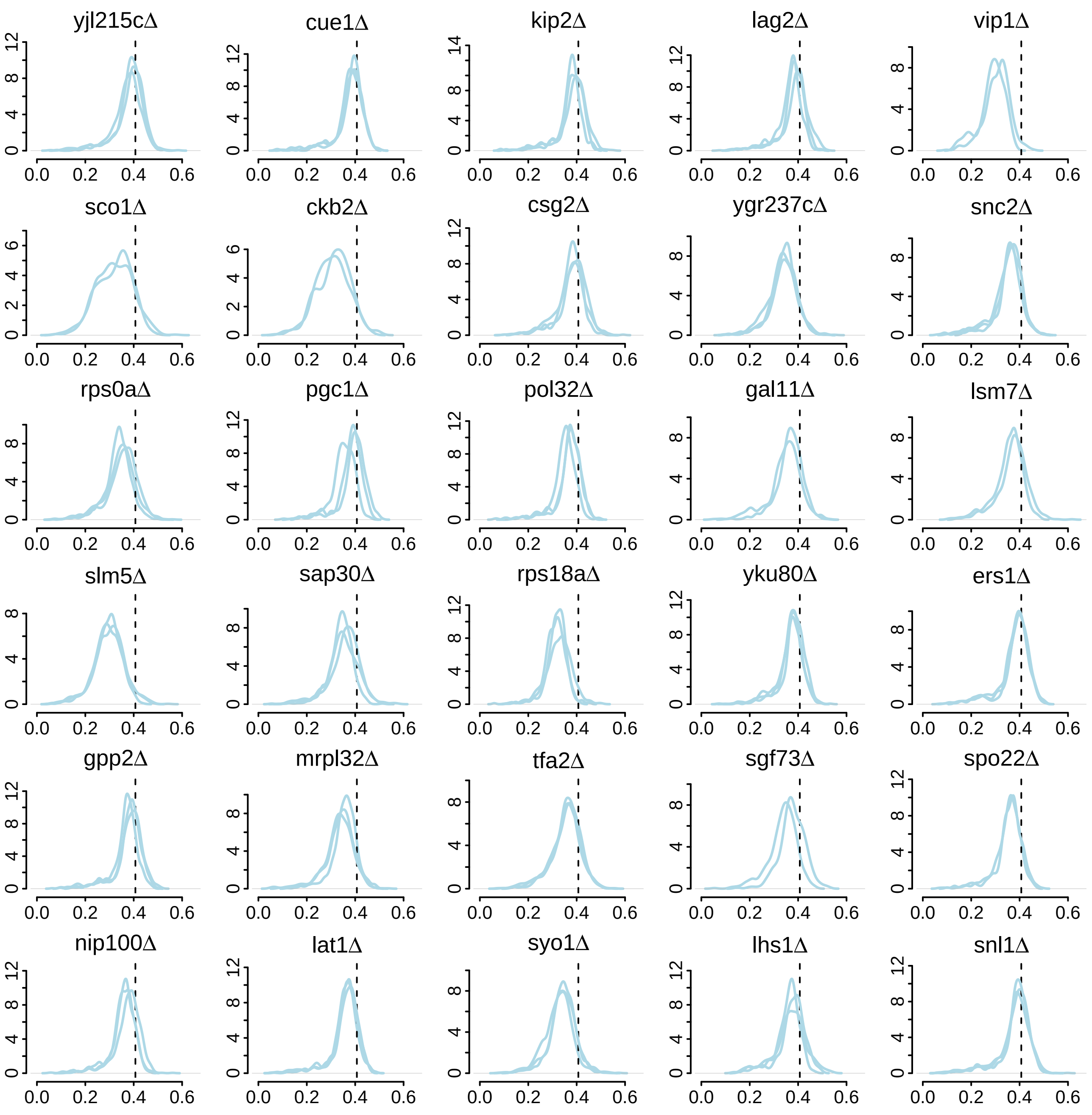
yjl215cΔ
cue1Δ
kip2Δ
lag2Δ
vip1Δ
sco1Δ
ckb2Δ
csg2Δ
ygr237cΔ
snc2Δ
rps0aΔ
pgc1Δ
pol32Δ
gal11Δ
lsm7Δ
slm5Δ
sap30Δ
rps18aΔ
yku80Δ
ers1Δ
gpp2Δ
mrpl32Δ
tfa2Δ
sgf73Δ
spo22Δ
nip100Δ
lat1Δ
syo1Δ
lhs1Δ
snl1Δ
0.0 0.2 0.4 0.6
0 4 8 12

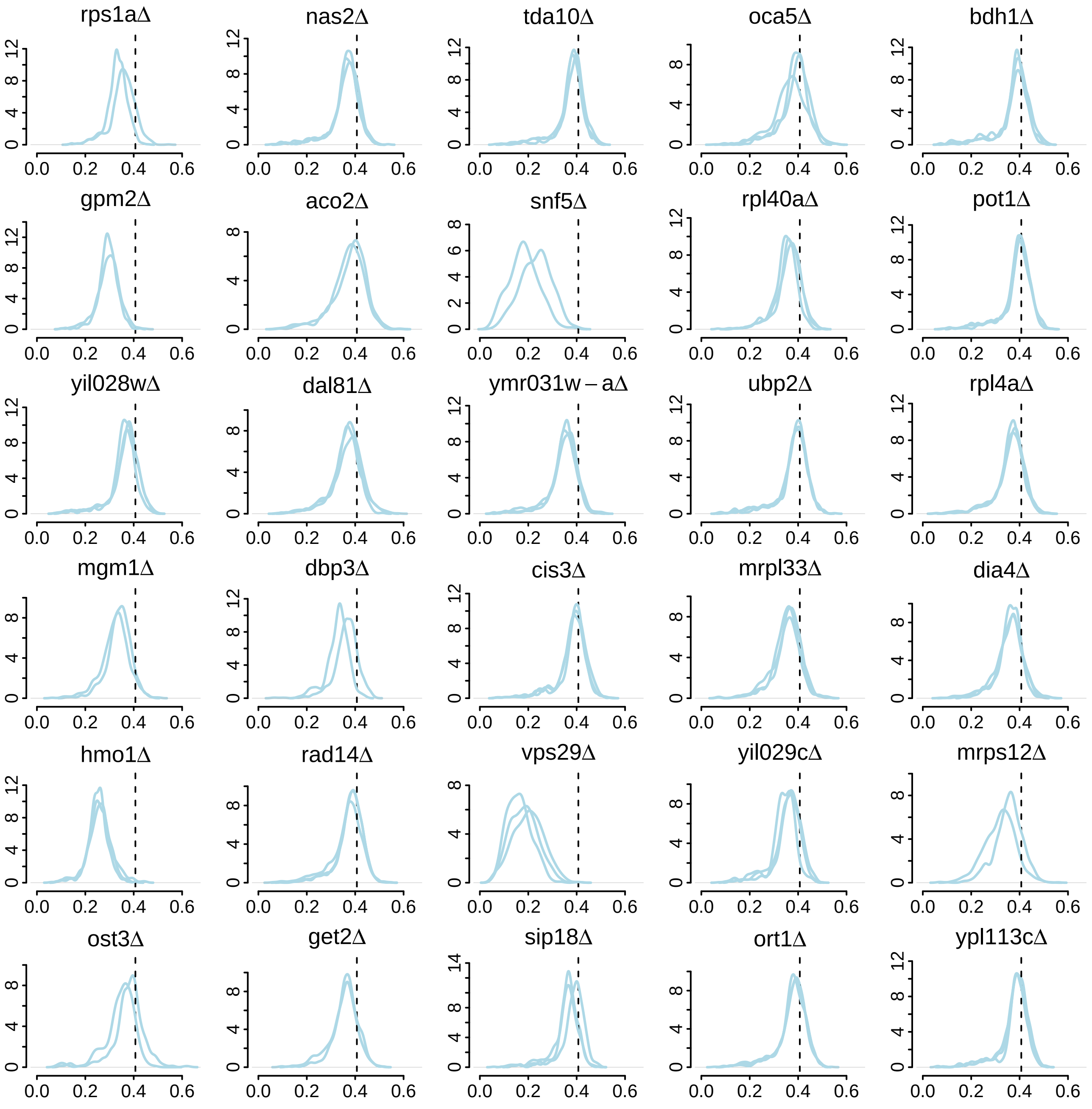
rps1aΔ
nas2Δ
tda10Δ
oca5Δ
bdh1Δ
gpm2Δ
aco2Δ
snf5Δ
rpl40aΔ
pot1Δ
yil028wΔ
dal81Δ
ymr031w – aΔ
ubp2Δ
rpl4aΔ
mgm1Δ
dbp3Δ
cis3Δ
mrpl33Δ
dia4Δ
hmo1Δ
rad14Δ
vps29Δ
yil029cΔ
mrps12Δ
ost3Δ
get2Δ
sip18Δ
ort1Δ
ypl113cΔ

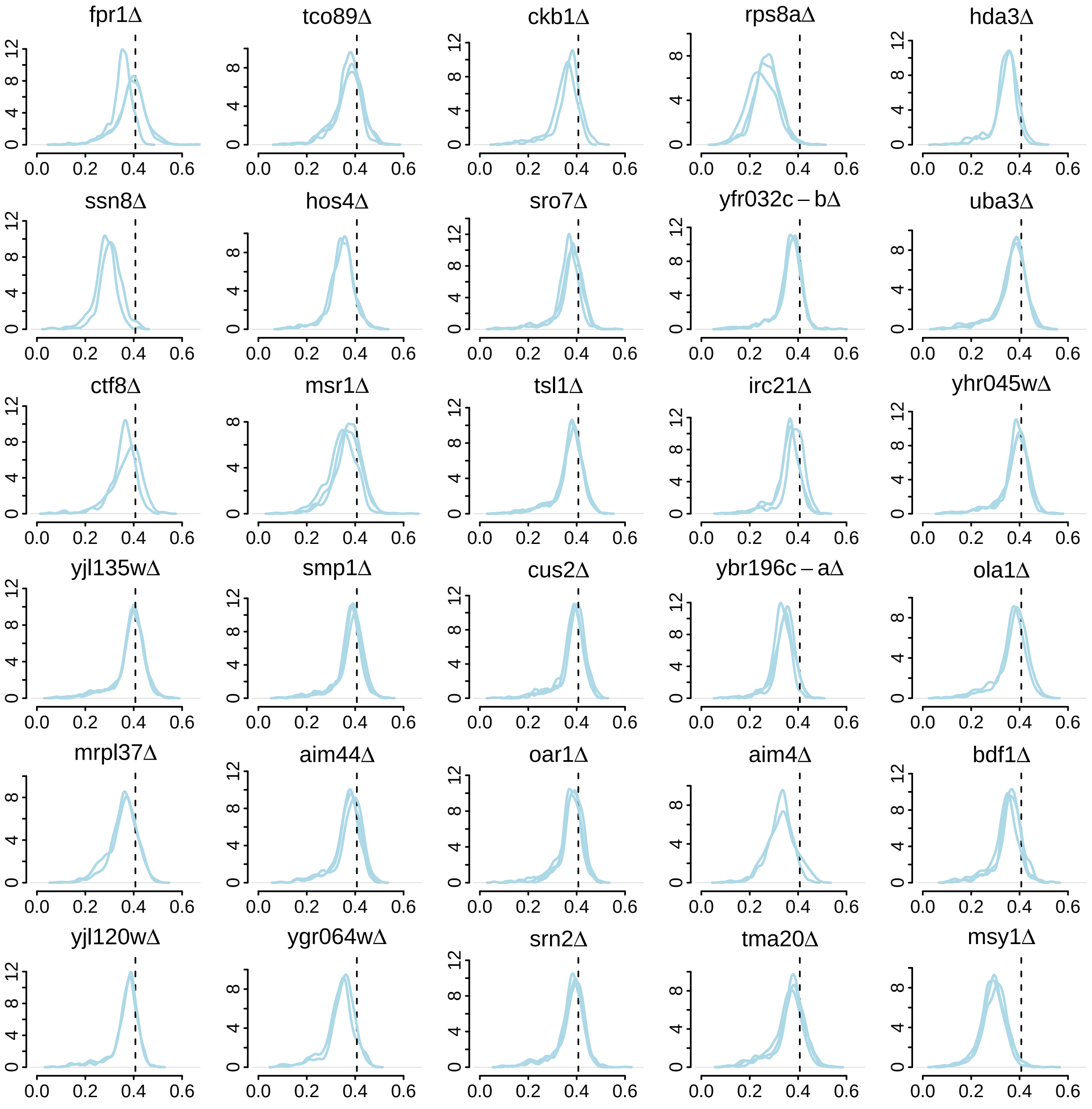
fpr1Δ
tco89Δ
ckb1Δ
rps8aΔ
hda3Δ
ssn8Δ
hos4Δ
sro7Δ
yfr032c – bΔ
uba3Δ
ctf8Δ
msr1Δ
tsl1Δ
irc21Δ
yhr045wΔ
yjl135wΔ
smp1Δ
cus2Δ
ybr196c – aΔ
ola1Δ
mrpl37Δ
aim44Δ
oar1Δ
aim4Δ
bdf1Δ
yjl120wΔ
ygr064wΔ
srn2Δ
tma20Δ
msy1Δ
0.0
0.2
0.4
0.6
0
4
8
12

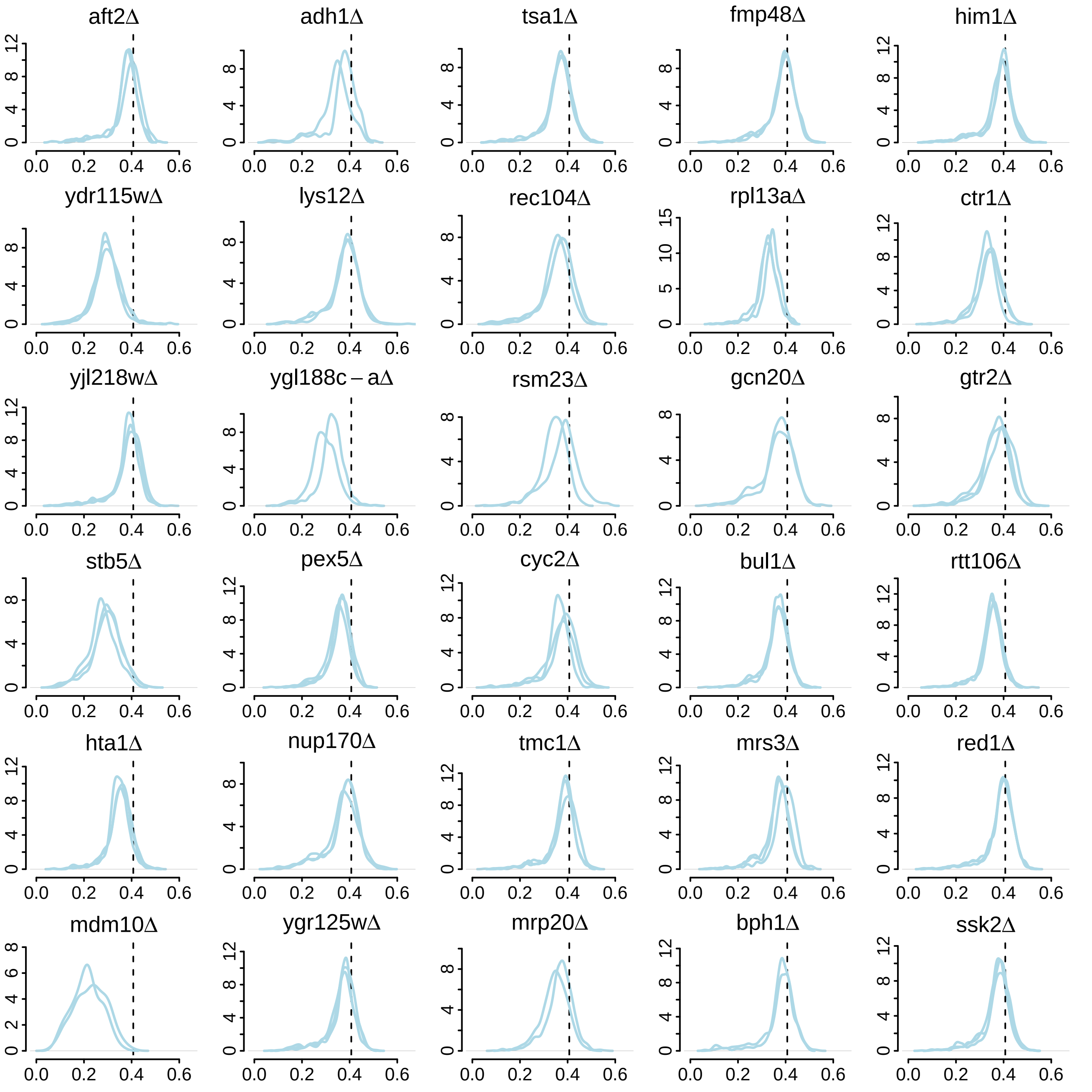
aft2Δ
adh1Δ
tsa1Δ
fmp48Δ
him1Δ
ydr115wΔ
lys12Δ
rec104Δ
rpl13aΔ
ctr1Δ
yjl218wΔ
ygl188c – aΔ
rsm23Δ
gcn20Δ
gtr2Δ
stb5Δ
pex5Δ
cyc2Δ
bul1Δ
rtt106Δ
hta1Δ
nup170Δ
tmc1Δ
mrs3Δ
red1Δ
mdm10Δ
ygr125wΔ
mrp20Δ
bph1Δ
ssk2Δ
0.0 0.2 0.4 0.6

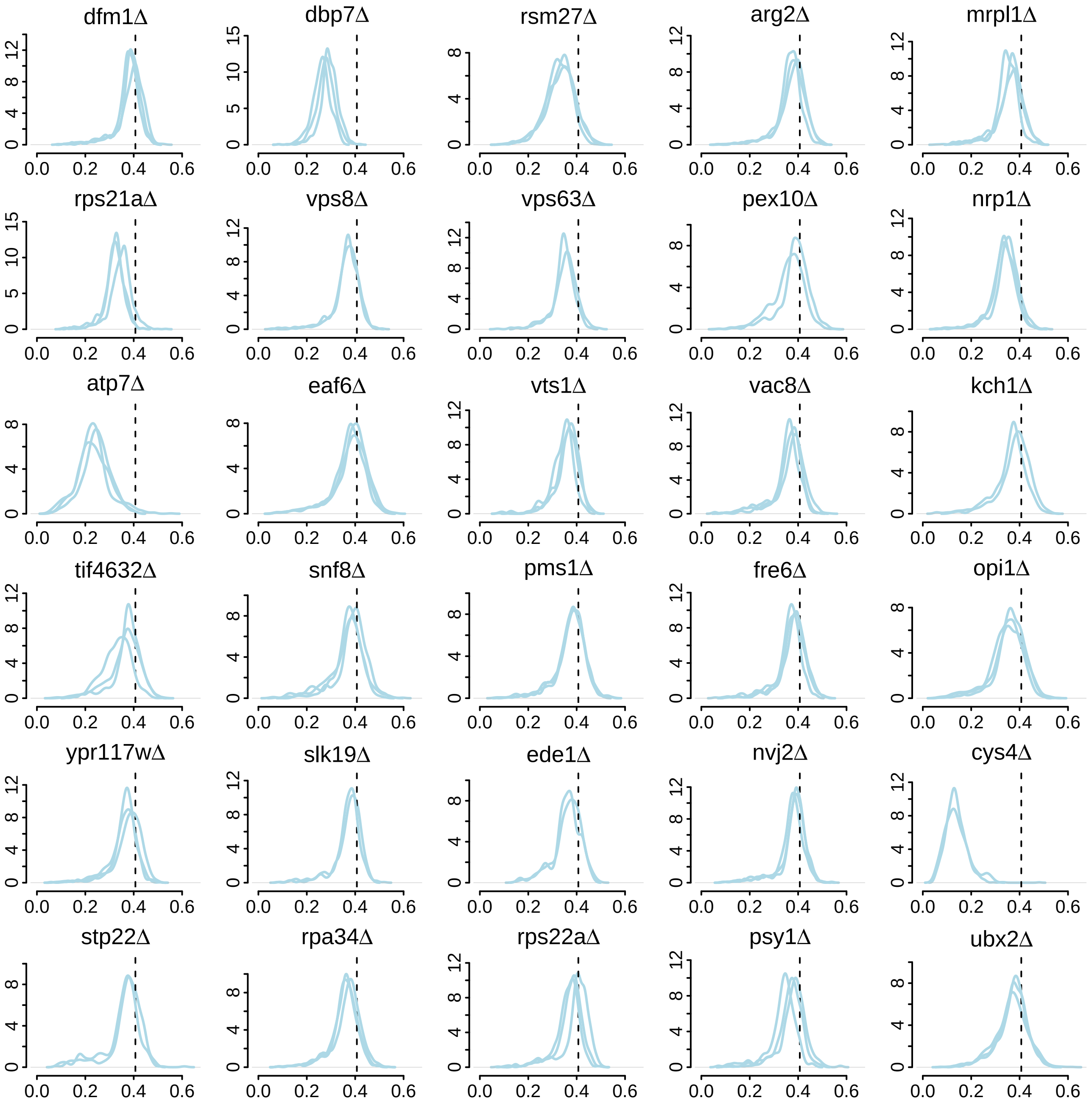

dfm1Δ
dbp7Δ
rsm27Δ
arg2Δ
mrpl1Δ
rps21aΔ
vps8Δ
vps63Δ
pex10Δ
nrp1Δ
atp7Δ
eaf6Δ
vts1Δ
vac8Δ
kch1Δ
tif4632Δ
snf8Δ
pms1Δ
fre6Δ
opi1Δ
ypr117wΔ
slk19Δ
ede1Δ
nvj2Δ
cys4Δ
stp22Δ
rpa34Δ
rps22aΔ
psy1Δ
ubx2Δ

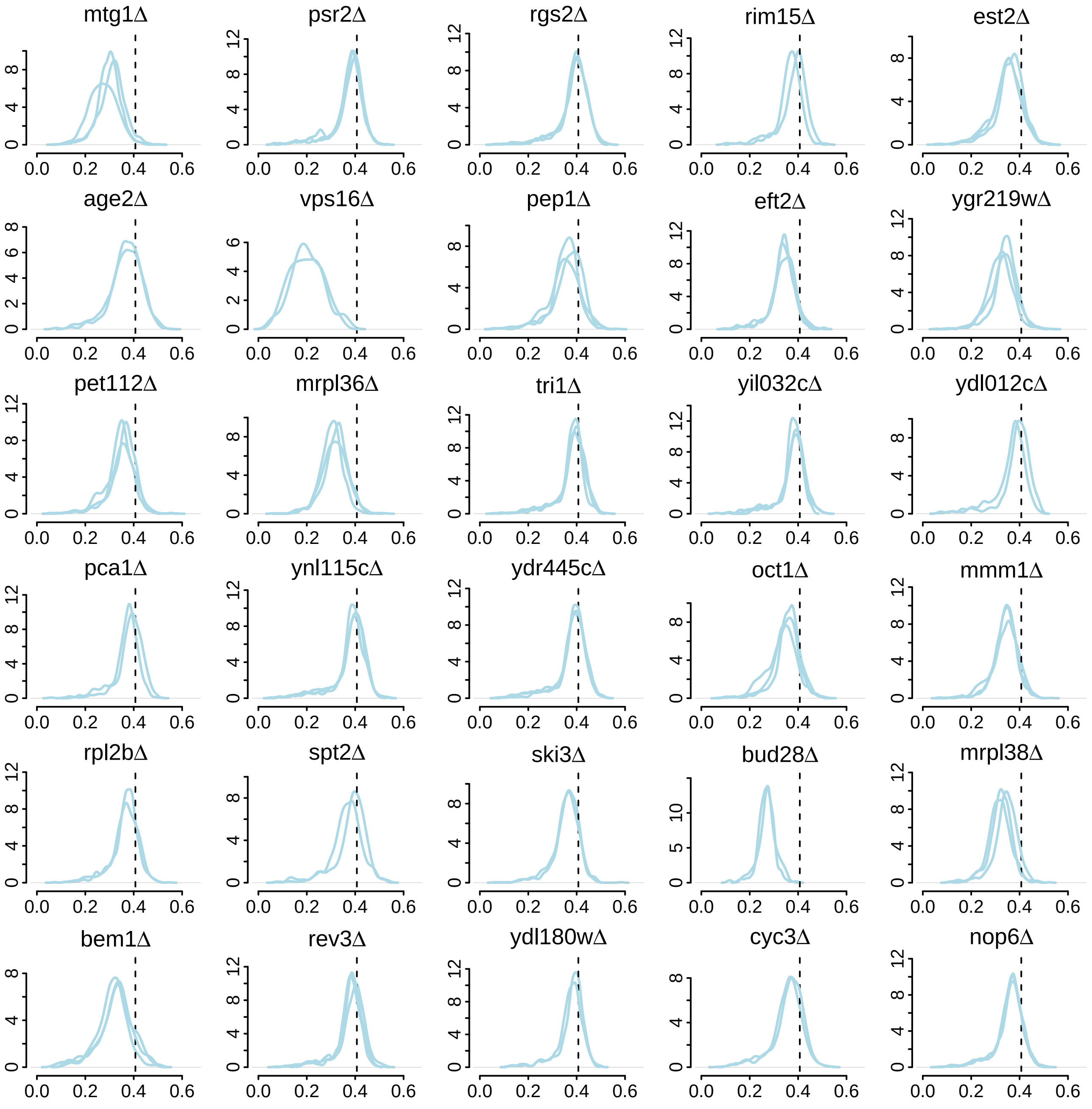
mtg1Δ
psr2Δ
rgs2Δ
rim15Δ
est2Δ
age2Δ
vps16Δ
pep1Δ
eft2Δ
ygr219wΔ
pet112Δ
mrpl36Δ
tri1Δ
yil032cΔ
ydl012cΔ
pca1Δ
ynl115cΔ
ydr445cΔ
oct1Δ
mmm1Δ
rpl2bΔ
spt2Δ
ski3Δ
bud28Δ
mrpl38Δ
bem1Δ
rev3Δ
ydl180wΔ
cyc3Δ
nop6Δ
0.0 0.2 0.4 0.6

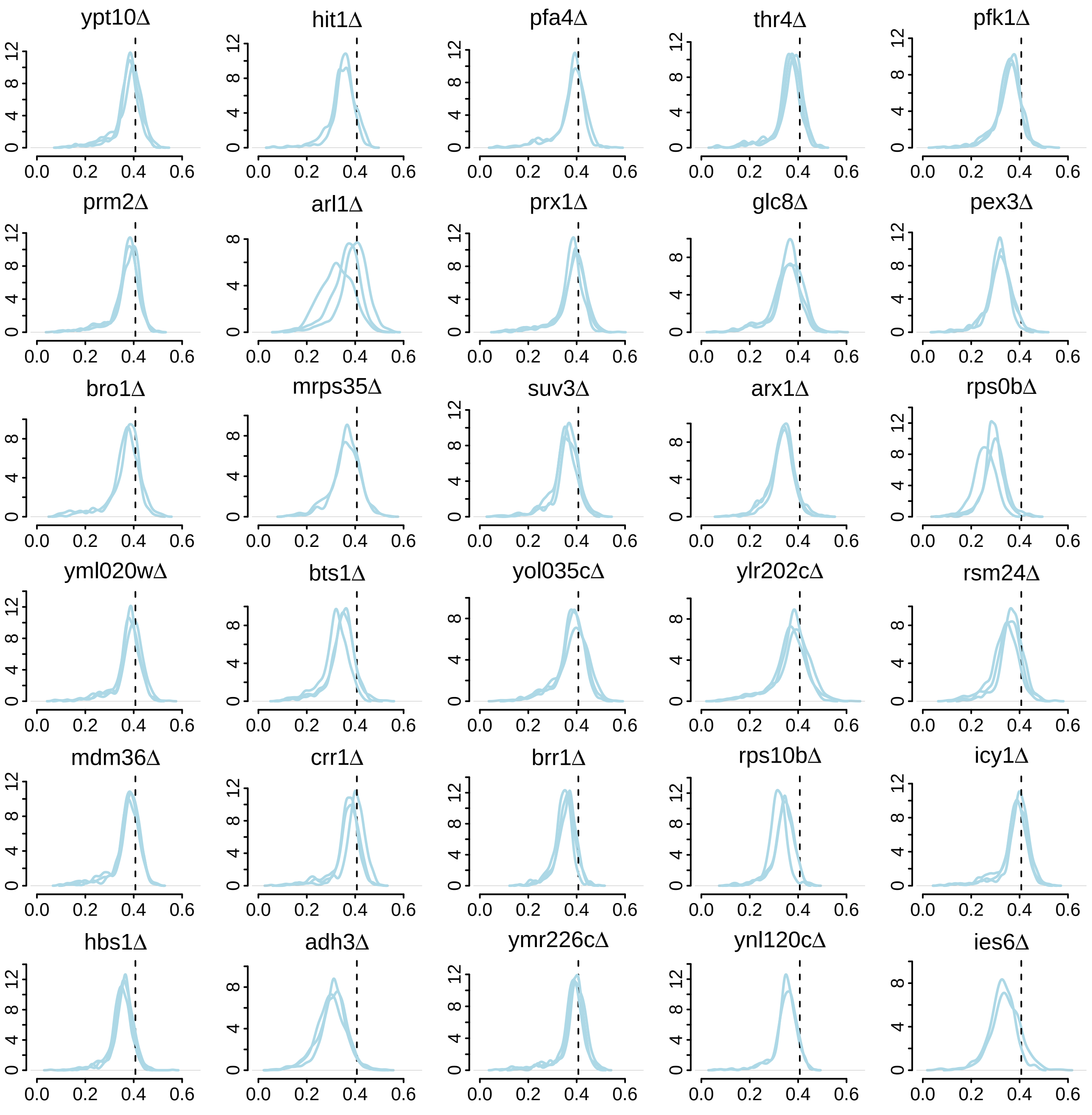
ypt10Δ
hit1Δ
pfa4Δ
thr4Δ
pfk1Δ
prm2Δ
arl1Δ
prx1Δ
glc8Δ
pex3Δ
bro1Δ
mrps35Δ
suv3Δ
arx1Δ
rps0bΔ
yml020wΔ
bts1Δ
yol035cΔ
ylr202cΔ
rsm24Δ
mdm36Δ
crr1Δ
brr1Δ
rps10bΔ
icy1Δ
hbs1Δ
adh3Δ
ymr226cΔ
ynl120cΔ
ies6Δ

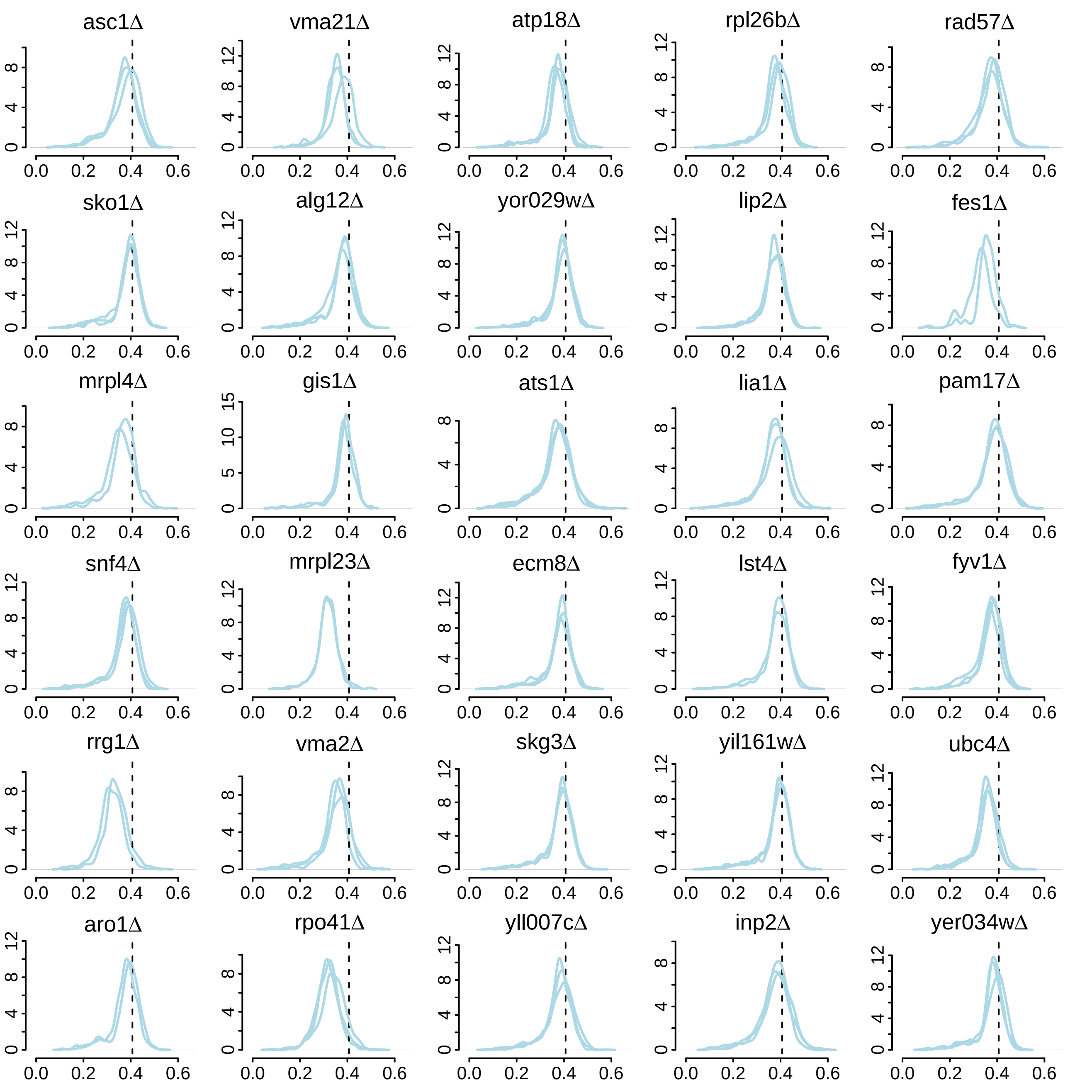

asc1Δ
vma21Δ
atp18Δ
rpl26bΔ
rad57Δ
sko1Δ
alg12Δ
yor029wΔ
lip2Δ
fes1Δ
mrpl4Δ
gis1Δ
ats1Δ
lia1Δ
pam17Δ
snf4Δ
mrpl23Δ
ecm8Δ
lst4Δ
fyv1Δ
rrg1Δ
vma2Δ
skg3Δ
yil161wΔ
ubc4Δ
aro1Δ
rpo41Δ
yll007cΔ
inp2Δ
yer034wΔ
0.0
0.2
0.4
0.6

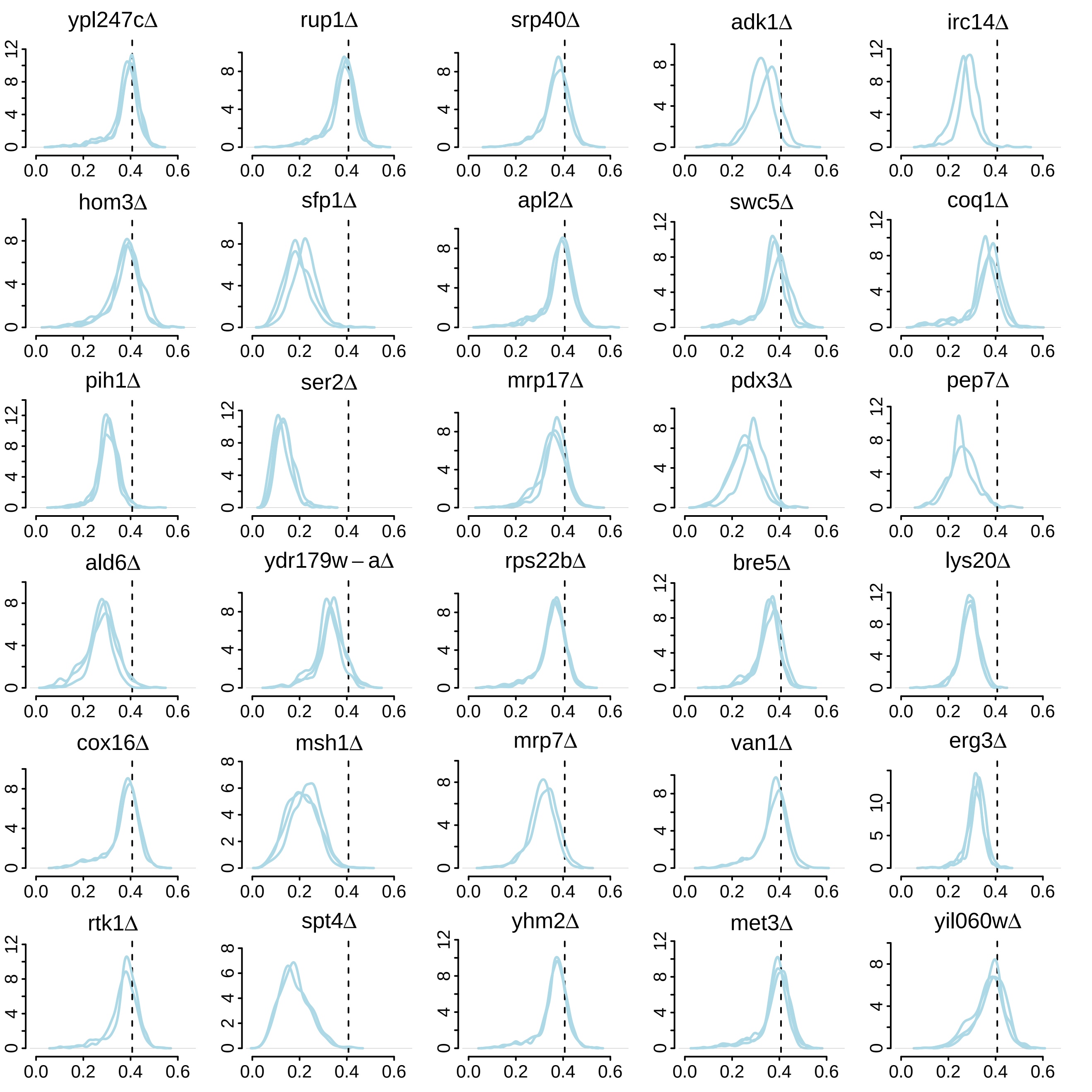
ypl247cΔ
rup1Δ
srp40Δ
adk1Δ
irc14Δ
hom3Δ
sfp1Δ
apl2Δ
swc5Δ
coq1Δ
pih1Δ
ser2Δ
mrp17Δ
pdx3Δ
pep7Δ
ald6Δ
ydr179w – aΔ
rps22bΔ
bre5Δ
lys20Δ
cox16Δ
msh1Δ
mrp7Δ
van1Δ
erg3Δ
rtk1Δ
spt4Δ
yhm2Δ
met3Δ
yil060wΔ
0.0
0.2
0.4
0.6

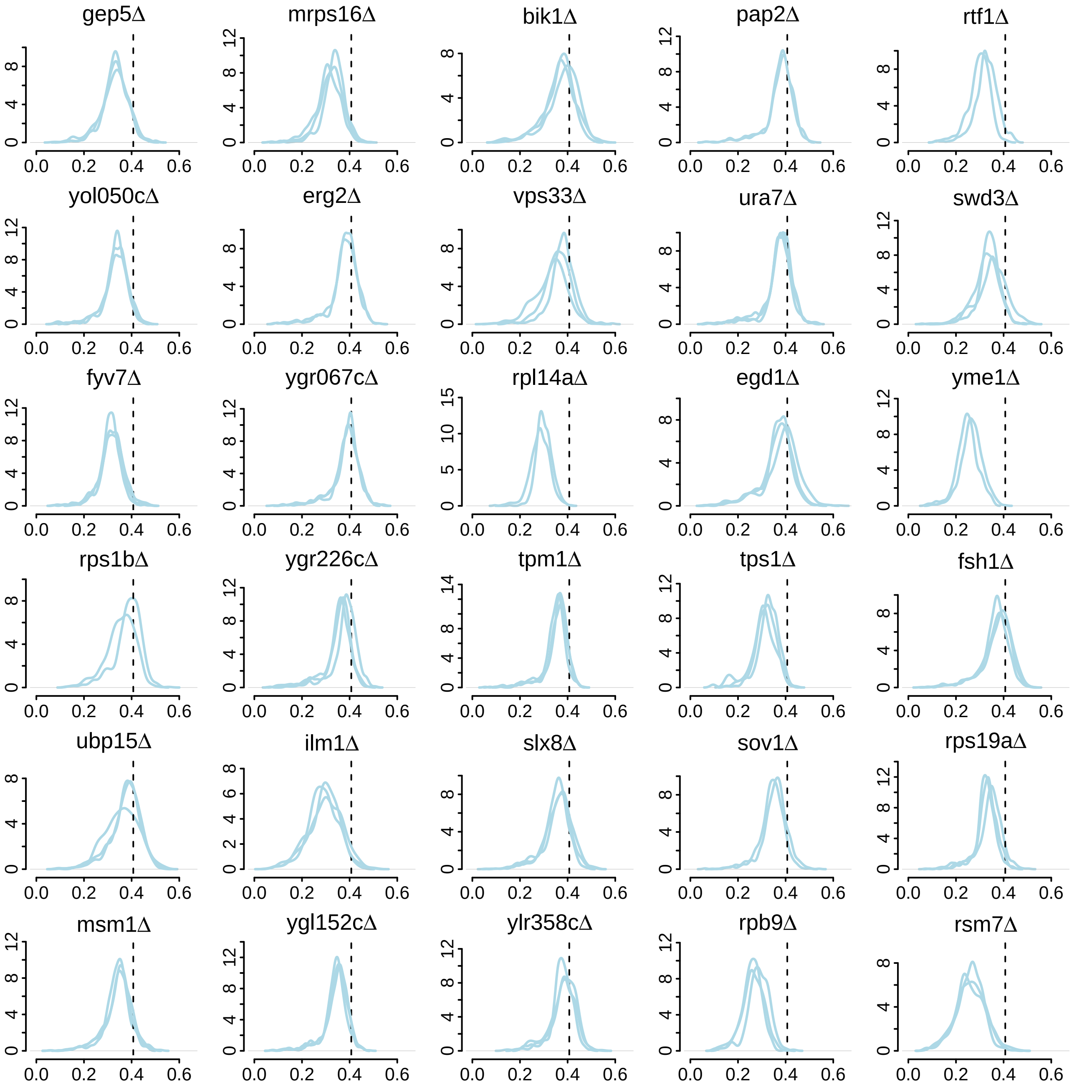
gep5Δ
mrps16Δ
bik1Δ
pap2Δ
rtf1Δ
yol050cΔ
erg2Δ
vps33Δ
ura7Δ
swd3Δ
fyv7Δ
ygr067cΔ
rpl14aΔ
egd1Δ
yme1Δ
rps1bΔ
ygr226cΔ
tpm1Δ
tps1Δ
fsh1Δ
ubp15Δ
ilm1Δ
slx8Δ
sov1Δ
rps19aΔ
msm1Δ
ygl152cΔ
ylr358cΔ
rpb9Δ
rsm7Δ

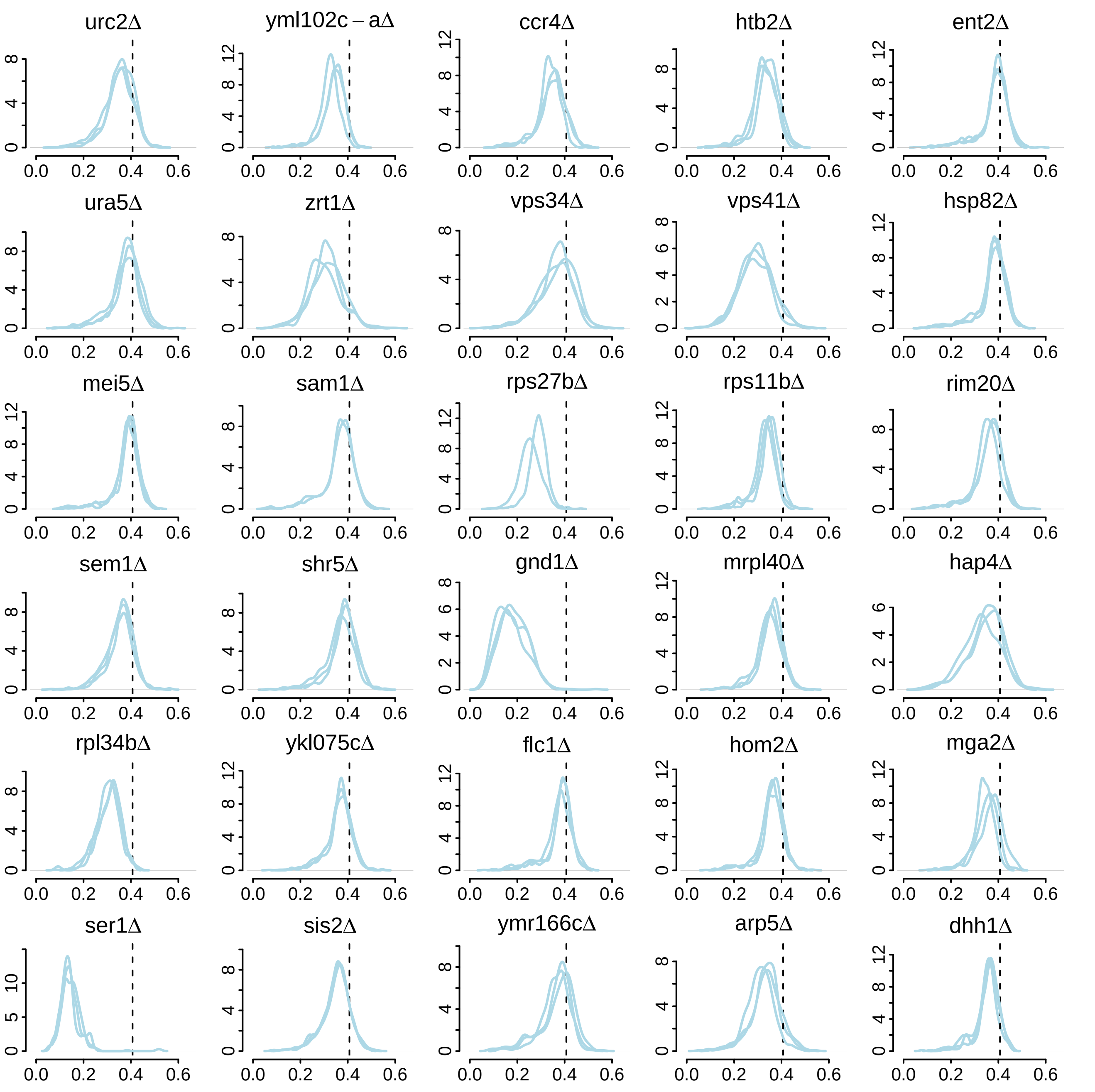

urc2Δ
yml102c – aΔ
ccr4Δ
htb2Δ
ent2Δ
ura5Δ
zrt1Δ
vps34Δ
vps41Δ
hsp82Δ
mei5Δ
sam1Δ
rps27bΔ
rps11bΔ
rim20Δ
sem1Δ
shr5Δ
gnd1Δ
mrpl40Δ
hap4Δ
rpl34bΔ
ykl075cΔ
flc1Δ
hom2Δ
mga2Δ
ser1Δ
sis2Δ
ymr166cΔ
arp5Δ
dhh1Δ

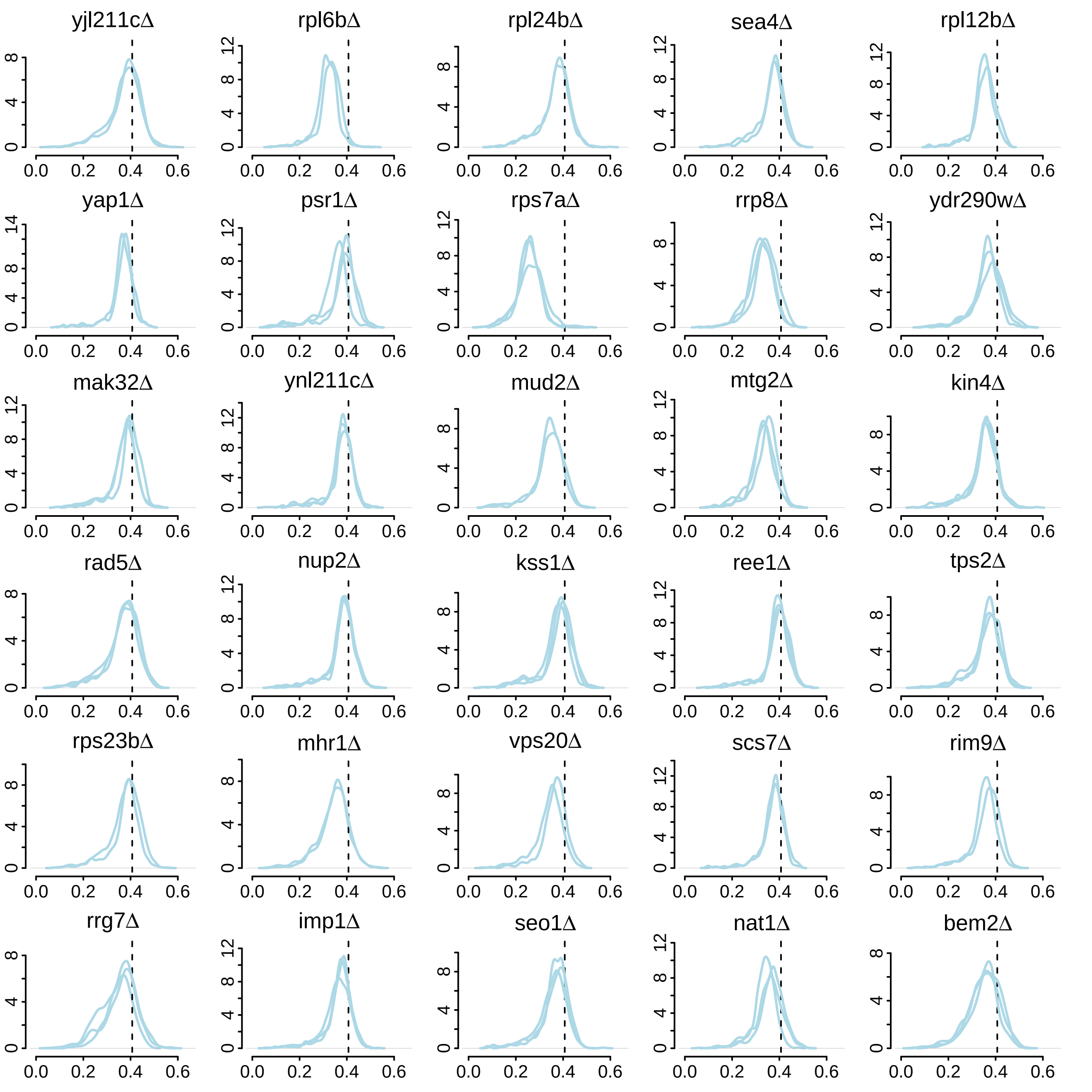
yjl211cΔ
rpl6bΔ
rpl24bΔ
sea4Δ
rpl12bΔ
yap1Δ
psr1Δ
rps7aΔ
rrp8Δ
ydr290wΔ
mak32Δ
ynl211cΔ
mud2Δ
mtg2Δ
kin4Δ
rad5Δ
nup2Δ
kss1Δ
ree1Δ
tps2Δ
rps23bΔ
mhr1Δ
vps20Δ
scs7Δ
rim9Δ
rrg7Δ
imp1Δ
seo1Δ
nat1Δ
bem2Δ

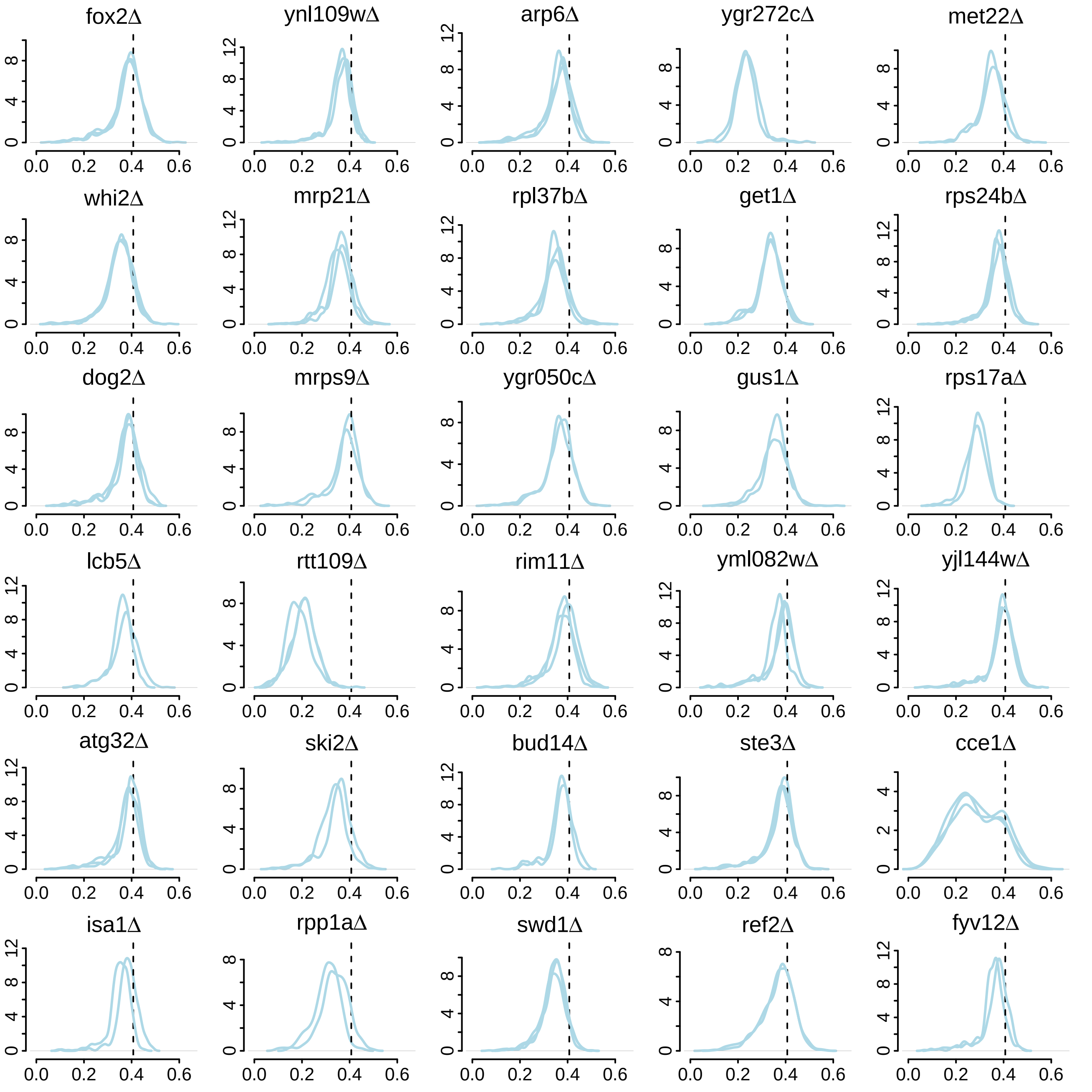

fox2Δ
ynl109wΔ
arp6Δ
ygr272cΔ
met22Δ
whi2Δ
mrp21Δ
rpl37bΔ
get1Δ
rps24bΔ
dog2Δ
mrps9Δ
ygr050cΔ
gus1Δ
rps17aΔ
lcb5Δ
rtt109Δ
rim11Δ
yml082wΔ
yjl144wΔ
atg32Δ
ski2Δ
bud14Δ
ste3Δ
cce1Δ
isa1Δ
rpp1aΔ
swd1Δ
ref2Δ
fyv12Δ

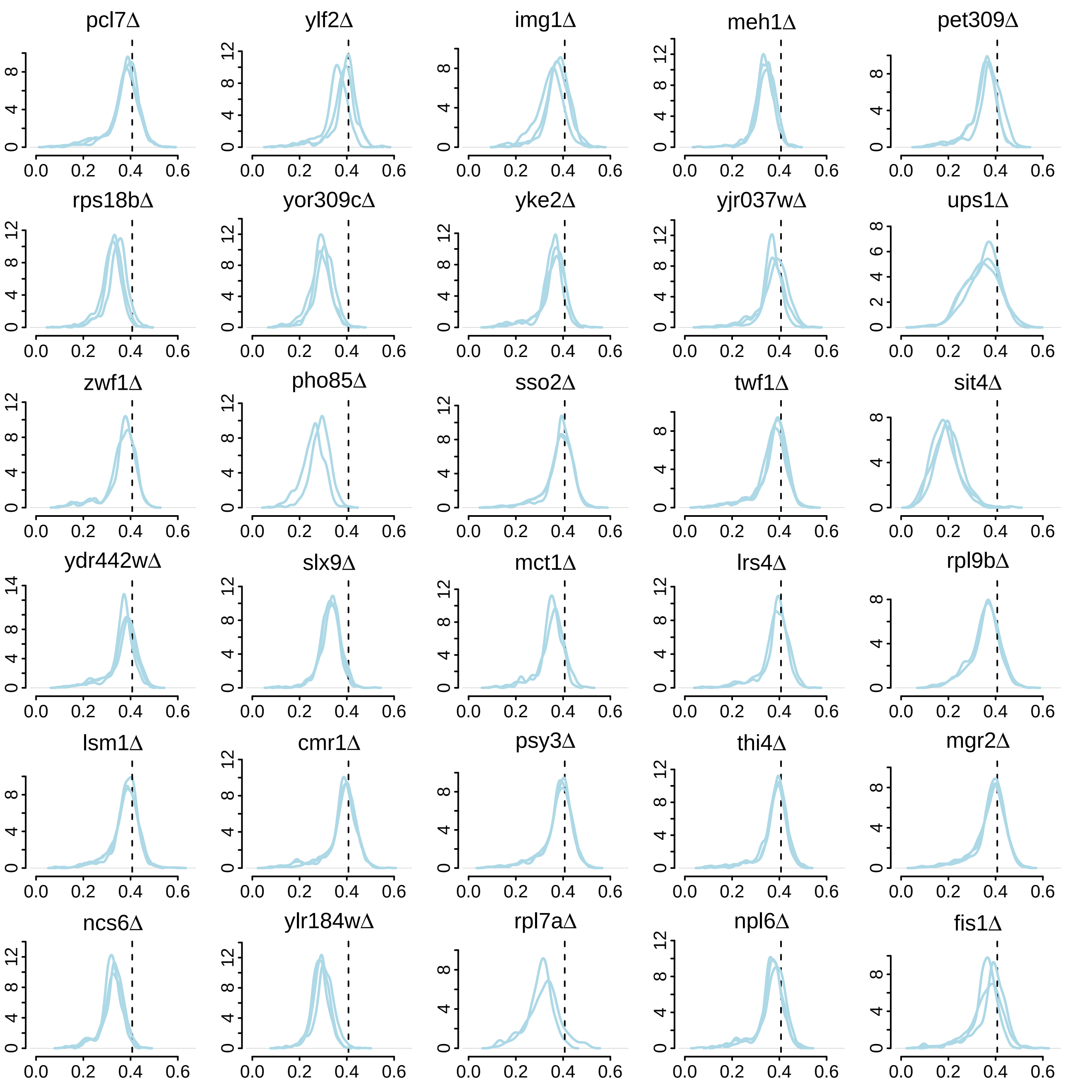
pcl7Δ
ylf2Δ
img1Δ
meh1Δ
pet309Δ
rps18bΔ
yor309cΔ
yke2Δ
yjr037wΔ
ups1Δ
zwf1Δ
pho85Δ
sso2Δ
twf1Δ
sit4Δ
ydr442wΔ
slx9Δ
mct1Δ
lrs4Δ
rpl9bΔ
lsm1Δ
cmr1Δ
psy3Δ
thi4Δ
mgr2Δ
ncs6Δ
ylr184wΔ
rpl7aΔ
npl6Δ
fis1Δ

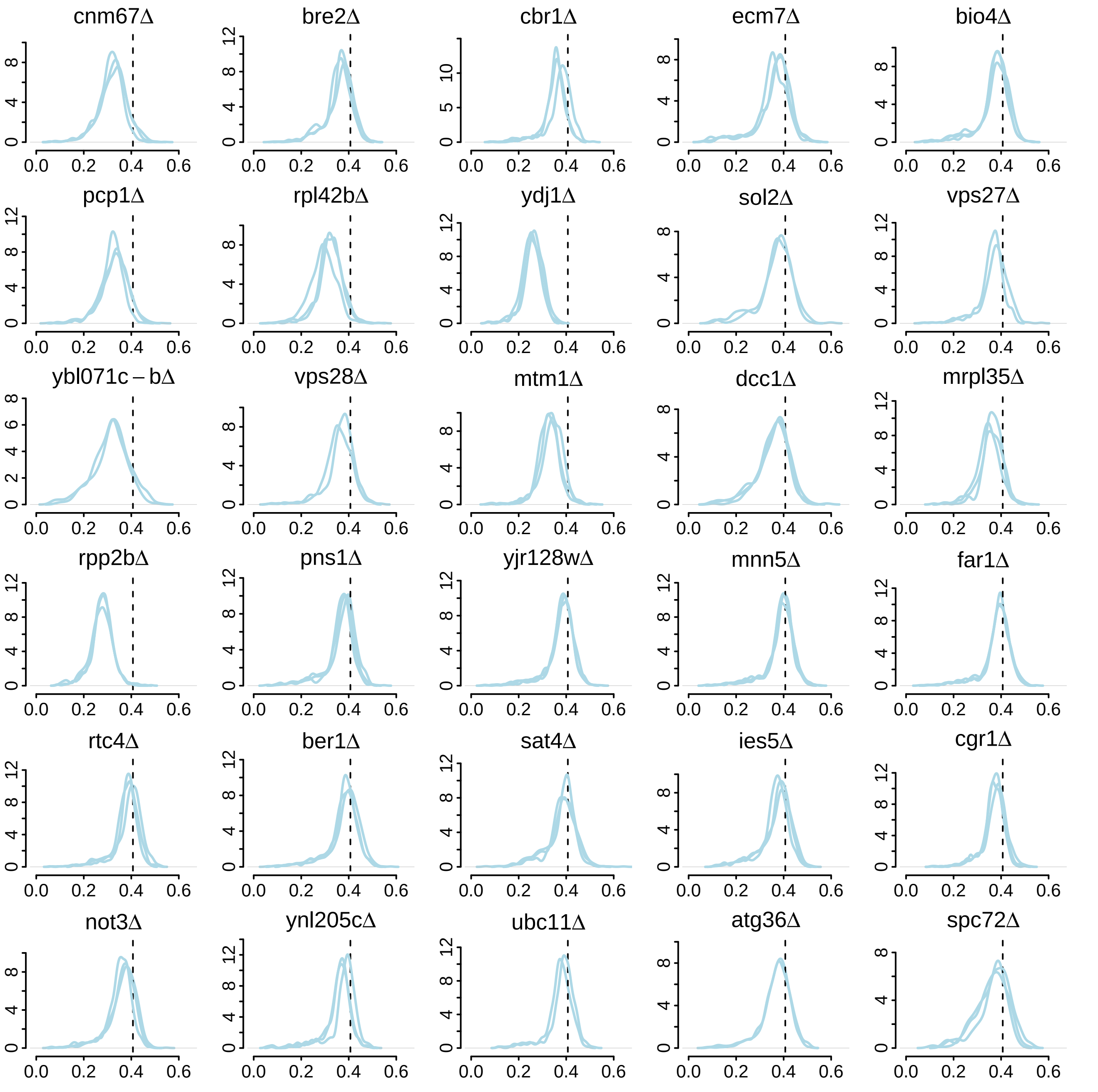
cnm67Δ
bre2Δ
cbr1Δ
ecm7Δ
bio4Δ
pcp1Δ
rpl42bΔ
ydj1Δ
sol2Δ
vps27Δ
ybl071c – bΔ
vps28Δ
mtm1Δ
dcc1Δ
mrpl35Δ
rpp2bΔ
pns1Δ
yjr128wΔ
mnn5Δ
far1Δ
rtc4Δ
ber1Δ
sat4Δ
ies5Δ
cgr1Δ
not3Δ
ynl205cΔ
ubc11Δ
atg36Δ
spc72Δ

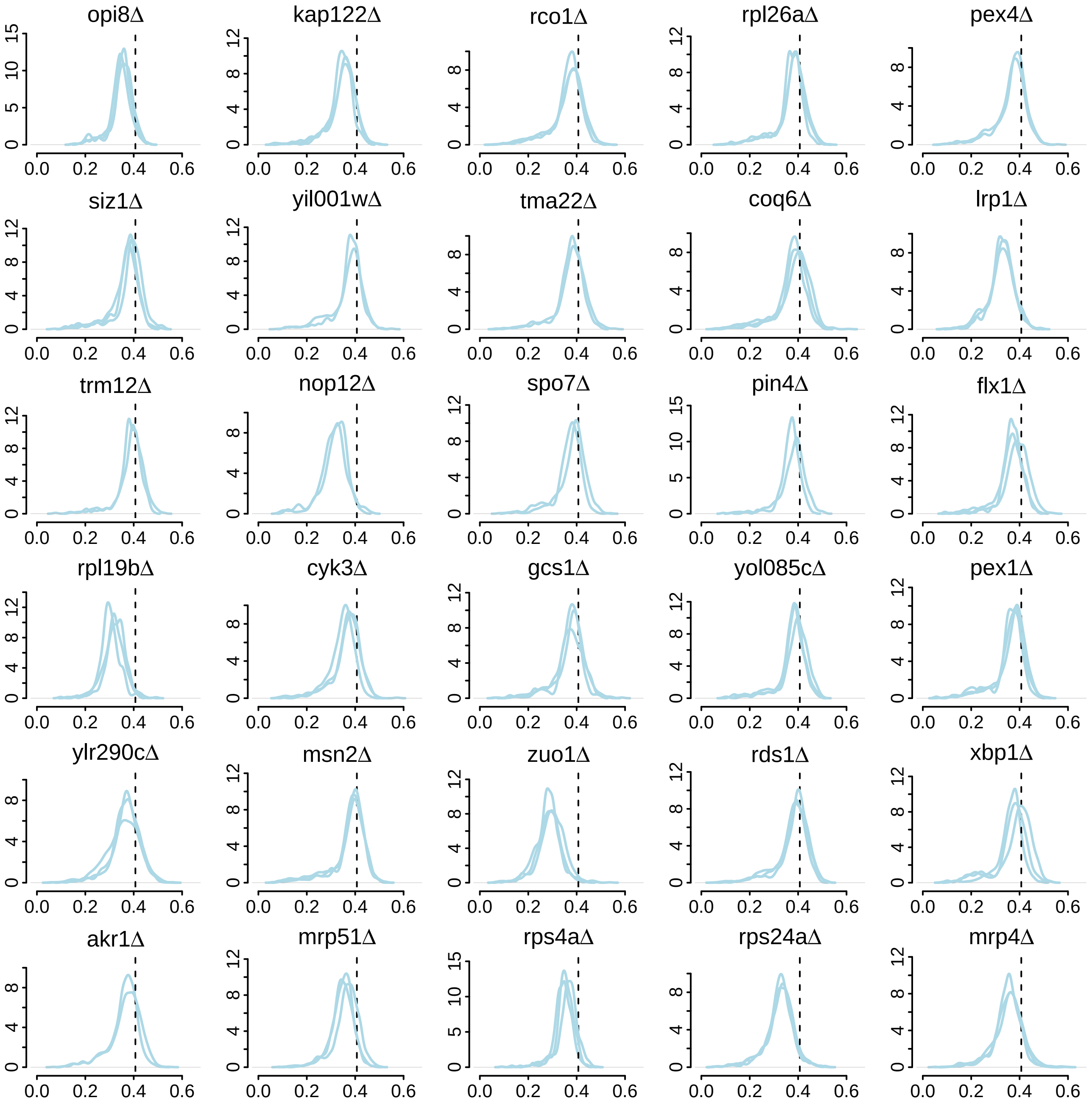
opi8Δ
kap122Δ
rco1Δ
rpl26aΔ
pex4Δ
siz1Δ
yil001wΔ
tma22Δ
coq6Δ
lrp1Δ
trm12Δ
nop12Δ
spo7Δ
pin4Δ
flx1Δ
rpl19bΔ
cyk3Δ
gcs1Δ
yol085cΔ
pex1Δ
ylr290cΔ
msn2Δ
zuo1Δ
rds1Δ
xbp1Δ
akr1Δ
mrp51Δ
rps4aΔ
rps24aΔ
mrp4Δ

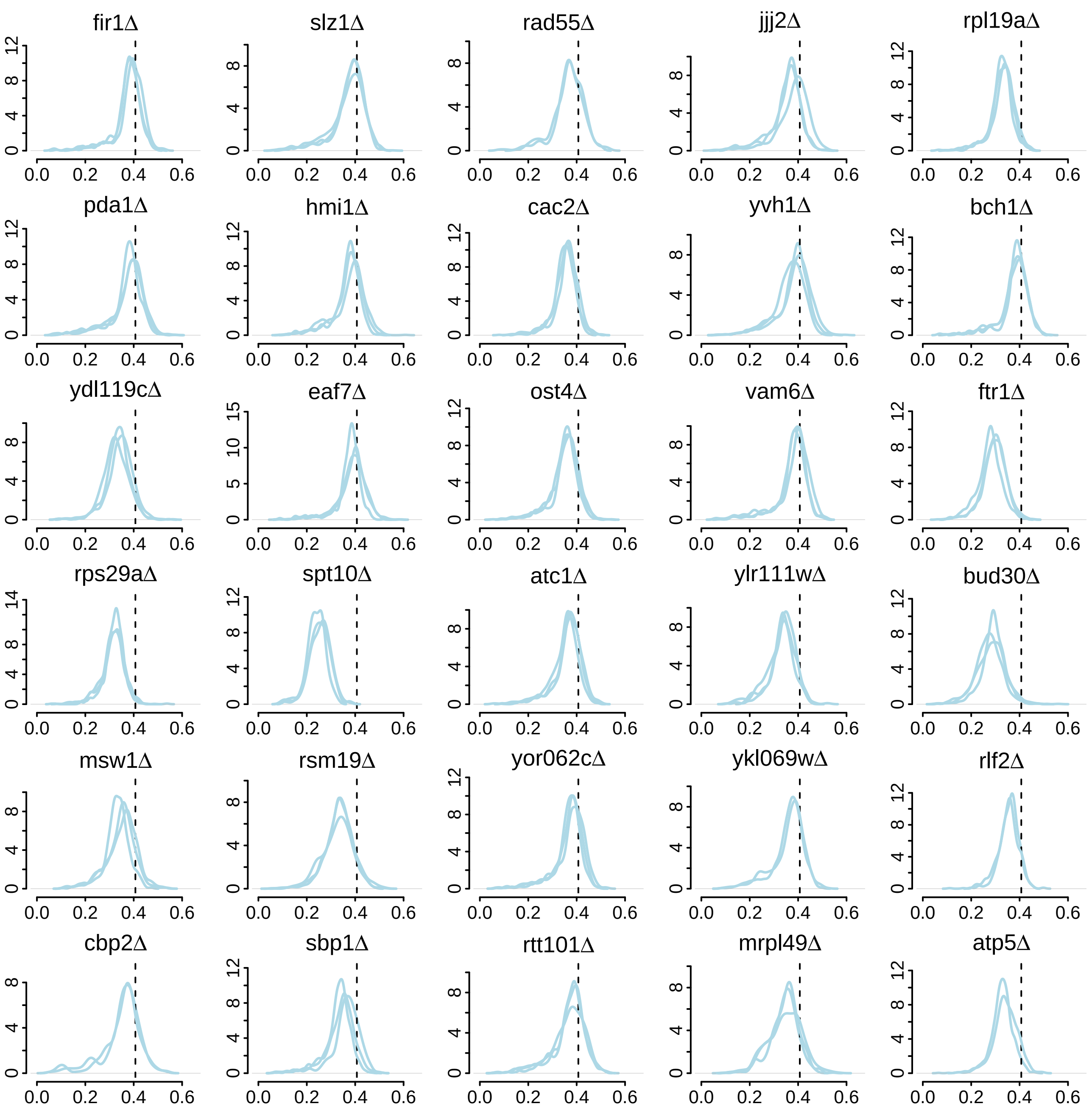
fir1Δ
slz1Δ
rad55Δ
jjj2Δ
rpl19aΔ
pda1Δ
hmi1Δ
cac2Δ
yvh1Δ
bch1Δ
ydl119cΔ
eaf7Δ
ost4Δ
vam6Δ
ftr1Δ
rps29aΔ
spt10Δ
atc1Δ
ylr111wΔ
bud30Δ
msw1Δ
rsm19Δ
yor062cΔ
ykl069wΔ
rlf2Δ
cbp2Δ
sbp1Δ
rtt101Δ
mrpl49Δ
atp5Δ
0.0
0.2
0.4
0.6
0
4
8
12
5
10
15
14

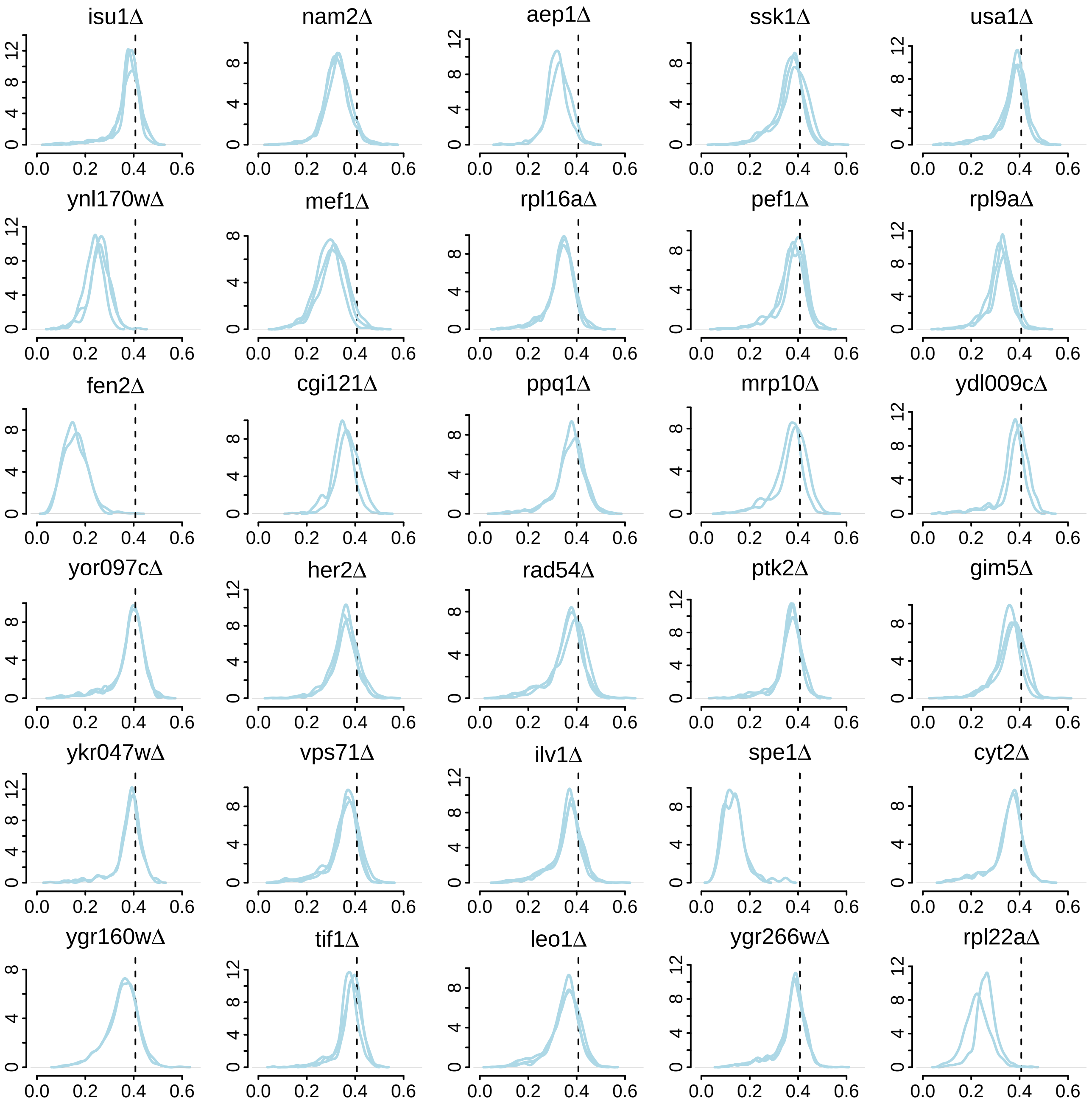
isu1Δ
nam2Δ
aep1Δ
ssk1Δ
usa1Δ
ynl170wΔ
mef1Δ
rpl16aΔ
pef1Δ
rpl9aΔ
fen2Δ
cgi121Δ
ppq1Δ
mrp10Δ
ydl009cΔ
yor097cΔ
her2Δ
rad54Δ
ptk2Δ
gim5Δ
ykr047wΔ
vps71Δ
ilv1Δ
spe1Δ
cyt2Δ
ygr160wΔ
tif1Δ
leo1Δ
ygr266wΔ
rpl22aΔ

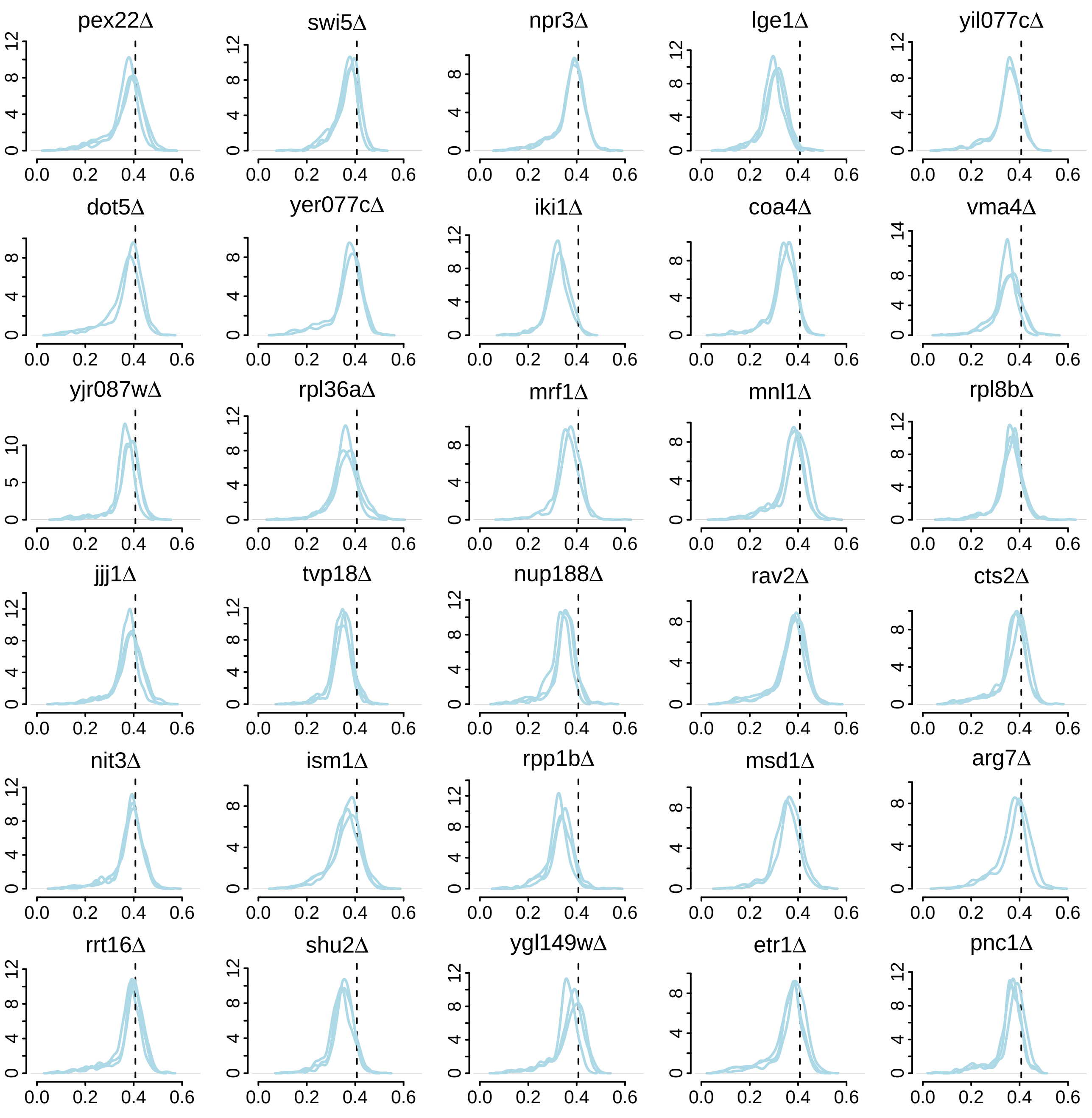
pex22Δ
swi5Δ
npr3Δ
lge1Δ
yil077cΔ
dot5Δ
yer077cΔ
iki1Δ
coa4Δ
vma4Δ
yjr087wΔ
rpl36aΔ
mrf1Δ
mnl1Δ
rpl8bΔ
jjj1Δ
tvp18Δ
nup188Δ
rav2Δ
cts2Δ
nit3Δ
ism1Δ
rpp1bΔ
msd1Δ
arg7Δ
rrt16Δ
shu2Δ
ygl149wΔ
etr1Δ
pnc1Δ

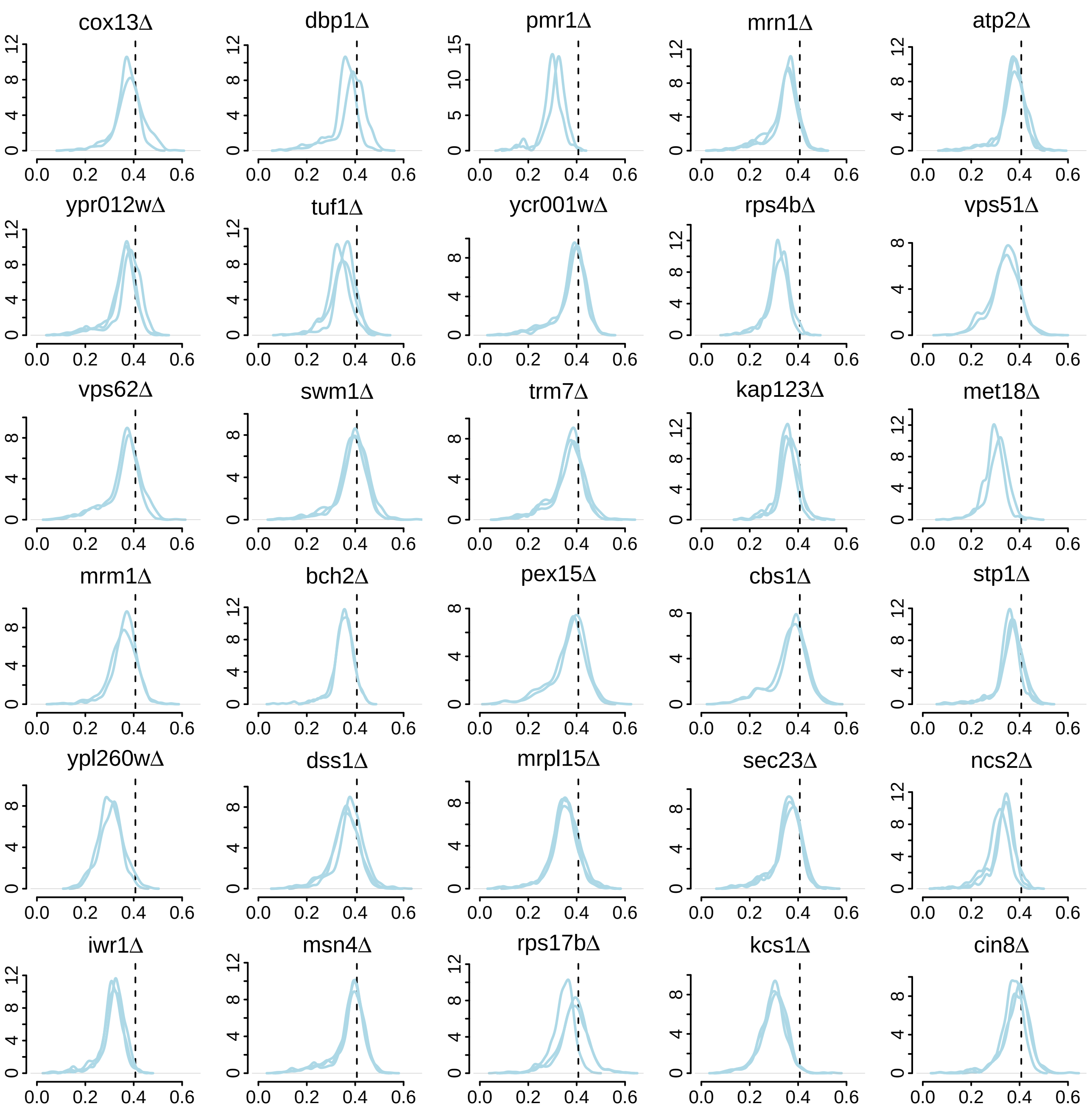
cox13Δ
dbp1Δ
pmr1Δ
mrn1Δ
atp2Δ
ypr012wΔ
tuf1Δ
ycr001wΔ
rps4bΔ
vps51Δ
vps62Δ
swm1Δ
trm7Δ
kap123Δ
met18Δ
mrm1Δ
bch2Δ
pex15Δ
cbs1Δ
stp1Δ
ypl260wΔ
dss1Δ
mrpl15Δ
sec23Δ
ncs2Δ
iwr1Δ
msn4Δ
rps17bΔ
kcs1Δ
cin8Δ

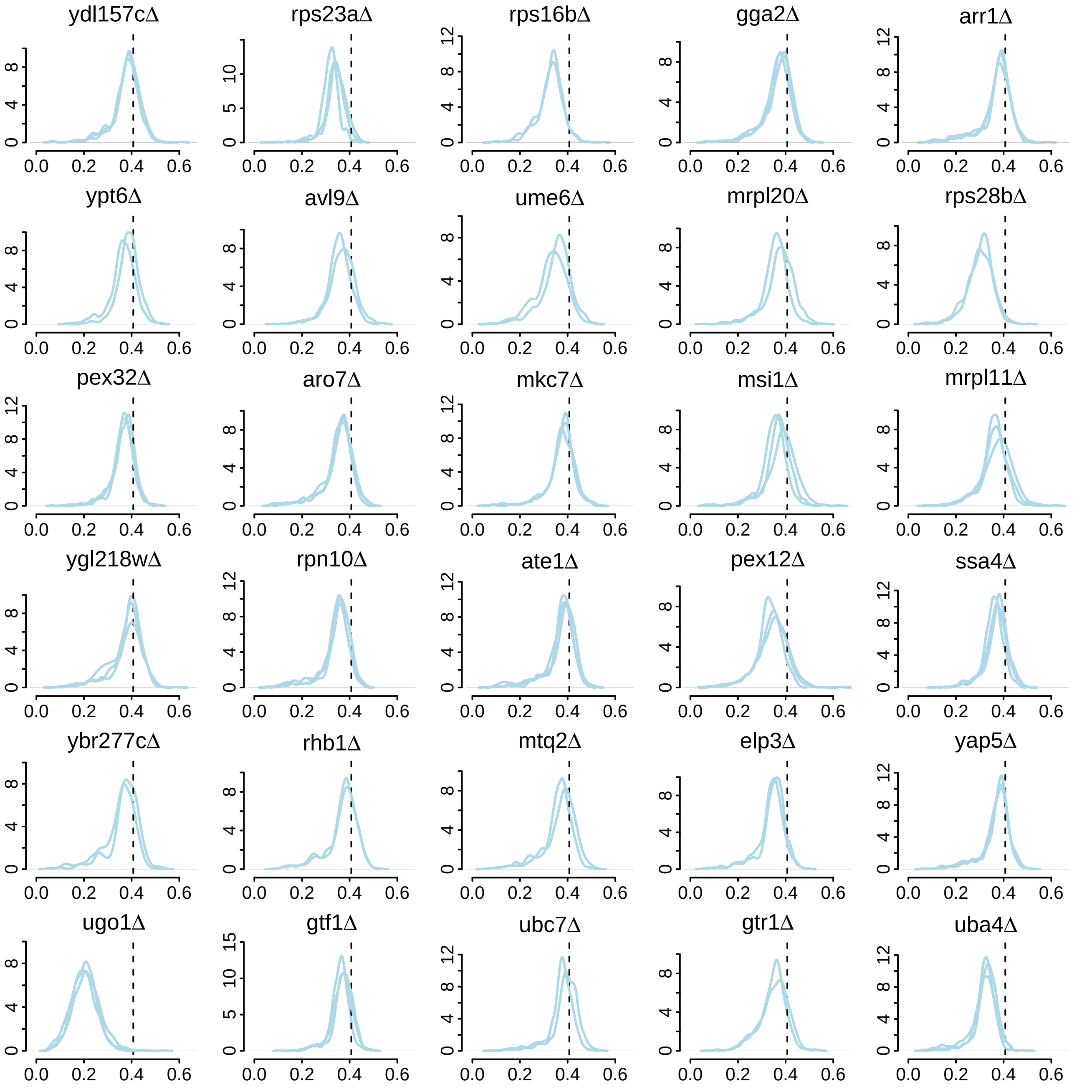
ydl157cΔ
rps23aΔ
rps16bΔ
gga2Δ
arr1Δ
ypt6Δ
avl9Δ
ume6Δ
mrpl20Δ
rps28bΔ
pex32Δ
aro7Δ
mkc7Δ
msi1Δ
mrpl11Δ
ygl218wΔ
rpn10Δ
ate1Δ
pex12Δ
ssa4Δ
ybr277cΔ
rhb1Δ
mtq2Δ
elp3Δ
yap5Δ
ugo1Δ
gtf1Δ
ubc7Δ
gtr1Δ
uba4Δ
0.0 0.2 0.4 0.6
0 4 8 12
0 5 10 15

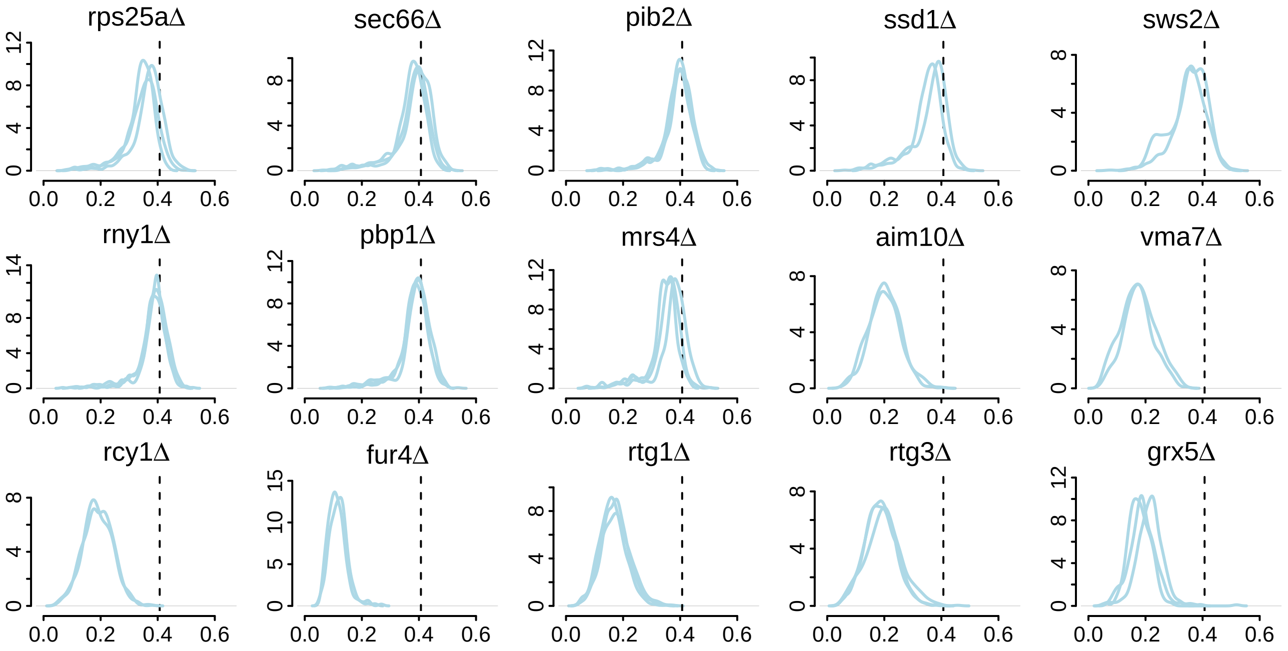
rps25aΔ
sec66Δ
pib2Δ
ssd1Δ
sws2Δ
rny1Δ
pbp1Δ
mrs4Δ
aim10Δ
vma7Δ
rcy1Δ
fur4Δ
rtg1Δ
rtg3Δ
grx5Δ
0.0 0.2 0.4 0.6

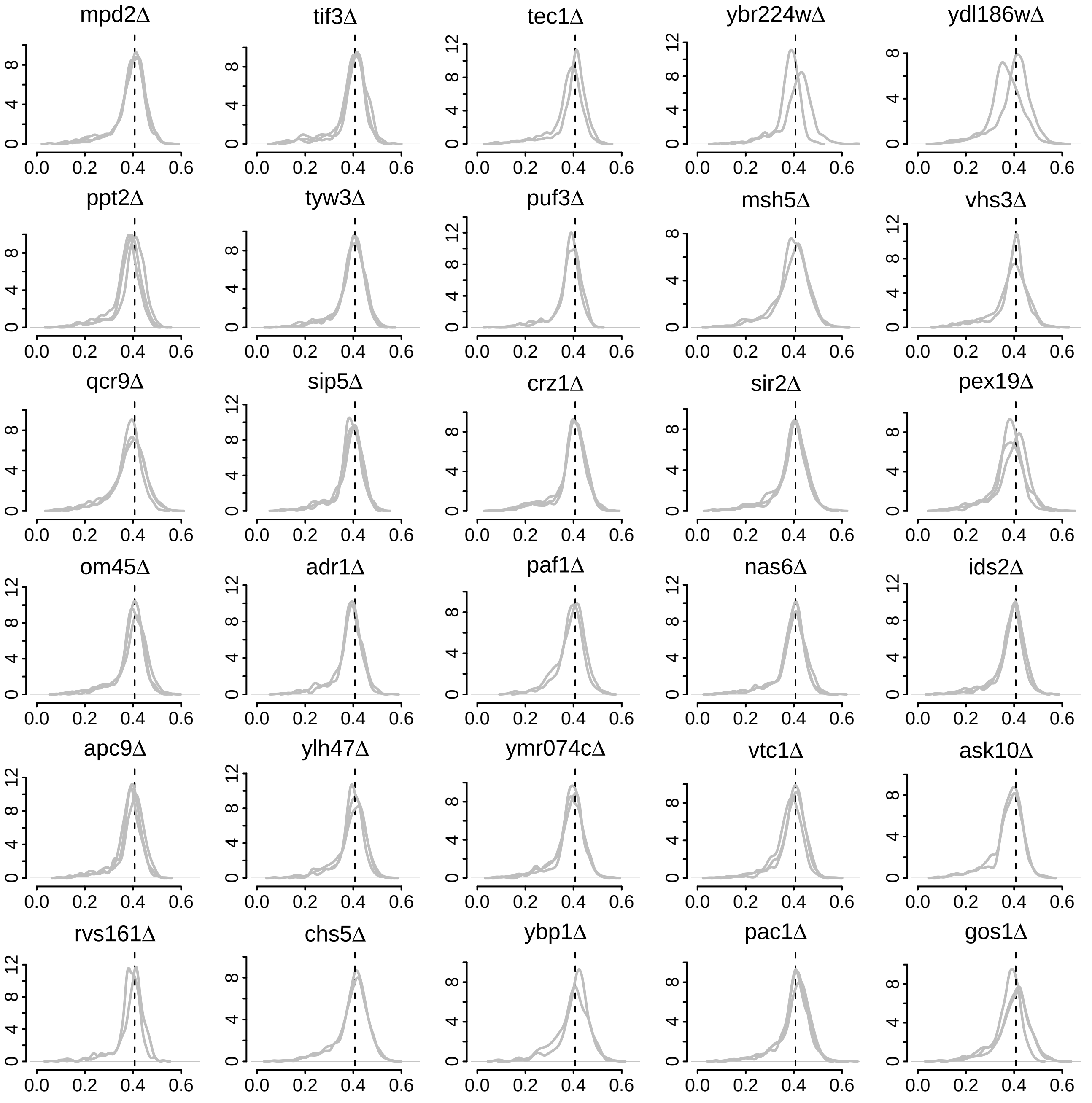
mpd2Δ
tif3Δ
tec1Δ
ybr224wΔ
ydl186wΔ
ppt2Δ
tyw3Δ
puf3Δ
msh5Δ
vhs3Δ
qcr9Δ
sip5Δ
crz1Δ
sir2Δ
pex19Δ
om45Δ
adr1Δ
paf1Δ
nas6Δ
ids2Δ
apc9Δ
ylh47Δ
ymr074cΔ
vtc1Δ
ask10Δ
rvs161Δ
chs5Δ
ybp1Δ
pac1Δ
gos1Δ
0.0 0.2 0.4 0.6
0 4 8 12

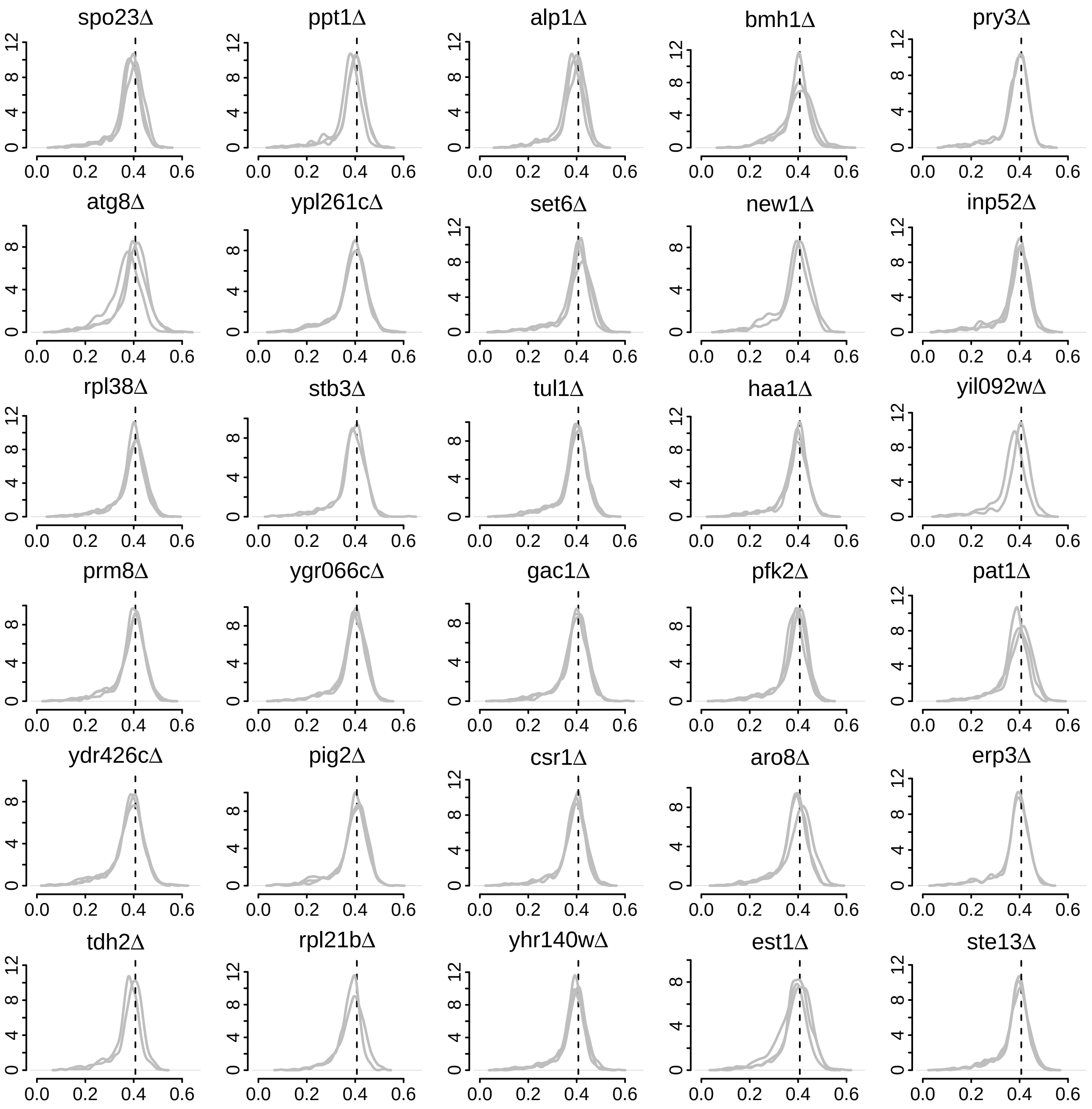
spo23Δ
ppt1Δ
alp1Δ
bmh1Δ
pry3Δ
atg8Δ
ypl261cΔ
set6Δ
new1Δ
inp52Δ
rpl38Δ
stb3Δ
tul1Δ
haa1Δ
yil092wΔ
prm8Δ
ygr066cΔ
gac1Δ
pfk2Δ
pat1Δ
ydr426cΔ
pig2Δ
csr1Δ
aro8Δ
erp3Δ
tdh2Δ
rpl21bΔ
yhr140wΔ
est1Δ
ste13Δ

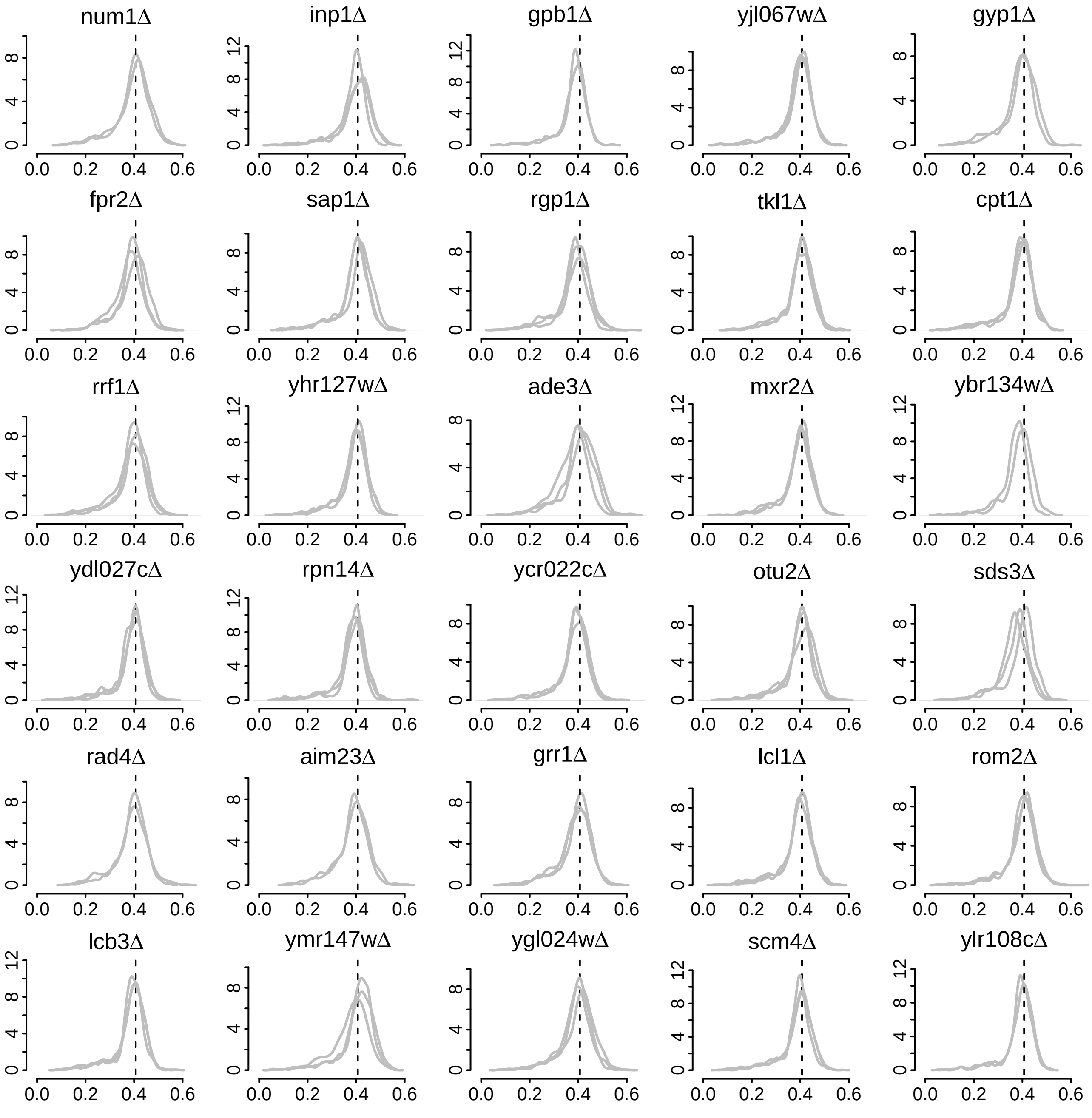

num1Δ
inp1Δ
gpb1Δ
yjl067wΔ
gyp1Δ
fpr2Δ
sap1Δ
rgp1Δ
tkl1Δ
cpt1Δ
rrf1Δ
yhr127wΔ
ade3Δ
mxr2Δ
ybr134wΔ
ydl027cΔ
rpn14Δ
ycr022cΔ
otu2Δ
sds3Δ
rad4Δ
aim23Δ
grr1Δ
lcl1Δ
rom2Δ
lcb3Δ
ymr147wΔ
ygl024wΔ
scm4Δ
ylr108cΔ
0.0 0.2 0.4 0.6
0 4 8 12

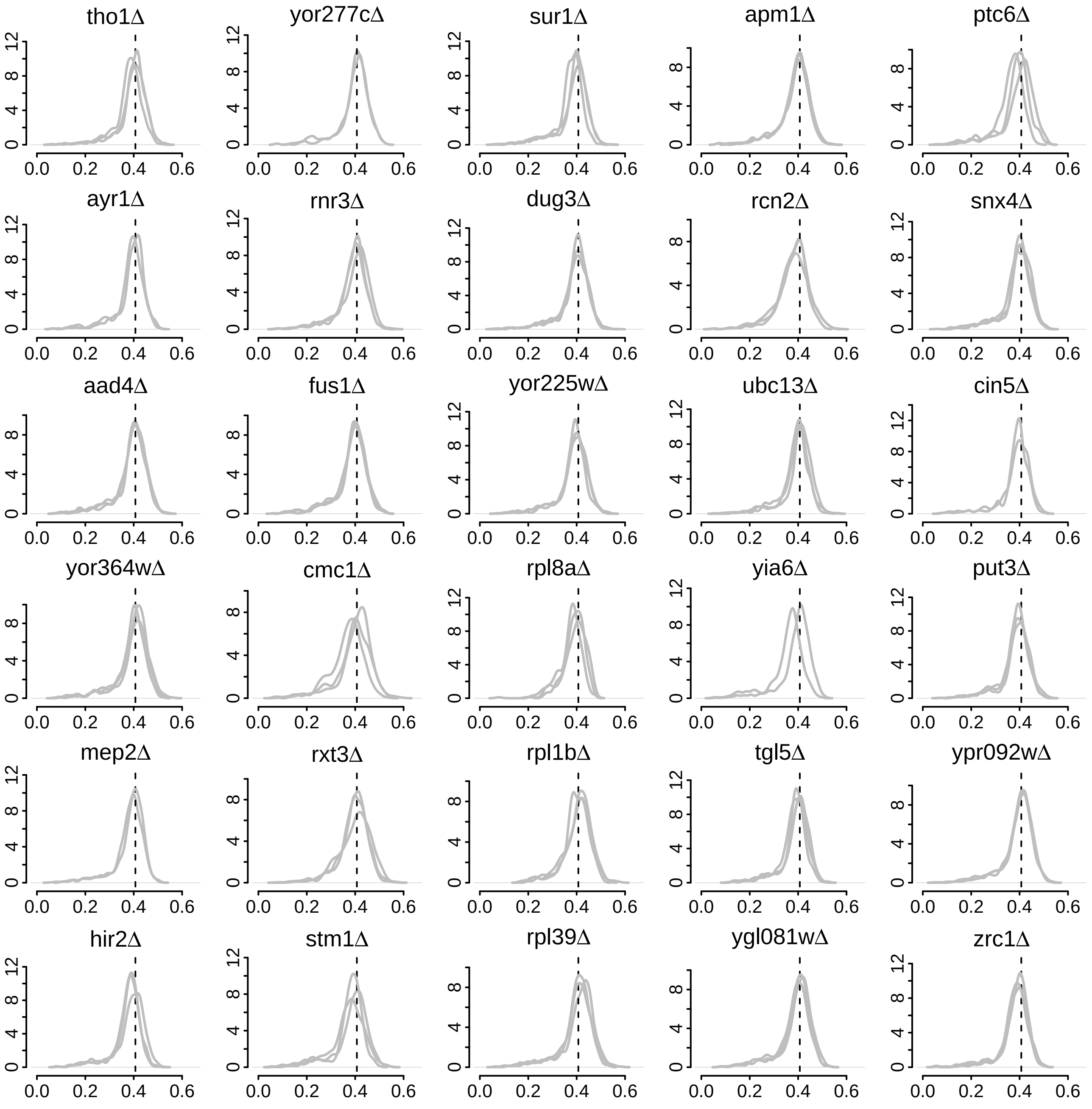

tho1Δ
yor277cΔ
sur1Δ
apm1Δ
ptc6Δ
ayr1Δ
rnr3Δ
dug3Δ
rcn2Δ
snx4Δ
aad4Δ
fus1Δ
yor225wΔ
ubc13Δ
cin5Δ
yor364wΔ
cmc1Δ
rpl8aΔ
yia6Δ
put3Δ
mep2Δ
rxt3Δ
rpl1bΔ
tgl5Δ
ypr092wΔ
hir2Δ
stm1Δ
rpl39Δ
ygl081wΔ
zrc1Δ
0.0 0.2 0.4 0.6
0 4 8 12

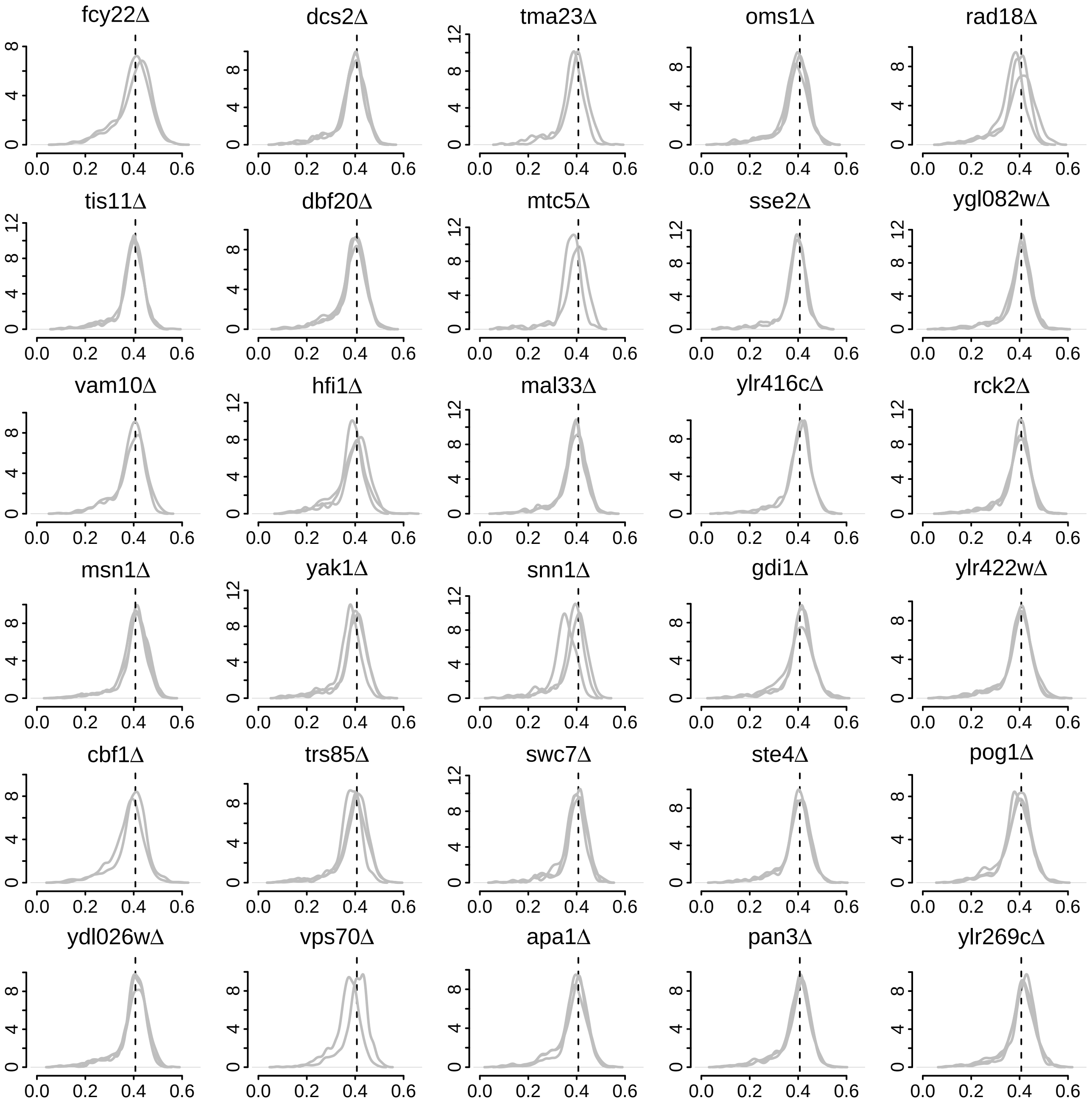
fcy22Δ
dcs2Δ
tma23Δ
oms1Δ
rad18Δ
tis11Δ
dbf20Δ
mtc5Δ
sse2Δ
ygl082wΔ
vam10Δ
hfi1Δ
mal33Δ
ylr416cΔ
rck2Δ
msn1Δ
yak1Δ
snn1Δ
gdi1Δ
ylr422wΔ
cbf1Δ
trs85Δ
swc7Δ
ste4Δ
pog1Δ
ydl026wΔ
vps70Δ
apa1Δ
pan3Δ
ylr269cΔ

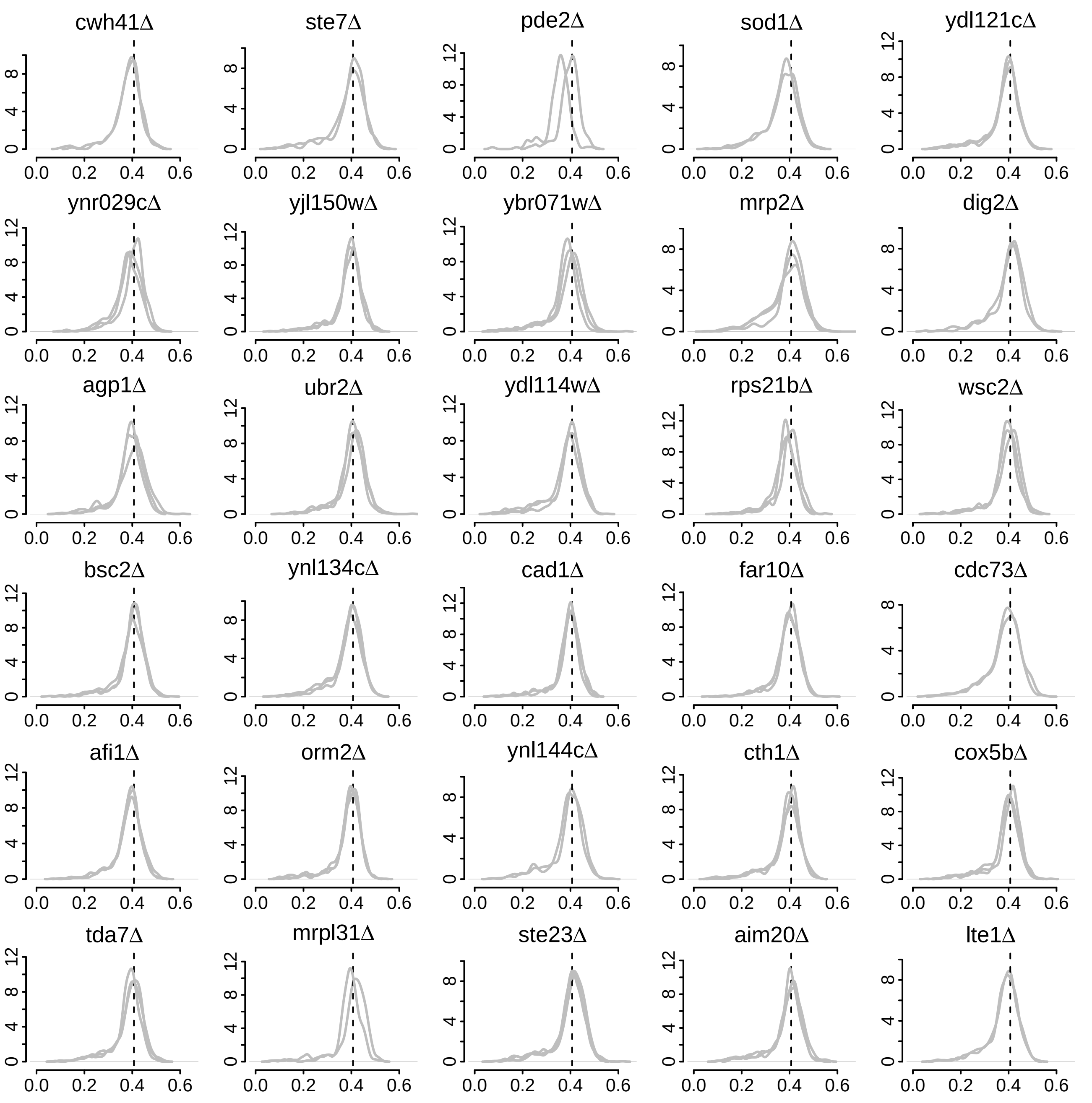

cwh41Δ
ste7Δ
pde2Δ
sod1Δ
ydl121cΔ
ynr029cΔ
yjl150wΔ
ybr071wΔ
mrp2Δ
dig2Δ
agp1Δ
ubr2Δ
ydl114wΔ
rps21bΔ
wsc2Δ
bsc2Δ
ynl134cΔ
cad1Δ
far10Δ
cdc73Δ
afi1Δ
orm2Δ
ynl144cΔ
cth1Δ
cox5bΔ
tda7Δ
mrpl31Δ
ste23Δ
aim20Δ
lte1Δ
0.0
0.2
0.4
0.6
0
4
8
12

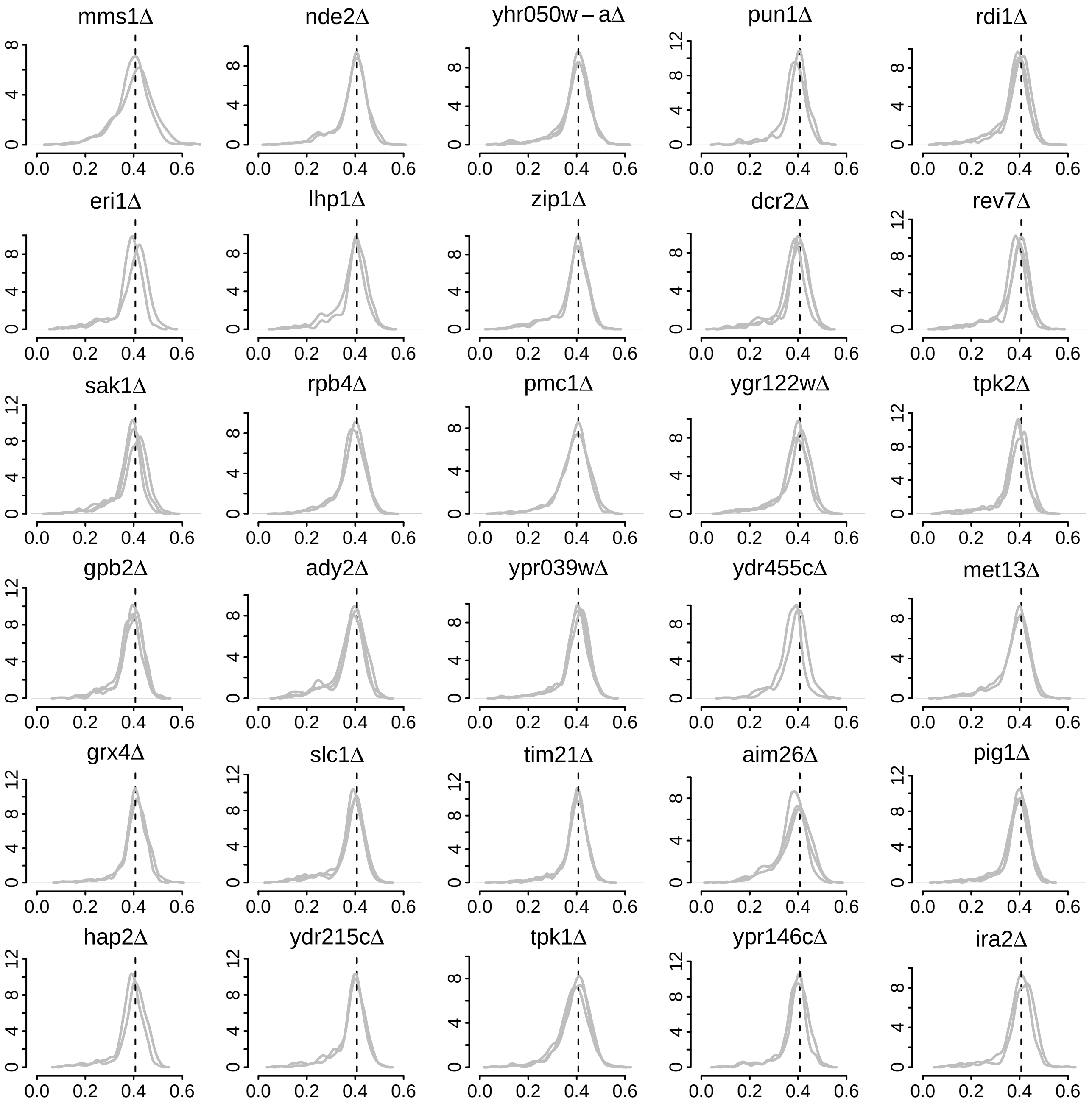
mms1Δ
nde2Δ
yhr050w – aΔ
pun1Δ
rdi1Δ
eri1Δ
lhp1Δ
zip1Δ
dcr2Δ
rev7Δ
sak1Δ
rpb4Δ
pmc1Δ
ygr122wΔ
tpk2Δ
gpb2Δ
ady2Δ
ypr039wΔ
ydr455cΔ
met13Δ
grx4Δ
slc1Δ
tim21Δ
aim26Δ
pig1Δ
hap2Δ
ydr215cΔ
tpk1Δ
ypr146cΔ
ira2Δ
0.0
0.2
0.4
0.6
0
4
8
12

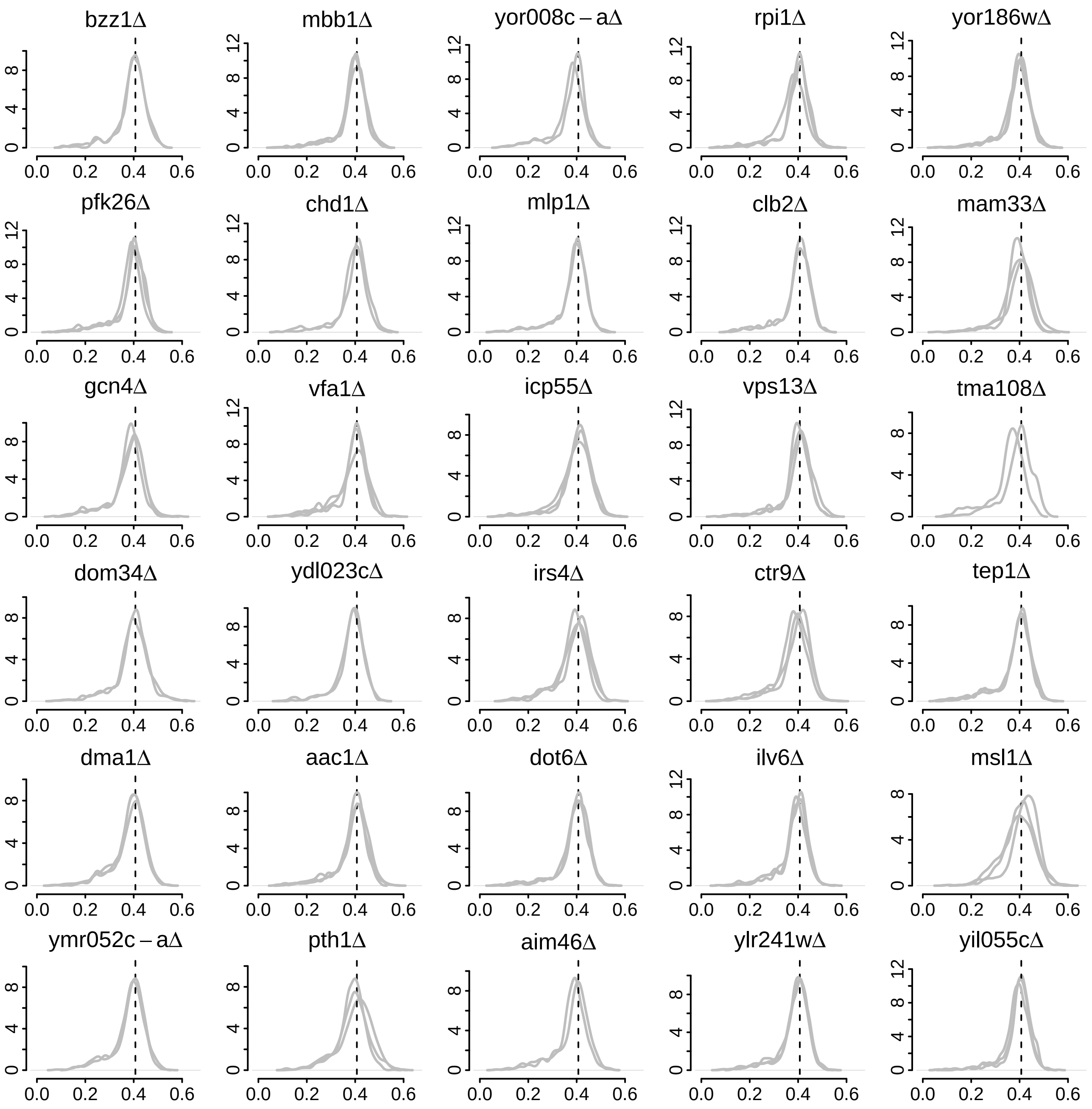
bzz1Δ
mbb1Δ
yor008c – aΔ
rpi1Δ
yor186wΔ
pfk26Δ
chd1Δ
mlp1Δ
clb2Δ
mam33Δ
gcn4Δ
vfa1Δ
icp55Δ
vps13Δ
tma108Δ
dom34Δ
ydl023cΔ
irs4Δ
ctr9Δ
tep1Δ
dma1Δ
aac1Δ
dot6Δ
ilv6Δ
msl1Δ
ymr052c – aΔ
pth1Δ
aim46Δ
ylr241wΔ
yil055cΔ
0.0
0.2
0.4
0.6
0
4
8
12

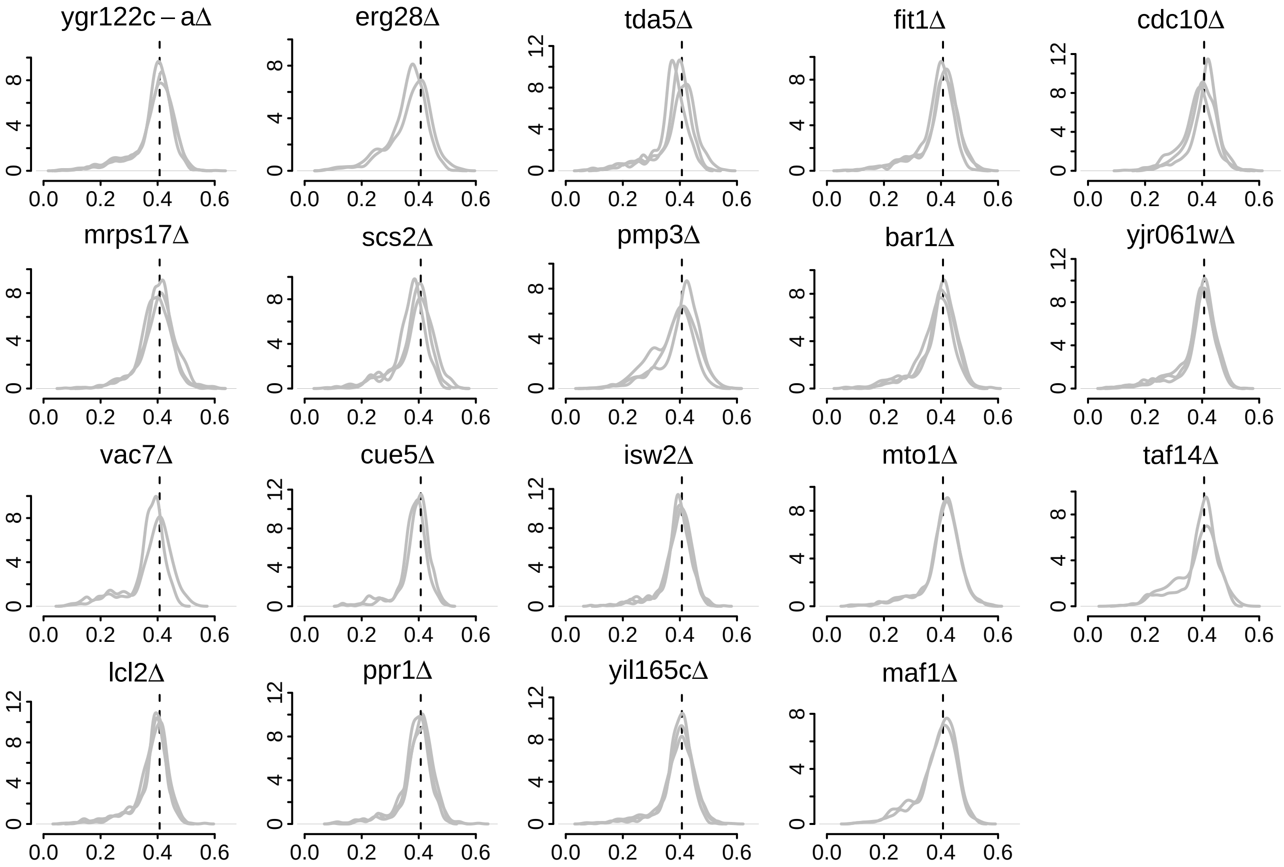
ygr122c – aΔ
erg28Δ
tda5Δ
fit1Δ
cdc10Δ
mrps17Δ
scs2Δ
pmp3Δ
bar1Δ
yjr061wΔ
vac7Δ
cue5Δ
isw2Δ
mto1Δ
taf14Δ
lcl2Δ
ppr1Δ
yil165cΔ
maf1Δ
0.0 0.2 0.4 0.6
0 4 8 12
