# Supplementary figures and images for: Single cell functional genomics reveals the importance of mitochondria in cell-to-cell phenotypic variation
Source: eLife. 2019 Jan 14;8:e38904. doi: 10.7554/eLife.38904 (PMC6366901; doi:10.7554/eLife.38904)

# FACSDiva Version 6.1.2

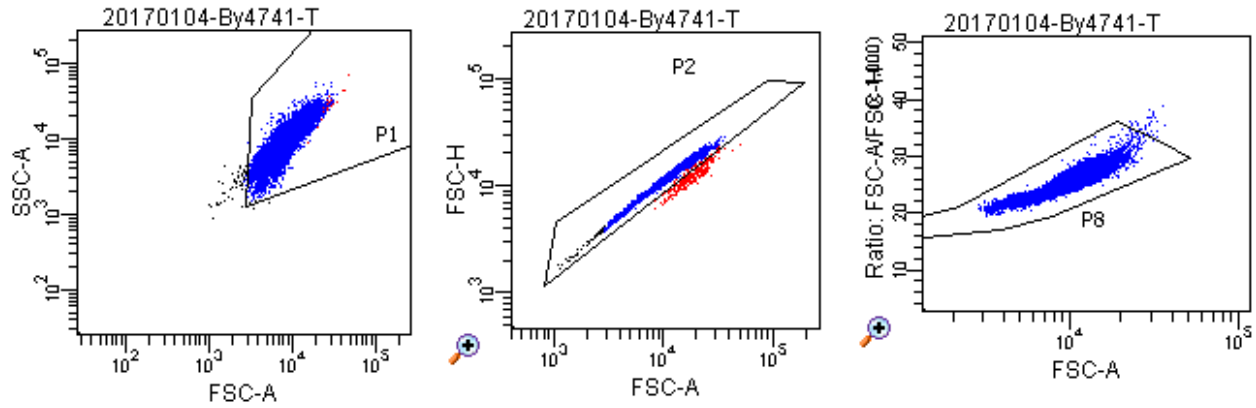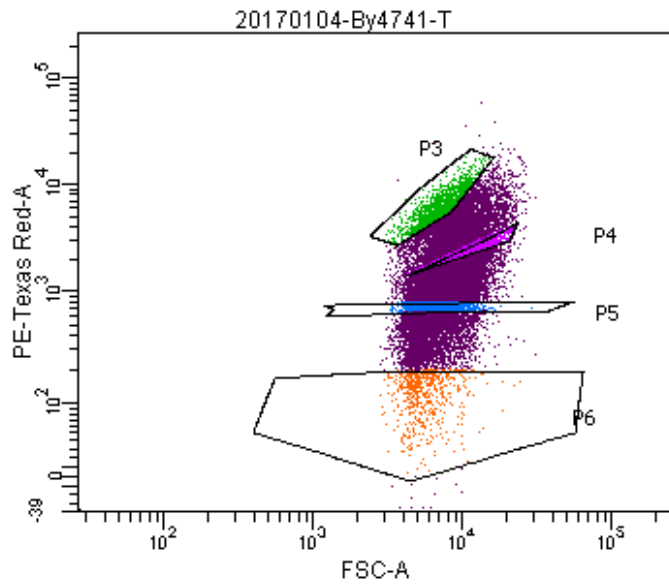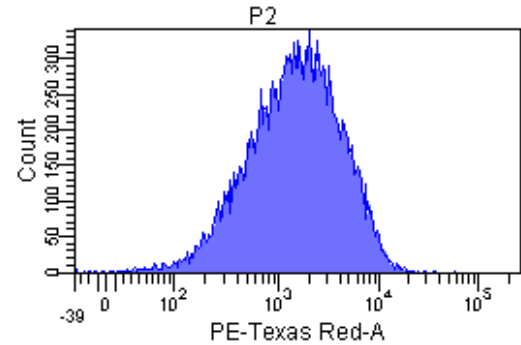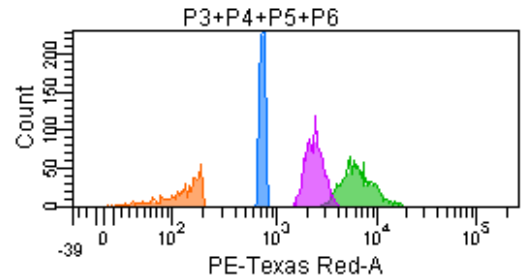

| Tube: By4741-T |         |         |        |
|----------------|---------|---------|--------|
| Population     | #Events | %Parent | %Total |
| All Events     | 20,357  | ###     | 100.0  |
| P1             | 20,300  | 99.7    | 99.7   |
| P2             | 20,000  | 98.5    | 98.2   |
| P8             | 19,972  | 99.9    | 98.1   |
| P3             | 1,133   | 5.7     | 5.6    |
| P4             | 1,168   | 5.8     | 5.7    |
| P5             | 1,129   | 5.7     | 5.5    |
| P6             | 633     | 3.2     | 3.1    |

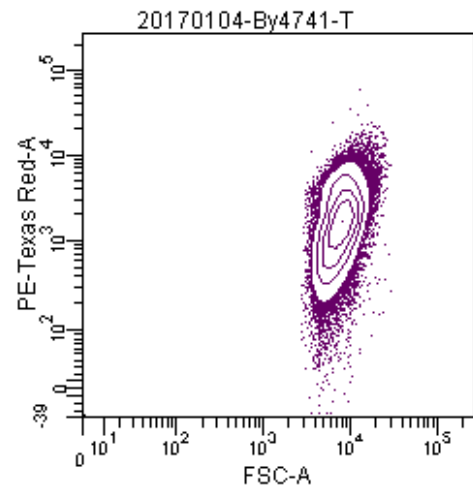

Supplement: Supplementary file 5. [file elife-38904-supp5.pdf]
