# Supplementary material for: Single cell functional genomics reveals the importance of mitochondria in cell-to-cell phenotypic variation
Source: eLife. 2019 Jan 14;8:e38904. doi: 10.7554/eLife.38904 (PMC6366901; doi:10.7554/eLife.38904)
Supplement: Supplementary file 6. [file elife-38904-supp6.docx]

**Supplementary file 6.** Key Resources Table.

| **Reagent type (species) or resource** | **Designation** | **Source or reference** | **Identifiers** | **Additional information** |
| --- | --- | --- | --- | --- |
| strain, strain background (*S. cerevisiae*) | BY4741 | Brachman *et al.*, 1998, Yeast |  | MATa his3Δ1 leu2Δ0 met15Δ0 ura3Δ0 |
| strain, strain background (*S. cerevisiae*) | BY4742 | Brachman *et al.*, 1998, Yeast |  | MATα his3Δ1 leu2Δ0 lys2Δ0 ura3Δ0 |
| strain, strain background (*S. cerevisiae*) | BY4743 | Brachman *et al.*, 1998, Yeast |  | MATa/α his3Δ1/his3Δ1 leu2Δ0/leu2Δ0 LYS2/lys2Δ0 met15Δ0/MET15 ura3Δ0/ura3Δ0 |
| strain, strain background (*S. cerevisiae*) | FY4 | Winston *et al.*, 1995, Yeast; Brachman *et al.*, 1998, Yeast |  | MATa |
| strain, strain background (*S. cerevisiae*) | yal002wΔ | YSC1053 Yeast MATa deletion collection |  | http://www-sequence.stanford.edu/group/yeast_deletion_project/deletions3.html |
| strain, strain background (*S. cerevisiae*) | yal009wΔ | YSC1053 Yeast MATa deletion collection |  | http://www-sequence.stanford.edu/group/yeast_deletion_project/deletions3.html |
| strain, strain background (*S. cerevisiae*) | yal010cΔ | YSC1053 Yeast MATa deletion collection |  | http://www-sequence.stanford.edu/group/yeast_deletion_project/deletions3.html |
| strain, strain background (*S. cerevisiae*) | yal011wΔ | YSC1053 Yeast MATa deletion collection |  | http://www-sequence.stanford.edu/group/yeast_deletion_project/deletions3.html |
| strain, strain background (*S. cerevisiae*) | yal012wΔ | YSC1053 Yeast MATa deletion collection |  | http://www-sequence.stanford.edu/group/yeast_deletion_project/deletions3.html |
| strain, strain background (*S. cerevisiae*) | yal013wΔ | YSC1053 Yeast MATa deletion collection |  | http://www-sequence.stanford.edu/group/yeast_deletion_project/deletions3.html |
| strain, strain background (*S. cerevisiae*) | yal014cΔ | YSC1053 Yeast MATa deletion collection |  | http://www-sequence.stanford.edu/group/yeast_deletion_project/deletions3.html |
| strain, strain background (*S. cerevisiae*) | yal019wΔ | YSC1053 Yeast MATa deletion collection |  | http://www-sequence.stanford.edu/group/yeast_deletion_project/deletions3.html |
| strain, strain background (*S. cerevisiae*) | yal020cΔ | YSC1053 Yeast MATa deletion collection |  | http://www-sequence.stanford.edu/group/yeast_deletion_project/deletions3.html |
| strain, strain background (*S. cerevisiae*) | yal021cΔ | YSC1053 Yeast MATa deletion collection |  | http://www-sequence.stanford.edu/group/yeast_deletion_project/deletions3.html |
| strain, strain background (*S. cerevisiae*) | yal022cΔ | YSC1053 Yeast MATa deletion collection |  | http://www-sequence.stanford.edu/group/yeast_deletion_project/deletions3.html |
| strain, strain background (*S. cerevisiae*) | yal024cΔ | YSC1053 Yeast MATa deletion collection |  | http://www-sequence.stanford.edu/group/yeast_deletion_project/deletions3.html |
| strain, strain background (*S. cerevisiae*) | yal036cΔ | YSC1053 Yeast MATa deletion collection |  | http://www-sequence.stanford.edu/group/yeast_deletion_project/deletions3.html |
| strain, strain background (*S. cerevisiae*) | yal039cΔ | YSC1053 Yeast MATa deletion collection |  | http://www-sequence.stanford.edu/group/yeast_deletion_project/deletions3.html |
| strain, strain background (*S. cerevisiae*) | yal042wΔ | YSC1053 Yeast MATa deletion collection |  | http://www-sequence.stanford.edu/group/yeast_deletion_project/deletions3.html |
| strain, strain background (*S. cerevisiae*) | yal044cΔ | YSC1053 Yeast MATa deletion collection |  | http://www-sequence.stanford.edu/group/yeast_deletion_project/deletions3.html |
| strain, strain background (*S. cerevisiae*) | yal047cΔ | YSC1053 Yeast MATa deletion collection |  | http://www-sequence.stanford.edu/group/yeast_deletion_project/deletions3.html |
| strain, strain background (*S. cerevisiae*) | yal048cΔ | YSC1053 Yeast MATa deletion collection |  | http://www-sequence.stanford.edu/group/yeast_deletion_project/deletions3.html |
| strain, strain background (*S. cerevisiae*) | yal055wΔ | YSC1053 Yeast MATa deletion collection |  | http://www-sequence.stanford.edu/group/yeast_deletion_project/deletions3.html |
| strain, strain background (*S. cerevisiae*) | yal056wΔ | YSC1053 Yeast MATa deletion collection |  | http://www-sequence.stanford.edu/group/yeast_deletion_project/deletions3.html |
| strain, strain background (*S. cerevisiae*) | yal060wΔ | YSC1053 Yeast MATa deletion collection |  | http://www-sequence.stanford.edu/group/yeast_deletion_project/deletions3.html |
| strain, strain background (*S. cerevisiae*) | yal064c-aΔ | YSC1053 Yeast MATa deletion collection |  | http://www-sequence.stanford.edu/group/yeast_deletion_project/deletions3.html |
| strain, strain background (*S. cerevisiae*) | yal067cΔ | YSC1053 Yeast MATa deletion collection |  | http://www-sequence.stanford.edu/group/yeast_deletion_project/deletions3.html |
| strain, strain background (*S. cerevisiae*) | yar003wΔ | YSC1053 Yeast MATa deletion collection |  | http://www-sequence.stanford.edu/group/yeast_deletion_project/deletions3.html |
| strain, strain background (*S. cerevisiae*) | yar014cΔ | YSC1053 Yeast MATa deletion collection |  | http://www-sequence.stanford.edu/group/yeast_deletion_project/deletions3.html |
| strain, strain background (*S. cerevisiae*) | yar029wΔ | YSC1053 Yeast MATa deletion collection |  | http://www-sequence.stanford.edu/group/yeast_deletion_project/deletions3.html |
| strain, strain background (*S. cerevisiae*) | yar044wΔ | YSC1053 Yeast MATa deletion collection |  | http://www-sequence.stanford.edu/group/yeast_deletion_project/deletions3.html |
| strain, strain background (*S. cerevisiae*) | ybl002wΔ | YSC1053 Yeast MATa deletion collection |  | http://www-sequence.stanford.edu/group/yeast_deletion_project/deletions3.html |
| strain, strain background (*S. cerevisiae*) | ybl006cΔ | YSC1053 Yeast MATa deletion collection |  | http://www-sequence.stanford.edu/group/yeast_deletion_project/deletions3.html |
| strain, strain background (*S. cerevisiae*) | ybl007cΔ | YSC1053 Yeast MATa deletion collection |  | http://www-sequence.stanford.edu/group/yeast_deletion_project/deletions3.html |
| strain, strain background (*S. cerevisiae*) | ybl016wΔ | YSC1053 Yeast MATa deletion collection |  | http://www-sequence.stanford.edu/group/yeast_deletion_project/deletions3.html |
| strain, strain background (*S. cerevisiae*) | ybl017cΔ | YSC1053 Yeast MATa deletion collection |  | http://www-sequence.stanford.edu/group/yeast_deletion_project/deletions3.html |
| strain, strain background (*S. cerevisiae*) | ybl021cΔ | YSC1053 Yeast MATa deletion collection |  | http://www-sequence.stanford.edu/group/yeast_deletion_project/deletions3.html |
| strain, strain background (*S. cerevisiae*) | ybl022cΔ | YSC1053 Yeast MATa deletion collection |  | http://www-sequence.stanford.edu/group/yeast_deletion_project/deletions3.html |
| strain, strain background (*S. cerevisiae*) | ybl024wΔ | YSC1053 Yeast MATa deletion collection |  | http://www-sequence.stanford.edu/group/yeast_deletion_project/deletions3.html |
| strain, strain background (*S. cerevisiae*) | ybl025wΔ | YSC1053 Yeast MATa deletion collection |  | http://www-sequence.stanford.edu/group/yeast_deletion_project/deletions3.html |
| strain, strain background (*S. cerevisiae*) | ybl027wΔ | YSC1053 Yeast MATa deletion collection |  | http://www-sequence.stanford.edu/group/yeast_deletion_project/deletions3.html |
| strain, strain background (*S. cerevisiae*) | ybl031wΔ | YSC1053 Yeast MATa deletion collection |  | http://www-sequence.stanford.edu/group/yeast_deletion_project/deletions3.html |
| strain, strain background (*S. cerevisiae*) | ybl038wΔ | YSC1053 Yeast MATa deletion collection |  | http://www-sequence.stanford.edu/group/yeast_deletion_project/deletions3.html |
| strain, strain background (*S. cerevisiae*) | ybl039cΔ | YSC1053 Yeast MATa deletion collection |  | http://www-sequence.stanford.edu/group/yeast_deletion_project/deletions3.html |
| strain, strain background (*S. cerevisiae*) | ybl045cΔ | YSC1053 Yeast MATa deletion collection |  | http://www-sequence.stanford.edu/group/yeast_deletion_project/deletions3.html |
| strain, strain background (*S. cerevisiae*) | ybl047cΔ | YSC1053 Yeast MATa deletion collection |  | http://www-sequence.stanford.edu/group/yeast_deletion_project/deletions3.html |
| strain, strain background (*S. cerevisiae*) | ybl051cΔ | YSC1053 Yeast MATa deletion collection |  | http://www-sequence.stanford.edu/group/yeast_deletion_project/deletions3.html |
| strain, strain background (*S. cerevisiae*) | ybl054wΔ | YSC1053 Yeast MATa deletion collection |  | http://www-sequence.stanford.edu/group/yeast_deletion_project/deletions3.html |
| strain, strain background (*S. cerevisiae*) | ybl058wΔ | YSC1053 Yeast MATa deletion collection |  | http://www-sequence.stanford.edu/group/yeast_deletion_project/deletions3.html |
| strain, strain background (*S. cerevisiae*) | ybl064cΔ | YSC1053 Yeast MATa deletion collection |  | http://www-sequence.stanford.edu/group/yeast_deletion_project/deletions3.html |
| strain, strain background (*S. cerevisiae*) | ybl071c-bΔ | YSC1053 Yeast MATa deletion collection |  | http://www-sequence.stanford.edu/group/yeast_deletion_project/deletions3.html |
| strain, strain background (*S. cerevisiae*) | ybl072cΔ | YSC1053 Yeast MATa deletion collection |  | http://www-sequence.stanford.edu/group/yeast_deletion_project/deletions3.html |
| strain, strain background (*S. cerevisiae*) | ybl078cΔ | YSC1053 Yeast MATa deletion collection |  | http://www-sequence.stanford.edu/group/yeast_deletion_project/deletions3.html |
| strain, strain background (*S. cerevisiae*) | ybl079wΔ | YSC1053 Yeast MATa deletion collection |  | http://www-sequence.stanford.edu/group/yeast_deletion_project/deletions3.html |
| strain, strain background (*S. cerevisiae*) | ybl080cΔ | YSC1053 Yeast MATa deletion collection |  | http://www-sequence.stanford.edu/group/yeast_deletion_project/deletions3.html |
| strain, strain background (*S. cerevisiae*) | ybl087cΔ | YSC1053 Yeast MATa deletion collection |  | http://www-sequence.stanford.edu/group/yeast_deletion_project/deletions3.html |
| strain, strain background (*S. cerevisiae*) | ybl090wΔ | YSC1053 Yeast MATa deletion collection |  | http://www-sequence.stanford.edu/group/yeast_deletion_project/deletions3.html |
| strain, strain background (*S. cerevisiae*) | ybl094cΔ | YSC1053 Yeast MATa deletion collection |  | http://www-sequence.stanford.edu/group/yeast_deletion_project/deletions3.html |
| strain, strain background (*S. cerevisiae*) | ybl099wΔ | YSC1053 Yeast MATa deletion collection |  | http://www-sequence.stanford.edu/group/yeast_deletion_project/deletions3.html |
| strain, strain background (*S. cerevisiae*) | ybl100cΔ | YSC1053 Yeast MATa deletion collection |  | http://www-sequence.stanford.edu/group/yeast_deletion_project/deletions3.html |
| strain, strain background (*S. cerevisiae*) | ybl103cΔ | YSC1053 Yeast MATa deletion collection |  | http://www-sequence.stanford.edu/group/yeast_deletion_project/deletions3.html |
| strain, strain background (*S. cerevisiae*) | ybl104cΔ | YSC1053 Yeast MATa deletion collection |  | http://www-sequence.stanford.edu/group/yeast_deletion_project/deletions3.html |
| strain, strain background (*S. cerevisiae*) | ybr001cΔ | YSC1053 Yeast MATa deletion collection |  | http://www-sequence.stanford.edu/group/yeast_deletion_project/deletions3.html |
| strain, strain background (*S. cerevisiae*) | ybr003wΔ | YSC1053 Yeast MATa deletion collection |  | http://www-sequence.stanford.edu/group/yeast_deletion_project/deletions3.html |
| strain, strain background (*S. cerevisiae*) | ybr006wΔ | YSC1053 Yeast MATa deletion collection |  | http://www-sequence.stanford.edu/group/yeast_deletion_project/deletions3.html |
| strain, strain background (*S. cerevisiae*) | ybr009cΔ | YSC1053 Yeast MATa deletion collection |  | http://www-sequence.stanford.edu/group/yeast_deletion_project/deletions3.html |
| strain, strain background (*S. cerevisiae*) | ybr016wΔ | YSC1053 Yeast MATa deletion collection |  | http://www-sequence.stanford.edu/group/yeast_deletion_project/deletions3.html |
| strain, strain background (*S. cerevisiae*) | ybr021wΔ | YSC1053 Yeast MATa deletion collection |  | http://www-sequence.stanford.edu/group/yeast_deletion_project/deletions3.html |
| strain, strain background (*S. cerevisiae*) | ybr025cΔ | YSC1053 Yeast MATa deletion collection |  | http://www-sequence.stanford.edu/group/yeast_deletion_project/deletions3.html |
| strain, strain background (*S. cerevisiae*) | ybr026cΔ | YSC1053 Yeast MATa deletion collection |  | http://www-sequence.stanford.edu/group/yeast_deletion_project/deletions3.html |
| strain, strain background (*S. cerevisiae*) | ybr031wΔ | YSC1053 Yeast MATa deletion collection |  | http://www-sequence.stanford.edu/group/yeast_deletion_project/deletions3.html |
| strain, strain background (*S. cerevisiae*) | ybr035cΔ | YSC1053 Yeast MATa deletion collection |  | http://www-sequence.stanford.edu/group/yeast_deletion_project/deletions3.html |
| strain, strain background (*S. cerevisiae*) | ybr036cΔ | YSC1053 Yeast MATa deletion collection |  | http://www-sequence.stanford.edu/group/yeast_deletion_project/deletions3.html |
| strain, strain background (*S. cerevisiae*) | ybr037cΔ | YSC1053 Yeast MATa deletion collection |  | http://www-sequence.stanford.edu/group/yeast_deletion_project/deletions3.html |
| strain, strain background (*S. cerevisiae*) | ybr048wΔ | YSC1053 Yeast MATa deletion collection |  | http://www-sequence.stanford.edu/group/yeast_deletion_project/deletions3.html |
| strain, strain background (*S. cerevisiae*) | ybr061cΔ | YSC1053 Yeast MATa deletion collection |  | http://www-sequence.stanford.edu/group/yeast_deletion_project/deletions3.html |
| strain, strain background (*S. cerevisiae*) | ybr063cΔ | YSC1053 Yeast MATa deletion collection |  | http://www-sequence.stanford.edu/group/yeast_deletion_project/deletions3.html |
| strain, strain background (*S. cerevisiae*) | ybr071wΔ | YSC1053 Yeast MATa deletion collection |  | http://www-sequence.stanford.edu/group/yeast_deletion_project/deletions3.html |
| strain, strain background (*S. cerevisiae*) | ybr076wΔ | YSC1053 Yeast MATa deletion collection |  | http://www-sequence.stanford.edu/group/yeast_deletion_project/deletions3.html |
| strain, strain background (*S. cerevisiae*) | ybr082cΔ | YSC1053 Yeast MATa deletion collection |  | http://www-sequence.stanford.edu/group/yeast_deletion_project/deletions3.html |
| strain, strain background (*S. cerevisiae*) | ybr083wΔ | YSC1053 Yeast MATa deletion collection |  | http://www-sequence.stanford.edu/group/yeast_deletion_project/deletions3.html |
| strain, strain background (*S. cerevisiae*) | ybr084c-aΔ | YSC1053 Yeast MATa deletion collection |  | http://www-sequence.stanford.edu/group/yeast_deletion_project/deletions3.html |
| strain, strain background (*S. cerevisiae*) | ybr094wΔ | YSC1053 Yeast MATa deletion collection |  | http://www-sequence.stanford.edu/group/yeast_deletion_project/deletions3.html |
| strain, strain background (*S. cerevisiae*) | ybr100wΔ | YSC1053 Yeast MATa deletion collection |  | http://www-sequence.stanford.edu/group/yeast_deletion_project/deletions3.html |
| strain, strain background (*S. cerevisiae*) | ybr101cΔ | YSC1053 Yeast MATa deletion collection |  | http://www-sequence.stanford.edu/group/yeast_deletion_project/deletions3.html |
| strain, strain background (*S. cerevisiae*) | ybr103wΔ | YSC1053 Yeast MATa deletion collection |  | http://www-sequence.stanford.edu/group/yeast_deletion_project/deletions3.html |
| strain, strain background (*S. cerevisiae*) | ybr106wΔ | YSC1053 Yeast MATa deletion collection |  | http://www-sequence.stanford.edu/group/yeast_deletion_project/deletions3.html |
| strain, strain background (*S. cerevisiae*) | ybr111w-aΔ | YSC1053 Yeast MATa deletion collection |  | http://www-sequence.stanford.edu/group/yeast_deletion_project/deletions3.html |
| strain, strain background (*S. cerevisiae*) | ybr120cΔ | YSC1053 Yeast MATa deletion collection |  | http://www-sequence.stanford.edu/group/yeast_deletion_project/deletions3.html |
| strain, strain background (*S. cerevisiae*) | ybr122cΔ | YSC1053 Yeast MATa deletion collection |  | http://www-sequence.stanford.edu/group/yeast_deletion_project/deletions3.html |
| strain, strain background (*S. cerevisiae*) | ybr126cΔ | YSC1053 Yeast MATa deletion collection |  | http://www-sequence.stanford.edu/group/yeast_deletion_project/deletions3.html |
| strain, strain background (*S. cerevisiae*) | ybr127cΔ | YSC1053 Yeast MATa deletion collection |  | http://www-sequence.stanford.edu/group/yeast_deletion_project/deletions3.html |
| strain, strain background (*S. cerevisiae*) | ybr131wΔ | YSC1053 Yeast MATa deletion collection |  | http://www-sequence.stanford.edu/group/yeast_deletion_project/deletions3.html |
| strain, strain background (*S. cerevisiae*) | ybr134wΔ | YSC1053 Yeast MATa deletion collection |  | http://www-sequence.stanford.edu/group/yeast_deletion_project/deletions3.html |
| strain, strain background (*S. cerevisiae*) | ybr146wΔ | YSC1053 Yeast MATa deletion collection |  | http://www-sequence.stanford.edu/group/yeast_deletion_project/deletions3.html |
| strain, strain background (*S. cerevisiae*) | ybr156cΔ | YSC1053 Yeast MATa deletion collection |  | http://www-sequence.stanford.edu/group/yeast_deletion_project/deletions3.html |
| strain, strain background (*S. cerevisiae*) | ybr161wΔ | YSC1053 Yeast MATa deletion collection |  | http://www-sequence.stanford.edu/group/yeast_deletion_project/deletions3.html |
| strain, strain background (*S. cerevisiae*) | ybr164cΔ | YSC1053 Yeast MATa deletion collection |  | http://www-sequence.stanford.edu/group/yeast_deletion_project/deletions3.html |
| strain, strain background (*S. cerevisiae*) | ybr168wΔ | YSC1053 Yeast MATa deletion collection |  | http://www-sequence.stanford.edu/group/yeast_deletion_project/deletions3.html |
| strain, strain background (*S. cerevisiae*) | ybr169cΔ | YSC1053 Yeast MATa deletion collection |  | http://www-sequence.stanford.edu/group/yeast_deletion_project/deletions3.html |
| strain, strain background (*S. cerevisiae*) | ybr171wΔ | YSC1053 Yeast MATa deletion collection |  | http://www-sequence.stanford.edu/group/yeast_deletion_project/deletions3.html |
| strain, strain background (*S. cerevisiae*) | ybr173cΔ | YSC1053 Yeast MATa deletion collection |  | http://www-sequence.stanford.edu/group/yeast_deletion_project/deletions3.html |
| strain, strain background (*S. cerevisiae*) | ybr174cΔ | YSC1053 Yeast MATa deletion collection |  | http://www-sequence.stanford.edu/group/yeast_deletion_project/deletions3.html |
| strain, strain background (*S. cerevisiae*) | ybr175wΔ | YSC1053 Yeast MATa deletion collection |  | http://www-sequence.stanford.edu/group/yeast_deletion_project/deletions3.html |
| strain, strain background (*S. cerevisiae*) | ybr181cΔ | YSC1053 Yeast MATa deletion collection |  | http://www-sequence.stanford.edu/group/yeast_deletion_project/deletions3.html |
| strain, strain background (*S. cerevisiae*) | ybr182cΔ | YSC1053 Yeast MATa deletion collection |  | http://www-sequence.stanford.edu/group/yeast_deletion_project/deletions3.html |
| strain, strain background (*S. cerevisiae*) | ybr189wΔ | YSC1053 Yeast MATa deletion collection |  | http://www-sequence.stanford.edu/group/yeast_deletion_project/deletions3.html |
| strain, strain background (*S. cerevisiae*) | ybr194wΔ | YSC1053 Yeast MATa deletion collection |  | http://www-sequence.stanford.edu/group/yeast_deletion_project/deletions3.html |
| strain, strain background (*S. cerevisiae*) | ybr195cΔ | YSC1053 Yeast MATa deletion collection |  | http://www-sequence.stanford.edu/group/yeast_deletion_project/deletions3.html |
| strain, strain background (*S. cerevisiae*) | ybr196c-aΔ | YSC1053 Yeast MATa deletion collection |  | http://www-sequence.stanford.edu/group/yeast_deletion_project/deletions3.html |
| strain, strain background (*S. cerevisiae*) | ybr200wΔ | YSC1053 Yeast MATa deletion collection |  | http://www-sequence.stanford.edu/group/yeast_deletion_project/deletions3.html |
| strain, strain background (*S. cerevisiae*) | ybr212wΔ | YSC1053 Yeast MATa deletion collection |  | http://www-sequence.stanford.edu/group/yeast_deletion_project/deletions3.html |
| strain, strain background (*S. cerevisiae*) | ybr216cΔ | YSC1053 Yeast MATa deletion collection |  | http://www-sequence.stanford.edu/group/yeast_deletion_project/deletions3.html |
| strain, strain background (*S. cerevisiae*) | ybr221cΔ | YSC1053 Yeast MATa deletion collection |  | http://www-sequence.stanford.edu/group/yeast_deletion_project/deletions3.html |
| strain, strain background (*S. cerevisiae*) | ybr224wΔ | YSC1053 Yeast MATa deletion collection |  | http://www-sequence.stanford.edu/group/yeast_deletion_project/deletions3.html |
| strain, strain background (*S. cerevisiae*) | ybr225wΔ | YSC1053 Yeast MATa deletion collection |  | http://www-sequence.stanford.edu/group/yeast_deletion_project/deletions3.html |
| strain, strain background (*S. cerevisiae*) | ybr226cΔ | YSC1053 Yeast MATa deletion collection |  | http://www-sequence.stanford.edu/group/yeast_deletion_project/deletions3.html |
| strain, strain background (*S. cerevisiae*) | ybr231cΔ | YSC1053 Yeast MATa deletion collection |  | http://www-sequence.stanford.edu/group/yeast_deletion_project/deletions3.html |
| strain, strain background (*S. cerevisiae*) | ybr250wΔ | YSC1053 Yeast MATa deletion collection |  | http://www-sequence.stanford.edu/group/yeast_deletion_project/deletions3.html |
| strain, strain background (*S. cerevisiae*) | ybr251wΔ | YSC1053 Yeast MATa deletion collection |  | http://www-sequence.stanford.edu/group/yeast_deletion_project/deletions3.html |
| strain, strain background (*S. cerevisiae*) | ybr264cΔ | YSC1053 Yeast MATa deletion collection |  | http://www-sequence.stanford.edu/group/yeast_deletion_project/deletions3.html |
| strain, strain background (*S. cerevisiae*) | ybr266cΔ | YSC1053 Yeast MATa deletion collection |  | http://www-sequence.stanford.edu/group/yeast_deletion_project/deletions3.html |
| strain, strain background (*S. cerevisiae*) | ybr267wΔ | YSC1053 Yeast MATa deletion collection |  | http://www-sequence.stanford.edu/group/yeast_deletion_project/deletions3.html |
| strain, strain background (*S. cerevisiae*) | ybr268wΔ | YSC1053 Yeast MATa deletion collection |  | http://www-sequence.stanford.edu/group/yeast_deletion_project/deletions3.html |
| strain, strain background (*S. cerevisiae*) | ybr277cΔ | YSC1053 Yeast MATa deletion collection |  | http://www-sequence.stanford.edu/group/yeast_deletion_project/deletions3.html |
| strain, strain background (*S. cerevisiae*) | ybr279wΔ | YSC1053 Yeast MATa deletion collection |  | http://www-sequence.stanford.edu/group/yeast_deletion_project/deletions3.html |
| strain, strain background (*S. cerevisiae*) | ybr281cΔ | YSC1053 Yeast MATa deletion collection |  | http://www-sequence.stanford.edu/group/yeast_deletion_project/deletions3.html |
| strain, strain background (*S. cerevisiae*) | ybr282wΔ | YSC1053 Yeast MATa deletion collection |  | http://www-sequence.stanford.edu/group/yeast_deletion_project/deletions3.html |
| strain, strain background (*S. cerevisiae*) | ybr289wΔ | YSC1053 Yeast MATa deletion collection |  | http://www-sequence.stanford.edu/group/yeast_deletion_project/deletions3.html |
| strain, strain background (*S. cerevisiae*) | ybr292cΔ | YSC1053 Yeast MATa deletion collection |  | http://www-sequence.stanford.edu/group/yeast_deletion_project/deletions3.html |
| strain, strain background (*S. cerevisiae*) | ybr295wΔ | YSC1053 Yeast MATa deletion collection |  | http://www-sequence.stanford.edu/group/yeast_deletion_project/deletions3.html |
| strain, strain background (*S. cerevisiae*) | ybr296cΔ | YSC1053 Yeast MATa deletion collection |  | http://www-sequence.stanford.edu/group/yeast_deletion_project/deletions3.html |
| strain, strain background (*S. cerevisiae*) | ybr297wΔ | YSC1053 Yeast MATa deletion collection |  | http://www-sequence.stanford.edu/group/yeast_deletion_project/deletions3.html |
| strain, strain background (*S. cerevisiae*) | ycl008cΔ | YSC1053 Yeast MATa deletion collection |  | http://www-sequence.stanford.edu/group/yeast_deletion_project/deletions3.html |
| strain, strain background (*S. cerevisiae*) | ycl009cΔ | YSC1053 Yeast MATa deletion collection |  | http://www-sequence.stanford.edu/group/yeast_deletion_project/deletions3.html |
| strain, strain background (*S. cerevisiae*) | ycl010cΔ | YSC1053 Yeast MATa deletion collection |  | http://www-sequence.stanford.edu/group/yeast_deletion_project/deletions3.html |
| strain, strain background (*S. cerevisiae*) | ycl016cΔ | YSC1053 Yeast MATa deletion collection |  | http://www-sequence.stanford.edu/group/yeast_deletion_project/deletions3.html |
| strain, strain background (*S. cerevisiae*) | ycl023cΔ | YSC1053 Yeast MATa deletion collection |  | http://www-sequence.stanford.edu/group/yeast_deletion_project/deletions3.html |
| strain, strain background (*S. cerevisiae*) | ycl025cΔ | YSC1053 Yeast MATa deletion collection |  | http://www-sequence.stanford.edu/group/yeast_deletion_project/deletions3.html |
| strain, strain background (*S. cerevisiae*) | ycl027wΔ | YSC1053 Yeast MATa deletion collection |  | http://www-sequence.stanford.edu/group/yeast_deletion_project/deletions3.html |
| strain, strain background (*S. cerevisiae*) | ycl029cΔ | YSC1053 Yeast MATa deletion collection |  | http://www-sequence.stanford.edu/group/yeast_deletion_project/deletions3.html |
| strain, strain background (*S. cerevisiae*) | ycl032wΔ | YSC1053 Yeast MATa deletion collection |  | http://www-sequence.stanford.edu/group/yeast_deletion_project/deletions3.html |
| strain, strain background (*S. cerevisiae*) | ycl033cΔ | YSC1053 Yeast MATa deletion collection |  | http://www-sequence.stanford.edu/group/yeast_deletion_project/deletions3.html |
| strain, strain background (*S. cerevisiae*) | ycl037cΔ | YSC1053 Yeast MATa deletion collection |  | http://www-sequence.stanford.edu/group/yeast_deletion_project/deletions3.html |
| strain, strain background (*S. cerevisiae*) | ycl050cΔ | YSC1053 Yeast MATa deletion collection |  | http://www-sequence.stanford.edu/group/yeast_deletion_project/deletions3.html |
| strain, strain background (*S. cerevisiae*) | ycl051wΔ | YSC1053 Yeast MATa deletion collection |  | http://www-sequence.stanford.edu/group/yeast_deletion_project/deletions3.html |
| strain, strain background (*S. cerevisiae*) | ycl061cΔ | YSC1053 Yeast MATa deletion collection |  | http://www-sequence.stanford.edu/group/yeast_deletion_project/deletions3.html |
| strain, strain background (*S. cerevisiae*) | ycl062wΔ | YSC1053 Yeast MATa deletion collection |  | http://www-sequence.stanford.edu/group/yeast_deletion_project/deletions3.html |
| strain, strain background (*S. cerevisiae*) | ycl063wΔ | YSC1053 Yeast MATa deletion collection |  | http://www-sequence.stanford.edu/group/yeast_deletion_project/deletions3.html |
| strain, strain background (*S. cerevisiae*) | ycr001wΔ | YSC1053 Yeast MATa deletion collection |  | http://www-sequence.stanford.edu/group/yeast_deletion_project/deletions3.html |
| strain, strain background (*S. cerevisiae*) | ycr002cΔ | YSC1053 Yeast MATa deletion collection |  | http://www-sequence.stanford.edu/group/yeast_deletion_project/deletions3.html |
| strain, strain background (*S. cerevisiae*) | ycr003wΔ | YSC1053 Yeast MATa deletion collection |  | http://www-sequence.stanford.edu/group/yeast_deletion_project/deletions3.html |
| strain, strain background (*S. cerevisiae*) | ycr008wΔ | YSC1053 Yeast MATa deletion collection |  | http://www-sequence.stanford.edu/group/yeast_deletion_project/deletions3.html |
| strain, strain background (*S. cerevisiae*) | ycr009cΔ | YSC1053 Yeast MATa deletion collection |  | http://www-sequence.stanford.edu/group/yeast_deletion_project/deletions3.html |
| strain, strain background (*S. cerevisiae*) | ycr010cΔ | YSC1053 Yeast MATa deletion collection |  | http://www-sequence.stanford.edu/group/yeast_deletion_project/deletions3.html |
| strain, strain background (*S. cerevisiae*) | ycr019wΔ | YSC1053 Yeast MATa deletion collection |  | http://www-sequence.stanford.edu/group/yeast_deletion_project/deletions3.html |
| strain, strain background (*S. cerevisiae*) | ycr022cΔ | YSC1053 Yeast MATa deletion collection |  | http://www-sequence.stanford.edu/group/yeast_deletion_project/deletions3.html |
| strain, strain background (*S. cerevisiae*) | ycr024cΔ | YSC1053 Yeast MATa deletion collection |  | http://www-sequence.stanford.edu/group/yeast_deletion_project/deletions3.html |
| strain, strain background (*S. cerevisiae*) | ycr027cΔ | YSC1053 Yeast MATa deletion collection |  | http://www-sequence.stanford.edu/group/yeast_deletion_project/deletions3.html |
| strain, strain background (*S. cerevisiae*) | ycr028cΔ | YSC1053 Yeast MATa deletion collection |  | http://www-sequence.stanford.edu/group/yeast_deletion_project/deletions3.html |
| strain, strain background (*S. cerevisiae*) | ycr028c-aΔ | YSC1053 Yeast MATa deletion collection |  | http://www-sequence.stanford.edu/group/yeast_deletion_project/deletions3.html |
| strain, strain background (*S. cerevisiae*) | ycr030cΔ | YSC1053 Yeast MATa deletion collection |  | http://www-sequence.stanford.edu/group/yeast_deletion_project/deletions3.html |
| strain, strain background (*S. cerevisiae*) | ycr031cΔ | YSC1053 Yeast MATa deletion collection |  | http://www-sequence.stanford.edu/group/yeast_deletion_project/deletions3.html |
| strain, strain background (*S. cerevisiae*) | ycr032wΔ | YSC1053 Yeast MATa deletion collection |  | http://www-sequence.stanford.edu/group/yeast_deletion_project/deletions3.html |
| strain, strain background (*S. cerevisiae*) | ycr034wΔ | YSC1053 Yeast MATa deletion collection |  | http://www-sequence.stanford.edu/group/yeast_deletion_project/deletions3.html |
| strain, strain background (*S. cerevisiae*) | ycr046cΔ | YSC1053 Yeast MATa deletion collection |  | http://www-sequence.stanford.edu/group/yeast_deletion_project/deletions3.html |
| strain, strain background (*S. cerevisiae*) | ycr047cΔ | YSC1053 Yeast MATa deletion collection |  | http://www-sequence.stanford.edu/group/yeast_deletion_project/deletions3.html |
| strain, strain background (*S. cerevisiae*) | ycr049cΔ | YSC1053 Yeast MATa deletion collection |  | http://www-sequence.stanford.edu/group/yeast_deletion_project/deletions3.html |
| strain, strain background (*S. cerevisiae*) | ycr053wΔ | YSC1053 Yeast MATa deletion collection |  | http://www-sequence.stanford.edu/group/yeast_deletion_project/deletions3.html |
| strain, strain background (*S. cerevisiae*) | ycr059cΔ | YSC1053 Yeast MATa deletion collection |  | http://www-sequence.stanford.edu/group/yeast_deletion_project/deletions3.html |
| strain, strain background (*S. cerevisiae*) | ycr063wΔ | YSC1053 Yeast MATa deletion collection |  | http://www-sequence.stanford.edu/group/yeast_deletion_project/deletions3.html |
| strain, strain background (*S. cerevisiae*) | ycr066wΔ | YSC1053 Yeast MATa deletion collection |  | http://www-sequence.stanford.edu/group/yeast_deletion_project/deletions3.html |
| strain, strain background (*S. cerevisiae*) | ycr073w-aΔ | YSC1053 Yeast MATa deletion collection |  | http://www-sequence.stanford.edu/group/yeast_deletion_project/deletions3.html |
| strain, strain background (*S. cerevisiae*) | ycr075cΔ | YSC1053 Yeast MATa deletion collection |  | http://www-sequence.stanford.edu/group/yeast_deletion_project/deletions3.html |
| strain, strain background (*S. cerevisiae*) | ycr077cΔ | YSC1053 Yeast MATa deletion collection |  | http://www-sequence.stanford.edu/group/yeast_deletion_project/deletions3.html |
| strain, strain background (*S. cerevisiae*) | ycr079wΔ | YSC1053 Yeast MATa deletion collection |  | http://www-sequence.stanford.edu/group/yeast_deletion_project/deletions3.html |
| strain, strain background (*S. cerevisiae*) | ycr094wΔ | YSC1053 Yeast MATa deletion collection |  | http://www-sequence.stanford.edu/group/yeast_deletion_project/deletions3.html |
| strain, strain background (*S. cerevisiae*) | ycr106wΔ | YSC1053 Yeast MATa deletion collection |  | http://www-sequence.stanford.edu/group/yeast_deletion_project/deletions3.html |
| strain, strain background (*S. cerevisiae*) | ydl002cΔ | YSC1053 Yeast MATa deletion collection |  | http://www-sequence.stanford.edu/group/yeast_deletion_project/deletions3.html |
| strain, strain background (*S. cerevisiae*) | ydl006wΔ | YSC1053 Yeast MATa deletion collection |  | http://www-sequence.stanford.edu/group/yeast_deletion_project/deletions3.html |
| strain, strain background (*S. cerevisiae*) | ydl009cΔ | YSC1053 Yeast MATa deletion collection |  | http://www-sequence.stanford.edu/group/yeast_deletion_project/deletions3.html |
| strain, strain background (*S. cerevisiae*) | ydl012cΔ | YSC1053 Yeast MATa deletion collection |  | http://www-sequence.stanford.edu/group/yeast_deletion_project/deletions3.html |
| strain, strain background (*S. cerevisiae*) | ydl013wΔ | YSC1053 Yeast MATa deletion collection |  | http://www-sequence.stanford.edu/group/yeast_deletion_project/deletions3.html |
| strain, strain background (*S. cerevisiae*) | ydl018cΔ | YSC1053 Yeast MATa deletion collection |  | http://www-sequence.stanford.edu/group/yeast_deletion_project/deletions3.html |
| strain, strain background (*S. cerevisiae*) | ydl020cΔ | YSC1053 Yeast MATa deletion collection |  | http://www-sequence.stanford.edu/group/yeast_deletion_project/deletions3.html |
| strain, strain background (*S. cerevisiae*) | ydl021wΔ | YSC1053 Yeast MATa deletion collection |  | http://www-sequence.stanford.edu/group/yeast_deletion_project/deletions3.html |
| strain, strain background (*S. cerevisiae*) | ydl023cΔ | YSC1053 Yeast MATa deletion collection |  | http://www-sequence.stanford.edu/group/yeast_deletion_project/deletions3.html |
| strain, strain background (*S. cerevisiae*) | ydl025cΔ | YSC1053 Yeast MATa deletion collection |  | http://www-sequence.stanford.edu/group/yeast_deletion_project/deletions3.html |
| strain, strain background (*S. cerevisiae*) | ydl026wΔ | YSC1053 Yeast MATa deletion collection |  | http://www-sequence.stanford.edu/group/yeast_deletion_project/deletions3.html |
| strain, strain background (*S. cerevisiae*) | ydl027cΔ | YSC1053 Yeast MATa deletion collection |  | http://www-sequence.stanford.edu/group/yeast_deletion_project/deletions3.html |
| strain, strain background (*S. cerevisiae*) | ydl033cΔ | YSC1053 Yeast MATa deletion collection |  | http://www-sequence.stanford.edu/group/yeast_deletion_project/deletions3.html |
| strain, strain background (*S. cerevisiae*) | ydl037cΔ | YSC1053 Yeast MATa deletion collection |  | http://www-sequence.stanford.edu/group/yeast_deletion_project/deletions3.html |
| strain, strain background (*S. cerevisiae*) | ydl040cΔ | YSC1053 Yeast MATa deletion collection |  | http://www-sequence.stanford.edu/group/yeast_deletion_project/deletions3.html |
| strain, strain background (*S. cerevisiae*) | ydl042cΔ | YSC1053 Yeast MATa deletion collection |  | http://www-sequence.stanford.edu/group/yeast_deletion_project/deletions3.html |
| strain, strain background (*S. cerevisiae*) | ydl044cΔ | YSC1053 Yeast MATa deletion collection |  | http://www-sequence.stanford.edu/group/yeast_deletion_project/deletions3.html |
| strain, strain background (*S. cerevisiae*) | ydl045w-aΔ | YSC1053 Yeast MATa deletion collection |  | http://www-sequence.stanford.edu/group/yeast_deletion_project/deletions3.html |
| strain, strain background (*S. cerevisiae*) | ydl047wΔ | YSC1053 Yeast MATa deletion collection |  | http://www-sequence.stanford.edu/group/yeast_deletion_project/deletions3.html |
| strain, strain background (*S. cerevisiae*) | ydl050cΔ | YSC1053 Yeast MATa deletion collection |  | http://www-sequence.stanford.edu/group/yeast_deletion_project/deletions3.html |
| strain, strain background (*S. cerevisiae*) | ydl051wΔ | YSC1053 Yeast MATa deletion collection |  | http://www-sequence.stanford.edu/group/yeast_deletion_project/deletions3.html |
| strain, strain background (*S. cerevisiae*) | ydl052cΔ | YSC1053 Yeast MATa deletion collection |  | http://www-sequence.stanford.edu/group/yeast_deletion_project/deletions3.html |
| strain, strain background (*S. cerevisiae*) | ydl061cΔ | YSC1053 Yeast MATa deletion collection |  | http://www-sequence.stanford.edu/group/yeast_deletion_project/deletions3.html |
| strain, strain background (*S. cerevisiae*) | ydl063cΔ | YSC1053 Yeast MATa deletion collection |  | http://www-sequence.stanford.edu/group/yeast_deletion_project/deletions3.html |
| strain, strain background (*S. cerevisiae*) | ydl065cΔ | YSC1053 Yeast MATa deletion collection |  | http://www-sequence.stanford.edu/group/yeast_deletion_project/deletions3.html |
| strain, strain background (*S. cerevisiae*) | ydl067cΔ | YSC1053 Yeast MATa deletion collection |  | http://www-sequence.stanford.edu/group/yeast_deletion_project/deletions3.html |
| strain, strain background (*S. cerevisiae*) | ydl069cΔ | YSC1053 Yeast MATa deletion collection |  | http://www-sequence.stanford.edu/group/yeast_deletion_project/deletions3.html |
| strain, strain background (*S. cerevisiae*) | ydl076cΔ | YSC1053 Yeast MATa deletion collection |  | http://www-sequence.stanford.edu/group/yeast_deletion_project/deletions3.html |
| strain, strain background (*S. cerevisiae*) | ydl077cΔ | YSC1053 Yeast MATa deletion collection |  | http://www-sequence.stanford.edu/group/yeast_deletion_project/deletions3.html |
| strain, strain background (*S. cerevisiae*) | ydl081cΔ | YSC1053 Yeast MATa deletion collection |  | http://www-sequence.stanford.edu/group/yeast_deletion_project/deletions3.html |
| strain, strain background (*S. cerevisiae*) | ydl082wΔ | YSC1053 Yeast MATa deletion collection |  | http://www-sequence.stanford.edu/group/yeast_deletion_project/deletions3.html |
| strain, strain background (*S. cerevisiae*) | ydl083cΔ | YSC1053 Yeast MATa deletion collection |  | http://www-sequence.stanford.edu/group/yeast_deletion_project/deletions3.html |
| strain, strain background (*S. cerevisiae*) | ydl085wΔ | YSC1053 Yeast MATa deletion collection |  | http://www-sequence.stanford.edu/group/yeast_deletion_project/deletions3.html |
| strain, strain background (*S. cerevisiae*) | ydl088cΔ | YSC1053 Yeast MATa deletion collection |  | http://www-sequence.stanford.edu/group/yeast_deletion_project/deletions3.html |
| strain, strain background (*S. cerevisiae*) | ydl090cΔ | YSC1053 Yeast MATa deletion collection |  | http://www-sequence.stanford.edu/group/yeast_deletion_project/deletions3.html |
| strain, strain background (*S. cerevisiae*) | ydl091cΔ | YSC1053 Yeast MATa deletion collection |  | http://www-sequence.stanford.edu/group/yeast_deletion_project/deletions3.html |
| strain, strain background (*S. cerevisiae*) | ydl094cΔ | YSC1053 Yeast MATa deletion collection |  | http://www-sequence.stanford.edu/group/yeast_deletion_project/deletions3.html |
| strain, strain background (*S. cerevisiae*) | ydl101cΔ | YSC1053 Yeast MATa deletion collection |  | http://www-sequence.stanford.edu/group/yeast_deletion_project/deletions3.html |
| strain, strain background (*S. cerevisiae*) | ydl104cΔ | YSC1053 Yeast MATa deletion collection |  | http://www-sequence.stanford.edu/group/yeast_deletion_project/deletions3.html |
| strain, strain background (*S. cerevisiae*) | ydl107wΔ | YSC1053 Yeast MATa deletion collection |  | http://www-sequence.stanford.edu/group/yeast_deletion_project/deletions3.html |
| strain, strain background (*S. cerevisiae*) | ydl113cΔ | YSC1053 Yeast MATa deletion collection |  | http://www-sequence.stanford.edu/group/yeast_deletion_project/deletions3.html |
| strain, strain background (*S. cerevisiae*) | ydl114wΔ | YSC1053 Yeast MATa deletion collection |  | http://www-sequence.stanford.edu/group/yeast_deletion_project/deletions3.html |
| strain, strain background (*S. cerevisiae*) | ydl115cΔ | YSC1053 Yeast MATa deletion collection |  | http://www-sequence.stanford.edu/group/yeast_deletion_project/deletions3.html |
| strain, strain background (*S. cerevisiae*) | ydl116wΔ | YSC1053 Yeast MATa deletion collection |  | http://www-sequence.stanford.edu/group/yeast_deletion_project/deletions3.html |
| strain, strain background (*S. cerevisiae*) | ydl117wΔ | YSC1053 Yeast MATa deletion collection |  | http://www-sequence.stanford.edu/group/yeast_deletion_project/deletions3.html |
| strain, strain background (*S. cerevisiae*) | ydl119cΔ | YSC1053 Yeast MATa deletion collection |  | http://www-sequence.stanford.edu/group/yeast_deletion_project/deletions3.html |
| strain, strain background (*S. cerevisiae*) | ydl121cΔ | YSC1053 Yeast MATa deletion collection |  | http://www-sequence.stanford.edu/group/yeast_deletion_project/deletions3.html |
| strain, strain background (*S. cerevisiae*) | ydl130wΔ | YSC1053 Yeast MATa deletion collection |  | http://www-sequence.stanford.edu/group/yeast_deletion_project/deletions3.html |
| strain, strain background (*S. cerevisiae*) | ydl135cΔ | YSC1053 Yeast MATa deletion collection |  | http://www-sequence.stanford.edu/group/yeast_deletion_project/deletions3.html |
| strain, strain background (*S. cerevisiae*) | ydl136wΔ | YSC1053 Yeast MATa deletion collection |  | http://www-sequence.stanford.edu/group/yeast_deletion_project/deletions3.html |
| strain, strain background (*S. cerevisiae*) | ydl142cΔ | YSC1053 Yeast MATa deletion collection |  | http://www-sequence.stanford.edu/group/yeast_deletion_project/deletions3.html |
| strain, strain background (*S. cerevisiae*) | ydl151cΔ | YSC1053 Yeast MATa deletion collection |  | http://www-sequence.stanford.edu/group/yeast_deletion_project/deletions3.html |
| strain, strain background (*S. cerevisiae*) | ydl154wΔ | YSC1053 Yeast MATa deletion collection |  | http://www-sequence.stanford.edu/group/yeast_deletion_project/deletions3.html |
| strain, strain background (*S. cerevisiae*) | ydl156wΔ | YSC1053 Yeast MATa deletion collection |  | http://www-sequence.stanford.edu/group/yeast_deletion_project/deletions3.html |
| strain, strain background (*S. cerevisiae*) | ydl157cΔ | YSC1053 Yeast MATa deletion collection |  | http://www-sequence.stanford.edu/group/yeast_deletion_project/deletions3.html |
| strain, strain background (*S. cerevisiae*) | ydl159wΔ | YSC1053 Yeast MATa deletion collection |  | http://www-sequence.stanford.edu/group/yeast_deletion_project/deletions3.html |
| strain, strain background (*S. cerevisiae*) | ydl160cΔ | YSC1053 Yeast MATa deletion collection |  | http://www-sequence.stanford.edu/group/yeast_deletion_project/deletions3.html |
| strain, strain background (*S. cerevisiae*) | ydl167cΔ | YSC1053 Yeast MATa deletion collection |  | http://www-sequence.stanford.edu/group/yeast_deletion_project/deletions3.html |
| strain, strain background (*S. cerevisiae*) | ydl177cΔ | YSC1053 Yeast MATa deletion collection |  | http://www-sequence.stanford.edu/group/yeast_deletion_project/deletions3.html |
| strain, strain background (*S. cerevisiae*) | ydl180wΔ | YSC1053 Yeast MATa deletion collection |  | http://www-sequence.stanford.edu/group/yeast_deletion_project/deletions3.html |
| strain, strain background (*S. cerevisiae*) | ydl182wΔ | YSC1053 Yeast MATa deletion collection |  | http://www-sequence.stanford.edu/group/yeast_deletion_project/deletions3.html |
| strain, strain background (*S. cerevisiae*) | ydl186wΔ | YSC1053 Yeast MATa deletion collection |  | http://www-sequence.stanford.edu/group/yeast_deletion_project/deletions3.html |
| strain, strain background (*S. cerevisiae*) | ydl189wΔ | YSC1053 Yeast MATa deletion collection |  | http://www-sequence.stanford.edu/group/yeast_deletion_project/deletions3.html |
| strain, strain background (*S. cerevisiae*) | ydl190cΔ | YSC1053 Yeast MATa deletion collection |  | http://www-sequence.stanford.edu/group/yeast_deletion_project/deletions3.html |
| strain, strain background (*S. cerevisiae*) | ydl191wΔ | YSC1053 Yeast MATa deletion collection |  | http://www-sequence.stanford.edu/group/yeast_deletion_project/deletions3.html |
| strain, strain background (*S. cerevisiae*) | ydl192wΔ | YSC1053 Yeast MATa deletion collection |  | http://www-sequence.stanford.edu/group/yeast_deletion_project/deletions3.html |
| strain, strain background (*S. cerevisiae*) | ydl194wΔ | YSC1053 Yeast MATa deletion collection |  | http://www-sequence.stanford.edu/group/yeast_deletion_project/deletions3.html |
| strain, strain background (*S. cerevisiae*) | ydl201wΔ | YSC1053 Yeast MATa deletion collection |  | http://www-sequence.stanford.edu/group/yeast_deletion_project/deletions3.html |
| strain, strain background (*S. cerevisiae*) | ydl202wΔ | YSC1053 Yeast MATa deletion collection |  | http://www-sequence.stanford.edu/group/yeast_deletion_project/deletions3.html |
| strain, strain background (*S. cerevisiae*) | ydl213cΔ | YSC1053 Yeast MATa deletion collection |  | http://www-sequence.stanford.edu/group/yeast_deletion_project/deletions3.html |
| strain, strain background (*S. cerevisiae*) | ydl226cΔ | YSC1053 Yeast MATa deletion collection |  | http://www-sequence.stanford.edu/group/yeast_deletion_project/deletions3.html |
| strain, strain background (*S. cerevisiae*) | ydl232wΔ | YSC1053 Yeast MATa deletion collection |  | http://www-sequence.stanford.edu/group/yeast_deletion_project/deletions3.html |
| strain, strain background (*S. cerevisiae*) | ydl243cΔ | YSC1053 Yeast MATa deletion collection |  | http://www-sequence.stanford.edu/group/yeast_deletion_project/deletions3.html |
| strain, strain background (*S. cerevisiae*) | ydr001cΔ | YSC1053 Yeast MATa deletion collection |  | http://www-sequence.stanford.edu/group/yeast_deletion_project/deletions3.html |
| strain, strain background (*S. cerevisiae*) | ydr004wΔ | YSC1053 Yeast MATa deletion collection |  | http://www-sequence.stanford.edu/group/yeast_deletion_project/deletions3.html |
| strain, strain background (*S. cerevisiae*) | ydr005cΔ | YSC1053 Yeast MATa deletion collection |  | http://www-sequence.stanford.edu/group/yeast_deletion_project/deletions3.html |
| strain, strain background (*S. cerevisiae*) | ydr017cΔ | YSC1053 Yeast MATa deletion collection |  | http://www-sequence.stanford.edu/group/yeast_deletion_project/deletions3.html |
| strain, strain background (*S. cerevisiae*) | ydr024wΔ | YSC1053 Yeast MATa deletion collection |  | http://www-sequence.stanford.edu/group/yeast_deletion_project/deletions3.html |
| strain, strain background (*S. cerevisiae*) | ydr025wΔ | YSC1053 Yeast MATa deletion collection |  | http://www-sequence.stanford.edu/group/yeast_deletion_project/deletions3.html |
| strain, strain background (*S. cerevisiae*) | ydr027cΔ | YSC1053 Yeast MATa deletion collection |  | http://www-sequence.stanford.edu/group/yeast_deletion_project/deletions3.html |
| strain, strain background (*S. cerevisiae*) | ydr049wΔ | YSC1053 Yeast MATa deletion collection |  | http://www-sequence.stanford.edu/group/yeast_deletion_project/deletions3.html |
| strain, strain background (*S. cerevisiae*) | ydr065wΔ | YSC1053 Yeast MATa deletion collection |  | http://www-sequence.stanford.edu/group/yeast_deletion_project/deletions3.html |
| strain, strain background (*S. cerevisiae*) | ydr069cΔ | YSC1053 Yeast MATa deletion collection |  | http://www-sequence.stanford.edu/group/yeast_deletion_project/deletions3.html |
| strain, strain background (*S. cerevisiae*) | ydr073wΔ | YSC1053 Yeast MATa deletion collection |  | http://www-sequence.stanford.edu/group/yeast_deletion_project/deletions3.html |
| strain, strain background (*S. cerevisiae*) | ydr074wΔ | YSC1053 Yeast MATa deletion collection |  | http://www-sequence.stanford.edu/group/yeast_deletion_project/deletions3.html |
| strain, strain background (*S. cerevisiae*) | ydr075wΔ | YSC1053 Yeast MATa deletion collection |  | http://www-sequence.stanford.edu/group/yeast_deletion_project/deletions3.html |
| strain, strain background (*S. cerevisiae*) | ydr076wΔ | YSC1053 Yeast MATa deletion collection |  | http://www-sequence.stanford.edu/group/yeast_deletion_project/deletions3.html |
| strain, strain background (*S. cerevisiae*) | ydr078cΔ | YSC1053 Yeast MATa deletion collection |  | http://www-sequence.stanford.edu/group/yeast_deletion_project/deletions3.html |
| strain, strain background (*S. cerevisiae*) | ydr079wΔ | YSC1053 Yeast MATa deletion collection |  | http://www-sequence.stanford.edu/group/yeast_deletion_project/deletions3.html |
| strain, strain background (*S. cerevisiae*) | ydr080wΔ | YSC1053 Yeast MATa deletion collection |  | http://www-sequence.stanford.edu/group/yeast_deletion_project/deletions3.html |
| strain, strain background (*S. cerevisiae*) | ydr083wΔ | YSC1053 Yeast MATa deletion collection |  | http://www-sequence.stanford.edu/group/yeast_deletion_project/deletions3.html |
| strain, strain background (*S. cerevisiae*) | ydr092wΔ | YSC1053 Yeast MATa deletion collection |  | http://www-sequence.stanford.edu/group/yeast_deletion_project/deletions3.html |
| strain, strain background (*S. cerevisiae*) | ydr096wΔ | YSC1053 Yeast MATa deletion collection |  | http://www-sequence.stanford.edu/group/yeast_deletion_project/deletions3.html |
| strain, strain background (*S. cerevisiae*) | ydr101cΔ | YSC1053 Yeast MATa deletion collection |  | http://www-sequence.stanford.edu/group/yeast_deletion_project/deletions3.html |
| strain, strain background (*S. cerevisiae*) | ydr103wΔ | YSC1053 Yeast MATa deletion collection |  | http://www-sequence.stanford.edu/group/yeast_deletion_project/deletions3.html |
| strain, strain background (*S. cerevisiae*) | ydr108wΔ | YSC1053 Yeast MATa deletion collection |  | http://www-sequence.stanford.edu/group/yeast_deletion_project/deletions3.html |
| strain, strain background (*S. cerevisiae*) | ydr115wΔ | YSC1053 Yeast MATa deletion collection |  | http://www-sequence.stanford.edu/group/yeast_deletion_project/deletions3.html |
| strain, strain background (*S. cerevisiae*) | ydr116cΔ | YSC1053 Yeast MATa deletion collection |  | http://www-sequence.stanford.edu/group/yeast_deletion_project/deletions3.html |
| strain, strain background (*S. cerevisiae*) | ydr123cΔ | YSC1053 Yeast MATa deletion collection |  | http://www-sequence.stanford.edu/group/yeast_deletion_project/deletions3.html |
| strain, strain background (*S. cerevisiae*) | ydr126wΔ | YSC1053 Yeast MATa deletion collection |  | http://www-sequence.stanford.edu/group/yeast_deletion_project/deletions3.html |
| strain, strain background (*S. cerevisiae*) | ydr127wΔ | YSC1053 Yeast MATa deletion collection |  | http://www-sequence.stanford.edu/group/yeast_deletion_project/deletions3.html |
| strain, strain background (*S. cerevisiae*) | ydr128wΔ | YSC1053 Yeast MATa deletion collection |  | http://www-sequence.stanford.edu/group/yeast_deletion_project/deletions3.html |
| strain, strain background (*S. cerevisiae*) | ydr129cΔ | YSC1053 Yeast MATa deletion collection |  | http://www-sequence.stanford.edu/group/yeast_deletion_project/deletions3.html |
| strain, strain background (*S. cerevisiae*) | ydr137wΔ | YSC1053 Yeast MATa deletion collection |  | http://www-sequence.stanford.edu/group/yeast_deletion_project/deletions3.html |
| strain, strain background (*S. cerevisiae*) | ydr138wΔ | YSC1053 Yeast MATa deletion collection |  | http://www-sequence.stanford.edu/group/yeast_deletion_project/deletions3.html |
| strain, strain background (*S. cerevisiae*) | ydr140wΔ | YSC1053 Yeast MATa deletion collection |  | http://www-sequence.stanford.edu/group/yeast_deletion_project/deletions3.html |
| strain, strain background (*S. cerevisiae*) | ydr144cΔ | YSC1053 Yeast MATa deletion collection |  | http://www-sequence.stanford.edu/group/yeast_deletion_project/deletions3.html |
| strain, strain background (*S. cerevisiae*) | ydr146cΔ | YSC1053 Yeast MATa deletion collection |  | http://www-sequence.stanford.edu/group/yeast_deletion_project/deletions3.html |
| strain, strain background (*S. cerevisiae*) | ydr150wΔ | YSC1053 Yeast MATa deletion collection |  | http://www-sequence.stanford.edu/group/yeast_deletion_project/deletions3.html |
| strain, strain background (*S. cerevisiae*) | ydr151cΔ | YSC1053 Yeast MATa deletion collection |  | http://www-sequence.stanford.edu/group/yeast_deletion_project/deletions3.html |
| strain, strain background (*S. cerevisiae*) | ydr155cΔ | YSC1053 Yeast MATa deletion collection |  | http://www-sequence.stanford.edu/group/yeast_deletion_project/deletions3.html |
| strain, strain background (*S. cerevisiae*) | ydr156wΔ | YSC1053 Yeast MATa deletion collection |  | http://www-sequence.stanford.edu/group/yeast_deletion_project/deletions3.html |
| strain, strain background (*S. cerevisiae*) | ydr158wΔ | YSC1053 Yeast MATa deletion collection |  | http://www-sequence.stanford.edu/group/yeast_deletion_project/deletions3.html |
| strain, strain background (*S. cerevisiae*) | ydr159wΔ | YSC1053 Yeast MATa deletion collection |  | http://www-sequence.stanford.edu/group/yeast_deletion_project/deletions3.html |
| strain, strain background (*S. cerevisiae*) | ydr162cΔ | YSC1053 Yeast MATa deletion collection |  | http://www-sequence.stanford.edu/group/yeast_deletion_project/deletions3.html |
| strain, strain background (*S. cerevisiae*) | ydr169cΔ | YSC1053 Yeast MATa deletion collection |  | http://www-sequence.stanford.edu/group/yeast_deletion_project/deletions3.html |
| strain, strain background (*S. cerevisiae*) | ydr173cΔ | YSC1053 Yeast MATa deletion collection |  | http://www-sequence.stanford.edu/group/yeast_deletion_project/deletions3.html |
| strain, strain background (*S. cerevisiae*) | ydr174wΔ | YSC1053 Yeast MATa deletion collection |  | http://www-sequence.stanford.edu/group/yeast_deletion_project/deletions3.html |
| strain, strain background (*S. cerevisiae*) | ydr175cΔ | YSC1053 Yeast MATa deletion collection |  | http://www-sequence.stanford.edu/group/yeast_deletion_project/deletions3.html |
| strain, strain background (*S. cerevisiae*) | ydr176wΔ | YSC1053 Yeast MATa deletion collection |  | http://www-sequence.stanford.edu/group/yeast_deletion_project/deletions3.html |
| strain, strain background (*S. cerevisiae*) | ydr179w-aΔ | YSC1053 Yeast MATa deletion collection |  | http://www-sequence.stanford.edu/group/yeast_deletion_project/deletions3.html |
| strain, strain background (*S. cerevisiae*) | ydr184cΔ | YSC1053 Yeast MATa deletion collection |  | http://www-sequence.stanford.edu/group/yeast_deletion_project/deletions3.html |
| strain, strain background (*S. cerevisiae*) | ydr194cΔ | YSC1053 Yeast MATa deletion collection |  | http://www-sequence.stanford.edu/group/yeast_deletion_project/deletions3.html |
| strain, strain background (*S. cerevisiae*) | ydr195wΔ | YSC1053 Yeast MATa deletion collection |  | http://www-sequence.stanford.edu/group/yeast_deletion_project/deletions3.html |
| strain, strain background (*S. cerevisiae*) | ydr197wΔ | YSC1053 Yeast MATa deletion collection |  | http://www-sequence.stanford.edu/group/yeast_deletion_project/deletions3.html |
| strain, strain background (*S. cerevisiae*) | ydr200cΔ | YSC1053 Yeast MATa deletion collection |  | http://www-sequence.stanford.edu/group/yeast_deletion_project/deletions3.html |
| strain, strain background (*S. cerevisiae*) | ydr202cΔ | YSC1053 Yeast MATa deletion collection |  | http://www-sequence.stanford.edu/group/yeast_deletion_project/deletions3.html |
| strain, strain background (*S. cerevisiae*) | ydr203wΔ | YSC1053 Yeast MATa deletion collection |  | http://www-sequence.stanford.edu/group/yeast_deletion_project/deletions3.html |
| strain, strain background (*S. cerevisiae*) | ydr204wΔ | YSC1053 Yeast MATa deletion collection |  | http://www-sequence.stanford.edu/group/yeast_deletion_project/deletions3.html |
| strain, strain background (*S. cerevisiae*) | ydr207cΔ | YSC1053 Yeast MATa deletion collection |  | http://www-sequence.stanford.edu/group/yeast_deletion_project/deletions3.html |
| strain, strain background (*S. cerevisiae*) | ydr215cΔ | YSC1053 Yeast MATa deletion collection |  | http://www-sequence.stanford.edu/group/yeast_deletion_project/deletions3.html |
| strain, strain background (*S. cerevisiae*) | ydr216wΔ | YSC1053 Yeast MATa deletion collection |  | http://www-sequence.stanford.edu/group/yeast_deletion_project/deletions3.html |
| strain, strain background (*S. cerevisiae*) | ydr225wΔ | YSC1053 Yeast MATa deletion collection |  | http://www-sequence.stanford.edu/group/yeast_deletion_project/deletions3.html |
| strain, strain background (*S. cerevisiae*) | ydr226wΔ | YSC1053 Yeast MATa deletion collection |  | http://www-sequence.stanford.edu/group/yeast_deletion_project/deletions3.html |
| strain, strain background (*S. cerevisiae*) | ydr231cΔ | YSC1053 Yeast MATa deletion collection |  | http://www-sequence.stanford.edu/group/yeast_deletion_project/deletions3.html |
| strain, strain background (*S. cerevisiae*) | ydr237wΔ | YSC1053 Yeast MATa deletion collection |  | http://www-sequence.stanford.edu/group/yeast_deletion_project/deletions3.html |
| strain, strain background (*S. cerevisiae*) | ydr241wΔ | YSC1053 Yeast MATa deletion collection |  | http://www-sequence.stanford.edu/group/yeast_deletion_project/deletions3.html |
| strain, strain background (*S. cerevisiae*) | ydr244wΔ | YSC1053 Yeast MATa deletion collection |  | http://www-sequence.stanford.edu/group/yeast_deletion_project/deletions3.html |
| strain, strain background (*S. cerevisiae*) | ydr245wΔ | YSC1053 Yeast MATa deletion collection |  | http://www-sequence.stanford.edu/group/yeast_deletion_project/deletions3.html |
| strain, strain background (*S. cerevisiae*) | ydr260cΔ | YSC1053 Yeast MATa deletion collection |  | http://www-sequence.stanford.edu/group/yeast_deletion_project/deletions3.html |
| strain, strain background (*S. cerevisiae*) | ydr261cΔ | YSC1053 Yeast MATa deletion collection |  | http://www-sequence.stanford.edu/group/yeast_deletion_project/deletions3.html |
| strain, strain background (*S. cerevisiae*) | ydr264cΔ | YSC1053 Yeast MATa deletion collection |  | http://www-sequence.stanford.edu/group/yeast_deletion_project/deletions3.html |
| strain, strain background (*S. cerevisiae*) | ydr265wΔ | YSC1053 Yeast MATa deletion collection |  | http://www-sequence.stanford.edu/group/yeast_deletion_project/deletions3.html |
| strain, strain background (*S. cerevisiae*) | ydr266cΔ | YSC1053 Yeast MATa deletion collection |  | http://www-sequence.stanford.edu/group/yeast_deletion_project/deletions3.html |
| strain, strain background (*S. cerevisiae*) | ydr268wΔ | YSC1053 Yeast MATa deletion collection |  | http://www-sequence.stanford.edu/group/yeast_deletion_project/deletions3.html |
| strain, strain background (*S. cerevisiae*) | ydr275wΔ | YSC1053 Yeast MATa deletion collection |  | http://www-sequence.stanford.edu/group/yeast_deletion_project/deletions3.html |
| strain, strain background (*S. cerevisiae*) | ydr276cΔ | YSC1053 Yeast MATa deletion collection |  | http://www-sequence.stanford.edu/group/yeast_deletion_project/deletions3.html |
| strain, strain background (*S. cerevisiae*) | ydr285wΔ | YSC1053 Yeast MATa deletion collection |  | http://www-sequence.stanford.edu/group/yeast_deletion_project/deletions3.html |
| strain, strain background (*S. cerevisiae*) | ydr290wΔ | YSC1053 Yeast MATa deletion collection |  | http://www-sequence.stanford.edu/group/yeast_deletion_project/deletions3.html |
| strain, strain background (*S. cerevisiae*) | ydr293cΔ | YSC1053 Yeast MATa deletion collection |  | http://www-sequence.stanford.edu/group/yeast_deletion_project/deletions3.html |
| strain, strain background (*S. cerevisiae*) | ydr295cΔ | YSC1053 Yeast MATa deletion collection |  | http://www-sequence.stanford.edu/group/yeast_deletion_project/deletions3.html |
| strain, strain background (*S. cerevisiae*) | ydr296wΔ | YSC1053 Yeast MATa deletion collection |  | http://www-sequence.stanford.edu/group/yeast_deletion_project/deletions3.html |
| strain, strain background (*S. cerevisiae*) | ydr297wΔ | YSC1053 Yeast MATa deletion collection |  | http://www-sequence.stanford.edu/group/yeast_deletion_project/deletions3.html |
| strain, strain background (*S. cerevisiae*) | ydr298cΔ | YSC1053 Yeast MATa deletion collection |  | http://www-sequence.stanford.edu/group/yeast_deletion_project/deletions3.html |
| strain, strain background (*S. cerevisiae*) | ydr300cΔ | YSC1053 Yeast MATa deletion collection |  | http://www-sequence.stanford.edu/group/yeast_deletion_project/deletions3.html |
| strain, strain background (*S. cerevisiae*) | ydr315cΔ | YSC1053 Yeast MATa deletion collection |  | http://www-sequence.stanford.edu/group/yeast_deletion_project/deletions3.html |
| strain, strain background (*S. cerevisiae*) | ydr316wΔ | YSC1053 Yeast MATa deletion collection |  | http://www-sequence.stanford.edu/group/yeast_deletion_project/deletions3.html |
| strain, strain background (*S. cerevisiae*) | ydr317wΔ | YSC1053 Yeast MATa deletion collection |  | http://www-sequence.stanford.edu/group/yeast_deletion_project/deletions3.html |
| strain, strain background (*S. cerevisiae*) | ydr320cΔ | YSC1053 Yeast MATa deletion collection |  | http://www-sequence.stanford.edu/group/yeast_deletion_project/deletions3.html |
| strain, strain background (*S. cerevisiae*) | ydr322c-aΔ | YSC1053 Yeast MATa deletion collection |  | http://www-sequence.stanford.edu/group/yeast_deletion_project/deletions3.html |
| strain, strain background (*S. cerevisiae*) | ydr322wΔ | YSC1053 Yeast MATa deletion collection |  | http://www-sequence.stanford.edu/group/yeast_deletion_project/deletions3.html |
| strain, strain background (*S. cerevisiae*) | ydr323cΔ | YSC1053 Yeast MATa deletion collection |  | http://www-sequence.stanford.edu/group/yeast_deletion_project/deletions3.html |
| strain, strain background (*S. cerevisiae*) | ydr329cΔ | YSC1053 Yeast MATa deletion collection |  | http://www-sequence.stanford.edu/group/yeast_deletion_project/deletions3.html |
| strain, strain background (*S. cerevisiae*) | ydr335wΔ | YSC1053 Yeast MATa deletion collection |  | http://www-sequence.stanford.edu/group/yeast_deletion_project/deletions3.html |
| strain, strain background (*S. cerevisiae*) | ydr337wΔ | YSC1053 Yeast MATa deletion collection |  | http://www-sequence.stanford.edu/group/yeast_deletion_project/deletions3.html |
| strain, strain background (*S. cerevisiae*) | ydr347wΔ | YSC1053 Yeast MATa deletion collection |  | http://www-sequence.stanford.edu/group/yeast_deletion_project/deletions3.html |
| strain, strain background (*S. cerevisiae*) | ydr350cΔ | YSC1053 Yeast MATa deletion collection |  | http://www-sequence.stanford.edu/group/yeast_deletion_project/deletions3.html |
| strain, strain background (*S. cerevisiae*) | ydr354wΔ | YSC1053 Yeast MATa deletion collection |  | http://www-sequence.stanford.edu/group/yeast_deletion_project/deletions3.html |
| strain, strain background (*S. cerevisiae*) | ydr359cΔ | YSC1053 Yeast MATa deletion collection |  | http://www-sequence.stanford.edu/group/yeast_deletion_project/deletions3.html |
| strain, strain background (*S. cerevisiae*) | ydr360wΔ | YSC1053 Yeast MATa deletion collection |  | http://www-sequence.stanford.edu/group/yeast_deletion_project/deletions3.html |
| strain, strain background (*S. cerevisiae*) | ydr363wΔ | YSC1053 Yeast MATa deletion collection |  | http://www-sequence.stanford.edu/group/yeast_deletion_project/deletions3.html |
| strain, strain background (*S. cerevisiae*) | ydr363w-aΔ | YSC1053 Yeast MATa deletion collection |  | http://www-sequence.stanford.edu/group/yeast_deletion_project/deletions3.html |
| strain, strain background (*S. cerevisiae*) | ydr364cΔ | YSC1053 Yeast MATa deletion collection |  | http://www-sequence.stanford.edu/group/yeast_deletion_project/deletions3.html |
| strain, strain background (*S. cerevisiae*) | ydr369cΔ | YSC1053 Yeast MATa deletion collection |  | http://www-sequence.stanford.edu/group/yeast_deletion_project/deletions3.html |
| strain, strain background (*S. cerevisiae*) | ydr371wΔ | YSC1053 Yeast MATa deletion collection |  | http://www-sequence.stanford.edu/group/yeast_deletion_project/deletions3.html |
| strain, strain background (*S. cerevisiae*) | ydr375cΔ | YSC1053 Yeast MATa deletion collection |  | http://www-sequence.stanford.edu/group/yeast_deletion_project/deletions3.html |
| strain, strain background (*S. cerevisiae*) | ydr377wΔ | YSC1053 Yeast MATa deletion collection |  | http://www-sequence.stanford.edu/group/yeast_deletion_project/deletions3.html |
| strain, strain background (*S. cerevisiae*) | ydr378cΔ | YSC1053 Yeast MATa deletion collection |  | http://www-sequence.stanford.edu/group/yeast_deletion_project/deletions3.html |
| strain, strain background (*S. cerevisiae*) | ydr382wΔ | YSC1053 Yeast MATa deletion collection |  | http://www-sequence.stanford.edu/group/yeast_deletion_project/deletions3.html |
| strain, strain background (*S. cerevisiae*) | ydr385wΔ | YSC1053 Yeast MATa deletion collection |  | http://www-sequence.stanford.edu/group/yeast_deletion_project/deletions3.html |
| strain, strain background (*S. cerevisiae*) | ydr388wΔ | YSC1053 Yeast MATa deletion collection |  | http://www-sequence.stanford.edu/group/yeast_deletion_project/deletions3.html |
| strain, strain background (*S. cerevisiae*) | ydr389wΔ | YSC1053 Yeast MATa deletion collection |  | http://www-sequence.stanford.edu/group/yeast_deletion_project/deletions3.html |
| strain, strain background (*S. cerevisiae*) | ydr392wΔ | YSC1053 Yeast MATa deletion collection |  | http://www-sequence.stanford.edu/group/yeast_deletion_project/deletions3.html |
| strain, strain background (*S. cerevisiae*) | ydr393wΔ | YSC1053 Yeast MATa deletion collection |  | http://www-sequence.stanford.edu/group/yeast_deletion_project/deletions3.html |
| strain, strain background (*S. cerevisiae*) | ydr405wΔ | YSC1053 Yeast MATa deletion collection |  | http://www-sequence.stanford.edu/group/yeast_deletion_project/deletions3.html |
| strain, strain background (*S. cerevisiae*) | ydr409wΔ | YSC1053 Yeast MATa deletion collection |  | http://www-sequence.stanford.edu/group/yeast_deletion_project/deletions3.html |
| strain, strain background (*S. cerevisiae*) | ydr411cΔ | YSC1053 Yeast MATa deletion collection |  | http://www-sequence.stanford.edu/group/yeast_deletion_project/deletions3.html |
| strain, strain background (*S. cerevisiae*) | ydr418wΔ | YSC1053 Yeast MATa deletion collection |  | http://www-sequence.stanford.edu/group/yeast_deletion_project/deletions3.html |
| strain, strain background (*S. cerevisiae*) | ydr420wΔ | YSC1053 Yeast MATa deletion collection |  | http://www-sequence.stanford.edu/group/yeast_deletion_project/deletions3.html |
| strain, strain background (*S. cerevisiae*) | ydr423cΔ | YSC1053 Yeast MATa deletion collection |  | http://www-sequence.stanford.edu/group/yeast_deletion_project/deletions3.html |
| strain, strain background (*S. cerevisiae*) | ydr425wΔ | YSC1053 Yeast MATa deletion collection |  | http://www-sequence.stanford.edu/group/yeast_deletion_project/deletions3.html |
| strain, strain background (*S. cerevisiae*) | ydr426cΔ | YSC1053 Yeast MATa deletion collection |  | http://www-sequence.stanford.edu/group/yeast_deletion_project/deletions3.html |
| strain, strain background (*S. cerevisiae*) | ydr432wΔ | YSC1053 Yeast MATa deletion collection |  | http://www-sequence.stanford.edu/group/yeast_deletion_project/deletions3.html |
| strain, strain background (*S. cerevisiae*) | ydr439wΔ | YSC1053 Yeast MATa deletion collection |  | http://www-sequence.stanford.edu/group/yeast_deletion_project/deletions3.html |
| strain, strain background (*S. cerevisiae*) | ydr441cΔ | YSC1053 Yeast MATa deletion collection |  | http://www-sequence.stanford.edu/group/yeast_deletion_project/deletions3.html |
| strain, strain background (*S. cerevisiae*) | ydr442wΔ | YSC1053 Yeast MATa deletion collection |  | http://www-sequence.stanford.edu/group/yeast_deletion_project/deletions3.html |
| strain, strain background (*S. cerevisiae*) | ydr443cΔ | YSC1053 Yeast MATa deletion collection |  | http://www-sequence.stanford.edu/group/yeast_deletion_project/deletions3.html |
| strain, strain background (*S. cerevisiae*) | ydr445cΔ | YSC1053 Yeast MATa deletion collection |  | http://www-sequence.stanford.edu/group/yeast_deletion_project/deletions3.html |
| strain, strain background (*S. cerevisiae*) | ydr447cΔ | YSC1053 Yeast MATa deletion collection |  | http://www-sequence.stanford.edu/group/yeast_deletion_project/deletions3.html |
| strain, strain background (*S. cerevisiae*) | ydr448wΔ | YSC1053 Yeast MATa deletion collection |  | http://www-sequence.stanford.edu/group/yeast_deletion_project/deletions3.html |
| strain, strain background (*S. cerevisiae*) | ydr450wΔ | YSC1053 Yeast MATa deletion collection |  | http://www-sequence.stanford.edu/group/yeast_deletion_project/deletions3.html |
| strain, strain background (*S. cerevisiae*) | ydr455cΔ | YSC1053 Yeast MATa deletion collection |  | http://www-sequence.stanford.edu/group/yeast_deletion_project/deletions3.html |
| strain, strain background (*S. cerevisiae*) | ydr456wΔ | YSC1053 Yeast MATa deletion collection |  | http://www-sequence.stanford.edu/group/yeast_deletion_project/deletions3.html |
| strain, strain background (*S. cerevisiae*) | ydr457wΔ | YSC1053 Yeast MATa deletion collection |  | http://www-sequence.stanford.edu/group/yeast_deletion_project/deletions3.html |
| strain, strain background (*S. cerevisiae*) | ydr462wΔ | YSC1053 Yeast MATa deletion collection |  | http://www-sequence.stanford.edu/group/yeast_deletion_project/deletions3.html |
| strain, strain background (*S. cerevisiae*) | ydr463wΔ | YSC1053 Yeast MATa deletion collection |  | http://www-sequence.stanford.edu/group/yeast_deletion_project/deletions3.html |
| strain, strain background (*S. cerevisiae*) | ydr470cΔ | YSC1053 Yeast MATa deletion collection |  | http://www-sequence.stanford.edu/group/yeast_deletion_project/deletions3.html |
| strain, strain background (*S. cerevisiae*) | ydr471wΔ | YSC1053 Yeast MATa deletion collection |  | http://www-sequence.stanford.edu/group/yeast_deletion_project/deletions3.html |
| strain, strain background (*S. cerevisiae*) | ydr477wΔ | YSC1053 Yeast MATa deletion collection |  | http://www-sequence.stanford.edu/group/yeast_deletion_project/deletions3.html |
| strain, strain background (*S. cerevisiae*) | ydr480wΔ | YSC1053 Yeast MATa deletion collection |  | http://www-sequence.stanford.edu/group/yeast_deletion_project/deletions3.html |
| strain, strain background (*S. cerevisiae*) | ydr484wΔ | YSC1053 Yeast MATa deletion collection |  | http://www-sequence.stanford.edu/group/yeast_deletion_project/deletions3.html |
| strain, strain background (*S. cerevisiae*) | ydr485cΔ | YSC1053 Yeast MATa deletion collection |  | http://www-sequence.stanford.edu/group/yeast_deletion_project/deletions3.html |
| strain, strain background (*S. cerevisiae*) | ydr495cΔ | YSC1053 Yeast MATa deletion collection |  | http://www-sequence.stanford.edu/group/yeast_deletion_project/deletions3.html |
| strain, strain background (*S. cerevisiae*) | ydr496cΔ | YSC1053 Yeast MATa deletion collection |  | http://www-sequence.stanford.edu/group/yeast_deletion_project/deletions3.html |
| strain, strain background (*S. cerevisiae*) | ydr500cΔ | YSC1053 Yeast MATa deletion collection |  | http://www-sequence.stanford.edu/group/yeast_deletion_project/deletions3.html |
| strain, strain background (*S. cerevisiae*) | ydr508cΔ | YSC1053 Yeast MATa deletion collection |  | http://www-sequence.stanford.edu/group/yeast_deletion_project/deletions3.html |
| strain, strain background (*S. cerevisiae*) | ydr509wΔ | YSC1053 Yeast MATa deletion collection |  | http://www-sequence.stanford.edu/group/yeast_deletion_project/deletions3.html |
| strain, strain background (*S. cerevisiae*) | ydr512cΔ | YSC1053 Yeast MATa deletion collection |  | http://www-sequence.stanford.edu/group/yeast_deletion_project/deletions3.html |
| strain, strain background (*S. cerevisiae*) | ydr518wΔ | YSC1053 Yeast MATa deletion collection |  | http://www-sequence.stanford.edu/group/yeast_deletion_project/deletions3.html |
| strain, strain background (*S. cerevisiae*) | ydr519wΔ | YSC1053 Yeast MATa deletion collection |  | http://www-sequence.stanford.edu/group/yeast_deletion_project/deletions3.html |
| strain, strain background (*S. cerevisiae*) | ydr520cΔ | YSC1053 Yeast MATa deletion collection |  | http://www-sequence.stanford.edu/group/yeast_deletion_project/deletions3.html |
| strain, strain background (*S. cerevisiae*) | ydr528wΔ | YSC1053 Yeast MATa deletion collection |  | http://www-sequence.stanford.edu/group/yeast_deletion_project/deletions3.html |
| strain, strain background (*S. cerevisiae*) | ydr529cΔ | YSC1053 Yeast MATa deletion collection |  | http://www-sequence.stanford.edu/group/yeast_deletion_project/deletions3.html |
| strain, strain background (*S. cerevisiae*) | ydr530cΔ | YSC1053 Yeast MATa deletion collection |  | http://www-sequence.stanford.edu/group/yeast_deletion_project/deletions3.html |
| strain, strain background (*S. cerevisiae*) | ydr532cΔ | YSC1053 Yeast MATa deletion collection |  | http://www-sequence.stanford.edu/group/yeast_deletion_project/deletions3.html |
| strain, strain background (*S. cerevisiae*) | ydr534cΔ | YSC1053 Yeast MATa deletion collection |  | http://www-sequence.stanford.edu/group/yeast_deletion_project/deletions3.html |
| strain, strain background (*S. cerevisiae*) | yel009cΔ | YSC1053 Yeast MATa deletion collection |  | http://www-sequence.stanford.edu/group/yeast_deletion_project/deletions3.html |
| strain, strain background (*S. cerevisiae*) | yel013wΔ | YSC1053 Yeast MATa deletion collection |  | http://www-sequence.stanford.edu/group/yeast_deletion_project/deletions3.html |
| strain, strain background (*S. cerevisiae*) | yel027wΔ | YSC1053 Yeast MATa deletion collection |  | http://www-sequence.stanford.edu/group/yeast_deletion_project/deletions3.html |
| strain, strain background (*S. cerevisiae*) | yel029cΔ | YSC1053 Yeast MATa deletion collection |  | http://www-sequence.stanford.edu/group/yeast_deletion_project/deletions3.html |
| strain, strain background (*S. cerevisiae*) | yel036cΔ | YSC1053 Yeast MATa deletion collection |  | http://www-sequence.stanford.edu/group/yeast_deletion_project/deletions3.html |
| strain, strain background (*S. cerevisiae*) | yel044wΔ | YSC1053 Yeast MATa deletion collection |  | http://www-sequence.stanford.edu/group/yeast_deletion_project/deletions3.html |
| strain, strain background (*S. cerevisiae*) | yel045cΔ | YSC1053 Yeast MATa deletion collection |  | http://www-sequence.stanford.edu/group/yeast_deletion_project/deletions3.html |
| strain, strain background (*S. cerevisiae*) | yel046cΔ | YSC1053 Yeast MATa deletion collection |  | http://www-sequence.stanford.edu/group/yeast_deletion_project/deletions3.html |
| strain, strain background (*S. cerevisiae*) | yel061cΔ | YSC1053 Yeast MATa deletion collection |  | http://www-sequence.stanford.edu/group/yeast_deletion_project/deletions3.html |
| strain, strain background (*S. cerevisiae*) | yer005wΔ | YSC1053 Yeast MATa deletion collection |  | http://www-sequence.stanford.edu/group/yeast_deletion_project/deletions3.html |
| strain, strain background (*S. cerevisiae*) | yer007c-aΔ | YSC1053 Yeast MATa deletion collection |  | http://www-sequence.stanford.edu/group/yeast_deletion_project/deletions3.html |
| strain, strain background (*S. cerevisiae*) | yer016wΔ | YSC1053 Yeast MATa deletion collection |  | http://www-sequence.stanford.edu/group/yeast_deletion_project/deletions3.html |
| strain, strain background (*S. cerevisiae*) | yer032wΔ | YSC1053 Yeast MATa deletion collection |  | http://www-sequence.stanford.edu/group/yeast_deletion_project/deletions3.html |
| strain, strain background (*S. cerevisiae*) | yer034wΔ | YSC1053 Yeast MATa deletion collection |  | http://www-sequence.stanford.edu/group/yeast_deletion_project/deletions3.html |
| strain, strain background (*S. cerevisiae*) | yer040wΔ | YSC1053 Yeast MATa deletion collection |  | http://www-sequence.stanford.edu/group/yeast_deletion_project/deletions3.html |
| strain, strain background (*S. cerevisiae*) | yer044cΔ | YSC1053 Yeast MATa deletion collection |  | http://www-sequence.stanford.edu/group/yeast_deletion_project/deletions3.html |
| strain, strain background (*S. cerevisiae*) | yer047cΔ | YSC1053 Yeast MATa deletion collection |  | http://www-sequence.stanford.edu/group/yeast_deletion_project/deletions3.html |
| strain, strain background (*S. cerevisiae*) | yer050cΔ | YSC1053 Yeast MATa deletion collection |  | http://www-sequence.stanford.edu/group/yeast_deletion_project/deletions3.html |
| strain, strain background (*S. cerevisiae*) | yer052cΔ | YSC1053 Yeast MATa deletion collection |  | http://www-sequence.stanford.edu/group/yeast_deletion_project/deletions3.html |
| strain, strain background (*S. cerevisiae*) | yer056c-aΔ | YSC1053 Yeast MATa deletion collection |  | http://www-sequence.stanford.edu/group/yeast_deletion_project/deletions3.html |
| strain, strain background (*S. cerevisiae*) | yer058wΔ | YSC1053 Yeast MATa deletion collection |  | http://www-sequence.stanford.edu/group/yeast_deletion_project/deletions3.html |
| strain, strain background (*S. cerevisiae*) | yer060w-aΔ | YSC1053 Yeast MATa deletion collection |  | http://www-sequence.stanford.edu/group/yeast_deletion_project/deletions3.html |
| strain, strain background (*S. cerevisiae*) | yer061cΔ | YSC1053 Yeast MATa deletion collection |  | http://www-sequence.stanford.edu/group/yeast_deletion_project/deletions3.html |
| strain, strain background (*S. cerevisiae*) | yer062cΔ | YSC1053 Yeast MATa deletion collection |  | http://www-sequence.stanford.edu/group/yeast_deletion_project/deletions3.html |
| strain, strain background (*S. cerevisiae*) | yer063wΔ | YSC1053 Yeast MATa deletion collection |  | http://www-sequence.stanford.edu/group/yeast_deletion_project/deletions3.html |
| strain, strain background (*S. cerevisiae*) | yer068wΔ | YSC1053 Yeast MATa deletion collection |  | http://www-sequence.stanford.edu/group/yeast_deletion_project/deletions3.html |
| strain, strain background (*S. cerevisiae*) | yer070wΔ | YSC1053 Yeast MATa deletion collection |  | http://www-sequence.stanford.edu/group/yeast_deletion_project/deletions3.html |
| strain, strain background (*S. cerevisiae*) | yer072wΔ | YSC1053 Yeast MATa deletion collection |  | http://www-sequence.stanford.edu/group/yeast_deletion_project/deletions3.html |
| strain, strain background (*S. cerevisiae*) | yer074wΔ | YSC1053 Yeast MATa deletion collection |  | http://www-sequence.stanford.edu/group/yeast_deletion_project/deletions3.html |
| strain, strain background (*S. cerevisiae*) | yer077cΔ | YSC1053 Yeast MATa deletion collection |  | http://www-sequence.stanford.edu/group/yeast_deletion_project/deletions3.html |
| strain, strain background (*S. cerevisiae*) | yer078cΔ | YSC1053 Yeast MATa deletion collection |  | http://www-sequence.stanford.edu/group/yeast_deletion_project/deletions3.html |
| strain, strain background (*S. cerevisiae*) | yer083cΔ | YSC1053 Yeast MATa deletion collection |  | http://www-sequence.stanford.edu/group/yeast_deletion_project/deletions3.html |
| strain, strain background (*S. cerevisiae*) | yer086wΔ | YSC1053 Yeast MATa deletion collection |  | http://www-sequence.stanford.edu/group/yeast_deletion_project/deletions3.html |
| strain, strain background (*S. cerevisiae*) | yer087wΔ | YSC1053 Yeast MATa deletion collection |  | http://www-sequence.stanford.edu/group/yeast_deletion_project/deletions3.html |
| strain, strain background (*S. cerevisiae*) | yer088cΔ | YSC1053 Yeast MATa deletion collection |  | http://www-sequence.stanford.edu/group/yeast_deletion_project/deletions3.html |
| strain, strain background (*S. cerevisiae*) | yer092wΔ | YSC1053 Yeast MATa deletion collection |  | http://www-sequence.stanford.edu/group/yeast_deletion_project/deletions3.html |
| strain, strain background (*S. cerevisiae*) | yer095wΔ | YSC1053 Yeast MATa deletion collection |  | http://www-sequence.stanford.edu/group/yeast_deletion_project/deletions3.html |
| strain, strain background (*S. cerevisiae*) | yer101cΔ | YSC1053 Yeast MATa deletion collection |  | http://www-sequence.stanford.edu/group/yeast_deletion_project/deletions3.html |
| strain, strain background (*S. cerevisiae*) | yer103wΔ | YSC1053 Yeast MATa deletion collection |  | http://www-sequence.stanford.edu/group/yeast_deletion_project/deletions3.html |
| strain, strain background (*S. cerevisiae*) | yer106wΔ | YSC1053 Yeast MATa deletion collection |  | http://www-sequence.stanford.edu/group/yeast_deletion_project/deletions3.html |
| strain, strain background (*S. cerevisiae*) | yer110cΔ | YSC1053 Yeast MATa deletion collection |  | http://www-sequence.stanford.edu/group/yeast_deletion_project/deletions3.html |
| strain, strain background (*S. cerevisiae*) | yer111cΔ | YSC1053 Yeast MATa deletion collection |  | http://www-sequence.stanford.edu/group/yeast_deletion_project/deletions3.html |
| strain, strain background (*S. cerevisiae*) | yer116cΔ | YSC1053 Yeast MATa deletion collection |  | http://www-sequence.stanford.edu/group/yeast_deletion_project/deletions3.html |
| strain, strain background (*S. cerevisiae*) | yer117wΔ | YSC1053 Yeast MATa deletion collection |  | http://www-sequence.stanford.edu/group/yeast_deletion_project/deletions3.html |
| strain, strain background (*S. cerevisiae*) | yer120wΔ | YSC1053 Yeast MATa deletion collection |  | http://www-sequence.stanford.edu/group/yeast_deletion_project/deletions3.html |
| strain, strain background (*S. cerevisiae*) | yer122cΔ | YSC1053 Yeast MATa deletion collection |  | http://www-sequence.stanford.edu/group/yeast_deletion_project/deletions3.html |
| strain, strain background (*S. cerevisiae*) | yer128wΔ | YSC1053 Yeast MATa deletion collection |  | http://www-sequence.stanford.edu/group/yeast_deletion_project/deletions3.html |
| strain, strain background (*S. cerevisiae*) | yer129wΔ | YSC1053 Yeast MATa deletion collection |  | http://www-sequence.stanford.edu/group/yeast_deletion_project/deletions3.html |
| strain, strain background (*S. cerevisiae*) | yer131wΔ | YSC1053 Yeast MATa deletion collection |  | http://www-sequence.stanford.edu/group/yeast_deletion_project/deletions3.html |
| strain, strain background (*S. cerevisiae*) | yer136wΔ | YSC1053 Yeast MATa deletion collection |  | http://www-sequence.stanford.edu/group/yeast_deletion_project/deletions3.html |
| strain, strain background (*S. cerevisiae*) | yer139cΔ | YSC1053 Yeast MATa deletion collection |  | http://www-sequence.stanford.edu/group/yeast_deletion_project/deletions3.html |
| strain, strain background (*S. cerevisiae*) | yer141wΔ | YSC1053 Yeast MATa deletion collection |  | http://www-sequence.stanford.edu/group/yeast_deletion_project/deletions3.html |
| strain, strain background (*S. cerevisiae*) | yer145cΔ | YSC1053 Yeast MATa deletion collection |  | http://www-sequence.stanford.edu/group/yeast_deletion_project/deletions3.html |
| strain, strain background (*S. cerevisiae*) | yer151cΔ | YSC1053 Yeast MATa deletion collection |  | http://www-sequence.stanford.edu/group/yeast_deletion_project/deletions3.html |
| strain, strain background (*S. cerevisiae*) | yer153cΔ | YSC1053 Yeast MATa deletion collection |  | http://www-sequence.stanford.edu/group/yeast_deletion_project/deletions3.html |
| strain, strain background (*S. cerevisiae*) | yer154wΔ | YSC1053 Yeast MATa deletion collection |  | http://www-sequence.stanford.edu/group/yeast_deletion_project/deletions3.html |
| strain, strain background (*S. cerevisiae*) | yer155cΔ | YSC1053 Yeast MATa deletion collection |  | http://www-sequence.stanford.edu/group/yeast_deletion_project/deletions3.html |
| strain, strain background (*S. cerevisiae*) | yer161cΔ | YSC1053 Yeast MATa deletion collection |  | http://www-sequence.stanford.edu/group/yeast_deletion_project/deletions3.html |
| strain, strain background (*S. cerevisiae*) | yer162cΔ | YSC1053 Yeast MATa deletion collection |  | http://www-sequence.stanford.edu/group/yeast_deletion_project/deletions3.html |
| strain, strain background (*S. cerevisiae*) | yer164wΔ | YSC1053 Yeast MATa deletion collection |  | http://www-sequence.stanford.edu/group/yeast_deletion_project/deletions3.html |
| strain, strain background (*S. cerevisiae*) | yer169wΔ | YSC1053 Yeast MATa deletion collection |  | http://www-sequence.stanford.edu/group/yeast_deletion_project/deletions3.html |
| strain, strain background (*S. cerevisiae*) | yer174cΔ | YSC1053 Yeast MATa deletion collection |  | http://www-sequence.stanford.edu/group/yeast_deletion_project/deletions3.html |
| strain, strain background (*S. cerevisiae*) | yer177wΔ | YSC1053 Yeast MATa deletion collection |  | http://www-sequence.stanford.edu/group/yeast_deletion_project/deletions3.html |
| strain, strain background (*S. cerevisiae*) | yer178wΔ | YSC1053 Yeast MATa deletion collection |  | http://www-sequence.stanford.edu/group/yeast_deletion_project/deletions3.html |
| strain, strain background (*S. cerevisiae*) | yer180c-aΔ | YSC1053 Yeast MATa deletion collection |  | http://www-sequence.stanford.edu/group/yeast_deletion_project/deletions3.html |
| strain, strain background (*S. cerevisiae*) | yfl001wΔ | YSC1053 Yeast MATa deletion collection |  | http://www-sequence.stanford.edu/group/yeast_deletion_project/deletions3.html |
| strain, strain background (*S. cerevisiae*) | yfl007wΔ | YSC1053 Yeast MATa deletion collection |  | http://www-sequence.stanford.edu/group/yeast_deletion_project/deletions3.html |
| strain, strain background (*S. cerevisiae*) | yfl013cΔ | YSC1053 Yeast MATa deletion collection |  | http://www-sequence.stanford.edu/group/yeast_deletion_project/deletions3.html |
| strain, strain background (*S. cerevisiae*) | yfl013w-aΔ | YSC1053 Yeast MATa deletion collection |  | http://www-sequence.stanford.edu/group/yeast_deletion_project/deletions3.html |
| strain, strain background (*S. cerevisiae*) | yfl016cΔ | YSC1053 Yeast MATa deletion collection |  | http://www-sequence.stanford.edu/group/yeast_deletion_project/deletions3.html |
| strain, strain background (*S. cerevisiae*) | yfl023wΔ | YSC1053 Yeast MATa deletion collection |  | http://www-sequence.stanford.edu/group/yeast_deletion_project/deletions3.html |
| strain, strain background (*S. cerevisiae*) | yfl033cΔ | YSC1053 Yeast MATa deletion collection |  | http://www-sequence.stanford.edu/group/yeast_deletion_project/deletions3.html |
| strain, strain background (*S. cerevisiae*) | yfl036wΔ | YSC1053 Yeast MATa deletion collection |  | http://www-sequence.stanford.edu/group/yeast_deletion_project/deletions3.html |
| strain, strain background (*S. cerevisiae*) | yfl050cΔ | YSC1053 Yeast MATa deletion collection |  | http://www-sequence.stanford.edu/group/yeast_deletion_project/deletions3.html |
| strain, strain background (*S. cerevisiae*) | yfr001wΔ | YSC1053 Yeast MATa deletion collection |  | http://www-sequence.stanford.edu/group/yeast_deletion_project/deletions3.html |
| strain, strain background (*S. cerevisiae*) | yfr008wΔ | YSC1053 Yeast MATa deletion collection |  | http://www-sequence.stanford.edu/group/yeast_deletion_project/deletions3.html |
| strain, strain background (*S. cerevisiae*) | yfr009wΔ | YSC1053 Yeast MATa deletion collection |  | http://www-sequence.stanford.edu/group/yeast_deletion_project/deletions3.html |
| strain, strain background (*S. cerevisiae*) | yfr010wΔ | YSC1053 Yeast MATa deletion collection |  | http://www-sequence.stanford.edu/group/yeast_deletion_project/deletions3.html |
| strain, strain background (*S. cerevisiae*) | yfr011cΔ | YSC1053 Yeast MATa deletion collection |  | http://www-sequence.stanford.edu/group/yeast_deletion_project/deletions3.html |
| strain, strain background (*S. cerevisiae*) | yfr032c-aΔ | YSC1053 Yeast MATa deletion collection |  | http://www-sequence.stanford.edu/group/yeast_deletion_project/deletions3.html |
| strain, strain background (*S. cerevisiae*) | yfr032c-bΔ | YSC1053 Yeast MATa deletion collection |  | http://www-sequence.stanford.edu/group/yeast_deletion_project/deletions3.html |
| strain, strain background (*S. cerevisiae*) | yfr040wΔ | YSC1053 Yeast MATa deletion collection |  | http://www-sequence.stanford.edu/group/yeast_deletion_project/deletions3.html |
| strain, strain background (*S. cerevisiae*) | ygl003cΔ | YSC1053 Yeast MATa deletion collection |  | http://www-sequence.stanford.edu/group/yeast_deletion_project/deletions3.html |
| strain, strain background (*S. cerevisiae*) | ygl004cΔ | YSC1053 Yeast MATa deletion collection |  | http://www-sequence.stanford.edu/group/yeast_deletion_project/deletions3.html |
| strain, strain background (*S. cerevisiae*) | ygl006wΔ | YSC1053 Yeast MATa deletion collection |  | http://www-sequence.stanford.edu/group/yeast_deletion_project/deletions3.html |
| strain, strain background (*S. cerevisiae*) | ygl007c-aΔ | YSC1053 Yeast MATa deletion collection |  | http://www-sequence.stanford.edu/group/yeast_deletion_project/deletions3.html |
| strain, strain background (*S. cerevisiae*) | ygl007wΔ | YSC1053 Yeast MATa deletion collection |  | http://www-sequence.stanford.edu/group/yeast_deletion_project/deletions3.html |
| strain, strain background (*S. cerevisiae*) | ygl012wΔ | YSC1053 Yeast MATa deletion collection |  | http://www-sequence.stanford.edu/group/yeast_deletion_project/deletions3.html |
| strain, strain background (*S. cerevisiae*) | ygl016wΔ | YSC1053 Yeast MATa deletion collection |  | http://www-sequence.stanford.edu/group/yeast_deletion_project/deletions3.html |
| strain, strain background (*S. cerevisiae*) | ygl017wΔ | YSC1053 Yeast MATa deletion collection |  | http://www-sequence.stanford.edu/group/yeast_deletion_project/deletions3.html |
| strain, strain background (*S. cerevisiae*) | ygl019wΔ | YSC1053 Yeast MATa deletion collection |  | http://www-sequence.stanford.edu/group/yeast_deletion_project/deletions3.html |
| strain, strain background (*S. cerevisiae*) | ygl020cΔ | YSC1053 Yeast MATa deletion collection |  | http://www-sequence.stanford.edu/group/yeast_deletion_project/deletions3.html |
| strain, strain background (*S. cerevisiae*) | ygl023cΔ | YSC1053 Yeast MATa deletion collection |  | http://www-sequence.stanford.edu/group/yeast_deletion_project/deletions3.html |
| strain, strain background (*S. cerevisiae*) | ygl024wΔ | YSC1053 Yeast MATa deletion collection |  | http://www-sequence.stanford.edu/group/yeast_deletion_project/deletions3.html |
| strain, strain background (*S. cerevisiae*) | ygl025cΔ | YSC1053 Yeast MATa deletion collection |  | http://www-sequence.stanford.edu/group/yeast_deletion_project/deletions3.html |
| strain, strain background (*S. cerevisiae*) | ygl027cΔ | YSC1053 Yeast MATa deletion collection |  | http://www-sequence.stanford.edu/group/yeast_deletion_project/deletions3.html |
| strain, strain background (*S. cerevisiae*) | ygl029wΔ | YSC1053 Yeast MATa deletion collection |  | http://www-sequence.stanford.edu/group/yeast_deletion_project/deletions3.html |
| strain, strain background (*S. cerevisiae*) | ygl031cΔ | YSC1053 Yeast MATa deletion collection |  | http://www-sequence.stanford.edu/group/yeast_deletion_project/deletions3.html |
| strain, strain background (*S. cerevisiae*) | ygl035cΔ | YSC1053 Yeast MATa deletion collection |  | http://www-sequence.stanford.edu/group/yeast_deletion_project/deletions3.html |
| strain, strain background (*S. cerevisiae*) | ygl037cΔ | YSC1053 Yeast MATa deletion collection |  | http://www-sequence.stanford.edu/group/yeast_deletion_project/deletions3.html |
| strain, strain background (*S. cerevisiae*) | ygl038cΔ | YSC1053 Yeast MATa deletion collection |  | http://www-sequence.stanford.edu/group/yeast_deletion_project/deletions3.html |
| strain, strain background (*S. cerevisiae*) | ygl042cΔ | YSC1053 Yeast MATa deletion collection |  | http://www-sequence.stanford.edu/group/yeast_deletion_project/deletions3.html |
| strain, strain background (*S. cerevisiae*) | ygl043wΔ | YSC1053 Yeast MATa deletion collection |  | http://www-sequence.stanford.edu/group/yeast_deletion_project/deletions3.html |
| strain, strain background (*S. cerevisiae*) | ygl045wΔ | YSC1053 Yeast MATa deletion collection |  | http://www-sequence.stanford.edu/group/yeast_deletion_project/deletions3.html |
| strain, strain background (*S. cerevisiae*) | ygl046wΔ | YSC1053 Yeast MATa deletion collection |  | http://www-sequence.stanford.edu/group/yeast_deletion_project/deletions3.html |
| strain, strain background (*S. cerevisiae*) | ygl049cΔ | YSC1053 Yeast MATa deletion collection |  | http://www-sequence.stanford.edu/group/yeast_deletion_project/deletions3.html |
| strain, strain background (*S. cerevisiae*) | ygl050wΔ | YSC1053 Yeast MATa deletion collection |  | http://www-sequence.stanford.edu/group/yeast_deletion_project/deletions3.html |
| strain, strain background (*S. cerevisiae*) | ygl053wΔ | YSC1053 Yeast MATa deletion collection |  | http://www-sequence.stanford.edu/group/yeast_deletion_project/deletions3.html |
| strain, strain background (*S. cerevisiae*) | ygl058wΔ | YSC1053 Yeast MATa deletion collection |  | http://www-sequence.stanford.edu/group/yeast_deletion_project/deletions3.html |
| strain, strain background (*S. cerevisiae*) | ygl064cΔ | YSC1053 Yeast MATa deletion collection |  | http://www-sequence.stanford.edu/group/yeast_deletion_project/deletions3.html |
| strain, strain background (*S. cerevisiae*) | ygl066wΔ | YSC1053 Yeast MATa deletion collection |  | http://www-sequence.stanford.edu/group/yeast_deletion_project/deletions3.html |
| strain, strain background (*S. cerevisiae*) | ygl070cΔ | YSC1053 Yeast MATa deletion collection |  | http://www-sequence.stanford.edu/group/yeast_deletion_project/deletions3.html |
| strain, strain background (*S. cerevisiae*) | ygl072cΔ | YSC1053 Yeast MATa deletion collection |  | http://www-sequence.stanford.edu/group/yeast_deletion_project/deletions3.html |
| strain, strain background (*S. cerevisiae*) | ygl076cΔ | YSC1053 Yeast MATa deletion collection |  | http://www-sequence.stanford.edu/group/yeast_deletion_project/deletions3.html |
| strain, strain background (*S. cerevisiae*) | ygl078cΔ | YSC1053 Yeast MATa deletion collection |  | http://www-sequence.stanford.edu/group/yeast_deletion_project/deletions3.html |
| strain, strain background (*S. cerevisiae*) | ygl081wΔ | YSC1053 Yeast MATa deletion collection |  | http://www-sequence.stanford.edu/group/yeast_deletion_project/deletions3.html |
| strain, strain background (*S. cerevisiae*) | ygl082wΔ | YSC1053 Yeast MATa deletion collection |  | http://www-sequence.stanford.edu/group/yeast_deletion_project/deletions3.html |
| strain, strain background (*S. cerevisiae*) | ygl084cΔ | YSC1053 Yeast MATa deletion collection |  | http://www-sequence.stanford.edu/group/yeast_deletion_project/deletions3.html |
| strain, strain background (*S. cerevisiae*) | ygl087cΔ | YSC1053 Yeast MATa deletion collection |  | http://www-sequence.stanford.edu/group/yeast_deletion_project/deletions3.html |
| strain, strain background (*S. cerevisiae*) | ygl094cΔ | YSC1053 Yeast MATa deletion collection |  | http://www-sequence.stanford.edu/group/yeast_deletion_project/deletions3.html |
| strain, strain background (*S. cerevisiae*) | ygl095cΔ | YSC1053 Yeast MATa deletion collection |  | http://www-sequence.stanford.edu/group/yeast_deletion_project/deletions3.html |
| strain, strain background (*S. cerevisiae*) | ygl100wΔ | YSC1053 Yeast MATa deletion collection |  | http://www-sequence.stanford.edu/group/yeast_deletion_project/deletions3.html |
| strain, strain background (*S. cerevisiae*) | ygl105wΔ | YSC1053 Yeast MATa deletion collection |  | http://www-sequence.stanford.edu/group/yeast_deletion_project/deletions3.html |
| strain, strain background (*S. cerevisiae*) | ygl107cΔ | YSC1053 Yeast MATa deletion collection |  | http://www-sequence.stanford.edu/group/yeast_deletion_project/deletions3.html |
| strain, strain background (*S. cerevisiae*) | ygl115wΔ | YSC1053 Yeast MATa deletion collection |  | http://www-sequence.stanford.edu/group/yeast_deletion_project/deletions3.html |
| strain, strain background (*S. cerevisiae*) | ygl119wΔ | YSC1053 Yeast MATa deletion collection |  | http://www-sequence.stanford.edu/group/yeast_deletion_project/deletions3.html |
| strain, strain background (*S. cerevisiae*) | ygl124cΔ | YSC1053 Yeast MATa deletion collection |  | http://www-sequence.stanford.edu/group/yeast_deletion_project/deletions3.html |
| strain, strain background (*S. cerevisiae*) | ygl125wΔ | YSC1053 Yeast MATa deletion collection |  | http://www-sequence.stanford.edu/group/yeast_deletion_project/deletions3.html |
| strain, strain background (*S. cerevisiae*) | ygl127cΔ | YSC1053 Yeast MATa deletion collection |  | http://www-sequence.stanford.edu/group/yeast_deletion_project/deletions3.html |
| strain, strain background (*S. cerevisiae*) | ygl129cΔ | YSC1053 Yeast MATa deletion collection |  | http://www-sequence.stanford.edu/group/yeast_deletion_project/deletions3.html |
| strain, strain background (*S. cerevisiae*) | ygl133wΔ | YSC1053 Yeast MATa deletion collection |  | http://www-sequence.stanford.edu/group/yeast_deletion_project/deletions3.html |
| strain, strain background (*S. cerevisiae*) | ygl135wΔ | YSC1053 Yeast MATa deletion collection |  | http://www-sequence.stanford.edu/group/yeast_deletion_project/deletions3.html |
| strain, strain background (*S. cerevisiae*) | ygl139wΔ | YSC1053 Yeast MATa deletion collection |  | http://www-sequence.stanford.edu/group/yeast_deletion_project/deletions3.html |
| strain, strain background (*S. cerevisiae*) | ygl143cΔ | YSC1053 Yeast MATa deletion collection |  | http://www-sequence.stanford.edu/group/yeast_deletion_project/deletions3.html |
| strain, strain background (*S. cerevisiae*) | ygl147cΔ | YSC1053 Yeast MATa deletion collection |  | http://www-sequence.stanford.edu/group/yeast_deletion_project/deletions3.html |
| strain, strain background (*S. cerevisiae*) | ygl148wΔ | YSC1053 Yeast MATa deletion collection |  | http://www-sequence.stanford.edu/group/yeast_deletion_project/deletions3.html |
| strain, strain background (*S. cerevisiae*) | ygl149wΔ | YSC1053 Yeast MATa deletion collection |  | http://www-sequence.stanford.edu/group/yeast_deletion_project/deletions3.html |
| strain, strain background (*S. cerevisiae*) | ygl151wΔ | YSC1053 Yeast MATa deletion collection |  | http://www-sequence.stanford.edu/group/yeast_deletion_project/deletions3.html |
| strain, strain background (*S. cerevisiae*) | ygl152cΔ | YSC1053 Yeast MATa deletion collection |  | http://www-sequence.stanford.edu/group/yeast_deletion_project/deletions3.html |
| strain, strain background (*S. cerevisiae*) | ygl153wΔ | YSC1053 Yeast MATa deletion collection |  | http://www-sequence.stanford.edu/group/yeast_deletion_project/deletions3.html |
| strain, strain background (*S. cerevisiae*) | ygl163cΔ | YSC1053 Yeast MATa deletion collection |  | http://www-sequence.stanford.edu/group/yeast_deletion_project/deletions3.html |
| strain, strain background (*S. cerevisiae*) | ygl167cΔ | YSC1053 Yeast MATa deletion collection |  | http://www-sequence.stanford.edu/group/yeast_deletion_project/deletions3.html |
| strain, strain background (*S. cerevisiae*) | ygl168wΔ | YSC1053 Yeast MATa deletion collection |  | http://www-sequence.stanford.edu/group/yeast_deletion_project/deletions3.html |
| strain, strain background (*S. cerevisiae*) | ygl173cΔ | YSC1053 Yeast MATa deletion collection |  | http://www-sequence.stanford.edu/group/yeast_deletion_project/deletions3.html |
| strain, strain background (*S. cerevisiae*) | ygl180wΔ | YSC1053 Yeast MATa deletion collection |  | http://www-sequence.stanford.edu/group/yeast_deletion_project/deletions3.html |
| strain, strain background (*S. cerevisiae*) | ygl186cΔ | YSC1053 Yeast MATa deletion collection |  | http://www-sequence.stanford.edu/group/yeast_deletion_project/deletions3.html |
| strain, strain background (*S. cerevisiae*) | ygl188c-aΔ | YSC1053 Yeast MATa deletion collection |  | http://www-sequence.stanford.edu/group/yeast_deletion_project/deletions3.html |
| strain, strain background (*S. cerevisiae*) | ygl190cΔ | YSC1053 Yeast MATa deletion collection |  | http://www-sequence.stanford.edu/group/yeast_deletion_project/deletions3.html |
| strain, strain background (*S. cerevisiae*) | ygl191wΔ | YSC1053 Yeast MATa deletion collection |  | http://www-sequence.stanford.edu/group/yeast_deletion_project/deletions3.html |
| strain, strain background (*S. cerevisiae*) | ygl195wΔ | YSC1053 Yeast MATa deletion collection |  | http://www-sequence.stanford.edu/group/yeast_deletion_project/deletions3.html |
| strain, strain background (*S. cerevisiae*) | ygl200cΔ | YSC1053 Yeast MATa deletion collection |  | http://www-sequence.stanford.edu/group/yeast_deletion_project/deletions3.html |
| strain, strain background (*S. cerevisiae*) | ygl202wΔ | YSC1053 Yeast MATa deletion collection |  | http://www-sequence.stanford.edu/group/yeast_deletion_project/deletions3.html |
| strain, strain background (*S. cerevisiae*) | ygl211wΔ | YSC1053 Yeast MATa deletion collection |  | http://www-sequence.stanford.edu/group/yeast_deletion_project/deletions3.html |
| strain, strain background (*S. cerevisiae*) | ygl212wΔ | YSC1053 Yeast MATa deletion collection |  | http://www-sequence.stanford.edu/group/yeast_deletion_project/deletions3.html |
| strain, strain background (*S. cerevisiae*) | ygl213cΔ | YSC1053 Yeast MATa deletion collection |  | http://www-sequence.stanford.edu/group/yeast_deletion_project/deletions3.html |
| strain, strain background (*S. cerevisiae*) | ygl218wΔ | YSC1053 Yeast MATa deletion collection |  | http://www-sequence.stanford.edu/group/yeast_deletion_project/deletions3.html |
| strain, strain background (*S. cerevisiae*) | ygl220wΔ | YSC1053 Yeast MATa deletion collection |  | http://www-sequence.stanford.edu/group/yeast_deletion_project/deletions3.html |
| strain, strain background (*S. cerevisiae*) | ygl221cΔ | YSC1053 Yeast MATa deletion collection |  | http://www-sequence.stanford.edu/group/yeast_deletion_project/deletions3.html |
| strain, strain background (*S. cerevisiae*) | ygl226c-aΔ | YSC1053 Yeast MATa deletion collection |  | http://www-sequence.stanford.edu/group/yeast_deletion_project/deletions3.html |
| strain, strain background (*S. cerevisiae*) | ygl226wΔ | YSC1053 Yeast MATa deletion collection |  | http://www-sequence.stanford.edu/group/yeast_deletion_project/deletions3.html |
| strain, strain background (*S. cerevisiae*) | ygl229cΔ | YSC1053 Yeast MATa deletion collection |  | http://www-sequence.stanford.edu/group/yeast_deletion_project/deletions3.html |
| strain, strain background (*S. cerevisiae*) | ygl236cΔ | YSC1053 Yeast MATa deletion collection |  | http://www-sequence.stanford.edu/group/yeast_deletion_project/deletions3.html |
| strain, strain background (*S. cerevisiae*) | ygl237cΔ | YSC1053 Yeast MATa deletion collection |  | http://www-sequence.stanford.edu/group/yeast_deletion_project/deletions3.html |
| strain, strain background (*S. cerevisiae*) | ygl240wΔ | YSC1053 Yeast MATa deletion collection |  | http://www-sequence.stanford.edu/group/yeast_deletion_project/deletions3.html |
| strain, strain background (*S. cerevisiae*) | ygl244wΔ | YSC1053 Yeast MATa deletion collection |  | http://www-sequence.stanford.edu/group/yeast_deletion_project/deletions3.html |
| strain, strain background (*S. cerevisiae*) | ygl245wΔ | YSC1053 Yeast MATa deletion collection |  | http://www-sequence.stanford.edu/group/yeast_deletion_project/deletions3.html |
| strain, strain background (*S. cerevisiae*) | ygl246cΔ | YSC1053 Yeast MATa deletion collection |  | http://www-sequence.stanford.edu/group/yeast_deletion_project/deletions3.html |
| strain, strain background (*S. cerevisiae*) | ygl252cΔ | YSC1053 Yeast MATa deletion collection |  | http://www-sequence.stanford.edu/group/yeast_deletion_project/deletions3.html |
| strain, strain background (*S. cerevisiae*) | ygl253wΔ | YSC1053 Yeast MATa deletion collection |  | http://www-sequence.stanford.edu/group/yeast_deletion_project/deletions3.html |
| strain, strain background (*S. cerevisiae*) | ygl255wΔ | YSC1053 Yeast MATa deletion collection |  | http://www-sequence.stanford.edu/group/yeast_deletion_project/deletions3.html |
| strain, strain background (*S. cerevisiae*) | ygr006wΔ | YSC1053 Yeast MATa deletion collection |  | http://www-sequence.stanford.edu/group/yeast_deletion_project/deletions3.html |
| strain, strain background (*S. cerevisiae*) | ygr020cΔ | YSC1053 Yeast MATa deletion collection |  | http://www-sequence.stanford.edu/group/yeast_deletion_project/deletions3.html |
| strain, strain background (*S. cerevisiae*) | ygr027cΔ | YSC1053 Yeast MATa deletion collection |  | http://www-sequence.stanford.edu/group/yeast_deletion_project/deletions3.html |
| strain, strain background (*S. cerevisiae*) | ygr033cΔ | YSC1053 Yeast MATa deletion collection |  | http://www-sequence.stanford.edu/group/yeast_deletion_project/deletions3.html |
| strain, strain background (*S. cerevisiae*) | ygr034wΔ | YSC1053 Yeast MATa deletion collection |  | http://www-sequence.stanford.edu/group/yeast_deletion_project/deletions3.html |
| strain, strain background (*S. cerevisiae*) | ygr036cΔ | YSC1053 Yeast MATa deletion collection |  | http://www-sequence.stanford.edu/group/yeast_deletion_project/deletions3.html |
| strain, strain background (*S. cerevisiae*) | ygr039wΔ | YSC1053 Yeast MATa deletion collection |  | http://www-sequence.stanford.edu/group/yeast_deletion_project/deletions3.html |
| strain, strain background (*S. cerevisiae*) | ygr040wΔ | YSC1053 Yeast MATa deletion collection |  | http://www-sequence.stanford.edu/group/yeast_deletion_project/deletions3.html |
| strain, strain background (*S. cerevisiae*) | ygr049wΔ | YSC1053 Yeast MATa deletion collection |  | http://www-sequence.stanford.edu/group/yeast_deletion_project/deletions3.html |
| strain, strain background (*S. cerevisiae*) | ygr050cΔ | YSC1053 Yeast MATa deletion collection |  | http://www-sequence.stanford.edu/group/yeast_deletion_project/deletions3.html |
| strain, strain background (*S. cerevisiae*) | ygr052wΔ | YSC1053 Yeast MATa deletion collection |  | http://www-sequence.stanford.edu/group/yeast_deletion_project/deletions3.html |
| strain, strain background (*S. cerevisiae*) | ygr057cΔ | YSC1053 Yeast MATa deletion collection |  | http://www-sequence.stanford.edu/group/yeast_deletion_project/deletions3.html |
| strain, strain background (*S. cerevisiae*) | ygr058wΔ | YSC1053 Yeast MATa deletion collection |  | http://www-sequence.stanford.edu/group/yeast_deletion_project/deletions3.html |
| strain, strain background (*S. cerevisiae*) | ygr062cΔ | YSC1053 Yeast MATa deletion collection |  | http://www-sequence.stanford.edu/group/yeast_deletion_project/deletions3.html |
| strain, strain background (*S. cerevisiae*) | ygr063cΔ | YSC1053 Yeast MATa deletion collection |  | http://www-sequence.stanford.edu/group/yeast_deletion_project/deletions3.html |
| strain, strain background (*S. cerevisiae*) | ygr064wΔ | YSC1053 Yeast MATa deletion collection |  | http://www-sequence.stanford.edu/group/yeast_deletion_project/deletions3.html |
| strain, strain background (*S. cerevisiae*) | ygr066cΔ | YSC1053 Yeast MATa deletion collection |  | http://www-sequence.stanford.edu/group/yeast_deletion_project/deletions3.html |
| strain, strain background (*S. cerevisiae*) | ygr067cΔ | YSC1053 Yeast MATa deletion collection |  | http://www-sequence.stanford.edu/group/yeast_deletion_project/deletions3.html |
| strain, strain background (*S. cerevisiae*) | ygr069wΔ | YSC1053 Yeast MATa deletion collection |  | http://www-sequence.stanford.edu/group/yeast_deletion_project/deletions3.html |
| strain, strain background (*S. cerevisiae*) | ygr072wΔ | YSC1053 Yeast MATa deletion collection |  | http://www-sequence.stanford.edu/group/yeast_deletion_project/deletions3.html |
| strain, strain background (*S. cerevisiae*) | ygr076cΔ | YSC1053 Yeast MATa deletion collection |  | http://www-sequence.stanford.edu/group/yeast_deletion_project/deletions3.html |
| strain, strain background (*S. cerevisiae*) | ygr077cΔ | YSC1053 Yeast MATa deletion collection |  | http://www-sequence.stanford.edu/group/yeast_deletion_project/deletions3.html |
| strain, strain background (*S. cerevisiae*) | ygr078cΔ | YSC1053 Yeast MATa deletion collection |  | http://www-sequence.stanford.edu/group/yeast_deletion_project/deletions3.html |
| strain, strain background (*S. cerevisiae*) | ygr080wΔ | YSC1053 Yeast MATa deletion collection |  | http://www-sequence.stanford.edu/group/yeast_deletion_project/deletions3.html |
| strain, strain background (*S. cerevisiae*) | ygr081cΔ | YSC1053 Yeast MATa deletion collection |  | http://www-sequence.stanford.edu/group/yeast_deletion_project/deletions3.html |
| strain, strain background (*S. cerevisiae*) | ygr092wΔ | YSC1053 Yeast MATa deletion collection |  | http://www-sequence.stanford.edu/group/yeast_deletion_project/deletions3.html |
| strain, strain background (*S. cerevisiae*) | ygr097wΔ | YSC1053 Yeast MATa deletion collection |  | http://www-sequence.stanford.edu/group/yeast_deletion_project/deletions3.html |
| strain, strain background (*S. cerevisiae*) | ygr101wΔ | YSC1053 Yeast MATa deletion collection |  | http://www-sequence.stanford.edu/group/yeast_deletion_project/deletions3.html |
| strain, strain background (*S. cerevisiae*) | ygr102cΔ | YSC1053 Yeast MATa deletion collection |  | http://www-sequence.stanford.edu/group/yeast_deletion_project/deletions3.html |
| strain, strain background (*S. cerevisiae*) | ygr104cΔ | YSC1053 Yeast MATa deletion collection |  | http://www-sequence.stanford.edu/group/yeast_deletion_project/deletions3.html |
| strain, strain background (*S. cerevisiae*) | ygr105wΔ | YSC1053 Yeast MATa deletion collection |  | http://www-sequence.stanford.edu/group/yeast_deletion_project/deletions3.html |
| strain, strain background (*S. cerevisiae*) | ygr118wΔ | YSC1053 Yeast MATa deletion collection |  | http://www-sequence.stanford.edu/group/yeast_deletion_project/deletions3.html |
| strain, strain background (*S. cerevisiae*) | ygr122c-aΔ | YSC1053 Yeast MATa deletion collection |  | http://www-sequence.stanford.edu/group/yeast_deletion_project/deletions3.html |
| strain, strain background (*S. cerevisiae*) | ygr122wΔ | YSC1053 Yeast MATa deletion collection |  | http://www-sequence.stanford.edu/group/yeast_deletion_project/deletions3.html |
| strain, strain background (*S. cerevisiae*) | ygr123cΔ | YSC1053 Yeast MATa deletion collection |  | http://www-sequence.stanford.edu/group/yeast_deletion_project/deletions3.html |
| strain, strain background (*S. cerevisiae*) | ygr125wΔ | YSC1053 Yeast MATa deletion collection |  | http://www-sequence.stanford.edu/group/yeast_deletion_project/deletions3.html |
| strain, strain background (*S. cerevisiae*) | ygr133wΔ | YSC1053 Yeast MATa deletion collection |  | http://www-sequence.stanford.edu/group/yeast_deletion_project/deletions3.html |
| strain, strain background (*S. cerevisiae*) | ygr135wΔ | YSC1053 Yeast MATa deletion collection |  | http://www-sequence.stanford.edu/group/yeast_deletion_project/deletions3.html |
| strain, strain background (*S. cerevisiae*) | ygr141wΔ | YSC1053 Yeast MATa deletion collection |  | http://www-sequence.stanford.edu/group/yeast_deletion_project/deletions3.html |
| strain, strain background (*S. cerevisiae*) | ygr144wΔ | YSC1053 Yeast MATa deletion collection |  | http://www-sequence.stanford.edu/group/yeast_deletion_project/deletions3.html |
| strain, strain background (*S. cerevisiae*) | ygr148cΔ | YSC1053 Yeast MATa deletion collection |  | http://www-sequence.stanford.edu/group/yeast_deletion_project/deletions3.html |
| strain, strain background (*S. cerevisiae*) | ygr150cΔ | YSC1053 Yeast MATa deletion collection |  | http://www-sequence.stanford.edu/group/yeast_deletion_project/deletions3.html |
| strain, strain background (*S. cerevisiae*) | ygr155wΔ | YSC1053 Yeast MATa deletion collection |  | http://www-sequence.stanford.edu/group/yeast_deletion_project/deletions3.html |
| strain, strain background (*S. cerevisiae*) | ygr159cΔ | YSC1053 Yeast MATa deletion collection |  | http://www-sequence.stanford.edu/group/yeast_deletion_project/deletions3.html |
| strain, strain background (*S. cerevisiae*) | ygr160wΔ | YSC1053 Yeast MATa deletion collection |  | http://www-sequence.stanford.edu/group/yeast_deletion_project/deletions3.html |
| strain, strain background (*S. cerevisiae*) | ygr162wΔ | YSC1053 Yeast MATa deletion collection |  | http://www-sequence.stanford.edu/group/yeast_deletion_project/deletions3.html |
| strain, strain background (*S. cerevisiae*) | ygr163wΔ | YSC1053 Yeast MATa deletion collection |  | http://www-sequence.stanford.edu/group/yeast_deletion_project/deletions3.html |
| strain, strain background (*S. cerevisiae*) | ygr165wΔ | YSC1053 Yeast MATa deletion collection |  | http://www-sequence.stanford.edu/group/yeast_deletion_project/deletions3.html |
| strain, strain background (*S. cerevisiae*) | ygr166wΔ | YSC1053 Yeast MATa deletion collection |  | http://www-sequence.stanford.edu/group/yeast_deletion_project/deletions3.html |
| strain, strain background (*S. cerevisiae*) | ygr171cΔ | YSC1053 Yeast MATa deletion collection |  | http://www-sequence.stanford.edu/group/yeast_deletion_project/deletions3.html |
| strain, strain background (*S. cerevisiae*) | ygr178cΔ | YSC1053 Yeast MATa deletion collection |  | http://www-sequence.stanford.edu/group/yeast_deletion_project/deletions3.html |
| strain, strain background (*S. cerevisiae*) | ygr183cΔ | YSC1053 Yeast MATa deletion collection |  | http://www-sequence.stanford.edu/group/yeast_deletion_project/deletions3.html |
| strain, strain background (*S. cerevisiae*) | ygr188cΔ | YSC1053 Yeast MATa deletion collection |  | http://www-sequence.stanford.edu/group/yeast_deletion_project/deletions3.html |
| strain, strain background (*S. cerevisiae*) | ygr200cΔ | YSC1053 Yeast MATa deletion collection |  | http://www-sequence.stanford.edu/group/yeast_deletion_project/deletions3.html |
| strain, strain background (*S. cerevisiae*) | ygr204wΔ | YSC1053 Yeast MATa deletion collection |  | http://www-sequence.stanford.edu/group/yeast_deletion_project/deletions3.html |
| strain, strain background (*S. cerevisiae*) | ygr205wΔ | YSC1053 Yeast MATa deletion collection |  | http://www-sequence.stanford.edu/group/yeast_deletion_project/deletions3.html |
| strain, strain background (*S. cerevisiae*) | ygr208wΔ | YSC1053 Yeast MATa deletion collection |  | http://www-sequence.stanford.edu/group/yeast_deletion_project/deletions3.html |
| strain, strain background (*S. cerevisiae*) | ygr214wΔ | YSC1053 Yeast MATa deletion collection |  | http://www-sequence.stanford.edu/group/yeast_deletion_project/deletions3.html |
| strain, strain background (*S. cerevisiae*) | ygr215wΔ | YSC1053 Yeast MATa deletion collection |  | http://www-sequence.stanford.edu/group/yeast_deletion_project/deletions3.html |
| strain, strain background (*S. cerevisiae*) | ygr219wΔ | YSC1053 Yeast MATa deletion collection |  | http://www-sequence.stanford.edu/group/yeast_deletion_project/deletions3.html |
| strain, strain background (*S. cerevisiae*) | ygr220cΔ | YSC1053 Yeast MATa deletion collection |  | http://www-sequence.stanford.edu/group/yeast_deletion_project/deletions3.html |
| strain, strain background (*S. cerevisiae*) | ygr222wΔ | YSC1053 Yeast MATa deletion collection |  | http://www-sequence.stanford.edu/group/yeast_deletion_project/deletions3.html |
| strain, strain background (*S. cerevisiae*) | ygr226cΔ | YSC1053 Yeast MATa deletion collection |  | http://www-sequence.stanford.edu/group/yeast_deletion_project/deletions3.html |
| strain, strain background (*S. cerevisiae*) | ygr227wΔ | YSC1053 Yeast MATa deletion collection |  | http://www-sequence.stanford.edu/group/yeast_deletion_project/deletions3.html |
| strain, strain background (*S. cerevisiae*) | ygr229cΔ | YSC1053 Yeast MATa deletion collection |  | http://www-sequence.stanford.edu/group/yeast_deletion_project/deletions3.html |
| strain, strain background (*S. cerevisiae*) | ygr232wΔ | YSC1053 Yeast MATa deletion collection |  | http://www-sequence.stanford.edu/group/yeast_deletion_project/deletions3.html |
| strain, strain background (*S. cerevisiae*) | ygr237cΔ | YSC1053 Yeast MATa deletion collection |  | http://www-sequence.stanford.edu/group/yeast_deletion_project/deletions3.html |
| strain, strain background (*S. cerevisiae*) | ygr240cΔ | YSC1053 Yeast MATa deletion collection |  | http://www-sequence.stanford.edu/group/yeast_deletion_project/deletions3.html |
| strain, strain background (*S. cerevisiae*) | ygr255cΔ | YSC1053 Yeast MATa deletion collection |  | http://www-sequence.stanford.edu/group/yeast_deletion_project/deletions3.html |
| strain, strain background (*S. cerevisiae*) | ygr257cΔ | YSC1053 Yeast MATa deletion collection |  | http://www-sequence.stanford.edu/group/yeast_deletion_project/deletions3.html |
| strain, strain background (*S. cerevisiae*) | ygr258cΔ | YSC1053 Yeast MATa deletion collection |  | http://www-sequence.stanford.edu/group/yeast_deletion_project/deletions3.html |
| strain, strain background (*S. cerevisiae*) | ygr266wΔ | YSC1053 Yeast MATa deletion collection |  | http://www-sequence.stanford.edu/group/yeast_deletion_project/deletions3.html |
| strain, strain background (*S. cerevisiae*) | ygr270wΔ | YSC1053 Yeast MATa deletion collection |  | http://www-sequence.stanford.edu/group/yeast_deletion_project/deletions3.html |
| strain, strain background (*S. cerevisiae*) | ygr272cΔ | YSC1053 Yeast MATa deletion collection |  | http://www-sequence.stanford.edu/group/yeast_deletion_project/deletions3.html |
| strain, strain background (*S. cerevisiae*) | ygr285cΔ | YSC1053 Yeast MATa deletion collection |  | http://www-sequence.stanford.edu/group/yeast_deletion_project/deletions3.html |
| strain, strain background (*S. cerevisiae*) | yhl004wΔ | YSC1053 Yeast MATa deletion collection |  | http://www-sequence.stanford.edu/group/yeast_deletion_project/deletions3.html |
| strain, strain background (*S. cerevisiae*) | yhl013cΔ | YSC1053 Yeast MATa deletion collection |  | http://www-sequence.stanford.edu/group/yeast_deletion_project/deletions3.html |
| strain, strain background (*S. cerevisiae*) | yhl014cΔ | YSC1053 Yeast MATa deletion collection |  | http://www-sequence.stanford.edu/group/yeast_deletion_project/deletions3.html |
| strain, strain background (*S. cerevisiae*) | yhl020cΔ | YSC1053 Yeast MATa deletion collection |  | http://www-sequence.stanford.edu/group/yeast_deletion_project/deletions3.html |
| strain, strain background (*S. cerevisiae*) | yhl023cΔ | YSC1053 Yeast MATa deletion collection |  | http://www-sequence.stanford.edu/group/yeast_deletion_project/deletions3.html |
| strain, strain background (*S. cerevisiae*) | yhl027wΔ | YSC1053 Yeast MATa deletion collection |  | http://www-sequence.stanford.edu/group/yeast_deletion_project/deletions3.html |
| strain, strain background (*S. cerevisiae*) | yhl029cΔ | YSC1053 Yeast MATa deletion collection |  | http://www-sequence.stanford.edu/group/yeast_deletion_project/deletions3.html |
| strain, strain background (*S. cerevisiae*) | yhl031cΔ | YSC1053 Yeast MATa deletion collection |  | http://www-sequence.stanford.edu/group/yeast_deletion_project/deletions3.html |
| strain, strain background (*S. cerevisiae*) | yhl033cΔ | YSC1053 Yeast MATa deletion collection |  | http://www-sequence.stanford.edu/group/yeast_deletion_project/deletions3.html |
| strain, strain background (*S. cerevisiae*) | yhl034cΔ | YSC1053 Yeast MATa deletion collection |  | http://www-sequence.stanford.edu/group/yeast_deletion_project/deletions3.html |
| strain, strain background (*S. cerevisiae*) | yhl038cΔ | YSC1053 Yeast MATa deletion collection |  | http://www-sequence.stanford.edu/group/yeast_deletion_project/deletions3.html |
| strain, strain background (*S. cerevisiae*) | yhr004cΔ | YSC1053 Yeast MATa deletion collection |  | http://www-sequence.stanford.edu/group/yeast_deletion_project/deletions3.html |
| strain, strain background (*S. cerevisiae*) | yhr011wΔ | YSC1053 Yeast MATa deletion collection |  | http://www-sequence.stanford.edu/group/yeast_deletion_project/deletions3.html |
| strain, strain background (*S. cerevisiae*) | yhr012wΔ | YSC1053 Yeast MATa deletion collection |  | http://www-sequence.stanford.edu/group/yeast_deletion_project/deletions3.html |
| strain, strain background (*S. cerevisiae*) | yhr013cΔ | YSC1053 Yeast MATa deletion collection |  | http://www-sequence.stanford.edu/group/yeast_deletion_project/deletions3.html |
| strain, strain background (*S. cerevisiae*) | yhr021cΔ | YSC1053 Yeast MATa deletion collection |  | http://www-sequence.stanford.edu/group/yeast_deletion_project/deletions3.html |
| strain, strain background (*S. cerevisiae*) | yhr026wΔ | YSC1053 Yeast MATa deletion collection |  | http://www-sequence.stanford.edu/group/yeast_deletion_project/deletions3.html |
| strain, strain background (*S. cerevisiae*) | yhr034cΔ | YSC1053 Yeast MATa deletion collection |  | http://www-sequence.stanford.edu/group/yeast_deletion_project/deletions3.html |
| strain, strain background (*S. cerevisiae*) | yhr038wΔ | YSC1053 Yeast MATa deletion collection |  | http://www-sequence.stanford.edu/group/yeast_deletion_project/deletions3.html |
| strain, strain background (*S. cerevisiae*) | yhr041cΔ | YSC1053 Yeast MATa deletion collection |  | http://www-sequence.stanford.edu/group/yeast_deletion_project/deletions3.html |
| strain, strain background (*S. cerevisiae*) | yhr043cΔ | YSC1053 Yeast MATa deletion collection |  | http://www-sequence.stanford.edu/group/yeast_deletion_project/deletions3.html |
| strain, strain background (*S. cerevisiae*) | yhr045wΔ | YSC1053 Yeast MATa deletion collection |  | http://www-sequence.stanford.edu/group/yeast_deletion_project/deletions3.html |
| strain, strain background (*S. cerevisiae*) | yhr049wΔ | YSC1053 Yeast MATa deletion collection |  | http://www-sequence.stanford.edu/group/yeast_deletion_project/deletions3.html |
| strain, strain background (*S. cerevisiae*) | yhr050wΔ | YSC1053 Yeast MATa deletion collection |  | http://www-sequence.stanford.edu/group/yeast_deletion_project/deletions3.html |
| strain, strain background (*S. cerevisiae*) | yhr050w-aΔ | YSC1053 Yeast MATa deletion collection |  | http://www-sequence.stanford.edu/group/yeast_deletion_project/deletions3.html |
| strain, strain background (*S. cerevisiae*) | yhr051wΔ | YSC1053 Yeast MATa deletion collection |  | http://www-sequence.stanford.edu/group/yeast_deletion_project/deletions3.html |
| strain, strain background (*S. cerevisiae*) | yhr057cΔ | YSC1053 Yeast MATa deletion collection |  | http://www-sequence.stanford.edu/group/yeast_deletion_project/deletions3.html |
| strain, strain background (*S. cerevisiae*) | yhr060wΔ | YSC1053 Yeast MATa deletion collection |  | http://www-sequence.stanford.edu/group/yeast_deletion_project/deletions3.html |
| strain, strain background (*S. cerevisiae*) | yhr064cΔ | YSC1053 Yeast MATa deletion collection |  | http://www-sequence.stanford.edu/group/yeast_deletion_project/deletions3.html |
| strain, strain background (*S. cerevisiae*) | yhr066wΔ | YSC1053 Yeast MATa deletion collection |  | http://www-sequence.stanford.edu/group/yeast_deletion_project/deletions3.html |
| strain, strain background (*S. cerevisiae*) | yhr067wΔ | YSC1053 Yeast MATa deletion collection |  | http://www-sequence.stanford.edu/group/yeast_deletion_project/deletions3.html |
| strain, strain background (*S. cerevisiae*) | yhr078wΔ | YSC1053 Yeast MATa deletion collection |  | http://www-sequence.stanford.edu/group/yeast_deletion_project/deletions3.html |
| strain, strain background (*S. cerevisiae*) | yhr081wΔ | YSC1053 Yeast MATa deletion collection |  | http://www-sequence.stanford.edu/group/yeast_deletion_project/deletions3.html |
| strain, strain background (*S. cerevisiae*) | yhr091cΔ | YSC1053 Yeast MATa deletion collection |  | http://www-sequence.stanford.edu/group/yeast_deletion_project/deletions3.html |
| strain, strain background (*S. cerevisiae*) | yhr100cΔ | YSC1053 Yeast MATa deletion collection |  | http://www-sequence.stanford.edu/group/yeast_deletion_project/deletions3.html |
| strain, strain background (*S. cerevisiae*) | yhr108wΔ | YSC1053 Yeast MATa deletion collection |  | http://www-sequence.stanford.edu/group/yeast_deletion_project/deletions3.html |
| strain, strain background (*S. cerevisiae*) | yhr111wΔ | YSC1053 Yeast MATa deletion collection |  | http://www-sequence.stanford.edu/group/yeast_deletion_project/deletions3.html |
| strain, strain background (*S. cerevisiae*) | yhr114wΔ | YSC1053 Yeast MATa deletion collection |  | http://www-sequence.stanford.edu/group/yeast_deletion_project/deletions3.html |
| strain, strain background (*S. cerevisiae*) | yhr115cΔ | YSC1053 Yeast MATa deletion collection |  | http://www-sequence.stanford.edu/group/yeast_deletion_project/deletions3.html |
| strain, strain background (*S. cerevisiae*) | yhr116wΔ | YSC1053 Yeast MATa deletion collection |  | http://www-sequence.stanford.edu/group/yeast_deletion_project/deletions3.html |
| strain, strain background (*S. cerevisiae*) | yhr120wΔ | YSC1053 Yeast MATa deletion collection |  | http://www-sequence.stanford.edu/group/yeast_deletion_project/deletions3.html |
| strain, strain background (*S. cerevisiae*) | yhr127wΔ | YSC1053 Yeast MATa deletion collection |  | http://www-sequence.stanford.edu/group/yeast_deletion_project/deletions3.html |
| strain, strain background (*S. cerevisiae*) | yhr129cΔ | YSC1053 Yeast MATa deletion collection |  | http://www-sequence.stanford.edu/group/yeast_deletion_project/deletions3.html |
| strain, strain background (*S. cerevisiae*) | yhr134wΔ | YSC1053 Yeast MATa deletion collection |  | http://www-sequence.stanford.edu/group/yeast_deletion_project/deletions3.html |
| strain, strain background (*S. cerevisiae*) | yhr139cΔ | YSC1053 Yeast MATa deletion collection |  | http://www-sequence.stanford.edu/group/yeast_deletion_project/deletions3.html |
| strain, strain background (*S. cerevisiae*) | yhr140wΔ | YSC1053 Yeast MATa deletion collection |  | http://www-sequence.stanford.edu/group/yeast_deletion_project/deletions3.html |
| strain, strain background (*S. cerevisiae*) | yhr141cΔ | YSC1053 Yeast MATa deletion collection |  | http://www-sequence.stanford.edu/group/yeast_deletion_project/deletions3.html |
| strain, strain background (*S. cerevisiae*) | yhr147cΔ | YSC1053 Yeast MATa deletion collection |  | http://www-sequence.stanford.edu/group/yeast_deletion_project/deletions3.html |
| strain, strain background (*S. cerevisiae*) | yhr157wΔ | YSC1053 Yeast MATa deletion collection |  | http://www-sequence.stanford.edu/group/yeast_deletion_project/deletions3.html |
| strain, strain background (*S. cerevisiae*) | yhr168wΔ | YSC1053 Yeast MATa deletion collection |  | http://www-sequence.stanford.edu/group/yeast_deletion_project/deletions3.html |
| strain, strain background (*S. cerevisiae*) | yhr178wΔ | YSC1053 Yeast MATa deletion collection |  | http://www-sequence.stanford.edu/group/yeast_deletion_project/deletions3.html |
| strain, strain background (*S. cerevisiae*) | yhr183wΔ | YSC1053 Yeast MATa deletion collection |  | http://www-sequence.stanford.edu/group/yeast_deletion_project/deletions3.html |
| strain, strain background (*S. cerevisiae*) | yhr187wΔ | YSC1053 Yeast MATa deletion collection |  | http://www-sequence.stanford.edu/group/yeast_deletion_project/deletions3.html |
| strain, strain background (*S. cerevisiae*) | yhr189wΔ | YSC1053 Yeast MATa deletion collection |  | http://www-sequence.stanford.edu/group/yeast_deletion_project/deletions3.html |
| strain, strain background (*S. cerevisiae*) | yhr191cΔ | YSC1053 Yeast MATa deletion collection |  | http://www-sequence.stanford.edu/group/yeast_deletion_project/deletions3.html |
| strain, strain background (*S. cerevisiae*) | yhr193cΔ | YSC1053 Yeast MATa deletion collection |  | http://www-sequence.stanford.edu/group/yeast_deletion_project/deletions3.html |
| strain, strain background (*S. cerevisiae*) | yhr194wΔ | YSC1053 Yeast MATa deletion collection |  | http://www-sequence.stanford.edu/group/yeast_deletion_project/deletions3.html |
| strain, strain background (*S. cerevisiae*) | yhr199cΔ | YSC1053 Yeast MATa deletion collection |  | http://www-sequence.stanford.edu/group/yeast_deletion_project/deletions3.html |
| strain, strain background (*S. cerevisiae*) | yhr200wΔ | YSC1053 Yeast MATa deletion collection |  | http://www-sequence.stanford.edu/group/yeast_deletion_project/deletions3.html |
| strain, strain background (*S. cerevisiae*) | yhr203cΔ | YSC1053 Yeast MATa deletion collection |  | http://www-sequence.stanford.edu/group/yeast_deletion_project/deletions3.html |
| strain, strain background (*S. cerevisiae*) | yhr204wΔ | YSC1053 Yeast MATa deletion collection |  | http://www-sequence.stanford.edu/group/yeast_deletion_project/deletions3.html |
| strain, strain background (*S. cerevisiae*) | yil001wΔ | YSC1053 Yeast MATa deletion collection |  | http://www-sequence.stanford.edu/group/yeast_deletion_project/deletions3.html |
| strain, strain background (*S. cerevisiae*) | yil005wΔ | YSC1053 Yeast MATa deletion collection |  | http://www-sequence.stanford.edu/group/yeast_deletion_project/deletions3.html |
| strain, strain background (*S. cerevisiae*) | yil006wΔ | YSC1053 Yeast MATa deletion collection |  | http://www-sequence.stanford.edu/group/yeast_deletion_project/deletions3.html |
| strain, strain background (*S. cerevisiae*) | yil007cΔ | YSC1053 Yeast MATa deletion collection |  | http://www-sequence.stanford.edu/group/yeast_deletion_project/deletions3.html |
| strain, strain background (*S. cerevisiae*) | yil008wΔ | YSC1053 Yeast MATa deletion collection |  | http://www-sequence.stanford.edu/group/yeast_deletion_project/deletions3.html |
| strain, strain background (*S. cerevisiae*) | yil009c-aΔ | YSC1053 Yeast MATa deletion collection |  | http://www-sequence.stanford.edu/group/yeast_deletion_project/deletions3.html |
| strain, strain background (*S. cerevisiae*) | yil010wΔ | YSC1053 Yeast MATa deletion collection |  | http://www-sequence.stanford.edu/group/yeast_deletion_project/deletions3.html |
| strain, strain background (*S. cerevisiae*) | yil011wΔ | YSC1053 Yeast MATa deletion collection |  | http://www-sequence.stanford.edu/group/yeast_deletion_project/deletions3.html |
| strain, strain background (*S. cerevisiae*) | yil015wΔ | YSC1053 Yeast MATa deletion collection |  | http://www-sequence.stanford.edu/group/yeast_deletion_project/deletions3.html |
| strain, strain background (*S. cerevisiae*) | yil016wΔ | YSC1053 Yeast MATa deletion collection |  | http://www-sequence.stanford.edu/group/yeast_deletion_project/deletions3.html |
| strain, strain background (*S. cerevisiae*) | yil018wΔ | YSC1053 Yeast MATa deletion collection |  | http://www-sequence.stanford.edu/group/yeast_deletion_project/deletions3.html |
| strain, strain background (*S. cerevisiae*) | yil023cΔ | YSC1053 Yeast MATa deletion collection |  | http://www-sequence.stanford.edu/group/yeast_deletion_project/deletions3.html |
| strain, strain background (*S. cerevisiae*) | yil028wΔ | YSC1053 Yeast MATa deletion collection |  | http://www-sequence.stanford.edu/group/yeast_deletion_project/deletions3.html |
| strain, strain background (*S. cerevisiae*) | yil029cΔ | YSC1053 Yeast MATa deletion collection |  | http://www-sequence.stanford.edu/group/yeast_deletion_project/deletions3.html |
| strain, strain background (*S. cerevisiae*) | yil032cΔ | YSC1053 Yeast MATa deletion collection |  | http://www-sequence.stanford.edu/group/yeast_deletion_project/deletions3.html |
| strain, strain background (*S. cerevisiae*) | yil037cΔ | YSC1053 Yeast MATa deletion collection |  | http://www-sequence.stanford.edu/group/yeast_deletion_project/deletions3.html |
| strain, strain background (*S. cerevisiae*) | yil038cΔ | YSC1053 Yeast MATa deletion collection |  | http://www-sequence.stanford.edu/group/yeast_deletion_project/deletions3.html |
| strain, strain background (*S. cerevisiae*) | yil040wΔ | YSC1053 Yeast MATa deletion collection |  | http://www-sequence.stanford.edu/group/yeast_deletion_project/deletions3.html |
| strain, strain background (*S. cerevisiae*) | yil043cΔ | YSC1053 Yeast MATa deletion collection |  | http://www-sequence.stanford.edu/group/yeast_deletion_project/deletions3.html |
| strain, strain background (*S. cerevisiae*) | yil044cΔ | YSC1053 Yeast MATa deletion collection |  | http://www-sequence.stanford.edu/group/yeast_deletion_project/deletions3.html |
| strain, strain background (*S. cerevisiae*) | yil045wΔ | YSC1053 Yeast MATa deletion collection |  | http://www-sequence.stanford.edu/group/yeast_deletion_project/deletions3.html |
| strain, strain background (*S. cerevisiae*) | yil050wΔ | YSC1053 Yeast MATa deletion collection |  | http://www-sequence.stanford.edu/group/yeast_deletion_project/deletions3.html |
| strain, strain background (*S. cerevisiae*) | yil052cΔ | YSC1053 Yeast MATa deletion collection |  | http://www-sequence.stanford.edu/group/yeast_deletion_project/deletions3.html |
| strain, strain background (*S. cerevisiae*) | yil053wΔ | YSC1053 Yeast MATa deletion collection |  | http://www-sequence.stanford.edu/group/yeast_deletion_project/deletions3.html |
| strain, strain background (*S. cerevisiae*) | yil055cΔ | YSC1053 Yeast MATa deletion collection |  | http://www-sequence.stanford.edu/group/yeast_deletion_project/deletions3.html |
| strain, strain background (*S. cerevisiae*) | yil056wΔ | YSC1053 Yeast MATa deletion collection |  | http://www-sequence.stanford.edu/group/yeast_deletion_project/deletions3.html |
| strain, strain background (*S. cerevisiae*) | yil057cΔ | YSC1053 Yeast MATa deletion collection |  | http://www-sequence.stanford.edu/group/yeast_deletion_project/deletions3.html |
| strain, strain background (*S. cerevisiae*) | yil060wΔ | YSC1053 Yeast MATa deletion collection |  | http://www-sequence.stanford.edu/group/yeast_deletion_project/deletions3.html |
| strain, strain background (*S. cerevisiae*) | yil064wΔ | YSC1053 Yeast MATa deletion collection |  | http://www-sequence.stanford.edu/group/yeast_deletion_project/deletions3.html |
| strain, strain background (*S. cerevisiae*) | yil065cΔ | YSC1053 Yeast MATa deletion collection |  | http://www-sequence.stanford.edu/group/yeast_deletion_project/deletions3.html |
| strain, strain background (*S. cerevisiae*) | yil066cΔ | YSC1053 Yeast MATa deletion collection |  | http://www-sequence.stanford.edu/group/yeast_deletion_project/deletions3.html |
| strain, strain background (*S. cerevisiae*) | yil069cΔ | YSC1053 Yeast MATa deletion collection |  | http://www-sequence.stanford.edu/group/yeast_deletion_project/deletions3.html |
| strain, strain background (*S. cerevisiae*) | yil070cΔ | YSC1053 Yeast MATa deletion collection |  | http://www-sequence.stanford.edu/group/yeast_deletion_project/deletions3.html |
| strain, strain background (*S. cerevisiae*) | yil073cΔ | YSC1053 Yeast MATa deletion collection |  | http://www-sequence.stanford.edu/group/yeast_deletion_project/deletions3.html |
| strain, strain background (*S. cerevisiae*) | yil076wΔ | YSC1053 Yeast MATa deletion collection |  | http://www-sequence.stanford.edu/group/yeast_deletion_project/deletions3.html |
| strain, strain background (*S. cerevisiae*) | yil077cΔ | YSC1053 Yeast MATa deletion collection |  | http://www-sequence.stanford.edu/group/yeast_deletion_project/deletions3.html |
| strain, strain background (*S. cerevisiae*) | yil084cΔ | YSC1053 Yeast MATa deletion collection |  | http://www-sequence.stanford.edu/group/yeast_deletion_project/deletions3.html |
| strain, strain background (*S. cerevisiae*) | yil087cΔ | YSC1053 Yeast MATa deletion collection |  | http://www-sequence.stanford.edu/group/yeast_deletion_project/deletions3.html |
| strain, strain background (*S. cerevisiae*) | yil090wΔ | YSC1053 Yeast MATa deletion collection |  | http://www-sequence.stanford.edu/group/yeast_deletion_project/deletions3.html |
| strain, strain background (*S. cerevisiae*) | yil092wΔ | YSC1053 Yeast MATa deletion collection |  | http://www-sequence.stanford.edu/group/yeast_deletion_project/deletions3.html |
| strain, strain background (*S. cerevisiae*) | yil093cΔ | YSC1053 Yeast MATa deletion collection |  | http://www-sequence.stanford.edu/group/yeast_deletion_project/deletions3.html |
| strain, strain background (*S. cerevisiae*) | yil094cΔ | YSC1053 Yeast MATa deletion collection |  | http://www-sequence.stanford.edu/group/yeast_deletion_project/deletions3.html |
| strain, strain background (*S. cerevisiae*) | yil095wΔ | YSC1053 Yeast MATa deletion collection |  | http://www-sequence.stanford.edu/group/yeast_deletion_project/deletions3.html |
| strain, strain background (*S. cerevisiae*) | yil096cΔ | YSC1053 Yeast MATa deletion collection |  | http://www-sequence.stanford.edu/group/yeast_deletion_project/deletions3.html |
| strain, strain background (*S. cerevisiae*) | yil098cΔ | YSC1053 Yeast MATa deletion collection |  | http://www-sequence.stanford.edu/group/yeast_deletion_project/deletions3.html |
| strain, strain background (*S. cerevisiae*) | yil101cΔ | YSC1053 Yeast MATa deletion collection |  | http://www-sequence.stanford.edu/group/yeast_deletion_project/deletions3.html |
| strain, strain background (*S. cerevisiae*) | yil105cΔ | YSC1053 Yeast MATa deletion collection |  | http://www-sequence.stanford.edu/group/yeast_deletion_project/deletions3.html |
| strain, strain background (*S. cerevisiae*) | yil107cΔ | YSC1053 Yeast MATa deletion collection |  | http://www-sequence.stanford.edu/group/yeast_deletion_project/deletions3.html |
| strain, strain background (*S. cerevisiae*) | yil110wΔ | YSC1053 Yeast MATa deletion collection |  | http://www-sequence.stanford.edu/group/yeast_deletion_project/deletions3.html |
| strain, strain background (*S. cerevisiae*) | yil111wΔ | YSC1053 Yeast MATa deletion collection |  | http://www-sequence.stanford.edu/group/yeast_deletion_project/deletions3.html |
| strain, strain background (*S. cerevisiae*) | yil112wΔ | YSC1053 Yeast MATa deletion collection |  | http://www-sequence.stanford.edu/group/yeast_deletion_project/deletions3.html |
| strain, strain background (*S. cerevisiae*) | yil116wΔ | YSC1053 Yeast MATa deletion collection |  | http://www-sequence.stanford.edu/group/yeast_deletion_project/deletions3.html |
| strain, strain background (*S. cerevisiae*) | yil119cΔ | YSC1053 Yeast MATa deletion collection |  | http://www-sequence.stanford.edu/group/yeast_deletion_project/deletions3.html |
| strain, strain background (*S. cerevisiae*) | yil122wΔ | YSC1053 Yeast MATa deletion collection |  | http://www-sequence.stanford.edu/group/yeast_deletion_project/deletions3.html |
| strain, strain background (*S. cerevisiae*) | yil123wΔ | YSC1053 Yeast MATa deletion collection |  | http://www-sequence.stanford.edu/group/yeast_deletion_project/deletions3.html |
| strain, strain background (*S. cerevisiae*) | yil124wΔ | YSC1053 Yeast MATa deletion collection |  | http://www-sequence.stanford.edu/group/yeast_deletion_project/deletions3.html |
| strain, strain background (*S. cerevisiae*) | yil128wΔ | YSC1053 Yeast MATa deletion collection |  | http://www-sequence.stanford.edu/group/yeast_deletion_project/deletions3.html |
| strain, strain background (*S. cerevisiae*) | yil130wΔ | YSC1053 Yeast MATa deletion collection |  | http://www-sequence.stanford.edu/group/yeast_deletion_project/deletions3.html |
| strain, strain background (*S. cerevisiae*) | yil133cΔ | YSC1053 Yeast MATa deletion collection |  | http://www-sequence.stanford.edu/group/yeast_deletion_project/deletions3.html |
| strain, strain background (*S. cerevisiae*) | yil134wΔ | YSC1053 Yeast MATa deletion collection |  | http://www-sequence.stanford.edu/group/yeast_deletion_project/deletions3.html |
| strain, strain background (*S. cerevisiae*) | yil136wΔ | YSC1053 Yeast MATa deletion collection |  | http://www-sequence.stanford.edu/group/yeast_deletion_project/deletions3.html |
| strain, strain background (*S. cerevisiae*) | yil137cΔ | YSC1053 Yeast MATa deletion collection |  | http://www-sequence.stanford.edu/group/yeast_deletion_project/deletions3.html |
| strain, strain background (*S. cerevisiae*) | yil139cΔ | YSC1053 Yeast MATa deletion collection |  | http://www-sequence.stanford.edu/group/yeast_deletion_project/deletions3.html |
| strain, strain background (*S. cerevisiae*) | yil146cΔ | YSC1053 Yeast MATa deletion collection |  | http://www-sequence.stanford.edu/group/yeast_deletion_project/deletions3.html |
| strain, strain background (*S. cerevisiae*) | yil148wΔ | YSC1053 Yeast MATa deletion collection |  | http://www-sequence.stanford.edu/group/yeast_deletion_project/deletions3.html |
| strain, strain background (*S. cerevisiae*) | yil153wΔ | YSC1053 Yeast MATa deletion collection |  | http://www-sequence.stanford.edu/group/yeast_deletion_project/deletions3.html |
| strain, strain background (*S. cerevisiae*) | yil157cΔ | YSC1053 Yeast MATa deletion collection |  | http://www-sequence.stanford.edu/group/yeast_deletion_project/deletions3.html |
| strain, strain background (*S. cerevisiae*) | yil158wΔ | YSC1053 Yeast MATa deletion collection |  | http://www-sequence.stanford.edu/group/yeast_deletion_project/deletions3.html |
| strain, strain background (*S. cerevisiae*) | yil159wΔ | YSC1053 Yeast MATa deletion collection |  | http://www-sequence.stanford.edu/group/yeast_deletion_project/deletions3.html |
| strain, strain background (*S. cerevisiae*) | yil160cΔ | YSC1053 Yeast MATa deletion collection |  | http://www-sequence.stanford.edu/group/yeast_deletion_project/deletions3.html |
| strain, strain background (*S. cerevisiae*) | yil161wΔ | YSC1053 Yeast MATa deletion collection |  | http://www-sequence.stanford.edu/group/yeast_deletion_project/deletions3.html |
| strain, strain background (*S. cerevisiae*) | yil165cΔ | YSC1053 Yeast MATa deletion collection |  | http://www-sequence.stanford.edu/group/yeast_deletion_project/deletions3.html |
| strain, strain background (*S. cerevisiae*) | yir005wΔ | YSC1053 Yeast MATa deletion collection |  | http://www-sequence.stanford.edu/group/yeast_deletion_project/deletions3.html |
| strain, strain background (*S. cerevisiae*) | yir009wΔ | YSC1053 Yeast MATa deletion collection |  | http://www-sequence.stanford.edu/group/yeast_deletion_project/deletions3.html |
| strain, strain background (*S. cerevisiae*) | yir018wΔ | YSC1053 Yeast MATa deletion collection |  | http://www-sequence.stanford.edu/group/yeast_deletion_project/deletions3.html |
| strain, strain background (*S. cerevisiae*) | yir021wΔ | YSC1053 Yeast MATa deletion collection |  | http://www-sequence.stanford.edu/group/yeast_deletion_project/deletions3.html |
| strain, strain background (*S. cerevisiae*) | yir023wΔ | YSC1053 Yeast MATa deletion collection |  | http://www-sequence.stanford.edu/group/yeast_deletion_project/deletions3.html |
| strain, strain background (*S. cerevisiae*) | yir026cΔ | YSC1053 Yeast MATa deletion collection |  | http://www-sequence.stanford.edu/group/yeast_deletion_project/deletions3.html |
| strain, strain background (*S. cerevisiae*) | yir033wΔ | YSC1053 Yeast MATa deletion collection |  | http://www-sequence.stanford.edu/group/yeast_deletion_project/deletions3.html |
| strain, strain background (*S. cerevisiae*) | yjl003wΔ | YSC1053 Yeast MATa deletion collection |  | http://www-sequence.stanford.edu/group/yeast_deletion_project/deletions3.html |
| strain, strain background (*S. cerevisiae*) | yjl013cΔ | YSC1053 Yeast MATa deletion collection |  | http://www-sequence.stanford.edu/group/yeast_deletion_project/deletions3.html |
| strain, strain background (*S. cerevisiae*) | yjl036wΔ | YSC1053 Yeast MATa deletion collection |  | http://www-sequence.stanford.edu/group/yeast_deletion_project/deletions3.html |
| strain, strain background (*S. cerevisiae*) | yjl047cΔ | YSC1053 Yeast MATa deletion collection |  | http://www-sequence.stanford.edu/group/yeast_deletion_project/deletions3.html |
| strain, strain background (*S. cerevisiae*) | yjl053wΔ | YSC1053 Yeast MATa deletion collection |  | http://www-sequence.stanford.edu/group/yeast_deletion_project/deletions3.html |
| strain, strain background (*S. cerevisiae*) | yjl056cΔ | YSC1053 Yeast MATa deletion collection |  | http://www-sequence.stanford.edu/group/yeast_deletion_project/deletions3.html |
| strain, strain background (*S. cerevisiae*) | yjl062w-aΔ | YSC1053 Yeast MATa deletion collection |  | http://www-sequence.stanford.edu/group/yeast_deletion_project/deletions3.html |
| strain, strain background (*S. cerevisiae*) | yjl063cΔ | YSC1053 Yeast MATa deletion collection |  | http://www-sequence.stanford.edu/group/yeast_deletion_project/deletions3.html |
| strain, strain background (*S. cerevisiae*) | yjl067wΔ | YSC1053 Yeast MATa deletion collection |  | http://www-sequence.stanford.edu/group/yeast_deletion_project/deletions3.html |
| strain, strain background (*S. cerevisiae*) | yjl071wΔ | YSC1053 Yeast MATa deletion collection |  | http://www-sequence.stanford.edu/group/yeast_deletion_project/deletions3.html |
| strain, strain background (*S. cerevisiae*) | yjl078cΔ | YSC1053 Yeast MATa deletion collection |  | http://www-sequence.stanford.edu/group/yeast_deletion_project/deletions3.html |
| strain, strain background (*S. cerevisiae*) | yjl096wΔ | YSC1053 Yeast MATa deletion collection |  | http://www-sequence.stanford.edu/group/yeast_deletion_project/deletions3.html |
| strain, strain background (*S. cerevisiae*) | yjl101cΔ | YSC1053 Yeast MATa deletion collection |  | http://www-sequence.stanford.edu/group/yeast_deletion_project/deletions3.html |
| strain, strain background (*S. cerevisiae*) | yjl102wΔ | YSC1053 Yeast MATa deletion collection |  | http://www-sequence.stanford.edu/group/yeast_deletion_project/deletions3.html |
| strain, strain background (*S. cerevisiae*) | yjl115wΔ | YSC1053 Yeast MATa deletion collection |  | http://www-sequence.stanford.edu/group/yeast_deletion_project/deletions3.html |
| strain, strain background (*S. cerevisiae*) | yjl117wΔ | YSC1053 Yeast MATa deletion collection |  | http://www-sequence.stanford.edu/group/yeast_deletion_project/deletions3.html |
| strain, strain background (*S. cerevisiae*) | yjl120wΔ | YSC1053 Yeast MATa deletion collection |  | http://www-sequence.stanford.edu/group/yeast_deletion_project/deletions3.html |
| strain, strain background (*S. cerevisiae*) | yjl121cΔ | YSC1053 Yeast MATa deletion collection |  | http://www-sequence.stanford.edu/group/yeast_deletion_project/deletions3.html |
| strain, strain background (*S. cerevisiae*) | yjl124cΔ | YSC1053 Yeast MATa deletion collection |  | http://www-sequence.stanford.edu/group/yeast_deletion_project/deletions3.html |
| strain, strain background (*S. cerevisiae*) | yjl127cΔ | YSC1053 Yeast MATa deletion collection |  | http://www-sequence.stanford.edu/group/yeast_deletion_project/deletions3.html |
| strain, strain background (*S. cerevisiae*) | yjl129cΔ | YSC1053 Yeast MATa deletion collection |  | http://www-sequence.stanford.edu/group/yeast_deletion_project/deletions3.html |
| strain, strain background (*S. cerevisiae*) | yjl130cΔ | YSC1053 Yeast MATa deletion collection |  | http://www-sequence.stanford.edu/group/yeast_deletion_project/deletions3.html |
| strain, strain background (*S. cerevisiae*) | yjl131cΔ | YSC1053 Yeast MATa deletion collection |  | http://www-sequence.stanford.edu/group/yeast_deletion_project/deletions3.html |
| strain, strain background (*S. cerevisiae*) | yjl133wΔ | YSC1053 Yeast MATa deletion collection |  | http://www-sequence.stanford.edu/group/yeast_deletion_project/deletions3.html |
| strain, strain background (*S. cerevisiae*) | yjl134wΔ | YSC1053 Yeast MATa deletion collection |  | http://www-sequence.stanford.edu/group/yeast_deletion_project/deletions3.html |
| strain, strain background (*S. cerevisiae*) | yjl135wΔ | YSC1053 Yeast MATa deletion collection |  | http://www-sequence.stanford.edu/group/yeast_deletion_project/deletions3.html |
| strain, strain background (*S. cerevisiae*) | yjl136cΔ | YSC1053 Yeast MATa deletion collection |  | http://www-sequence.stanford.edu/group/yeast_deletion_project/deletions3.html |
| strain, strain background (*S. cerevisiae*) | yjl138cΔ | YSC1053 Yeast MATa deletion collection |  | http://www-sequence.stanford.edu/group/yeast_deletion_project/deletions3.html |
| strain, strain background (*S. cerevisiae*) | yjl140wΔ | YSC1053 Yeast MATa deletion collection |  | http://www-sequence.stanford.edu/group/yeast_deletion_project/deletions3.html |
| strain, strain background (*S. cerevisiae*) | yjl141cΔ | YSC1053 Yeast MATa deletion collection |  | http://www-sequence.stanford.edu/group/yeast_deletion_project/deletions3.html |
| strain, strain background (*S. cerevisiae*) | yjl144wΔ | YSC1053 Yeast MATa deletion collection |  | http://www-sequence.stanford.edu/group/yeast_deletion_project/deletions3.html |
| strain, strain background (*S. cerevisiae*) | yjl146wΔ | YSC1053 Yeast MATa deletion collection |  | http://www-sequence.stanford.edu/group/yeast_deletion_project/deletions3.html |
| strain, strain background (*S. cerevisiae*) | yjl148wΔ | YSC1053 Yeast MATa deletion collection |  | http://www-sequence.stanford.edu/group/yeast_deletion_project/deletions3.html |
| strain, strain background (*S. cerevisiae*) | yjl150wΔ | YSC1053 Yeast MATa deletion collection |  | http://www-sequence.stanford.edu/group/yeast_deletion_project/deletions3.html |
| strain, strain background (*S. cerevisiae*) | yjl154cΔ | YSC1053 Yeast MATa deletion collection |  | http://www-sequence.stanford.edu/group/yeast_deletion_project/deletions3.html |
| strain, strain background (*S. cerevisiae*) | yjl157cΔ | YSC1053 Yeast MATa deletion collection |  | http://www-sequence.stanford.edu/group/yeast_deletion_project/deletions3.html |
| strain, strain background (*S. cerevisiae*) | yjl158cΔ | YSC1053 Yeast MATa deletion collection |  | http://www-sequence.stanford.edu/group/yeast_deletion_project/deletions3.html |
| strain, strain background (*S. cerevisiae*) | yjl162cΔ | YSC1053 Yeast MATa deletion collection |  | http://www-sequence.stanford.edu/group/yeast_deletion_project/deletions3.html |
| strain, strain background (*S. cerevisiae*) | yjl164cΔ | YSC1053 Yeast MATa deletion collection |  | http://www-sequence.stanford.edu/group/yeast_deletion_project/deletions3.html |
| strain, strain background (*S. cerevisiae*) | yjl166wΔ | YSC1053 Yeast MATa deletion collection |  | http://www-sequence.stanford.edu/group/yeast_deletion_project/deletions3.html |
| strain, strain background (*S. cerevisiae*) | yjl168cΔ | YSC1053 Yeast MATa deletion collection |  | http://www-sequence.stanford.edu/group/yeast_deletion_project/deletions3.html |
| strain, strain background (*S. cerevisiae*) | yjl177wΔ | YSC1053 Yeast MATa deletion collection |  | http://www-sequence.stanford.edu/group/yeast_deletion_project/deletions3.html |
| strain, strain background (*S. cerevisiae*) | yjl180cΔ | YSC1053 Yeast MATa deletion collection |  | http://www-sequence.stanford.edu/group/yeast_deletion_project/deletions3.html |
| strain, strain background (*S. cerevisiae*) | yjl184wΔ | YSC1053 Yeast MATa deletion collection |  | http://www-sequence.stanford.edu/group/yeast_deletion_project/deletions3.html |
| strain, strain background (*S. cerevisiae*) | yjl185cΔ | YSC1053 Yeast MATa deletion collection |  | http://www-sequence.stanford.edu/group/yeast_deletion_project/deletions3.html |
| strain, strain background (*S. cerevisiae*) | yjl186wΔ | YSC1053 Yeast MATa deletion collection |  | http://www-sequence.stanford.edu/group/yeast_deletion_project/deletions3.html |
| strain, strain background (*S. cerevisiae*) | yjl187cΔ | YSC1053 Yeast MATa deletion collection |  | http://www-sequence.stanford.edu/group/yeast_deletion_project/deletions3.html |
| strain, strain background (*S. cerevisiae*) | yjl188cΔ | YSC1053 Yeast MATa deletion collection |  | http://www-sequence.stanford.edu/group/yeast_deletion_project/deletions3.html |
| strain, strain background (*S. cerevisiae*) | yjl189wΔ | YSC1053 Yeast MATa deletion collection |  | http://www-sequence.stanford.edu/group/yeast_deletion_project/deletions3.html |
| strain, strain background (*S. cerevisiae*) | yjl190cΔ | YSC1053 Yeast MATa deletion collection |  | http://www-sequence.stanford.edu/group/yeast_deletion_project/deletions3.html |
| strain, strain background (*S. cerevisiae*) | yjl199cΔ | YSC1053 Yeast MATa deletion collection |  | http://www-sequence.stanford.edu/group/yeast_deletion_project/deletions3.html |
| strain, strain background (*S. cerevisiae*) | yjl200cΔ | YSC1053 Yeast MATa deletion collection |  | http://www-sequence.stanford.edu/group/yeast_deletion_project/deletions3.html |
| strain, strain background (*S. cerevisiae*) | yjl204cΔ | YSC1053 Yeast MATa deletion collection |  | http://www-sequence.stanford.edu/group/yeast_deletion_project/deletions3.html |
| strain, strain background (*S. cerevisiae*) | yjl209wΔ | YSC1053 Yeast MATa deletion collection |  | http://www-sequence.stanford.edu/group/yeast_deletion_project/deletions3.html |
| strain, strain background (*S. cerevisiae*) | yjl210wΔ | YSC1053 Yeast MATa deletion collection |  | http://www-sequence.stanford.edu/group/yeast_deletion_project/deletions3.html |
| strain, strain background (*S. cerevisiae*) | yjl211cΔ | YSC1053 Yeast MATa deletion collection |  | http://www-sequence.stanford.edu/group/yeast_deletion_project/deletions3.html |
| strain, strain background (*S. cerevisiae*) | yjl215cΔ | YSC1053 Yeast MATa deletion collection |  | http://www-sequence.stanford.edu/group/yeast_deletion_project/deletions3.html |
| strain, strain background (*S. cerevisiae*) | yjl217wΔ | YSC1053 Yeast MATa deletion collection |  | http://www-sequence.stanford.edu/group/yeast_deletion_project/deletions3.html |
| strain, strain background (*S. cerevisiae*) | yjl218wΔ | YSC1053 Yeast MATa deletion collection |  | http://www-sequence.stanford.edu/group/yeast_deletion_project/deletions3.html |
| strain, strain background (*S. cerevisiae*) | yjr009cΔ | YSC1053 Yeast MATa deletion collection |  | http://www-sequence.stanford.edu/group/yeast_deletion_project/deletions3.html |
| strain, strain background (*S. cerevisiae*) | yjr010wΔ | YSC1053 Yeast MATa deletion collection |  | http://www-sequence.stanford.edu/group/yeast_deletion_project/deletions3.html |
| strain, strain background (*S. cerevisiae*) | yjr014wΔ | YSC1053 Yeast MATa deletion collection |  | http://www-sequence.stanford.edu/group/yeast_deletion_project/deletions3.html |
| strain, strain background (*S. cerevisiae*) | yjr032wΔ | YSC1053 Yeast MATa deletion collection |  | http://www-sequence.stanford.edu/group/yeast_deletion_project/deletions3.html |
| strain, strain background (*S. cerevisiae*) | yjr034wΔ | YSC1053 Yeast MATa deletion collection |  | http://www-sequence.stanford.edu/group/yeast_deletion_project/deletions3.html |
| strain, strain background (*S. cerevisiae*) | yjr037wΔ | YSC1053 Yeast MATa deletion collection |  | http://www-sequence.stanford.edu/group/yeast_deletion_project/deletions3.html |
| strain, strain background (*S. cerevisiae*) | yjr043cΔ | YSC1053 Yeast MATa deletion collection |  | http://www-sequence.stanford.edu/group/yeast_deletion_project/deletions3.html |
| strain, strain background (*S. cerevisiae*) | yjr044cΔ | YSC1053 Yeast MATa deletion collection |  | http://www-sequence.stanford.edu/group/yeast_deletion_project/deletions3.html |
| strain, strain background (*S. cerevisiae*) | yjr054wΔ | YSC1053 Yeast MATa deletion collection |  | http://www-sequence.stanford.edu/group/yeast_deletion_project/deletions3.html |
| strain, strain background (*S. cerevisiae*) | yjr055wΔ | YSC1053 Yeast MATa deletion collection |  | http://www-sequence.stanford.edu/group/yeast_deletion_project/deletions3.html |
| strain, strain background (*S. cerevisiae*) | yjr059wΔ | YSC1053 Yeast MATa deletion collection |  | http://www-sequence.stanford.edu/group/yeast_deletion_project/deletions3.html |
| strain, strain background (*S. cerevisiae*) | yjr060wΔ | YSC1053 Yeast MATa deletion collection |  | http://www-sequence.stanford.edu/group/yeast_deletion_project/deletions3.html |
| strain, strain background (*S. cerevisiae*) | yjr061wΔ | YSC1053 Yeast MATa deletion collection |  | http://www-sequence.stanford.edu/group/yeast_deletion_project/deletions3.html |
| strain, strain background (*S. cerevisiae*) | yjr070cΔ | YSC1053 Yeast MATa deletion collection |  | http://www-sequence.stanford.edu/group/yeast_deletion_project/deletions3.html |
| strain, strain background (*S. cerevisiae*) | yjr073cΔ | YSC1053 Yeast MATa deletion collection |  | http://www-sequence.stanford.edu/group/yeast_deletion_project/deletions3.html |
| strain, strain background (*S. cerevisiae*) | yjr074wΔ | YSC1053 Yeast MATa deletion collection |  | http://www-sequence.stanford.edu/group/yeast_deletion_project/deletions3.html |
| strain, strain background (*S. cerevisiae*) | yjr082cΔ | YSC1053 Yeast MATa deletion collection |  | http://www-sequence.stanford.edu/group/yeast_deletion_project/deletions3.html |
| strain, strain background (*S. cerevisiae*) | yjr084wΔ | YSC1053 Yeast MATa deletion collection |  | http://www-sequence.stanford.edu/group/yeast_deletion_project/deletions3.html |
| strain, strain background (*S. cerevisiae*) | yjr086wΔ | YSC1053 Yeast MATa deletion collection |  | http://www-sequence.stanford.edu/group/yeast_deletion_project/deletions3.html |
| strain, strain background (*S. cerevisiae*) | yjr087wΔ | YSC1053 Yeast MATa deletion collection |  | http://www-sequence.stanford.edu/group/yeast_deletion_project/deletions3.html |
| strain, strain background (*S. cerevisiae*) | yjr090cΔ | YSC1053 Yeast MATa deletion collection |  | http://www-sequence.stanford.edu/group/yeast_deletion_project/deletions3.html |
| strain, strain background (*S. cerevisiae*) | yjr094w-aΔ | YSC1053 Yeast MATa deletion collection |  | http://www-sequence.stanford.edu/group/yeast_deletion_project/deletions3.html |
| strain, strain background (*S. cerevisiae*) | yjr102cΔ | YSC1053 Yeast MATa deletion collection |  | http://www-sequence.stanford.edu/group/yeast_deletion_project/deletions3.html |
| strain, strain background (*S. cerevisiae*) | yjr104cΔ | YSC1053 Yeast MATa deletion collection |  | http://www-sequence.stanford.edu/group/yeast_deletion_project/deletions3.html |
| strain, strain background (*S. cerevisiae*) | yjr105wΔ | YSC1053 Yeast MATa deletion collection |  | http://www-sequence.stanford.edu/group/yeast_deletion_project/deletions3.html |
| strain, strain background (*S. cerevisiae*) | yjr113cΔ | YSC1053 Yeast MATa deletion collection |  | http://www-sequence.stanford.edu/group/yeast_deletion_project/deletions3.html |
| strain, strain background (*S. cerevisiae*) | yjr117wΔ | YSC1053 Yeast MATa deletion collection |  | http://www-sequence.stanford.edu/group/yeast_deletion_project/deletions3.html |
| strain, strain background (*S. cerevisiae*) | yjr118cΔ | YSC1053 Yeast MATa deletion collection |  | http://www-sequence.stanford.edu/group/yeast_deletion_project/deletions3.html |
| strain, strain background (*S. cerevisiae*) | yjr121wΔ | YSC1053 Yeast MATa deletion collection |  | http://www-sequence.stanford.edu/group/yeast_deletion_project/deletions3.html |
| strain, strain background (*S. cerevisiae*) | yjr126cΔ | YSC1053 Yeast MATa deletion collection |  | http://www-sequence.stanford.edu/group/yeast_deletion_project/deletions3.html |
| strain, strain background (*S. cerevisiae*) | yjr128wΔ | YSC1053 Yeast MATa deletion collection |  | http://www-sequence.stanford.edu/group/yeast_deletion_project/deletions3.html |
| strain, strain background (*S. cerevisiae*) | yjr139cΔ | YSC1053 Yeast MATa deletion collection |  | http://www-sequence.stanford.edu/group/yeast_deletion_project/deletions3.html |
| strain, strain background (*S. cerevisiae*) | yjr144wΔ | YSC1053 Yeast MATa deletion collection |  | http://www-sequence.stanford.edu/group/yeast_deletion_project/deletions3.html |
| strain, strain background (*S. cerevisiae*) | yjr145cΔ | YSC1053 Yeast MATa deletion collection |  | http://www-sequence.stanford.edu/group/yeast_deletion_project/deletions3.html |
| strain, strain background (*S. cerevisiae*) | ykl002wΔ | YSC1053 Yeast MATa deletion collection |  | http://www-sequence.stanford.edu/group/yeast_deletion_project/deletions3.html |
| strain, strain background (*S. cerevisiae*) | ykl003cΔ | YSC1053 Yeast MATa deletion collection |  | http://www-sequence.stanford.edu/group/yeast_deletion_project/deletions3.html |
| strain, strain background (*S. cerevisiae*) | ykl006wΔ | YSC1053 Yeast MATa deletion collection |  | http://www-sequence.stanford.edu/group/yeast_deletion_project/deletions3.html |
| strain, strain background (*S. cerevisiae*) | ykl009wΔ | YSC1053 Yeast MATa deletion collection |  | http://www-sequence.stanford.edu/group/yeast_deletion_project/deletions3.html |
| strain, strain background (*S. cerevisiae*) | ykl011cΔ | YSC1053 Yeast MATa deletion collection |  | http://www-sequence.stanford.edu/group/yeast_deletion_project/deletions3.html |
| strain, strain background (*S. cerevisiae*) | ykl015wΔ | YSC1053 Yeast MATa deletion collection |  | http://www-sequence.stanford.edu/group/yeast_deletion_project/deletions3.html |
| strain, strain background (*S. cerevisiae*) | ykl016cΔ | YSC1053 Yeast MATa deletion collection |  | http://www-sequence.stanford.edu/group/yeast_deletion_project/deletions3.html |
| strain, strain background (*S. cerevisiae*) | ykl023wΔ | YSC1053 Yeast MATa deletion collection |  | http://www-sequence.stanford.edu/group/yeast_deletion_project/deletions3.html |
| strain, strain background (*S. cerevisiae*) | ykl025cΔ | YSC1053 Yeast MATa deletion collection |  | http://www-sequence.stanford.edu/group/yeast_deletion_project/deletions3.html |
| strain, strain background (*S. cerevisiae*) | ykl032cΔ | YSC1053 Yeast MATa deletion collection |  | http://www-sequence.stanford.edu/group/yeast_deletion_project/deletions3.html |
| strain, strain background (*S. cerevisiae*) | ykl034wΔ | YSC1053 Yeast MATa deletion collection |  | http://www-sequence.stanford.edu/group/yeast_deletion_project/deletions3.html |
| strain, strain background (*S. cerevisiae*) | ykl037wΔ | YSC1053 Yeast MATa deletion collection |  | http://www-sequence.stanford.edu/group/yeast_deletion_project/deletions3.html |
| strain, strain background (*S. cerevisiae*) | ykl041wΔ | YSC1053 Yeast MATa deletion collection |  | http://www-sequence.stanford.edu/group/yeast_deletion_project/deletions3.html |
| strain, strain background (*S. cerevisiae*) | ykl048cΔ | YSC1053 Yeast MATa deletion collection |  | http://www-sequence.stanford.edu/group/yeast_deletion_project/deletions3.html |
| strain, strain background (*S. cerevisiae*) | ykl053wΔ | YSC1053 Yeast MATa deletion collection |  | http://www-sequence.stanford.edu/group/yeast_deletion_project/deletions3.html |
| strain, strain background (*S. cerevisiae*) | ykl054cΔ | YSC1053 Yeast MATa deletion collection |  | http://www-sequence.stanford.edu/group/yeast_deletion_project/deletions3.html |
| strain, strain background (*S. cerevisiae*) | ykl055cΔ | YSC1053 Yeast MATa deletion collection |  | http://www-sequence.stanford.edu/group/yeast_deletion_project/deletions3.html |
| strain, strain background (*S. cerevisiae*) | ykl056cΔ | YSC1053 Yeast MATa deletion collection |  | http://www-sequence.stanford.edu/group/yeast_deletion_project/deletions3.html |
| strain, strain background (*S. cerevisiae*) | ykl057cΔ | YSC1053 Yeast MATa deletion collection |  | http://www-sequence.stanford.edu/group/yeast_deletion_project/deletions3.html |
| strain, strain background (*S. cerevisiae*) | ykl062wΔ | YSC1053 Yeast MATa deletion collection |  | http://www-sequence.stanford.edu/group/yeast_deletion_project/deletions3.html |
| strain, strain background (*S. cerevisiae*) | ykl064wΔ | YSC1053 Yeast MATa deletion collection |  | http://www-sequence.stanford.edu/group/yeast_deletion_project/deletions3.html |
| strain, strain background (*S. cerevisiae*) | ykl069wΔ | YSC1053 Yeast MATa deletion collection |  | http://www-sequence.stanford.edu/group/yeast_deletion_project/deletions3.html |
| strain, strain background (*S. cerevisiae*) | ykl073wΔ | YSC1053 Yeast MATa deletion collection |  | http://www-sequence.stanford.edu/group/yeast_deletion_project/deletions3.html |
| strain, strain background (*S. cerevisiae*) | ykl074cΔ | YSC1053 Yeast MATa deletion collection |  | http://www-sequence.stanford.edu/group/yeast_deletion_project/deletions3.html |
| strain, strain background (*S. cerevisiae*) | ykl075cΔ | YSC1053 Yeast MATa deletion collection |  | http://www-sequence.stanford.edu/group/yeast_deletion_project/deletions3.html |
| strain, strain background (*S. cerevisiae*) | ykl076cΔ | YSC1053 Yeast MATa deletion collection |  | http://www-sequence.stanford.edu/group/yeast_deletion_project/deletions3.html |
| strain, strain background (*S. cerevisiae*) | ykl080wΔ | YSC1053 Yeast MATa deletion collection |  | http://www-sequence.stanford.edu/group/yeast_deletion_project/deletions3.html |
| strain, strain background (*S. cerevisiae*) | ykl081wΔ | YSC1053 Yeast MATa deletion collection |  | http://www-sequence.stanford.edu/group/yeast_deletion_project/deletions3.html |
| strain, strain background (*S. cerevisiae*) | ykl087cΔ | YSC1053 Yeast MATa deletion collection |  | http://www-sequence.stanford.edu/group/yeast_deletion_project/deletions3.html |
| strain, strain background (*S. cerevisiae*) | ykl101wΔ | YSC1053 Yeast MATa deletion collection |  | http://www-sequence.stanford.edu/group/yeast_deletion_project/deletions3.html |
| strain, strain background (*S. cerevisiae*) | ykl106wΔ | YSC1053 Yeast MATa deletion collection |  | http://www-sequence.stanford.edu/group/yeast_deletion_project/deletions3.html |
| strain, strain background (*S. cerevisiae*) | ykl109wΔ | YSC1053 Yeast MATa deletion collection |  | http://www-sequence.stanford.edu/group/yeast_deletion_project/deletions3.html |
| strain, strain background (*S. cerevisiae*) | ykl110cΔ | YSC1053 Yeast MATa deletion collection |  | http://www-sequence.stanford.edu/group/yeast_deletion_project/deletions3.html |
| strain, strain background (*S. cerevisiae*) | ykl113cΔ | YSC1053 Yeast MATa deletion collection |  | http://www-sequence.stanford.edu/group/yeast_deletion_project/deletions3.html |
| strain, strain background (*S. cerevisiae*) | ykl118wΔ | YSC1053 Yeast MATa deletion collection |  | http://www-sequence.stanford.edu/group/yeast_deletion_project/deletions3.html |
| strain, strain background (*S. cerevisiae*) | ykl119cΔ | YSC1053 Yeast MATa deletion collection |  | http://www-sequence.stanford.edu/group/yeast_deletion_project/deletions3.html |
| strain, strain background (*S. cerevisiae*) | ykl134cΔ | YSC1053 Yeast MATa deletion collection |  | http://www-sequence.stanford.edu/group/yeast_deletion_project/deletions3.html |
| strain, strain background (*S. cerevisiae*) | ykl135cΔ | YSC1053 Yeast MATa deletion collection |  | http://www-sequence.stanford.edu/group/yeast_deletion_project/deletions3.html |
| strain, strain background (*S. cerevisiae*) | ykl137wΔ | YSC1053 Yeast MATa deletion collection |  | http://www-sequence.stanford.edu/group/yeast_deletion_project/deletions3.html |
| strain, strain background (*S. cerevisiae*) | ykl138cΔ | YSC1053 Yeast MATa deletion collection |  | http://www-sequence.stanford.edu/group/yeast_deletion_project/deletions3.html |
| strain, strain background (*S. cerevisiae*) | ykl139wΔ | YSC1053 Yeast MATa deletion collection |  | http://www-sequence.stanford.edu/group/yeast_deletion_project/deletions3.html |
| strain, strain background (*S. cerevisiae*) | ykl155cΔ | YSC1053 Yeast MATa deletion collection |  | http://www-sequence.stanford.edu/group/yeast_deletion_project/deletions3.html |
| strain, strain background (*S. cerevisiae*) | ykl160wΔ | YSC1053 Yeast MATa deletion collection |  | http://www-sequence.stanford.edu/group/yeast_deletion_project/deletions3.html |
| strain, strain background (*S. cerevisiae*) | ykl170wΔ | YSC1053 Yeast MATa deletion collection |  | http://www-sequence.stanford.edu/group/yeast_deletion_project/deletions3.html |
| strain, strain background (*S. cerevisiae*) | ykl176cΔ | YSC1053 Yeast MATa deletion collection |  | http://www-sequence.stanford.edu/group/yeast_deletion_project/deletions3.html |
| strain, strain background (*S. cerevisiae*) | ykl178cΔ | YSC1053 Yeast MATa deletion collection |  | http://www-sequence.stanford.edu/group/yeast_deletion_project/deletions3.html |
| strain, strain background (*S. cerevisiae*) | ykl179cΔ | YSC1053 Yeast MATa deletion collection |  | http://www-sequence.stanford.edu/group/yeast_deletion_project/deletions3.html |
| strain, strain background (*S. cerevisiae*) | ykl184wΔ | YSC1053 Yeast MATa deletion collection |  | http://www-sequence.stanford.edu/group/yeast_deletion_project/deletions3.html |
| strain, strain background (*S. cerevisiae*) | ykl194cΔ | YSC1053 Yeast MATa deletion collection |  | http://www-sequence.stanford.edu/group/yeast_deletion_project/deletions3.html |
| strain, strain background (*S. cerevisiae*) | ykl197cΔ | YSC1053 Yeast MATa deletion collection |  | http://www-sequence.stanford.edu/group/yeast_deletion_project/deletions3.html |
| strain, strain background (*S. cerevisiae*) | ykl204wΔ | YSC1053 Yeast MATa deletion collection |  | http://www-sequence.stanford.edu/group/yeast_deletion_project/deletions3.html |
| strain, strain background (*S. cerevisiae*) | ykl208wΔ | YSC1053 Yeast MATa deletion collection |  | http://www-sequence.stanford.edu/group/yeast_deletion_project/deletions3.html |
| strain, strain background (*S. cerevisiae*) | ykl212wΔ | YSC1053 Yeast MATa deletion collection |  | http://www-sequence.stanford.edu/group/yeast_deletion_project/deletions3.html |
| strain, strain background (*S. cerevisiae*) | ykl213cΔ | YSC1053 Yeast MATa deletion collection |  | http://www-sequence.stanford.edu/group/yeast_deletion_project/deletions3.html |
| strain, strain background (*S. cerevisiae*) | ykr006cΔ | YSC1053 Yeast MATa deletion collection |  | http://www-sequence.stanford.edu/group/yeast_deletion_project/deletions3.html |
| strain, strain background (*S. cerevisiae*) | ykr007wΔ | YSC1053 Yeast MATa deletion collection |  | http://www-sequence.stanford.edu/group/yeast_deletion_project/deletions3.html |
| strain, strain background (*S. cerevisiae*) | ykr009cΔ | YSC1053 Yeast MATa deletion collection |  | http://www-sequence.stanford.edu/group/yeast_deletion_project/deletions3.html |
| strain, strain background (*S. cerevisiae*) | ykr019cΔ | YSC1053 Yeast MATa deletion collection |  | http://www-sequence.stanford.edu/group/yeast_deletion_project/deletions3.html |
| strain, strain background (*S. cerevisiae*) | ykr020wΔ | YSC1053 Yeast MATa deletion collection |  | http://www-sequence.stanford.edu/group/yeast_deletion_project/deletions3.html |
| strain, strain background (*S. cerevisiae*) | ykr024cΔ | YSC1053 Yeast MATa deletion collection |  | http://www-sequence.stanford.edu/group/yeast_deletion_project/deletions3.html |
| strain, strain background (*S. cerevisiae*) | ykr027wΔ | YSC1053 Yeast MATa deletion collection |  | http://www-sequence.stanford.edu/group/yeast_deletion_project/deletions3.html |
| strain, strain background (*S. cerevisiae*) | ykr035cΔ | YSC1053 Yeast MATa deletion collection |  | http://www-sequence.stanford.edu/group/yeast_deletion_project/deletions3.html |
| strain, strain background (*S. cerevisiae*) | ykr042wΔ | YSC1053 Yeast MATa deletion collection |  | http://www-sequence.stanford.edu/group/yeast_deletion_project/deletions3.html |
| strain, strain background (*S. cerevisiae*) | ykr047wΔ | YSC1053 Yeast MATa deletion collection |  | http://www-sequence.stanford.edu/group/yeast_deletion_project/deletions3.html |
| strain, strain background (*S. cerevisiae*) | ykr051wΔ | YSC1053 Yeast MATa deletion collection |  | http://www-sequence.stanford.edu/group/yeast_deletion_project/deletions3.html |
| strain, strain background (*S. cerevisiae*) | ykr052cΔ | YSC1053 Yeast MATa deletion collection |  | http://www-sequence.stanford.edu/group/yeast_deletion_project/deletions3.html |
| strain, strain background (*S. cerevisiae*) | ykr054cΔ | YSC1053 Yeast MATa deletion collection |  | http://www-sequence.stanford.edu/group/yeast_deletion_project/deletions3.html |
| strain, strain background (*S. cerevisiae*) | ykr057wΔ | YSC1053 Yeast MATa deletion collection |  | http://www-sequence.stanford.edu/group/yeast_deletion_project/deletions3.html |
| strain, strain background (*S. cerevisiae*) | ykr059wΔ | YSC1053 Yeast MATa deletion collection |  | http://www-sequence.stanford.edu/group/yeast_deletion_project/deletions3.html |
| strain, strain background (*S. cerevisiae*) | ykr062wΔ | YSC1053 Yeast MATa deletion collection |  | http://www-sequence.stanford.edu/group/yeast_deletion_project/deletions3.html |
| strain, strain background (*S. cerevisiae*) | ykr065cΔ | YSC1053 Yeast MATa deletion collection |  | http://www-sequence.stanford.edu/group/yeast_deletion_project/deletions3.html |
| strain, strain background (*S. cerevisiae*) | ykr072cΔ | YSC1053 Yeast MATa deletion collection |  | http://www-sequence.stanford.edu/group/yeast_deletion_project/deletions3.html |
| strain, strain background (*S. cerevisiae*) | ykr074wΔ | YSC1053 Yeast MATa deletion collection |  | http://www-sequence.stanford.edu/group/yeast_deletion_project/deletions3.html |
| strain, strain background (*S. cerevisiae*) | ykr082wΔ | YSC1053 Yeast MATa deletion collection |  | http://www-sequence.stanford.edu/group/yeast_deletion_project/deletions3.html |
| strain, strain background (*S. cerevisiae*) | ykr084cΔ | YSC1053 Yeast MATa deletion collection |  | http://www-sequence.stanford.edu/group/yeast_deletion_project/deletions3.html |
| strain, strain background (*S. cerevisiae*) | ykr085cΔ | YSC1053 Yeast MATa deletion collection |  | http://www-sequence.stanford.edu/group/yeast_deletion_project/deletions3.html |
| strain, strain background (*S. cerevisiae*) | ykr092cΔ | YSC1053 Yeast MATa deletion collection |  | http://www-sequence.stanford.edu/group/yeast_deletion_project/deletions3.html |
| strain, strain background (*S. cerevisiae*) | ykr094cΔ | YSC1053 Yeast MATa deletion collection |  | http://www-sequence.stanford.edu/group/yeast_deletion_project/deletions3.html |
| strain, strain background (*S. cerevisiae*) | ykr095wΔ | YSC1053 Yeast MATa deletion collection |  | http://www-sequence.stanford.edu/group/yeast_deletion_project/deletions3.html |
| strain, strain background (*S. cerevisiae*) | ykr101wΔ | YSC1053 Yeast MATa deletion collection |  | http://www-sequence.stanford.edu/group/yeast_deletion_project/deletions3.html |
| strain, strain background (*S. cerevisiae*) | yll002wΔ | YSC1053 Yeast MATa deletion collection |  | http://www-sequence.stanford.edu/group/yeast_deletion_project/deletions3.html |
| strain, strain background (*S. cerevisiae*) | yll006wΔ | YSC1053 Yeast MATa deletion collection |  | http://www-sequence.stanford.edu/group/yeast_deletion_project/deletions3.html |
| strain, strain background (*S. cerevisiae*) | yll007cΔ | YSC1053 Yeast MATa deletion collection |  | http://www-sequence.stanford.edu/group/yeast_deletion_project/deletions3.html |
| strain, strain background (*S. cerevisiae*) | yll009cΔ | YSC1053 Yeast MATa deletion collection |  | http://www-sequence.stanford.edu/group/yeast_deletion_project/deletions3.html |
| strain, strain background (*S. cerevisiae*) | yll010cΔ | YSC1053 Yeast MATa deletion collection |  | http://www-sequence.stanford.edu/group/yeast_deletion_project/deletions3.html |
| strain, strain background (*S. cerevisiae*) | yll013cΔ | YSC1053 Yeast MATa deletion collection |  | http://www-sequence.stanford.edu/group/yeast_deletion_project/deletions3.html |
| strain, strain background (*S. cerevisiae*) | yll018c-aΔ | YSC1053 Yeast MATa deletion collection |  | http://www-sequence.stanford.edu/group/yeast_deletion_project/deletions3.html |
| strain, strain background (*S. cerevisiae*) | yll020cΔ | YSC1053 Yeast MATa deletion collection |  | http://www-sequence.stanford.edu/group/yeast_deletion_project/deletions3.html |
| strain, strain background (*S. cerevisiae*) | yll027wΔ | YSC1053 Yeast MATa deletion collection |  | http://www-sequence.stanford.edu/group/yeast_deletion_project/deletions3.html |
| strain, strain background (*S. cerevisiae*) | yll029wΔ | YSC1053 Yeast MATa deletion collection |  | http://www-sequence.stanford.edu/group/yeast_deletion_project/deletions3.html |
| strain, strain background (*S. cerevisiae*) | yll033wΔ | YSC1053 Yeast MATa deletion collection |  | http://www-sequence.stanford.edu/group/yeast_deletion_project/deletions3.html |
| strain, strain background (*S. cerevisiae*) | yll038cΔ | YSC1053 Yeast MATa deletion collection |  | http://www-sequence.stanford.edu/group/yeast_deletion_project/deletions3.html |
| strain, strain background (*S. cerevisiae*) | yll039cΔ | YSC1053 Yeast MATa deletion collection |  | http://www-sequence.stanford.edu/group/yeast_deletion_project/deletions3.html |
| strain, strain background (*S. cerevisiae*) | yll040cΔ | YSC1053 Yeast MATa deletion collection |  | http://www-sequence.stanford.edu/group/yeast_deletion_project/deletions3.html |
| strain, strain background (*S. cerevisiae*) | yll043wΔ | YSC1053 Yeast MATa deletion collection |  | http://www-sequence.stanford.edu/group/yeast_deletion_project/deletions3.html |
| strain, strain background (*S. cerevisiae*) | yll045cΔ | YSC1053 Yeast MATa deletion collection |  | http://www-sequence.stanford.edu/group/yeast_deletion_project/deletions3.html |
| strain, strain background (*S. cerevisiae*) | yll049wΔ | YSC1053 Yeast MATa deletion collection |  | http://www-sequence.stanford.edu/group/yeast_deletion_project/deletions3.html |
| strain, strain background (*S. cerevisiae*) | yll051cΔ | YSC1053 Yeast MATa deletion collection |  | http://www-sequence.stanford.edu/group/yeast_deletion_project/deletions3.html |
| strain, strain background (*S. cerevisiae*) | ylr006cΔ | YSC1053 Yeast MATa deletion collection |  | http://www-sequence.stanford.edu/group/yeast_deletion_project/deletions3.html |
| strain, strain background (*S. cerevisiae*) | ylr014cΔ | YSC1053 Yeast MATa deletion collection |  | http://www-sequence.stanford.edu/group/yeast_deletion_project/deletions3.html |
| strain, strain background (*S. cerevisiae*) | ylr015wΔ | YSC1053 Yeast MATa deletion collection |  | http://www-sequence.stanford.edu/group/yeast_deletion_project/deletions3.html |
| strain, strain background (*S. cerevisiae*) | ylr019wΔ | YSC1053 Yeast MATa deletion collection |  | http://www-sequence.stanford.edu/group/yeast_deletion_project/deletions3.html |
| strain, strain background (*S. cerevisiae*) | ylr021wΔ | YSC1053 Yeast MATa deletion collection |  | http://www-sequence.stanford.edu/group/yeast_deletion_project/deletions3.html |
| strain, strain background (*S. cerevisiae*) | ylr024cΔ | YSC1053 Yeast MATa deletion collection |  | http://www-sequence.stanford.edu/group/yeast_deletion_project/deletions3.html |
| strain, strain background (*S. cerevisiae*) | ylr025wΔ | YSC1053 Yeast MATa deletion collection |  | http://www-sequence.stanford.edu/group/yeast_deletion_project/deletions3.html |
| strain, strain background (*S. cerevisiae*) | ylr027cΔ | YSC1053 Yeast MATa deletion collection |  | http://www-sequence.stanford.edu/group/yeast_deletion_project/deletions3.html |
| strain, strain background (*S. cerevisiae*) | ylr032wΔ | YSC1053 Yeast MATa deletion collection |  | http://www-sequence.stanford.edu/group/yeast_deletion_project/deletions3.html |
| strain, strain background (*S. cerevisiae*) | ylr038cΔ | YSC1053 Yeast MATa deletion collection |  | http://www-sequence.stanford.edu/group/yeast_deletion_project/deletions3.html |
| strain, strain background (*S. cerevisiae*) | ylr039cΔ | YSC1053 Yeast MATa deletion collection |  | http://www-sequence.stanford.edu/group/yeast_deletion_project/deletions3.html |
| strain, strain background (*S. cerevisiae*) | ylr048wΔ | YSC1053 Yeast MATa deletion collection |  | http://www-sequence.stanford.edu/group/yeast_deletion_project/deletions3.html |
| strain, strain background (*S. cerevisiae*) | ylr052wΔ | YSC1053 Yeast MATa deletion collection |  | http://www-sequence.stanford.edu/group/yeast_deletion_project/deletions3.html |
| strain, strain background (*S. cerevisiae*) | ylr055cΔ | YSC1053 Yeast MATa deletion collection |  | http://www-sequence.stanford.edu/group/yeast_deletion_project/deletions3.html |
| strain, strain background (*S. cerevisiae*) | ylr056wΔ | YSC1053 Yeast MATa deletion collection |  | http://www-sequence.stanford.edu/group/yeast_deletion_project/deletions3.html |
| strain, strain background (*S. cerevisiae*) | ylr061wΔ | YSC1053 Yeast MATa deletion collection |  | http://www-sequence.stanford.edu/group/yeast_deletion_project/deletions3.html |
| strain, strain background (*S. cerevisiae*) | ylr062cΔ | YSC1053 Yeast MATa deletion collection |  | http://www-sequence.stanford.edu/group/yeast_deletion_project/deletions3.html |
| strain, strain background (*S. cerevisiae*) | ylr067cΔ | YSC1053 Yeast MATa deletion collection |  | http://www-sequence.stanford.edu/group/yeast_deletion_project/deletions3.html |
| strain, strain background (*S. cerevisiae*) | ylr068wΔ | YSC1053 Yeast MATa deletion collection |  | http://www-sequence.stanford.edu/group/yeast_deletion_project/deletions3.html |
| strain, strain background (*S. cerevisiae*) | ylr069cΔ | YSC1053 Yeast MATa deletion collection |  | http://www-sequence.stanford.edu/group/yeast_deletion_project/deletions3.html |
| strain, strain background (*S. cerevisiae*) | ylr079wΔ | YSC1053 Yeast MATa deletion collection |  | http://www-sequence.stanford.edu/group/yeast_deletion_project/deletions3.html |
| strain, strain background (*S. cerevisiae*) | ylr085cΔ | YSC1053 Yeast MATa deletion collection |  | http://www-sequence.stanford.edu/group/yeast_deletion_project/deletions3.html |
| strain, strain background (*S. cerevisiae*) | ylr087cΔ | YSC1053 Yeast MATa deletion collection |  | http://www-sequence.stanford.edu/group/yeast_deletion_project/deletions3.html |
| strain, strain background (*S. cerevisiae*) | ylr089cΔ | YSC1053 Yeast MATa deletion collection |  | http://www-sequence.stanford.edu/group/yeast_deletion_project/deletions3.html |
| strain, strain background (*S. cerevisiae*) | ylr091wΔ | YSC1053 Yeast MATa deletion collection |  | http://www-sequence.stanford.edu/group/yeast_deletion_project/deletions3.html |
| strain, strain background (*S. cerevisiae*) | ylr102cΔ | YSC1053 Yeast MATa deletion collection |  | http://www-sequence.stanford.edu/group/yeast_deletion_project/deletions3.html |
| strain, strain background (*S. cerevisiae*) | ylr104wΔ | YSC1053 Yeast MATa deletion collection |  | http://www-sequence.stanford.edu/group/yeast_deletion_project/deletions3.html |
| strain, strain background (*S. cerevisiae*) | ylr108cΔ | YSC1053 Yeast MATa deletion collection |  | http://www-sequence.stanford.edu/group/yeast_deletion_project/deletions3.html |
| strain, strain background (*S. cerevisiae*) | ylr110cΔ | YSC1053 Yeast MATa deletion collection |  | http://www-sequence.stanford.edu/group/yeast_deletion_project/deletions3.html |
| strain, strain background (*S. cerevisiae*) | ylr111wΔ | YSC1053 Yeast MATa deletion collection |  | http://www-sequence.stanford.edu/group/yeast_deletion_project/deletions3.html |
| strain, strain background (*S. cerevisiae*) | ylr113wΔ | YSC1053 Yeast MATa deletion collection |  | http://www-sequence.stanford.edu/group/yeast_deletion_project/deletions3.html |
| strain, strain background (*S. cerevisiae*) | ylr114cΔ | YSC1053 Yeast MATa deletion collection |  | http://www-sequence.stanford.edu/group/yeast_deletion_project/deletions3.html |
| strain, strain background (*S. cerevisiae*) | ylr119wΔ | YSC1053 Yeast MATa deletion collection |  | http://www-sequence.stanford.edu/group/yeast_deletion_project/deletions3.html |
| strain, strain background (*S. cerevisiae*) | ylr124wΔ | YSC1053 Yeast MATa deletion collection |  | http://www-sequence.stanford.edu/group/yeast_deletion_project/deletions3.html |
| strain, strain background (*S. cerevisiae*) | ylr136cΔ | YSC1053 Yeast MATa deletion collection |  | http://www-sequence.stanford.edu/group/yeast_deletion_project/deletions3.html |
| strain, strain background (*S. cerevisiae*) | ylr139cΔ | YSC1053 Yeast MATa deletion collection |  | http://www-sequence.stanford.edu/group/yeast_deletion_project/deletions3.html |
| strain, strain background (*S. cerevisiae*) | ylr148wΔ | YSC1053 Yeast MATa deletion collection |  | http://www-sequence.stanford.edu/group/yeast_deletion_project/deletions3.html |
| strain, strain background (*S. cerevisiae*) | ylr150wΔ | YSC1053 Yeast MATa deletion collection |  | http://www-sequence.stanford.edu/group/yeast_deletion_project/deletions3.html |
| strain, strain background (*S. cerevisiae*) | ylr151cΔ | YSC1053 Yeast MATa deletion collection |  | http://www-sequence.stanford.edu/group/yeast_deletion_project/deletions3.html |
| strain, strain background (*S. cerevisiae*) | ylr180wΔ | YSC1053 Yeast MATa deletion collection |  | http://www-sequence.stanford.edu/group/yeast_deletion_project/deletions3.html |
| strain, strain background (*S. cerevisiae*) | ylr182wΔ | YSC1053 Yeast MATa deletion collection |  | http://www-sequence.stanford.edu/group/yeast_deletion_project/deletions3.html |
| strain, strain background (*S. cerevisiae*) | ylr184wΔ | YSC1053 Yeast MATa deletion collection |  | http://www-sequence.stanford.edu/group/yeast_deletion_project/deletions3.html |
| strain, strain background (*S. cerevisiae*) | ylr185wΔ | YSC1053 Yeast MATa deletion collection |  | http://www-sequence.stanford.edu/group/yeast_deletion_project/deletions3.html |
| strain, strain background (*S. cerevisiae*) | ylr187wΔ | YSC1053 Yeast MATa deletion collection |  | http://www-sequence.stanford.edu/group/yeast_deletion_project/deletions3.html |
| strain, strain background (*S. cerevisiae*) | ylr190wΔ | YSC1053 Yeast MATa deletion collection |  | http://www-sequence.stanford.edu/group/yeast_deletion_project/deletions3.html |
| strain, strain background (*S. cerevisiae*) | ylr191wΔ | YSC1053 Yeast MATa deletion collection |  | http://www-sequence.stanford.edu/group/yeast_deletion_project/deletions3.html |
| strain, strain background (*S. cerevisiae*) | ylr192cΔ | YSC1053 Yeast MATa deletion collection |  | http://www-sequence.stanford.edu/group/yeast_deletion_project/deletions3.html |
| strain, strain background (*S. cerevisiae*) | ylr193cΔ | YSC1053 Yeast MATa deletion collection |  | http://www-sequence.stanford.edu/group/yeast_deletion_project/deletions3.html |
| strain, strain background (*S. cerevisiae*) | ylr200wΔ | YSC1053 Yeast MATa deletion collection |  | http://www-sequence.stanford.edu/group/yeast_deletion_project/deletions3.html |
| strain, strain background (*S. cerevisiae*) | ylr201cΔ | YSC1053 Yeast MATa deletion collection |  | http://www-sequence.stanford.edu/group/yeast_deletion_project/deletions3.html |
| strain, strain background (*S. cerevisiae*) | ylr202cΔ | YSC1053 Yeast MATa deletion collection |  | http://www-sequence.stanford.edu/group/yeast_deletion_project/deletions3.html |
| strain, strain background (*S. cerevisiae*) | ylr203cΔ | YSC1053 Yeast MATa deletion collection |  | http://www-sequence.stanford.edu/group/yeast_deletion_project/deletions3.html |
| strain, strain background (*S. cerevisiae*) | ylr204wΔ | YSC1053 Yeast MATa deletion collection |  | http://www-sequence.stanford.edu/group/yeast_deletion_project/deletions3.html |
| strain, strain background (*S. cerevisiae*) | ylr206wΔ | YSC1053 Yeast MATa deletion collection |  | http://www-sequence.stanford.edu/group/yeast_deletion_project/deletions3.html |
| strain, strain background (*S. cerevisiae*) | ylr207wΔ | YSC1053 Yeast MATa deletion collection |  | http://www-sequence.stanford.edu/group/yeast_deletion_project/deletions3.html |
| strain, strain background (*S. cerevisiae*) | ylr213cΔ | YSC1053 Yeast MATa deletion collection |  | http://www-sequence.stanford.edu/group/yeast_deletion_project/deletions3.html |
| strain, strain background (*S. cerevisiae*) | ylr218cΔ | YSC1053 Yeast MATa deletion collection |  | http://www-sequence.stanford.edu/group/yeast_deletion_project/deletions3.html |
| strain, strain background (*S. cerevisiae*) | ylr226wΔ | YSC1053 Yeast MATa deletion collection |  | http://www-sequence.stanford.edu/group/yeast_deletion_project/deletions3.html |
| strain, strain background (*S. cerevisiae*) | ylr233cΔ | YSC1053 Yeast MATa deletion collection |  | http://www-sequence.stanford.edu/group/yeast_deletion_project/deletions3.html |
| strain, strain background (*S. cerevisiae*) | ylr234wΔ | YSC1053 Yeast MATa deletion collection |  | http://www-sequence.stanford.edu/group/yeast_deletion_project/deletions3.html |
| strain, strain background (*S. cerevisiae*) | ylr235cΔ | YSC1053 Yeast MATa deletion collection |  | http://www-sequence.stanford.edu/group/yeast_deletion_project/deletions3.html |
| strain, strain background (*S. cerevisiae*) | ylr238wΔ | YSC1053 Yeast MATa deletion collection |  | http://www-sequence.stanford.edu/group/yeast_deletion_project/deletions3.html |
| strain, strain background (*S. cerevisiae*) | ylr239cΔ | YSC1053 Yeast MATa deletion collection |  | http://www-sequence.stanford.edu/group/yeast_deletion_project/deletions3.html |
| strain, strain background (*S. cerevisiae*) | ylr240wΔ | YSC1053 Yeast MATa deletion collection |  | http://www-sequence.stanford.edu/group/yeast_deletion_project/deletions3.html |
| strain, strain background (*S. cerevisiae*) | ylr241wΔ | YSC1053 Yeast MATa deletion collection |  | http://www-sequence.stanford.edu/group/yeast_deletion_project/deletions3.html |
| strain, strain background (*S. cerevisiae*) | ylr242cΔ | YSC1053 Yeast MATa deletion collection |  | http://www-sequence.stanford.edu/group/yeast_deletion_project/deletions3.html |
| strain, strain background (*S. cerevisiae*) | ylr244cΔ | YSC1053 Yeast MATa deletion collection |  | http://www-sequence.stanford.edu/group/yeast_deletion_project/deletions3.html |
| strain, strain background (*S. cerevisiae*) | ylr246wΔ | YSC1053 Yeast MATa deletion collection |  | http://www-sequence.stanford.edu/group/yeast_deletion_project/deletions3.html |
| strain, strain background (*S. cerevisiae*) | ylr248wΔ | YSC1053 Yeast MATa deletion collection |  | http://www-sequence.stanford.edu/group/yeast_deletion_project/deletions3.html |
| strain, strain background (*S. cerevisiae*) | ylr260wΔ | YSC1053 Yeast MATa deletion collection |  | http://www-sequence.stanford.edu/group/yeast_deletion_project/deletions3.html |
| strain, strain background (*S. cerevisiae*) | ylr261cΔ | YSC1053 Yeast MATa deletion collection |  | http://www-sequence.stanford.edu/group/yeast_deletion_project/deletions3.html |
| strain, strain background (*S. cerevisiae*) | ylr262cΔ | YSC1053 Yeast MATa deletion collection |  | http://www-sequence.stanford.edu/group/yeast_deletion_project/deletions3.html |
| strain, strain background (*S. cerevisiae*) | ylr263wΔ | YSC1053 Yeast MATa deletion collection |  | http://www-sequence.stanford.edu/group/yeast_deletion_project/deletions3.html |
| strain, strain background (*S. cerevisiae*) | ylr264c-aΔ | YSC1053 Yeast MATa deletion collection |  | http://www-sequence.stanford.edu/group/yeast_deletion_project/deletions3.html |
| strain, strain background (*S. cerevisiae*) | ylr264wΔ | YSC1053 Yeast MATa deletion collection |  | http://www-sequence.stanford.edu/group/yeast_deletion_project/deletions3.html |
| strain, strain background (*S. cerevisiae*) | ylr266cΔ | YSC1053 Yeast MATa deletion collection |  | http://www-sequence.stanford.edu/group/yeast_deletion_project/deletions3.html |
| strain, strain background (*S. cerevisiae*) | ylr269cΔ | YSC1053 Yeast MATa deletion collection |  | http://www-sequence.stanford.edu/group/yeast_deletion_project/deletions3.html |
| strain, strain background (*S. cerevisiae*) | ylr273cΔ | YSC1053 Yeast MATa deletion collection |  | http://www-sequence.stanford.edu/group/yeast_deletion_project/deletions3.html |
| strain, strain background (*S. cerevisiae*) | ylr290cΔ | YSC1053 Yeast MATa deletion collection |  | http://www-sequence.stanford.edu/group/yeast_deletion_project/deletions3.html |
| strain, strain background (*S. cerevisiae*) | ylr295cΔ | YSC1053 Yeast MATa deletion collection |  | http://www-sequence.stanford.edu/group/yeast_deletion_project/deletions3.html |
| strain, strain background (*S. cerevisiae*) | ylr304cΔ | YSC1053 Yeast MATa deletion collection |  | http://www-sequence.stanford.edu/group/yeast_deletion_project/deletions3.html |
| strain, strain background (*S. cerevisiae*) | ylr312w-aΔ | YSC1053 Yeast MATa deletion collection |  | http://www-sequence.stanford.edu/group/yeast_deletion_project/deletions3.html |
| strain, strain background (*S. cerevisiae*) | ylr315wΔ | YSC1053 Yeast MATa deletion collection |  | http://www-sequence.stanford.edu/group/yeast_deletion_project/deletions3.html |
| strain, strain background (*S. cerevisiae*) | ylr318wΔ | YSC1053 Yeast MATa deletion collection |  | http://www-sequence.stanford.edu/group/yeast_deletion_project/deletions3.html |
| strain, strain background (*S. cerevisiae*) | ylr320wΔ | YSC1053 Yeast MATa deletion collection |  | http://www-sequence.stanford.edu/group/yeast_deletion_project/deletions3.html |
| strain, strain background (*S. cerevisiae*) | ylr322wΔ | YSC1053 Yeast MATa deletion collection |  | http://www-sequence.stanford.edu/group/yeast_deletion_project/deletions3.html |
| strain, strain background (*S. cerevisiae*) | ylr325cΔ | YSC1053 Yeast MATa deletion collection |  | http://www-sequence.stanford.edu/group/yeast_deletion_project/deletions3.html |
| strain, strain background (*S. cerevisiae*) | ylr330wΔ | YSC1053 Yeast MATa deletion collection |  | http://www-sequence.stanford.edu/group/yeast_deletion_project/deletions3.html |
| strain, strain background (*S. cerevisiae*) | ylr335wΔ | YSC1053 Yeast MATa deletion collection |  | http://www-sequence.stanford.edu/group/yeast_deletion_project/deletions3.html |
| strain, strain background (*S. cerevisiae*) | ylr337cΔ | YSC1053 Yeast MATa deletion collection |  | http://www-sequence.stanford.edu/group/yeast_deletion_project/deletions3.html |
| strain, strain background (*S. cerevisiae*) | ylr338wΔ | YSC1053 Yeast MATa deletion collection |  | http://www-sequence.stanford.edu/group/yeast_deletion_project/deletions3.html |
| strain, strain background (*S. cerevisiae*) | ylr342wΔ | YSC1053 Yeast MATa deletion collection |  | http://www-sequence.stanford.edu/group/yeast_deletion_project/deletions3.html |
| strain, strain background (*S. cerevisiae*) | ylr344wΔ | YSC1053 Yeast MATa deletion collection |  | http://www-sequence.stanford.edu/group/yeast_deletion_project/deletions3.html |
| strain, strain background (*S. cerevisiae*) | ylr350wΔ | YSC1053 Yeast MATa deletion collection |  | http://www-sequence.stanford.edu/group/yeast_deletion_project/deletions3.html |
| strain, strain background (*S. cerevisiae*) | ylr351cΔ | YSC1053 Yeast MATa deletion collection |  | http://www-sequence.stanford.edu/group/yeast_deletion_project/deletions3.html |
| strain, strain background (*S. cerevisiae*) | ylr357wΔ | YSC1053 Yeast MATa deletion collection |  | http://www-sequence.stanford.edu/group/yeast_deletion_project/deletions3.html |
| strain, strain background (*S. cerevisiae*) | ylr358cΔ | YSC1053 Yeast MATa deletion collection |  | http://www-sequence.stanford.edu/group/yeast_deletion_project/deletions3.html |
| strain, strain background (*S. cerevisiae*) | ylr361cΔ | YSC1053 Yeast MATa deletion collection |  | http://www-sequence.stanford.edu/group/yeast_deletion_project/deletions3.html |
| strain, strain background (*S. cerevisiae*) | ylr362wΔ | YSC1053 Yeast MATa deletion collection |  | http://www-sequence.stanford.edu/group/yeast_deletion_project/deletions3.html |
| strain, strain background (*S. cerevisiae*) | ylr363w-aΔ | YSC1053 Yeast MATa deletion collection |  | http://www-sequence.stanford.edu/group/yeast_deletion_project/deletions3.html |
| strain, strain background (*S. cerevisiae*) | ylr366wΔ | YSC1053 Yeast MATa deletion collection |  | http://www-sequence.stanford.edu/group/yeast_deletion_project/deletions3.html |
| strain, strain background (*S. cerevisiae*) | ylr367wΔ | YSC1053 Yeast MATa deletion collection |  | http://www-sequence.stanford.edu/group/yeast_deletion_project/deletions3.html |
| strain, strain background (*S. cerevisiae*) | ylr369wΔ | YSC1053 Yeast MATa deletion collection |  | http://www-sequence.stanford.edu/group/yeast_deletion_project/deletions3.html |
| strain, strain background (*S. cerevisiae*) | ylr370cΔ | YSC1053 Yeast MATa deletion collection |  | http://www-sequence.stanford.edu/group/yeast_deletion_project/deletions3.html |
| strain, strain background (*S. cerevisiae*) | ylr371wΔ | YSC1053 Yeast MATa deletion collection |  | http://www-sequence.stanford.edu/group/yeast_deletion_project/deletions3.html |
| strain, strain background (*S. cerevisiae*) | ylr372wΔ | YSC1053 Yeast MATa deletion collection |  | http://www-sequence.stanford.edu/group/yeast_deletion_project/deletions3.html |
| strain, strain background (*S. cerevisiae*) | ylr373cΔ | YSC1053 Yeast MATa deletion collection |  | http://www-sequence.stanford.edu/group/yeast_deletion_project/deletions3.html |
| strain, strain background (*S. cerevisiae*) | ylr374cΔ | YSC1053 Yeast MATa deletion collection |  | http://www-sequence.stanford.edu/group/yeast_deletion_project/deletions3.html |
| strain, strain background (*S. cerevisiae*) | ylr376cΔ | YSC1053 Yeast MATa deletion collection |  | http://www-sequence.stanford.edu/group/yeast_deletion_project/deletions3.html |
| strain, strain background (*S. cerevisiae*) | ylr380wΔ | YSC1053 Yeast MATa deletion collection |  | http://www-sequence.stanford.edu/group/yeast_deletion_project/deletions3.html |
| strain, strain background (*S. cerevisiae*) | ylr382cΔ | YSC1053 Yeast MATa deletion collection |  | http://www-sequence.stanford.edu/group/yeast_deletion_project/deletions3.html |
| strain, strain background (*S. cerevisiae*) | ylr384cΔ | YSC1053 Yeast MATa deletion collection |  | http://www-sequence.stanford.edu/group/yeast_deletion_project/deletions3.html |
| strain, strain background (*S. cerevisiae*) | ylr385cΔ | YSC1053 Yeast MATa deletion collection |  | http://www-sequence.stanford.edu/group/yeast_deletion_project/deletions3.html |
| strain, strain background (*S. cerevisiae*) | ylr388wΔ | YSC1053 Yeast MATa deletion collection |  | http://www-sequence.stanford.edu/group/yeast_deletion_project/deletions3.html |
| strain, strain background (*S. cerevisiae*) | ylr389cΔ | YSC1053 Yeast MATa deletion collection |  | http://www-sequence.stanford.edu/group/yeast_deletion_project/deletions3.html |
| strain, strain background (*S. cerevisiae*) | ylr393wΔ | YSC1053 Yeast MATa deletion collection |  | http://www-sequence.stanford.edu/group/yeast_deletion_project/deletions3.html |
| strain, strain background (*S. cerevisiae*) | ylr396cΔ | YSC1053 Yeast MATa deletion collection |  | http://www-sequence.stanford.edu/group/yeast_deletion_project/deletions3.html |
| strain, strain background (*S. cerevisiae*) | ylr398cΔ | YSC1053 Yeast MATa deletion collection |  | http://www-sequence.stanford.edu/group/yeast_deletion_project/deletions3.html |
| strain, strain background (*S. cerevisiae*) | ylr399cΔ | YSC1053 Yeast MATa deletion collection |  | http://www-sequence.stanford.edu/group/yeast_deletion_project/deletions3.html |
| strain, strain background (*S. cerevisiae*) | ylr402wΔ | YSC1053 Yeast MATa deletion collection |  | http://www-sequence.stanford.edu/group/yeast_deletion_project/deletions3.html |
| strain, strain background (*S. cerevisiae*) | ylr403wΔ | YSC1053 Yeast MATa deletion collection |  | http://www-sequence.stanford.edu/group/yeast_deletion_project/deletions3.html |
| strain, strain background (*S. cerevisiae*) | ylr407wΔ | YSC1053 Yeast MATa deletion collection |  | http://www-sequence.stanford.edu/group/yeast_deletion_project/deletions3.html |
| strain, strain background (*S. cerevisiae*) | ylr410wΔ | YSC1053 Yeast MATa deletion collection |  | http://www-sequence.stanford.edu/group/yeast_deletion_project/deletions3.html |
| strain, strain background (*S. cerevisiae*) | ylr412wΔ | YSC1053 Yeast MATa deletion collection |  | http://www-sequence.stanford.edu/group/yeast_deletion_project/deletions3.html |
| strain, strain background (*S. cerevisiae*) | ylr414cΔ | YSC1053 Yeast MATa deletion collection |  | http://www-sequence.stanford.edu/group/yeast_deletion_project/deletions3.html |
| strain, strain background (*S. cerevisiae*) | ylr416cΔ | YSC1053 Yeast MATa deletion collection |  | http://www-sequence.stanford.edu/group/yeast_deletion_project/deletions3.html |
| strain, strain background (*S. cerevisiae*) | ylr417wΔ | YSC1053 Yeast MATa deletion collection |  | http://www-sequence.stanford.edu/group/yeast_deletion_project/deletions3.html |
| strain, strain background (*S. cerevisiae*) | ylr418cΔ | YSC1053 Yeast MATa deletion collection |  | http://www-sequence.stanford.edu/group/yeast_deletion_project/deletions3.html |
| strain, strain background (*S. cerevisiae*) | ylr422wΔ | YSC1053 Yeast MATa deletion collection |  | http://www-sequence.stanford.edu/group/yeast_deletion_project/deletions3.html |
| strain, strain background (*S. cerevisiae*) | ylr425wΔ | YSC1053 Yeast MATa deletion collection |  | http://www-sequence.stanford.edu/group/yeast_deletion_project/deletions3.html |
| strain, strain background (*S. cerevisiae*) | ylr426wΔ | YSC1053 Yeast MATa deletion collection |  | http://www-sequence.stanford.edu/group/yeast_deletion_project/deletions3.html |
| strain, strain background (*S. cerevisiae*) | ylr439wΔ | YSC1053 Yeast MATa deletion collection |  | http://www-sequence.stanford.edu/group/yeast_deletion_project/deletions3.html |
| strain, strain background (*S. cerevisiae*) | ylr441cΔ | YSC1053 Yeast MATa deletion collection |  | http://www-sequence.stanford.edu/group/yeast_deletion_project/deletions3.html |
| strain, strain background (*S. cerevisiae*) | ylr443wΔ | YSC1053 Yeast MATa deletion collection |  | http://www-sequence.stanford.edu/group/yeast_deletion_project/deletions3.html |
| strain, strain background (*S. cerevisiae*) | ylr445wΔ | YSC1053 Yeast MATa deletion collection |  | http://www-sequence.stanford.edu/group/yeast_deletion_project/deletions3.html |
| strain, strain background (*S. cerevisiae*) | ylr447cΔ | YSC1053 Yeast MATa deletion collection |  | http://www-sequence.stanford.edu/group/yeast_deletion_project/deletions3.html |
| strain, strain background (*S. cerevisiae*) | ylr448wΔ | YSC1053 Yeast MATa deletion collection |  | http://www-sequence.stanford.edu/group/yeast_deletion_project/deletions3.html |
| strain, strain background (*S. cerevisiae*) | ylr452cΔ | YSC1053 Yeast MATa deletion collection |  | http://www-sequence.stanford.edu/group/yeast_deletion_project/deletions3.html |
| strain, strain background (*S. cerevisiae*) | yml001wΔ | YSC1053 Yeast MATa deletion collection |  | http://www-sequence.stanford.edu/group/yeast_deletion_project/deletions3.html |
| strain, strain background (*S. cerevisiae*) | yml005wΔ | YSC1053 Yeast MATa deletion collection |  | http://www-sequence.stanford.edu/group/yeast_deletion_project/deletions3.html |
| strain, strain background (*S. cerevisiae*) | yml006cΔ | YSC1053 Yeast MATa deletion collection |  | http://www-sequence.stanford.edu/group/yeast_deletion_project/deletions3.html |
| strain, strain background (*S. cerevisiae*) | yml007wΔ | YSC1053 Yeast MATa deletion collection |  | http://www-sequence.stanford.edu/group/yeast_deletion_project/deletions3.html |
| strain, strain background (*S. cerevisiae*) | yml010c-bΔ | YSC1053 Yeast MATa deletion collection |  | http://www-sequence.stanford.edu/group/yeast_deletion_project/deletions3.html |
| strain, strain background (*S. cerevisiae*) | yml013wΔ | YSC1053 Yeast MATa deletion collection |  | http://www-sequence.stanford.edu/group/yeast_deletion_project/deletions3.html |
| strain, strain background (*S. cerevisiae*) | yml014wΔ | YSC1053 Yeast MATa deletion collection |  | http://www-sequence.stanford.edu/group/yeast_deletion_project/deletions3.html |
| strain, strain background (*S. cerevisiae*) | yml020wΔ | YSC1053 Yeast MATa deletion collection |  | http://www-sequence.stanford.edu/group/yeast_deletion_project/deletions3.html |
| strain, strain background (*S. cerevisiae*) | yml021cΔ | YSC1053 Yeast MATa deletion collection |  | http://www-sequence.stanford.edu/group/yeast_deletion_project/deletions3.html |
| strain, strain background (*S. cerevisiae*) | yml024wΔ | YSC1053 Yeast MATa deletion collection |  | http://www-sequence.stanford.edu/group/yeast_deletion_project/deletions3.html |
| strain, strain background (*S. cerevisiae*) | yml026cΔ | YSC1053 Yeast MATa deletion collection |  | http://www-sequence.stanford.edu/group/yeast_deletion_project/deletions3.html |
| strain, strain background (*S. cerevisiae*) | yml028wΔ | YSC1053 Yeast MATa deletion collection |  | http://www-sequence.stanford.edu/group/yeast_deletion_project/deletions3.html |
| strain, strain background (*S. cerevisiae*) | yml029wΔ | YSC1053 Yeast MATa deletion collection |  | http://www-sequence.stanford.edu/group/yeast_deletion_project/deletions3.html |
| strain, strain background (*S. cerevisiae*) | yml032cΔ | YSC1053 Yeast MATa deletion collection |  | http://www-sequence.stanford.edu/group/yeast_deletion_project/deletions3.html |
| strain, strain background (*S. cerevisiae*) | yml036wΔ | YSC1053 Yeast MATa deletion collection |  | http://www-sequence.stanford.edu/group/yeast_deletion_project/deletions3.html |
| strain, strain background (*S. cerevisiae*) | yml041cΔ | YSC1053 Yeast MATa deletion collection |  | http://www-sequence.stanford.edu/group/yeast_deletion_project/deletions3.html |
| strain, strain background (*S. cerevisiae*) | yml061cΔ | YSC1053 Yeast MATa deletion collection |  | http://www-sequence.stanford.edu/group/yeast_deletion_project/deletions3.html |
| strain, strain background (*S. cerevisiae*) | yml063wΔ | YSC1053 Yeast MATa deletion collection |  | http://www-sequence.stanford.edu/group/yeast_deletion_project/deletions3.html |
| strain, strain background (*S. cerevisiae*) | yml066cΔ | YSC1053 Yeast MATa deletion collection |  | http://www-sequence.stanford.edu/group/yeast_deletion_project/deletions3.html |
| strain, strain background (*S. cerevisiae*) | yml073cΔ | YSC1053 Yeast MATa deletion collection |  | http://www-sequence.stanford.edu/group/yeast_deletion_project/deletions3.html |
| strain, strain background (*S. cerevisiae*) | yml076cΔ | YSC1053 Yeast MATa deletion collection |  | http://www-sequence.stanford.edu/group/yeast_deletion_project/deletions3.html |
| strain, strain background (*S. cerevisiae*) | yml081c-aΔ | YSC1053 Yeast MATa deletion collection |  | http://www-sequence.stanford.edu/group/yeast_deletion_project/deletions3.html |
| strain, strain background (*S. cerevisiae*) | yml081wΔ | YSC1053 Yeast MATa deletion collection |  | http://www-sequence.stanford.edu/group/yeast_deletion_project/deletions3.html |
| strain, strain background (*S. cerevisiae*) | yml082wΔ | YSC1053 Yeast MATa deletion collection |  | http://www-sequence.stanford.edu/group/yeast_deletion_project/deletions3.html |
| strain, strain background (*S. cerevisiae*) | yml088wΔ | YSC1053 Yeast MATa deletion collection |  | http://www-sequence.stanford.edu/group/yeast_deletion_project/deletions3.html |
| strain, strain background (*S. cerevisiae*) | yml090wΔ | YSC1053 Yeast MATa deletion collection |  | http://www-sequence.stanford.edu/group/yeast_deletion_project/deletions3.html |
| strain, strain background (*S. cerevisiae*) | yml094wΔ | YSC1053 Yeast MATa deletion collection |  | http://www-sequence.stanford.edu/group/yeast_deletion_project/deletions3.html |
| strain, strain background (*S. cerevisiae*) | yml097cΔ | YSC1053 Yeast MATa deletion collection |  | http://www-sequence.stanford.edu/group/yeast_deletion_project/deletions3.html |
| strain, strain background (*S. cerevisiae*) | yml100wΔ | YSC1053 Yeast MATa deletion collection |  | http://www-sequence.stanford.edu/group/yeast_deletion_project/deletions3.html |
| strain, strain background (*S. cerevisiae*) | yml102c-aΔ | YSC1053 Yeast MATa deletion collection |  | http://www-sequence.stanford.edu/group/yeast_deletion_project/deletions3.html |
| strain, strain background (*S. cerevisiae*) | yml102wΔ | YSC1053 Yeast MATa deletion collection |  | http://www-sequence.stanford.edu/group/yeast_deletion_project/deletions3.html |
| strain, strain background (*S. cerevisiae*) | yml103cΔ | YSC1053 Yeast MATa deletion collection |  | http://www-sequence.stanford.edu/group/yeast_deletion_project/deletions3.html |
| strain, strain background (*S. cerevisiae*) | yml106wΔ | YSC1053 Yeast MATa deletion collection |  | http://www-sequence.stanford.edu/group/yeast_deletion_project/deletions3.html |
| strain, strain background (*S. cerevisiae*) | yml110cΔ | YSC1053 Yeast MATa deletion collection |  | http://www-sequence.stanford.edu/group/yeast_deletion_project/deletions3.html |
| strain, strain background (*S. cerevisiae*) | yml112wΔ | YSC1053 Yeast MATa deletion collection |  | http://www-sequence.stanford.edu/group/yeast_deletion_project/deletions3.html |
| strain, strain background (*S. cerevisiae*) | yml115cΔ | YSC1053 Yeast MATa deletion collection |  | http://www-sequence.stanford.edu/group/yeast_deletion_project/deletions3.html |
| strain, strain background (*S. cerevisiae*) | yml121wΔ | YSC1053 Yeast MATa deletion collection |  | http://www-sequence.stanford.edu/group/yeast_deletion_project/deletions3.html |
| strain, strain background (*S. cerevisiae*) | yml123cΔ | YSC1053 Yeast MATa deletion collection |  | http://www-sequence.stanford.edu/group/yeast_deletion_project/deletions3.html |
| strain, strain background (*S. cerevisiae*) | yml128cΔ | YSC1053 Yeast MATa deletion collection |  | http://www-sequence.stanford.edu/group/yeast_deletion_project/deletions3.html |
| strain, strain background (*S. cerevisiae*) | ymr016cΔ | YSC1053 Yeast MATa deletion collection |  | http://www-sequence.stanford.edu/group/yeast_deletion_project/deletions3.html |
| strain, strain background (*S. cerevisiae*) | ymr022wΔ | YSC1053 Yeast MATa deletion collection |  | http://www-sequence.stanford.edu/group/yeast_deletion_project/deletions3.html |
| strain, strain background (*S. cerevisiae*) | ymr023cΔ | YSC1053 Yeast MATa deletion collection |  | http://www-sequence.stanford.edu/group/yeast_deletion_project/deletions3.html |
| strain, strain background (*S. cerevisiae*) | ymr024wΔ | YSC1053 Yeast MATa deletion collection |  | http://www-sequence.stanford.edu/group/yeast_deletion_project/deletions3.html |
| strain, strain background (*S. cerevisiae*) | ymr026cΔ | YSC1053 Yeast MATa deletion collection |  | http://www-sequence.stanford.edu/group/yeast_deletion_project/deletions3.html |
| strain, strain background (*S. cerevisiae*) | ymr031w-aΔ | YSC1053 Yeast MATa deletion collection |  | http://www-sequence.stanford.edu/group/yeast_deletion_project/deletions3.html |
| strain, strain background (*S. cerevisiae*) | ymr032wΔ | YSC1053 Yeast MATa deletion collection |  | http://www-sequence.stanford.edu/group/yeast_deletion_project/deletions3.html |
| strain, strain background (*S. cerevisiae*) | ymr037cΔ | YSC1053 Yeast MATa deletion collection |  | http://www-sequence.stanford.edu/group/yeast_deletion_project/deletions3.html |
| strain, strain background (*S. cerevisiae*) | ymr052c-aΔ | YSC1053 Yeast MATa deletion collection |  | http://www-sequence.stanford.edu/group/yeast_deletion_project/deletions3.html |
| strain, strain background (*S. cerevisiae*) | ymr056cΔ | YSC1053 Yeast MATa deletion collection |  | http://www-sequence.stanford.edu/group/yeast_deletion_project/deletions3.html |
| strain, strain background (*S. cerevisiae*) | ymr060cΔ | YSC1053 Yeast MATa deletion collection |  | http://www-sequence.stanford.edu/group/yeast_deletion_project/deletions3.html |
| strain, strain background (*S. cerevisiae*) | ymr062cΔ | YSC1053 Yeast MATa deletion collection |  | http://www-sequence.stanford.edu/group/yeast_deletion_project/deletions3.html |
| strain, strain background (*S. cerevisiae*) | ymr063wΔ | YSC1053 Yeast MATa deletion collection |  | http://www-sequence.stanford.edu/group/yeast_deletion_project/deletions3.html |
| strain, strain background (*S. cerevisiae*) | ymr064wΔ | YSC1053 Yeast MATa deletion collection |  | http://www-sequence.stanford.edu/group/yeast_deletion_project/deletions3.html |
| strain, strain background (*S. cerevisiae*) | ymr066wΔ | YSC1053 Yeast MATa deletion collection |  | http://www-sequence.stanford.edu/group/yeast_deletion_project/deletions3.html |
| strain, strain background (*S. cerevisiae*) | ymr071cΔ | YSC1053 Yeast MATa deletion collection |  | http://www-sequence.stanford.edu/group/yeast_deletion_project/deletions3.html |
| strain, strain background (*S. cerevisiae*) | ymr072wΔ | YSC1053 Yeast MATa deletion collection |  | http://www-sequence.stanford.edu/group/yeast_deletion_project/deletions3.html |
| strain, strain background (*S. cerevisiae*) | ymr073cΔ | YSC1053 Yeast MATa deletion collection |  | http://www-sequence.stanford.edu/group/yeast_deletion_project/deletions3.html |
| strain, strain background (*S. cerevisiae*) | ymr074cΔ | YSC1053 Yeast MATa deletion collection |  | http://www-sequence.stanford.edu/group/yeast_deletion_project/deletions3.html |
| strain, strain background (*S. cerevisiae*) | ymr075wΔ | YSC1053 Yeast MATa deletion collection |  | http://www-sequence.stanford.edu/group/yeast_deletion_project/deletions3.html |
| strain, strain background (*S. cerevisiae*) | ymr077cΔ | YSC1053 Yeast MATa deletion collection |  | http://www-sequence.stanford.edu/group/yeast_deletion_project/deletions3.html |
| strain, strain background (*S. cerevisiae*) | ymr078cΔ | YSC1053 Yeast MATa deletion collection |  | http://www-sequence.stanford.edu/group/yeast_deletion_project/deletions3.html |
| strain, strain background (*S. cerevisiae*) | ymr083wΔ | YSC1053 Yeast MATa deletion collection |  | http://www-sequence.stanford.edu/group/yeast_deletion_project/deletions3.html |
| strain, strain background (*S. cerevisiae*) | ymr089cΔ | YSC1053 Yeast MATa deletion collection |  | http://www-sequence.stanford.edu/group/yeast_deletion_project/deletions3.html |
| strain, strain background (*S. cerevisiae*) | ymr091cΔ | YSC1053 Yeast MATa deletion collection |  | http://www-sequence.stanford.edu/group/yeast_deletion_project/deletions3.html |
| strain, strain background (*S. cerevisiae*) | ymr097cΔ | YSC1053 Yeast MATa deletion collection |  | http://www-sequence.stanford.edu/group/yeast_deletion_project/deletions3.html |
| strain, strain background (*S. cerevisiae*) | ymr100wΔ | YSC1053 Yeast MATa deletion collection |  | http://www-sequence.stanford.edu/group/yeast_deletion_project/deletions3.html |
| strain, strain background (*S. cerevisiae*) | ymr106cΔ | YSC1053 Yeast MATa deletion collection |  | http://www-sequence.stanford.edu/group/yeast_deletion_project/deletions3.html |
| strain, strain background (*S. cerevisiae*) | ymr116cΔ | YSC1053 Yeast MATa deletion collection |  | http://www-sequence.stanford.edu/group/yeast_deletion_project/deletions3.html |
| strain, strain background (*S. cerevisiae*) | ymr123wΔ | YSC1053 Yeast MATa deletion collection |  | http://www-sequence.stanford.edu/group/yeast_deletion_project/deletions3.html |
| strain, strain background (*S. cerevisiae*) | ymr125wΔ | YSC1053 Yeast MATa deletion collection |  | http://www-sequence.stanford.edu/group/yeast_deletion_project/deletions3.html |
| strain, strain background (*S. cerevisiae*) | ymr136wΔ | YSC1053 Yeast MATa deletion collection |  | http://www-sequence.stanford.edu/group/yeast_deletion_project/deletions3.html |
| strain, strain background (*S. cerevisiae*) | ymr139wΔ | YSC1053 Yeast MATa deletion collection |  | http://www-sequence.stanford.edu/group/yeast_deletion_project/deletions3.html |
| strain, strain background (*S. cerevisiae*) | ymr140wΔ | YSC1053 Yeast MATa deletion collection |  | http://www-sequence.stanford.edu/group/yeast_deletion_project/deletions3.html |
| strain, strain background (*S. cerevisiae*) | ymr142cΔ | YSC1053 Yeast MATa deletion collection |  | http://www-sequence.stanford.edu/group/yeast_deletion_project/deletions3.html |
| strain, strain background (*S. cerevisiae*) | ymr143wΔ | YSC1053 Yeast MATa deletion collection |  | http://www-sequence.stanford.edu/group/yeast_deletion_project/deletions3.html |
| strain, strain background (*S. cerevisiae*) | ymr145cΔ | YSC1053 Yeast MATa deletion collection |  | http://www-sequence.stanford.edu/group/yeast_deletion_project/deletions3.html |
| strain, strain background (*S. cerevisiae*) | ymr147wΔ | YSC1053 Yeast MATa deletion collection |  | http://www-sequence.stanford.edu/group/yeast_deletion_project/deletions3.html |
| strain, strain background (*S. cerevisiae*) | ymr148wΔ | YSC1053 Yeast MATa deletion collection |  | http://www-sequence.stanford.edu/group/yeast_deletion_project/deletions3.html |
| strain, strain background (*S. cerevisiae*) | ymr150cΔ | YSC1053 Yeast MATa deletion collection |  | http://www-sequence.stanford.edu/group/yeast_deletion_project/deletions3.html |
| strain, strain background (*S. cerevisiae*) | ymr153c-aΔ | YSC1053 Yeast MATa deletion collection |  | http://www-sequence.stanford.edu/group/yeast_deletion_project/deletions3.html |
| strain, strain background (*S. cerevisiae*) | ymr154cΔ | YSC1053 Yeast MATa deletion collection |  | http://www-sequence.stanford.edu/group/yeast_deletion_project/deletions3.html |
| strain, strain background (*S. cerevisiae*) | ymr158wΔ | YSC1053 Yeast MATa deletion collection |  | http://www-sequence.stanford.edu/group/yeast_deletion_project/deletions3.html |
| strain, strain background (*S. cerevisiae*) | ymr163cΔ | YSC1053 Yeast MATa deletion collection |  | http://www-sequence.stanford.edu/group/yeast_deletion_project/deletions3.html |
| strain, strain background (*S. cerevisiae*) | ymr166cΔ | YSC1053 Yeast MATa deletion collection |  | http://www-sequence.stanford.edu/group/yeast_deletion_project/deletions3.html |
| strain, strain background (*S. cerevisiae*) | ymr167wΔ | YSC1053 Yeast MATa deletion collection |  | http://www-sequence.stanford.edu/group/yeast_deletion_project/deletions3.html |
| strain, strain background (*S. cerevisiae*) | ymr172wΔ | YSC1053 Yeast MATa deletion collection |  | http://www-sequence.stanford.edu/group/yeast_deletion_project/deletions3.html |
| strain, strain background (*S. cerevisiae*) | ymr175wΔ | YSC1053 Yeast MATa deletion collection |  | http://www-sequence.stanford.edu/group/yeast_deletion_project/deletions3.html |
| strain, strain background (*S. cerevisiae*) | ymr179wΔ | YSC1053 Yeast MATa deletion collection |  | http://www-sequence.stanford.edu/group/yeast_deletion_project/deletions3.html |
| strain, strain background (*S. cerevisiae*) | ymr183cΔ | YSC1053 Yeast MATa deletion collection |  | http://www-sequence.stanford.edu/group/yeast_deletion_project/deletions3.html |
| strain, strain background (*S. cerevisiae*) | ymr186wΔ | YSC1053 Yeast MATa deletion collection |  | http://www-sequence.stanford.edu/group/yeast_deletion_project/deletions3.html |
| strain, strain background (*S. cerevisiae*) | ymr188cΔ | YSC1053 Yeast MATa deletion collection |  | http://www-sequence.stanford.edu/group/yeast_deletion_project/deletions3.html |
| strain, strain background (*S. cerevisiae*) | ymr190cΔ | YSC1053 Yeast MATa deletion collection |  | http://www-sequence.stanford.edu/group/yeast_deletion_project/deletions3.html |
| strain, strain background (*S. cerevisiae*) | ymr191wΔ | YSC1053 Yeast MATa deletion collection |  | http://www-sequence.stanford.edu/group/yeast_deletion_project/deletions3.html |
| strain, strain background (*S. cerevisiae*) | ymr193c-aΔ | YSC1053 Yeast MATa deletion collection |  | http://www-sequence.stanford.edu/group/yeast_deletion_project/deletions3.html |
| strain, strain background (*S. cerevisiae*) | ymr193wΔ | YSC1053 Yeast MATa deletion collection |  | http://www-sequence.stanford.edu/group/yeast_deletion_project/deletions3.html |
| strain, strain background (*S. cerevisiae*) | ymr194wΔ | YSC1053 Yeast MATa deletion collection |  | http://www-sequence.stanford.edu/group/yeast_deletion_project/deletions3.html |
| strain, strain background (*S. cerevisiae*) | ymr195wΔ | YSC1053 Yeast MATa deletion collection |  | http://www-sequence.stanford.edu/group/yeast_deletion_project/deletions3.html |
| strain, strain background (*S. cerevisiae*) | ymr198wΔ | YSC1053 Yeast MATa deletion collection |  | http://www-sequence.stanford.edu/group/yeast_deletion_project/deletions3.html |
| strain, strain background (*S. cerevisiae*) | ymr201cΔ | YSC1053 Yeast MATa deletion collection |  | http://www-sequence.stanford.edu/group/yeast_deletion_project/deletions3.html |
| strain, strain background (*S. cerevisiae*) | ymr202wΔ | YSC1053 Yeast MATa deletion collection |  | http://www-sequence.stanford.edu/group/yeast_deletion_project/deletions3.html |
| strain, strain background (*S. cerevisiae*) | ymr204cΔ | YSC1053 Yeast MATa deletion collection |  | http://www-sequence.stanford.edu/group/yeast_deletion_project/deletions3.html |
| strain, strain background (*S. cerevisiae*) | ymr205cΔ | YSC1053 Yeast MATa deletion collection |  | http://www-sequence.stanford.edu/group/yeast_deletion_project/deletions3.html |
| strain, strain background (*S. cerevisiae*) | ymr207cΔ | YSC1053 Yeast MATa deletion collection |  | http://www-sequence.stanford.edu/group/yeast_deletion_project/deletions3.html |
| strain, strain background (*S. cerevisiae*) | ymr214wΔ | YSC1053 Yeast MATa deletion collection |  | http://www-sequence.stanford.edu/group/yeast_deletion_project/deletions3.html |
| strain, strain background (*S. cerevisiae*) | ymr216cΔ | YSC1053 Yeast MATa deletion collection |  | http://www-sequence.stanford.edu/group/yeast_deletion_project/deletions3.html |
| strain, strain background (*S. cerevisiae*) | ymr223wΔ | YSC1053 Yeast MATa deletion collection |  | http://www-sequence.stanford.edu/group/yeast_deletion_project/deletions3.html |
| strain, strain background (*S. cerevisiae*) | ymr224cΔ | YSC1053 Yeast MATa deletion collection |  | http://www-sequence.stanford.edu/group/yeast_deletion_project/deletions3.html |
| strain, strain background (*S. cerevisiae*) | ymr226cΔ | YSC1053 Yeast MATa deletion collection |  | http://www-sequence.stanford.edu/group/yeast_deletion_project/deletions3.html |
| strain, strain background (*S. cerevisiae*) | ymr228wΔ | YSC1053 Yeast MATa deletion collection |  | http://www-sequence.stanford.edu/group/yeast_deletion_project/deletions3.html |
| strain, strain background (*S. cerevisiae*) | ymr230wΔ | YSC1053 Yeast MATa deletion collection |  | http://www-sequence.stanford.edu/group/yeast_deletion_project/deletions3.html |
| strain, strain background (*S. cerevisiae*) | ymr231wΔ | YSC1053 Yeast MATa deletion collection |  | http://www-sequence.stanford.edu/group/yeast_deletion_project/deletions3.html |
| strain, strain background (*S. cerevisiae*) | ymr233wΔ | YSC1053 Yeast MATa deletion collection |  | http://www-sequence.stanford.edu/group/yeast_deletion_project/deletions3.html |
| strain, strain background (*S. cerevisiae*) | ymr237wΔ | YSC1053 Yeast MATa deletion collection |  | http://www-sequence.stanford.edu/group/yeast_deletion_project/deletions3.html |
| strain, strain background (*S. cerevisiae*) | ymr241wΔ | YSC1053 Yeast MATa deletion collection |  | http://www-sequence.stanford.edu/group/yeast_deletion_project/deletions3.html |
| strain, strain background (*S. cerevisiae*) | ymr242cΔ | YSC1053 Yeast MATa deletion collection |  | http://www-sequence.stanford.edu/group/yeast_deletion_project/deletions3.html |
| strain, strain background (*S. cerevisiae*) | ymr242w-aΔ | YSC1053 Yeast MATa deletion collection |  | http://www-sequence.stanford.edu/group/yeast_deletion_project/deletions3.html |
| strain, strain background (*S. cerevisiae*) | ymr243cΔ | YSC1053 Yeast MATa deletion collection |  | http://www-sequence.stanford.edu/group/yeast_deletion_project/deletions3.html |
| strain, strain background (*S. cerevisiae*) | ymr256cΔ | YSC1053 Yeast MATa deletion collection |  | http://www-sequence.stanford.edu/group/yeast_deletion_project/deletions3.html |
| strain, strain background (*S. cerevisiae*) | ymr257cΔ | YSC1053 Yeast MATa deletion collection |  | http://www-sequence.stanford.edu/group/yeast_deletion_project/deletions3.html |
| strain, strain background (*S. cerevisiae*) | ymr263wΔ | YSC1053 Yeast MATa deletion collection |  | http://www-sequence.stanford.edu/group/yeast_deletion_project/deletions3.html |
| strain, strain background (*S. cerevisiae*) | ymr264wΔ | YSC1053 Yeast MATa deletion collection |  | http://www-sequence.stanford.edu/group/yeast_deletion_project/deletions3.html |
| strain, strain background (*S. cerevisiae*) | ymr269wΔ | YSC1053 Yeast MATa deletion collection |  | http://www-sequence.stanford.edu/group/yeast_deletion_project/deletions3.html |
| strain, strain background (*S. cerevisiae*) | ymr272cΔ | YSC1053 Yeast MATa deletion collection |  | http://www-sequence.stanford.edu/group/yeast_deletion_project/deletions3.html |
| strain, strain background (*S. cerevisiae*) | ymr275cΔ | YSC1053 Yeast MATa deletion collection |  | http://www-sequence.stanford.edu/group/yeast_deletion_project/deletions3.html |
| strain, strain background (*S. cerevisiae*) | ymr282cΔ | YSC1053 Yeast MATa deletion collection |  | http://www-sequence.stanford.edu/group/yeast_deletion_project/deletions3.html |
| strain, strain background (*S. cerevisiae*) | ymr286wΔ | YSC1053 Yeast MATa deletion collection |  | http://www-sequence.stanford.edu/group/yeast_deletion_project/deletions3.html |
| strain, strain background (*S. cerevisiae*) | ymr287cΔ | YSC1053 Yeast MATa deletion collection |  | http://www-sequence.stanford.edu/group/yeast_deletion_project/deletions3.html |
| strain, strain background (*S. cerevisiae*) | ymr293cΔ | YSC1053 Yeast MATa deletion collection |  | http://www-sequence.stanford.edu/group/yeast_deletion_project/deletions3.html |
| strain, strain background (*S. cerevisiae*) | ymr294wΔ | YSC1053 Yeast MATa deletion collection |  | http://www-sequence.stanford.edu/group/yeast_deletion_project/deletions3.html |
| strain, strain background (*S. cerevisiae*) | ymr304wΔ | YSC1053 Yeast MATa deletion collection |  | http://www-sequence.stanford.edu/group/yeast_deletion_project/deletions3.html |
| strain, strain background (*S. cerevisiae*) | ymr307wΔ | YSC1053 Yeast MATa deletion collection |  | http://www-sequence.stanford.edu/group/yeast_deletion_project/deletions3.html |
| strain, strain background (*S. cerevisiae*) | ymr311cΔ | YSC1053 Yeast MATa deletion collection |  | http://www-sequence.stanford.edu/group/yeast_deletion_project/deletions3.html |
| strain, strain background (*S. cerevisiae*) | ymr312wΔ | YSC1053 Yeast MATa deletion collection |  | http://www-sequence.stanford.edu/group/yeast_deletion_project/deletions3.html |
| strain, strain background (*S. cerevisiae*) | ynl001wΔ | YSC1053 Yeast MATa deletion collection |  | http://www-sequence.stanford.edu/group/yeast_deletion_project/deletions3.html |
| strain, strain background (*S. cerevisiae*) | ynl003cΔ | YSC1053 Yeast MATa deletion collection |  | http://www-sequence.stanford.edu/group/yeast_deletion_project/deletions3.html |
| strain, strain background (*S. cerevisiae*) | ynl005cΔ | YSC1053 Yeast MATa deletion collection |  | http://www-sequence.stanford.edu/group/yeast_deletion_project/deletions3.html |
| strain, strain background (*S. cerevisiae*) | ynl013cΔ | YSC1053 Yeast MATa deletion collection |  | http://www-sequence.stanford.edu/group/yeast_deletion_project/deletions3.html |
| strain, strain background (*S. cerevisiae*) | ynl016wΔ | YSC1053 Yeast MATa deletion collection |  | http://www-sequence.stanford.edu/group/yeast_deletion_project/deletions3.html |
| strain, strain background (*S. cerevisiae*) | ynl021wΔ | YSC1053 Yeast MATa deletion collection |  | http://www-sequence.stanford.edu/group/yeast_deletion_project/deletions3.html |
| strain, strain background (*S. cerevisiae*) | ynl025cΔ | YSC1053 Yeast MATa deletion collection |  | http://www-sequence.stanford.edu/group/yeast_deletion_project/deletions3.html |
| strain, strain background (*S. cerevisiae*) | ynl027wΔ | YSC1053 Yeast MATa deletion collection |  | http://www-sequence.stanford.edu/group/yeast_deletion_project/deletions3.html |
| strain, strain background (*S. cerevisiae*) | ynl037cΔ | YSC1053 Yeast MATa deletion collection |  | http://www-sequence.stanford.edu/group/yeast_deletion_project/deletions3.html |
| strain, strain background (*S. cerevisiae*) | ynl052wΔ | YSC1053 Yeast MATa deletion collection |  | http://www-sequence.stanford.edu/group/yeast_deletion_project/deletions3.html |
| strain, strain background (*S. cerevisiae*) | ynl054wΔ | YSC1053 Yeast MATa deletion collection |  | http://www-sequence.stanford.edu/group/yeast_deletion_project/deletions3.html |
| strain, strain background (*S. cerevisiae*) | ynl055cΔ | YSC1053 Yeast MATa deletion collection |  | http://www-sequence.stanford.edu/group/yeast_deletion_project/deletions3.html |
| strain, strain background (*S. cerevisiae*) | ynl059cΔ | YSC1053 Yeast MATa deletion collection |  | http://www-sequence.stanford.edu/group/yeast_deletion_project/deletions3.html |
| strain, strain background (*S. cerevisiae*) | ynl064cΔ | YSC1053 Yeast MATa deletion collection |  | http://www-sequence.stanford.edu/group/yeast_deletion_project/deletions3.html |
| strain, strain background (*S. cerevisiae*) | ynl067wΔ | YSC1053 Yeast MATa deletion collection |  | http://www-sequence.stanford.edu/group/yeast_deletion_project/deletions3.html |
| strain, strain background (*S. cerevisiae*) | ynl067w-bΔ | YSC1053 Yeast MATa deletion collection |  | http://www-sequence.stanford.edu/group/yeast_deletion_project/deletions3.html |
| strain, strain background (*S. cerevisiae*) | ynl071wΔ | YSC1053 Yeast MATa deletion collection |  | http://www-sequence.stanford.edu/group/yeast_deletion_project/deletions3.html |
| strain, strain background (*S. cerevisiae*) | ynl073wΔ | YSC1053 Yeast MATa deletion collection |  | http://www-sequence.stanford.edu/group/yeast_deletion_project/deletions3.html |
| strain, strain background (*S. cerevisiae*) | ynl076wΔ | YSC1053 Yeast MATa deletion collection |  | http://www-sequence.stanford.edu/group/yeast_deletion_project/deletions3.html |
| strain, strain background (*S. cerevisiae*) | ynl077wΔ | YSC1053 Yeast MATa deletion collection |  | http://www-sequence.stanford.edu/group/yeast_deletion_project/deletions3.html |
| strain, strain background (*S. cerevisiae*) | ynl079cΔ | YSC1053 Yeast MATa deletion collection |  | http://www-sequence.stanford.edu/group/yeast_deletion_project/deletions3.html |
| strain, strain background (*S. cerevisiae*) | ynl080cΔ | YSC1053 Yeast MATa deletion collection |  | http://www-sequence.stanford.edu/group/yeast_deletion_project/deletions3.html |
| strain, strain background (*S. cerevisiae*) | ynl081cΔ | YSC1053 Yeast MATa deletion collection |  | http://www-sequence.stanford.edu/group/yeast_deletion_project/deletions3.html |
| strain, strain background (*S. cerevisiae*) | ynl082wΔ | YSC1053 Yeast MATa deletion collection |  | http://www-sequence.stanford.edu/group/yeast_deletion_project/deletions3.html |
| strain, strain background (*S. cerevisiae*) | ynl083wΔ | YSC1053 Yeast MATa deletion collection |  | http://www-sequence.stanford.edu/group/yeast_deletion_project/deletions3.html |
| strain, strain background (*S. cerevisiae*) | ynl084cΔ | YSC1053 Yeast MATa deletion collection |  | http://www-sequence.stanford.edu/group/yeast_deletion_project/deletions3.html |
| strain, strain background (*S. cerevisiae*) | ynl086wΔ | YSC1053 Yeast MATa deletion collection |  | http://www-sequence.stanford.edu/group/yeast_deletion_project/deletions3.html |
| strain, strain background (*S. cerevisiae*) | ynl091wΔ | YSC1053 Yeast MATa deletion collection |  | http://www-sequence.stanford.edu/group/yeast_deletion_project/deletions3.html |
| strain, strain background (*S. cerevisiae*) | ynl096cΔ | YSC1053 Yeast MATa deletion collection |  | http://www-sequence.stanford.edu/group/yeast_deletion_project/deletions3.html |
| strain, strain background (*S. cerevisiae*) | ynl097cΔ | YSC1053 Yeast MATa deletion collection |  | http://www-sequence.stanford.edu/group/yeast_deletion_project/deletions3.html |
| strain, strain background (*S. cerevisiae*) | ynl098cΔ | YSC1053 Yeast MATa deletion collection |  | http://www-sequence.stanford.edu/group/yeast_deletion_project/deletions3.html |
| strain, strain background (*S. cerevisiae*) | ynl099cΔ | YSC1053 Yeast MATa deletion collection |  | http://www-sequence.stanford.edu/group/yeast_deletion_project/deletions3.html |
| strain, strain background (*S. cerevisiae*) | ynl100wΔ | YSC1053 Yeast MATa deletion collection |  | http://www-sequence.stanford.edu/group/yeast_deletion_project/deletions3.html |
| strain, strain background (*S. cerevisiae*) | ynl105wΔ | YSC1053 Yeast MATa deletion collection |  | http://www-sequence.stanford.edu/group/yeast_deletion_project/deletions3.html |
| strain, strain background (*S. cerevisiae*) | ynl106cΔ | YSC1053 Yeast MATa deletion collection |  | http://www-sequence.stanford.edu/group/yeast_deletion_project/deletions3.html |
| strain, strain background (*S. cerevisiae*) | ynl107wΔ | YSC1053 Yeast MATa deletion collection |  | http://www-sequence.stanford.edu/group/yeast_deletion_project/deletions3.html |
| strain, strain background (*S. cerevisiae*) | ynl109wΔ | YSC1053 Yeast MATa deletion collection |  | http://www-sequence.stanford.edu/group/yeast_deletion_project/deletions3.html |
| strain, strain background (*S. cerevisiae*) | ynl115cΔ | YSC1053 Yeast MATa deletion collection |  | http://www-sequence.stanford.edu/group/yeast_deletion_project/deletions3.html |
| strain, strain background (*S. cerevisiae*) | ynl116wΔ | YSC1053 Yeast MATa deletion collection |  | http://www-sequence.stanford.edu/group/yeast_deletion_project/deletions3.html |
| strain, strain background (*S. cerevisiae*) | ynl119wΔ | YSC1053 Yeast MATa deletion collection |  | http://www-sequence.stanford.edu/group/yeast_deletion_project/deletions3.html |
| strain, strain background (*S. cerevisiae*) | ynl120cΔ | YSC1053 Yeast MATa deletion collection |  | http://www-sequence.stanford.edu/group/yeast_deletion_project/deletions3.html |
| strain, strain background (*S. cerevisiae*) | ynl127wΔ | YSC1053 Yeast MATa deletion collection |  | http://www-sequence.stanford.edu/group/yeast_deletion_project/deletions3.html |
| strain, strain background (*S. cerevisiae*) | ynl128wΔ | YSC1053 Yeast MATa deletion collection |  | http://www-sequence.stanford.edu/group/yeast_deletion_project/deletions3.html |
| strain, strain background (*S. cerevisiae*) | ynl130cΔ | YSC1053 Yeast MATa deletion collection |  | http://www-sequence.stanford.edu/group/yeast_deletion_project/deletions3.html |
| strain, strain background (*S. cerevisiae*) | ynl133cΔ | YSC1053 Yeast MATa deletion collection |  | http://www-sequence.stanford.edu/group/yeast_deletion_project/deletions3.html |
| strain, strain background (*S. cerevisiae*) | ynl134cΔ | YSC1053 Yeast MATa deletion collection |  | http://www-sequence.stanford.edu/group/yeast_deletion_project/deletions3.html |
| strain, strain background (*S. cerevisiae*) | ynl135cΔ | YSC1053 Yeast MATa deletion collection |  | http://www-sequence.stanford.edu/group/yeast_deletion_project/deletions3.html |
| strain, strain background (*S. cerevisiae*) | ynl136wΔ | YSC1053 Yeast MATa deletion collection |  | http://www-sequence.stanford.edu/group/yeast_deletion_project/deletions3.html |
| strain, strain background (*S. cerevisiae*) | ynl138wΔ | YSC1053 Yeast MATa deletion collection |  | http://www-sequence.stanford.edu/group/yeast_deletion_project/deletions3.html |
| strain, strain background (*S. cerevisiae*) | ynl139cΔ | YSC1053 Yeast MATa deletion collection |  | http://www-sequence.stanford.edu/group/yeast_deletion_project/deletions3.html |
| strain, strain background (*S. cerevisiae*) | ynl141wΔ | YSC1053 Yeast MATa deletion collection |  | http://www-sequence.stanford.edu/group/yeast_deletion_project/deletions3.html |
| strain, strain background (*S. cerevisiae*) | ynl142wΔ | YSC1053 Yeast MATa deletion collection |  | http://www-sequence.stanford.edu/group/yeast_deletion_project/deletions3.html |
| strain, strain background (*S. cerevisiae*) | ynl144cΔ | YSC1053 Yeast MATa deletion collection |  | http://www-sequence.stanford.edu/group/yeast_deletion_project/deletions3.html |
| strain, strain background (*S. cerevisiae*) | ynl146wΔ | YSC1053 Yeast MATa deletion collection |  | http://www-sequence.stanford.edu/group/yeast_deletion_project/deletions3.html |
| strain, strain background (*S. cerevisiae*) | ynl147wΔ | YSC1053 Yeast MATa deletion collection |  | http://www-sequence.stanford.edu/group/yeast_deletion_project/deletions3.html |
| strain, strain background (*S. cerevisiae*) | ynl153cΔ | YSC1053 Yeast MATa deletion collection |  | http://www-sequence.stanford.edu/group/yeast_deletion_project/deletions3.html |
| strain, strain background (*S. cerevisiae*) | ynl167cΔ | YSC1053 Yeast MATa deletion collection |  | http://www-sequence.stanford.edu/group/yeast_deletion_project/deletions3.html |
| strain, strain background (*S. cerevisiae*) | ynl170wΔ | YSC1053 Yeast MATa deletion collection |  | http://www-sequence.stanford.edu/group/yeast_deletion_project/deletions3.html |
| strain, strain background (*S. cerevisiae*) | ynl171cΔ | YSC1053 Yeast MATa deletion collection |  | http://www-sequence.stanford.edu/group/yeast_deletion_project/deletions3.html |
| strain, strain background (*S. cerevisiae*) | ynl175cΔ | YSC1053 Yeast MATa deletion collection |  | http://www-sequence.stanford.edu/group/yeast_deletion_project/deletions3.html |
| strain, strain background (*S. cerevisiae*) | ynl176cΔ | YSC1053 Yeast MATa deletion collection |  | http://www-sequence.stanford.edu/group/yeast_deletion_project/deletions3.html |
| strain, strain background (*S. cerevisiae*) | ynl183cΔ | YSC1053 Yeast MATa deletion collection |  | http://www-sequence.stanford.edu/group/yeast_deletion_project/deletions3.html |
| strain, strain background (*S. cerevisiae*) | ynl191wΔ | YSC1053 Yeast MATa deletion collection |  | http://www-sequence.stanford.edu/group/yeast_deletion_project/deletions3.html |
| strain, strain background (*S. cerevisiae*) | ynl196cΔ | YSC1053 Yeast MATa deletion collection |  | http://www-sequence.stanford.edu/group/yeast_deletion_project/deletions3.html |
| strain, strain background (*S. cerevisiae*) | ynl205cΔ | YSC1053 Yeast MATa deletion collection |  | http://www-sequence.stanford.edu/group/yeast_deletion_project/deletions3.html |
| strain, strain background (*S. cerevisiae*) | ynl206cΔ | YSC1053 Yeast MATa deletion collection |  | http://www-sequence.stanford.edu/group/yeast_deletion_project/deletions3.html |
| strain, strain background (*S. cerevisiae*) | ynl211cΔ | YSC1053 Yeast MATa deletion collection |  | http://www-sequence.stanford.edu/group/yeast_deletion_project/deletions3.html |
| strain, strain background (*S. cerevisiae*) | ynl215wΔ | YSC1053 Yeast MATa deletion collection |  | http://www-sequence.stanford.edu/group/yeast_deletion_project/deletions3.html |
| strain, strain background (*S. cerevisiae*) | ynl225cΔ | YSC1053 Yeast MATa deletion collection |  | http://www-sequence.stanford.edu/group/yeast_deletion_project/deletions3.html |
| strain, strain background (*S. cerevisiae*) | ynl227cΔ | YSC1053 Yeast MATa deletion collection |  | http://www-sequence.stanford.edu/group/yeast_deletion_project/deletions3.html |
| strain, strain background (*S. cerevisiae*) | ynl229cΔ | YSC1053 Yeast MATa deletion collection |  | http://www-sequence.stanford.edu/group/yeast_deletion_project/deletions3.html |
| strain, strain background (*S. cerevisiae*) | ynl241cΔ | YSC1053 Yeast MATa deletion collection |  | http://www-sequence.stanford.edu/group/yeast_deletion_project/deletions3.html |
| strain, strain background (*S. cerevisiae*) | ynl246wΔ | YSC1053 Yeast MATa deletion collection |  | http://www-sequence.stanford.edu/group/yeast_deletion_project/deletions3.html |
| strain, strain background (*S. cerevisiae*) | ynl250wΔ | YSC1053 Yeast MATa deletion collection |  | http://www-sequence.stanford.edu/group/yeast_deletion_project/deletions3.html |
| strain, strain background (*S. cerevisiae*) | ynl254cΔ | YSC1053 Yeast MATa deletion collection |  | http://www-sequence.stanford.edu/group/yeast_deletion_project/deletions3.html |
| strain, strain background (*S. cerevisiae*) | ynl270cΔ | YSC1053 Yeast MATa deletion collection |  | http://www-sequence.stanford.edu/group/yeast_deletion_project/deletions3.html |
| strain, strain background (*S. cerevisiae*) | ynl283cΔ | YSC1053 Yeast MATa deletion collection |  | http://www-sequence.stanford.edu/group/yeast_deletion_project/deletions3.html |
| strain, strain background (*S. cerevisiae*) | ynl286wΔ | YSC1053 Yeast MATa deletion collection |  | http://www-sequence.stanford.edu/group/yeast_deletion_project/deletions3.html |
| strain, strain background (*S. cerevisiae*) | ynl302cΔ | YSC1053 Yeast MATa deletion collection |  | http://www-sequence.stanford.edu/group/yeast_deletion_project/deletions3.html |
| strain, strain background (*S. cerevisiae*) | ynr004wΔ | YSC1053 Yeast MATa deletion collection |  | http://www-sequence.stanford.edu/group/yeast_deletion_project/deletions3.html |
| strain, strain background (*S. cerevisiae*) | ynr006wΔ | YSC1053 Yeast MATa deletion collection |  | http://www-sequence.stanford.edu/group/yeast_deletion_project/deletions3.html |
| strain, strain background (*S. cerevisiae*) | ynr020cΔ | YSC1053 Yeast MATa deletion collection |  | http://www-sequence.stanford.edu/group/yeast_deletion_project/deletions3.html |
| strain, strain background (*S. cerevisiae*) | ynr029cΔ | YSC1053 Yeast MATa deletion collection |  | http://www-sequence.stanford.edu/group/yeast_deletion_project/deletions3.html |
| strain, strain background (*S. cerevisiae*) | ynr030wΔ | YSC1053 Yeast MATa deletion collection |  | http://www-sequence.stanford.edu/group/yeast_deletion_project/deletions3.html |
| strain, strain background (*S. cerevisiae*) | ynr031cΔ | YSC1053 Yeast MATa deletion collection |  | http://www-sequence.stanford.edu/group/yeast_deletion_project/deletions3.html |
| strain, strain background (*S. cerevisiae*) | ynr036cΔ | YSC1053 Yeast MATa deletion collection |  | http://www-sequence.stanford.edu/group/yeast_deletion_project/deletions3.html |
| strain, strain background (*S. cerevisiae*) | ynr037cΔ | YSC1053 Yeast MATa deletion collection |  | http://www-sequence.stanford.edu/group/yeast_deletion_project/deletions3.html |
| strain, strain background (*S. cerevisiae*) | ynr041cΔ | YSC1053 Yeast MATa deletion collection |  | http://www-sequence.stanford.edu/group/yeast_deletion_project/deletions3.html |
| strain, strain background (*S. cerevisiae*) | ynr050cΔ | YSC1053 Yeast MATa deletion collection |  | http://www-sequence.stanford.edu/group/yeast_deletion_project/deletions3.html |
| strain, strain background (*S. cerevisiae*) | ynr051cΔ | YSC1053 Yeast MATa deletion collection |  | http://www-sequence.stanford.edu/group/yeast_deletion_project/deletions3.html |
| strain, strain background (*S. cerevisiae*) | ynr052cΔ | YSC1053 Yeast MATa deletion collection |  | http://www-sequence.stanford.edu/group/yeast_deletion_project/deletions3.html |
| strain, strain background (*S. cerevisiae*) | ynr057cΔ | YSC1053 Yeast MATa deletion collection |  | http://www-sequence.stanford.edu/group/yeast_deletion_project/deletions3.html |
| strain, strain background (*S. cerevisiae*) | ynr058wΔ | YSC1053 Yeast MATa deletion collection |  | http://www-sequence.stanford.edu/group/yeast_deletion_project/deletions3.html |
| strain, strain background (*S. cerevisiae*) | yol001wΔ | YSC1053 Yeast MATa deletion collection |  | http://www-sequence.stanford.edu/group/yeast_deletion_project/deletions3.html |
| strain, strain background (*S. cerevisiae*) | yol003cΔ | YSC1053 Yeast MATa deletion collection |  | http://www-sequence.stanford.edu/group/yeast_deletion_project/deletions3.html |
| strain, strain background (*S. cerevisiae*) | yol004wΔ | YSC1053 Yeast MATa deletion collection |  | http://www-sequence.stanford.edu/group/yeast_deletion_project/deletions3.html |
| strain, strain background (*S. cerevisiae*) | yol008wΔ | YSC1053 Yeast MATa deletion collection |  | http://www-sequence.stanford.edu/group/yeast_deletion_project/deletions3.html |
| strain, strain background (*S. cerevisiae*) | yol009cΔ | YSC1053 Yeast MATa deletion collection |  | http://www-sequence.stanford.edu/group/yeast_deletion_project/deletions3.html |
| strain, strain background (*S. cerevisiae*) | yol012cΔ | YSC1053 Yeast MATa deletion collection |  | http://www-sequence.stanford.edu/group/yeast_deletion_project/deletions3.html |
| strain, strain background (*S. cerevisiae*) | yol018cΔ | YSC1053 Yeast MATa deletion collection |  | http://www-sequence.stanford.edu/group/yeast_deletion_project/deletions3.html |
| strain, strain background (*S. cerevisiae*) | yol023wΔ | YSC1053 Yeast MATa deletion collection |  | http://www-sequence.stanford.edu/group/yeast_deletion_project/deletions3.html |
| strain, strain background (*S. cerevisiae*) | yol025wΔ | YSC1053 Yeast MATa deletion collection |  | http://www-sequence.stanford.edu/group/yeast_deletion_project/deletions3.html |
| strain, strain background (*S. cerevisiae*) | yol027cΔ | YSC1053 Yeast MATa deletion collection |  | http://www-sequence.stanford.edu/group/yeast_deletion_project/deletions3.html |
| strain, strain background (*S. cerevisiae*) | yol033wΔ | YSC1053 Yeast MATa deletion collection |  | http://www-sequence.stanford.edu/group/yeast_deletion_project/deletions3.html |
| strain, strain background (*S. cerevisiae*) | yol035cΔ | YSC1053 Yeast MATa deletion collection |  | http://www-sequence.stanford.edu/group/yeast_deletion_project/deletions3.html |
| strain, strain background (*S. cerevisiae*) | yol041cΔ | YSC1053 Yeast MATa deletion collection |  | http://www-sequence.stanford.edu/group/yeast_deletion_project/deletions3.html |
| strain, strain background (*S. cerevisiae*) | yol044wΔ | YSC1053 Yeast MATa deletion collection |  | http://www-sequence.stanford.edu/group/yeast_deletion_project/deletions3.html |
| strain, strain background (*S. cerevisiae*) | yol049wΔ | YSC1053 Yeast MATa deletion collection |  | http://www-sequence.stanford.edu/group/yeast_deletion_project/deletions3.html |
| strain, strain background (*S. cerevisiae*) | yol050cΔ | YSC1053 Yeast MATa deletion collection |  | http://www-sequence.stanford.edu/group/yeast_deletion_project/deletions3.html |
| strain, strain background (*S. cerevisiae*) | yol051wΔ | YSC1053 Yeast MATa deletion collection |  | http://www-sequence.stanford.edu/group/yeast_deletion_project/deletions3.html |
| strain, strain background (*S. cerevisiae*) | yol052cΔ | YSC1053 Yeast MATa deletion collection |  | http://www-sequence.stanford.edu/group/yeast_deletion_project/deletions3.html |
| strain, strain background (*S. cerevisiae*) | yol064cΔ | YSC1053 Yeast MATa deletion collection |  | http://www-sequence.stanford.edu/group/yeast_deletion_project/deletions3.html |
| strain, strain background (*S. cerevisiae*) | yol067cΔ | YSC1053 Yeast MATa deletion collection |  | http://www-sequence.stanford.edu/group/yeast_deletion_project/deletions3.html |
| strain, strain background (*S. cerevisiae*) | yol072wΔ | YSC1053 Yeast MATa deletion collection |  | http://www-sequence.stanford.edu/group/yeast_deletion_project/deletions3.html |
| strain, strain background (*S. cerevisiae*) | yol076wΔ | YSC1053 Yeast MATa deletion collection |  | http://www-sequence.stanford.edu/group/yeast_deletion_project/deletions3.html |
| strain, strain background (*S. cerevisiae*) | yol081wΔ | YSC1053 Yeast MATa deletion collection |  | http://www-sequence.stanford.edu/group/yeast_deletion_project/deletions3.html |
| strain, strain background (*S. cerevisiae*) | yol085cΔ | YSC1053 Yeast MATa deletion collection |  | http://www-sequence.stanford.edu/group/yeast_deletion_project/deletions3.html |
| strain, strain background (*S. cerevisiae*) | yol086cΔ | YSC1053 Yeast MATa deletion collection |  | http://www-sequence.stanford.edu/group/yeast_deletion_project/deletions3.html |
| strain, strain background (*S. cerevisiae*) | yol087cΔ | YSC1053 Yeast MATa deletion collection |  | http://www-sequence.stanford.edu/group/yeast_deletion_project/deletions3.html |
| strain, strain background (*S. cerevisiae*) | yol088cΔ | YSC1053 Yeast MATa deletion collection |  | http://www-sequence.stanford.edu/group/yeast_deletion_project/deletions3.html |
| strain, strain background (*S. cerevisiae*) | yol090wΔ | YSC1053 Yeast MATa deletion collection |  | http://www-sequence.stanford.edu/group/yeast_deletion_project/deletions3.html |
| strain, strain background (*S. cerevisiae*) | yol095cΔ | YSC1053 Yeast MATa deletion collection |  | http://www-sequence.stanford.edu/group/yeast_deletion_project/deletions3.html |
| strain, strain background (*S. cerevisiae*) | yol096cΔ | YSC1053 Yeast MATa deletion collection |  | http://www-sequence.stanford.edu/group/yeast_deletion_project/deletions3.html |
| strain, strain background (*S. cerevisiae*) | yol108cΔ | YSC1053 Yeast MATa deletion collection |  | http://www-sequence.stanford.edu/group/yeast_deletion_project/deletions3.html |
| strain, strain background (*S. cerevisiae*) | yol110wΔ | YSC1053 Yeast MATa deletion collection |  | http://www-sequence.stanford.edu/group/yeast_deletion_project/deletions3.html |
| strain, strain background (*S. cerevisiae*) | yol115wΔ | YSC1053 Yeast MATa deletion collection |  | http://www-sequence.stanford.edu/group/yeast_deletion_project/deletions3.html |
| strain, strain background (*S. cerevisiae*) | yol116wΔ | YSC1053 Yeast MATa deletion collection |  | http://www-sequence.stanford.edu/group/yeast_deletion_project/deletions3.html |
| strain, strain background (*S. cerevisiae*) | yol121cΔ | YSC1053 Yeast MATa deletion collection |  | http://www-sequence.stanford.edu/group/yeast_deletion_project/deletions3.html |
| strain, strain background (*S. cerevisiae*) | yol140wΔ | YSC1053 Yeast MATa deletion collection |  | http://www-sequence.stanford.edu/group/yeast_deletion_project/deletions3.html |
| strain, strain background (*S. cerevisiae*) | yol143cΔ | YSC1053 Yeast MATa deletion collection |  | http://www-sequence.stanford.edu/group/yeast_deletion_project/deletions3.html |
| strain, strain background (*S. cerevisiae*) | yol145cΔ | YSC1053 Yeast MATa deletion collection |  | http://www-sequence.stanford.edu/group/yeast_deletion_project/deletions3.html |
| strain, strain background (*S. cerevisiae*) | yor001wΔ | YSC1053 Yeast MATa deletion collection |  | http://www-sequence.stanford.edu/group/yeast_deletion_project/deletions3.html |
| strain, strain background (*S. cerevisiae*) | yor008cΔ | YSC1053 Yeast MATa deletion collection |  | http://www-sequence.stanford.edu/group/yeast_deletion_project/deletions3.html |
| strain, strain background (*S. cerevisiae*) | yor008c-aΔ | YSC1053 Yeast MATa deletion collection |  | http://www-sequence.stanford.edu/group/yeast_deletion_project/deletions3.html |
| strain, strain background (*S. cerevisiae*) | yor014wΔ | YSC1053 Yeast MATa deletion collection |  | http://www-sequence.stanford.edu/group/yeast_deletion_project/deletions3.html |
| strain, strain background (*S. cerevisiae*) | yor026wΔ | YSC1053 Yeast MATa deletion collection |  | http://www-sequence.stanford.edu/group/yeast_deletion_project/deletions3.html |
| strain, strain background (*S. cerevisiae*) | yor028cΔ | YSC1053 Yeast MATa deletion collection |  | http://www-sequence.stanford.edu/group/yeast_deletion_project/deletions3.html |
| strain, strain background (*S. cerevisiae*) | yor029wΔ | YSC1053 Yeast MATa deletion collection |  | http://www-sequence.stanford.edu/group/yeast_deletion_project/deletions3.html |
| strain, strain background (*S. cerevisiae*) | yor030wΔ | YSC1053 Yeast MATa deletion collection |  | http://www-sequence.stanford.edu/group/yeast_deletion_project/deletions3.html |
| strain, strain background (*S. cerevisiae*) | yor033cΔ | YSC1053 Yeast MATa deletion collection |  | http://www-sequence.stanford.edu/group/yeast_deletion_project/deletions3.html |
| strain, strain background (*S. cerevisiae*) | yor035cΔ | YSC1053 Yeast MATa deletion collection |  | http://www-sequence.stanford.edu/group/yeast_deletion_project/deletions3.html |
| strain, strain background (*S. cerevisiae*) | yor036wΔ | YSC1053 Yeast MATa deletion collection |  | http://www-sequence.stanford.edu/group/yeast_deletion_project/deletions3.html |
| strain, strain background (*S. cerevisiae*) | yor037wΔ | YSC1053 Yeast MATa deletion collection |  | http://www-sequence.stanford.edu/group/yeast_deletion_project/deletions3.html |
| strain, strain background (*S. cerevisiae*) | yor038cΔ | YSC1053 Yeast MATa deletion collection |  | http://www-sequence.stanford.edu/group/yeast_deletion_project/deletions3.html |
| strain, strain background (*S. cerevisiae*) | yor039wΔ | YSC1053 Yeast MATa deletion collection |  | http://www-sequence.stanford.edu/group/yeast_deletion_project/deletions3.html |
| strain, strain background (*S. cerevisiae*) | yor042wΔ | YSC1053 Yeast MATa deletion collection |  | http://www-sequence.stanford.edu/group/yeast_deletion_project/deletions3.html |
| strain, strain background (*S. cerevisiae*) | yor043wΔ | YSC1053 Yeast MATa deletion collection |  | http://www-sequence.stanford.edu/group/yeast_deletion_project/deletions3.html |
| strain, strain background (*S. cerevisiae*) | yor049cΔ | YSC1053 Yeast MATa deletion collection |  | http://www-sequence.stanford.edu/group/yeast_deletion_project/deletions3.html |
| strain, strain background (*S. cerevisiae*) | yor051cΔ | YSC1053 Yeast MATa deletion collection |  | http://www-sequence.stanford.edu/group/yeast_deletion_project/deletions3.html |
| strain, strain background (*S. cerevisiae*) | yor052cΔ | YSC1053 Yeast MATa deletion collection |  | http://www-sequence.stanford.edu/group/yeast_deletion_project/deletions3.html |
| strain, strain background (*S. cerevisiae*) | yor054cΔ | YSC1053 Yeast MATa deletion collection |  | http://www-sequence.stanford.edu/group/yeast_deletion_project/deletions3.html |
| strain, strain background (*S. cerevisiae*) | yor062cΔ | YSC1053 Yeast MATa deletion collection |  | http://www-sequence.stanford.edu/group/yeast_deletion_project/deletions3.html |
| strain, strain background (*S. cerevisiae*) | yor065wΔ | YSC1053 Yeast MATa deletion collection |  | http://www-sequence.stanford.edu/group/yeast_deletion_project/deletions3.html |
| strain, strain background (*S. cerevisiae*) | yor068cΔ | YSC1053 Yeast MATa deletion collection |  | http://www-sequence.stanford.edu/group/yeast_deletion_project/deletions3.html |
| strain, strain background (*S. cerevisiae*) | yor069wΔ | YSC1053 Yeast MATa deletion collection |  | http://www-sequence.stanford.edu/group/yeast_deletion_project/deletions3.html |
| strain, strain background (*S. cerevisiae*) | yor070cΔ | YSC1053 Yeast MATa deletion collection |  | http://www-sequence.stanford.edu/group/yeast_deletion_project/deletions3.html |
| strain, strain background (*S. cerevisiae*) | yor078wΔ | YSC1053 Yeast MATa deletion collection |  | http://www-sequence.stanford.edu/group/yeast_deletion_project/deletions3.html |
| strain, strain background (*S. cerevisiae*) | yor080wΔ | YSC1053 Yeast MATa deletion collection |  | http://www-sequence.stanford.edu/group/yeast_deletion_project/deletions3.html |
| strain, strain background (*S. cerevisiae*) | yor081cΔ | YSC1053 Yeast MATa deletion collection |  | http://www-sequence.stanford.edu/group/yeast_deletion_project/deletions3.html |
| strain, strain background (*S. cerevisiae*) | yor083wΔ | YSC1053 Yeast MATa deletion collection |  | http://www-sequence.stanford.edu/group/yeast_deletion_project/deletions3.html |
| strain, strain background (*S. cerevisiae*) | yor085wΔ | YSC1053 Yeast MATa deletion collection |  | http://www-sequence.stanford.edu/group/yeast_deletion_project/deletions3.html |
| strain, strain background (*S. cerevisiae*) | yor089cΔ | YSC1053 Yeast MATa deletion collection |  | http://www-sequence.stanford.edu/group/yeast_deletion_project/deletions3.html |
| strain, strain background (*S. cerevisiae*) | yor096wΔ | YSC1053 Yeast MATa deletion collection |  | http://www-sequence.stanford.edu/group/yeast_deletion_project/deletions3.html |
| strain, strain background (*S. cerevisiae*) | yor097cΔ | YSC1053 Yeast MATa deletion collection |  | http://www-sequence.stanford.edu/group/yeast_deletion_project/deletions3.html |
| strain, strain background (*S. cerevisiae*) | yor100cΔ | YSC1053 Yeast MATa deletion collection |  | http://www-sequence.stanford.edu/group/yeast_deletion_project/deletions3.html |
| strain, strain background (*S. cerevisiae*) | yor106wΔ | YSC1053 Yeast MATa deletion collection |  | http://www-sequence.stanford.edu/group/yeast_deletion_project/deletions3.html |
| strain, strain background (*S. cerevisiae*) | yor107wΔ | YSC1053 Yeast MATa deletion collection |  | http://www-sequence.stanford.edu/group/yeast_deletion_project/deletions3.html |
| strain, strain background (*S. cerevisiae*) | yor123cΔ | YSC1053 Yeast MATa deletion collection |  | http://www-sequence.stanford.edu/group/yeast_deletion_project/deletions3.html |
| strain, strain background (*S. cerevisiae*) | yor124cΔ | YSC1053 Yeast MATa deletion collection |  | http://www-sequence.stanford.edu/group/yeast_deletion_project/deletions3.html |
| strain, strain background (*S. cerevisiae*) | yor129cΔ | YSC1053 Yeast MATa deletion collection |  | http://www-sequence.stanford.edu/group/yeast_deletion_project/deletions3.html |
| strain, strain background (*S. cerevisiae*) | yor130cΔ | YSC1053 Yeast MATa deletion collection |  | http://www-sequence.stanford.edu/group/yeast_deletion_project/deletions3.html |
| strain, strain background (*S. cerevisiae*) | yor132wΔ | YSC1053 Yeast MATa deletion collection |  | http://www-sequence.stanford.edu/group/yeast_deletion_project/deletions3.html |
| strain, strain background (*S. cerevisiae*) | yor135cΔ | YSC1053 Yeast MATa deletion collection |  | http://www-sequence.stanford.edu/group/yeast_deletion_project/deletions3.html |
| strain, strain background (*S. cerevisiae*) | yor136wΔ | YSC1053 Yeast MATa deletion collection |  | http://www-sequence.stanford.edu/group/yeast_deletion_project/deletions3.html |
| strain, strain background (*S. cerevisiae*) | yor138cΔ | YSC1053 Yeast MATa deletion collection |  | http://www-sequence.stanford.edu/group/yeast_deletion_project/deletions3.html |
| strain, strain background (*S. cerevisiae*) | yor139cΔ | YSC1053 Yeast MATa deletion collection |  | http://www-sequence.stanford.edu/group/yeast_deletion_project/deletions3.html |
| strain, strain background (*S. cerevisiae*) | yor140wΔ | YSC1053 Yeast MATa deletion collection |  | http://www-sequence.stanford.edu/group/yeast_deletion_project/deletions3.html |
| strain, strain background (*S. cerevisiae*) | yor141cΔ | YSC1053 Yeast MATa deletion collection |  | http://www-sequence.stanford.edu/group/yeast_deletion_project/deletions3.html |
| strain, strain background (*S. cerevisiae*) | yor150wΔ | YSC1053 Yeast MATa deletion collection |  | http://www-sequence.stanford.edu/group/yeast_deletion_project/deletions3.html |
| strain, strain background (*S. cerevisiae*) | yor158wΔ | YSC1053 Yeast MATa deletion collection |  | http://www-sequence.stanford.edu/group/yeast_deletion_project/deletions3.html |
| strain, strain background (*S. cerevisiae*) | yor161cΔ | YSC1053 Yeast MATa deletion collection |  | http://www-sequence.stanford.edu/group/yeast_deletion_project/deletions3.html |
| strain, strain background (*S. cerevisiae*) | yor173wΔ | YSC1053 Yeast MATa deletion collection |  | http://www-sequence.stanford.edu/group/yeast_deletion_project/deletions3.html |
| strain, strain background (*S. cerevisiae*) | yor178cΔ | YSC1053 Yeast MATa deletion collection |  | http://www-sequence.stanford.edu/group/yeast_deletion_project/deletions3.html |
| strain, strain background (*S. cerevisiae*) | yor182cΔ | YSC1053 Yeast MATa deletion collection |  | http://www-sequence.stanford.edu/group/yeast_deletion_project/deletions3.html |
| strain, strain background (*S. cerevisiae*) | yor183wΔ | YSC1053 Yeast MATa deletion collection |  | http://www-sequence.stanford.edu/group/yeast_deletion_project/deletions3.html |
| strain, strain background (*S. cerevisiae*) | yor184wΔ | YSC1053 Yeast MATa deletion collection |  | http://www-sequence.stanford.edu/group/yeast_deletion_project/deletions3.html |
| strain, strain background (*S. cerevisiae*) | yor186wΔ | YSC1053 Yeast MATa deletion collection |  | http://www-sequence.stanford.edu/group/yeast_deletion_project/deletions3.html |
| strain, strain background (*S. cerevisiae*) | yor187wΔ | YSC1053 Yeast MATa deletion collection |  | http://www-sequence.stanford.edu/group/yeast_deletion_project/deletions3.html |
| strain, strain background (*S. cerevisiae*) | yor195wΔ | YSC1053 Yeast MATa deletion collection |  | http://www-sequence.stanford.edu/group/yeast_deletion_project/deletions3.html |
| strain, strain background (*S. cerevisiae*) | yor198cΔ | YSC1053 Yeast MATa deletion collection |  | http://www-sequence.stanford.edu/group/yeast_deletion_project/deletions3.html |
| strain, strain background (*S. cerevisiae*) | yor201cΔ | YSC1053 Yeast MATa deletion collection |  | http://www-sequence.stanford.edu/group/yeast_deletion_project/deletions3.html |
| strain, strain background (*S. cerevisiae*) | yor205cΔ | YSC1053 Yeast MATa deletion collection |  | http://www-sequence.stanford.edu/group/yeast_deletion_project/deletions3.html |
| strain, strain background (*S. cerevisiae*) | yor211cΔ | YSC1053 Yeast MATa deletion collection |  | http://www-sequence.stanford.edu/group/yeast_deletion_project/deletions3.html |
| strain, strain background (*S. cerevisiae*) | yor212wΔ | YSC1053 Yeast MATa deletion collection |  | http://www-sequence.stanford.edu/group/yeast_deletion_project/deletions3.html |
| strain, strain background (*S. cerevisiae*) | yor219cΔ | YSC1053 Yeast MATa deletion collection |  | http://www-sequence.stanford.edu/group/yeast_deletion_project/deletions3.html |
| strain, strain background (*S. cerevisiae*) | yor220wΔ | YSC1053 Yeast MATa deletion collection |  | http://www-sequence.stanford.edu/group/yeast_deletion_project/deletions3.html |
| strain, strain background (*S. cerevisiae*) | yor221cΔ | YSC1053 Yeast MATa deletion collection |  | http://www-sequence.stanford.edu/group/yeast_deletion_project/deletions3.html |
| strain, strain background (*S. cerevisiae*) | yor225wΔ | YSC1053 Yeast MATa deletion collection |  | http://www-sequence.stanford.edu/group/yeast_deletion_project/deletions3.html |
| strain, strain background (*S. cerevisiae*) | yor226cΔ | YSC1053 Yeast MATa deletion collection |  | http://www-sequence.stanford.edu/group/yeast_deletion_project/deletions3.html |
| strain, strain background (*S. cerevisiae*) | yor233wΔ | YSC1053 Yeast MATa deletion collection |  | http://www-sequence.stanford.edu/group/yeast_deletion_project/deletions3.html |
| strain, strain background (*S. cerevisiae*) | yor235wΔ | YSC1053 Yeast MATa deletion collection |  | http://www-sequence.stanford.edu/group/yeast_deletion_project/deletions3.html |
| strain, strain background (*S. cerevisiae*) | yor241wΔ | YSC1053 Yeast MATa deletion collection |  | http://www-sequence.stanford.edu/group/yeast_deletion_project/deletions3.html |
| strain, strain background (*S. cerevisiae*) | yor269wΔ | YSC1053 Yeast MATa deletion collection |  | http://www-sequence.stanford.edu/group/yeast_deletion_project/deletions3.html |
| strain, strain background (*S. cerevisiae*) | yor270cΔ | YSC1053 Yeast MATa deletion collection |  | http://www-sequence.stanford.edu/group/yeast_deletion_project/deletions3.html |
| strain, strain background (*S. cerevisiae*) | yor275cΔ | YSC1053 Yeast MATa deletion collection |  | http://www-sequence.stanford.edu/group/yeast_deletion_project/deletions3.html |
| strain, strain background (*S. cerevisiae*) | yor277cΔ | YSC1053 Yeast MATa deletion collection |  | http://www-sequence.stanford.edu/group/yeast_deletion_project/deletions3.html |
| strain, strain background (*S. cerevisiae*) | yor290cΔ | YSC1053 Yeast MATa deletion collection |  | http://www-sequence.stanford.edu/group/yeast_deletion_project/deletions3.html |
| strain, strain background (*S. cerevisiae*) | yor293wΔ | YSC1053 Yeast MATa deletion collection |  | http://www-sequence.stanford.edu/group/yeast_deletion_project/deletions3.html |
| strain, strain background (*S. cerevisiae*) | yor298c-aΔ | YSC1053 Yeast MATa deletion collection |  | http://www-sequence.stanford.edu/group/yeast_deletion_project/deletions3.html |
| strain, strain background (*S. cerevisiae*) | yor304wΔ | YSC1053 Yeast MATa deletion collection |  | http://www-sequence.stanford.edu/group/yeast_deletion_project/deletions3.html |
| strain, strain background (*S. cerevisiae*) | yor305wΔ | YSC1053 Yeast MATa deletion collection |  | http://www-sequence.stanford.edu/group/yeast_deletion_project/deletions3.html |
| strain, strain background (*S. cerevisiae*) | yor309cΔ | YSC1053 Yeast MATa deletion collection |  | http://www-sequence.stanford.edu/group/yeast_deletion_project/deletions3.html |
| strain, strain background (*S. cerevisiae*) | yor322cΔ | YSC1053 Yeast MATa deletion collection |  | http://www-sequence.stanford.edu/group/yeast_deletion_project/deletions3.html |
| strain, strain background (*S. cerevisiae*) | yor323cΔ | YSC1053 Yeast MATa deletion collection |  | http://www-sequence.stanford.edu/group/yeast_deletion_project/deletions3.html |
| strain, strain background (*S. cerevisiae*) | yor327cΔ | YSC1053 Yeast MATa deletion collection |  | http://www-sequence.stanford.edu/group/yeast_deletion_project/deletions3.html |
| strain, strain background (*S. cerevisiae*) | yor330cΔ | YSC1053 Yeast MATa deletion collection |  | http://www-sequence.stanford.edu/group/yeast_deletion_project/deletions3.html |
| strain, strain background (*S. cerevisiae*) | yor332wΔ | YSC1053 Yeast MATa deletion collection |  | http://www-sequence.stanford.edu/group/yeast_deletion_project/deletions3.html |
| strain, strain background (*S. cerevisiae*) | yor339cΔ | YSC1053 Yeast MATa deletion collection |  | http://www-sequence.stanford.edu/group/yeast_deletion_project/deletions3.html |
| strain, strain background (*S. cerevisiae*) | yor350cΔ | YSC1053 Yeast MATa deletion collection |  | http://www-sequence.stanford.edu/group/yeast_deletion_project/deletions3.html |
| strain, strain background (*S. cerevisiae*) | yor358wΔ | YSC1053 Yeast MATa deletion collection |  | http://www-sequence.stanford.edu/group/yeast_deletion_project/deletions3.html |
| strain, strain background (*S. cerevisiae*) | yor359wΔ | YSC1053 Yeast MATa deletion collection |  | http://www-sequence.stanford.edu/group/yeast_deletion_project/deletions3.html |
| strain, strain background (*S. cerevisiae*) | yor360cΔ | YSC1053 Yeast MATa deletion collection |  | http://www-sequence.stanford.edu/group/yeast_deletion_project/deletions3.html |
| strain, strain background (*S. cerevisiae*) | yor364wΔ | YSC1053 Yeast MATa deletion collection |  | http://www-sequence.stanford.edu/group/yeast_deletion_project/deletions3.html |
| strain, strain background (*S. cerevisiae*) | yor371cΔ | YSC1053 Yeast MATa deletion collection |  | http://www-sequence.stanford.edu/group/yeast_deletion_project/deletions3.html |
| strain, strain background (*S. cerevisiae*) | yor376w-aΔ | YSC1053 Yeast MATa deletion collection |  | http://www-sequence.stanford.edu/group/yeast_deletion_project/deletions3.html |
| strain, strain background (*S. cerevisiae*) | yor378wΔ | YSC1053 Yeast MATa deletion collection |  | http://www-sequence.stanford.edu/group/yeast_deletion_project/deletions3.html |
| strain, strain background (*S. cerevisiae*) | ypl002cΔ | YSC1053 Yeast MATa deletion collection |  | http://www-sequence.stanford.edu/group/yeast_deletion_project/deletions3.html |
| strain, strain background (*S. cerevisiae*) | ypl005wΔ | YSC1053 Yeast MATa deletion collection |  | http://www-sequence.stanford.edu/group/yeast_deletion_project/deletions3.html |
| strain, strain background (*S. cerevisiae*) | ypl013cΔ | YSC1053 Yeast MATa deletion collection |  | http://www-sequence.stanford.edu/group/yeast_deletion_project/deletions3.html |
| strain, strain background (*S. cerevisiae*) | ypl024wΔ | YSC1053 Yeast MATa deletion collection |  | http://www-sequence.stanford.edu/group/yeast_deletion_project/deletions3.html |
| strain, strain background (*S. cerevisiae*) | ypl029wΔ | YSC1053 Yeast MATa deletion collection |  | http://www-sequence.stanford.edu/group/yeast_deletion_project/deletions3.html |
| strain, strain background (*S. cerevisiae*) | ypl031cΔ | YSC1053 Yeast MATa deletion collection |  | http://www-sequence.stanford.edu/group/yeast_deletion_project/deletions3.html |
| strain, strain background (*S. cerevisiae*) | ypl036wΔ | YSC1053 Yeast MATa deletion collection |  | http://www-sequence.stanford.edu/group/yeast_deletion_project/deletions3.html |
| strain, strain background (*S. cerevisiae*) | ypl037cΔ | YSC1053 Yeast MATa deletion collection |  | http://www-sequence.stanford.edu/group/yeast_deletion_project/deletions3.html |
| strain, strain background (*S. cerevisiae*) | ypl040cΔ | YSC1053 Yeast MATa deletion collection |  | http://www-sequence.stanford.edu/group/yeast_deletion_project/deletions3.html |
| strain, strain background (*S. cerevisiae*) | ypl042cΔ | YSC1053 Yeast MATa deletion collection |  | http://www-sequence.stanford.edu/group/yeast_deletion_project/deletions3.html |
| strain, strain background (*S. cerevisiae*) | ypl045wΔ | YSC1053 Yeast MATa deletion collection |  | http://www-sequence.stanford.edu/group/yeast_deletion_project/deletions3.html |
| strain, strain background (*S. cerevisiae*) | ypl047wΔ | YSC1053 Yeast MATa deletion collection |  | http://www-sequence.stanford.edu/group/yeast_deletion_project/deletions3.html |
| strain, strain background (*S. cerevisiae*) | ypl049cΔ | YSC1053 Yeast MATa deletion collection |  | http://www-sequence.stanford.edu/group/yeast_deletion_project/deletions3.html |
| strain, strain background (*S. cerevisiae*) | ypl050cΔ | YSC1053 Yeast MATa deletion collection |  | http://www-sequence.stanford.edu/group/yeast_deletion_project/deletions3.html |
| strain, strain background (*S. cerevisiae*) | ypl055cΔ | YSC1053 Yeast MATa deletion collection |  | http://www-sequence.stanford.edu/group/yeast_deletion_project/deletions3.html |
| strain, strain background (*S. cerevisiae*) | ypl056cΔ | YSC1053 Yeast MATa deletion collection |  | http://www-sequence.stanford.edu/group/yeast_deletion_project/deletions3.html |
| strain, strain background (*S. cerevisiae*) | ypl057cΔ | YSC1053 Yeast MATa deletion collection |  | http://www-sequence.stanford.edu/group/yeast_deletion_project/deletions3.html |
| strain, strain background (*S. cerevisiae*) | ypl059wΔ | YSC1053 Yeast MATa deletion collection |  | http://www-sequence.stanford.edu/group/yeast_deletion_project/deletions3.html |
| strain, strain background (*S. cerevisiae*) | ypl061wΔ | YSC1053 Yeast MATa deletion collection |  | http://www-sequence.stanford.edu/group/yeast_deletion_project/deletions3.html |
| strain, strain background (*S. cerevisiae*) | ypl062wΔ | YSC1053 Yeast MATa deletion collection |  | http://www-sequence.stanford.edu/group/yeast_deletion_project/deletions3.html |
| strain, strain background (*S. cerevisiae*) | ypl065wΔ | YSC1053 Yeast MATa deletion collection |  | http://www-sequence.stanford.edu/group/yeast_deletion_project/deletions3.html |
| strain, strain background (*S. cerevisiae*) | ypl069cΔ | YSC1053 Yeast MATa deletion collection |  | http://www-sequence.stanford.edu/group/yeast_deletion_project/deletions3.html |
| strain, strain background (*S. cerevisiae*) | ypl078cΔ | YSC1053 Yeast MATa deletion collection |  | http://www-sequence.stanford.edu/group/yeast_deletion_project/deletions3.html |
| strain, strain background (*S. cerevisiae*) | ypl079wΔ | YSC1053 Yeast MATa deletion collection |  | http://www-sequence.stanford.edu/group/yeast_deletion_project/deletions3.html |
| strain, strain background (*S. cerevisiae*) | ypl080cΔ | YSC1053 Yeast MATa deletion collection |  | http://www-sequence.stanford.edu/group/yeast_deletion_project/deletions3.html |
| strain, strain background (*S. cerevisiae*) | ypl084wΔ | YSC1053 Yeast MATa deletion collection |  | http://www-sequence.stanford.edu/group/yeast_deletion_project/deletions3.html |
| strain, strain background (*S. cerevisiae*) | ypl086cΔ | YSC1053 Yeast MATa deletion collection |  | http://www-sequence.stanford.edu/group/yeast_deletion_project/deletions3.html |
| strain, strain background (*S. cerevisiae*) | ypl089cΔ | YSC1053 Yeast MATa deletion collection |  | http://www-sequence.stanford.edu/group/yeast_deletion_project/deletions3.html |
| strain, strain background (*S. cerevisiae*) | ypl090cΔ | YSC1053 Yeast MATa deletion collection |  | http://www-sequence.stanford.edu/group/yeast_deletion_project/deletions3.html |
| strain, strain background (*S. cerevisiae*) | ypl096c-aΔ | YSC1053 Yeast MATa deletion collection |  | http://www-sequence.stanford.edu/group/yeast_deletion_project/deletions3.html |
| strain, strain background (*S. cerevisiae*) | ypl097wΔ | YSC1053 Yeast MATa deletion collection |  | http://www-sequence.stanford.edu/group/yeast_deletion_project/deletions3.html |
| strain, strain background (*S. cerevisiae*) | ypl098cΔ | YSC1053 Yeast MATa deletion collection |  | http://www-sequence.stanford.edu/group/yeast_deletion_project/deletions3.html |
| strain, strain background (*S. cerevisiae*) | ypl101wΔ | YSC1053 Yeast MATa deletion collection |  | http://www-sequence.stanford.edu/group/yeast_deletion_project/deletions3.html |
| strain, strain background (*S. cerevisiae*) | ypl102cΔ | YSC1053 Yeast MATa deletion collection |  | http://www-sequence.stanford.edu/group/yeast_deletion_project/deletions3.html |
| strain, strain background (*S. cerevisiae*) | ypl104wΔ | YSC1053 Yeast MATa deletion collection |  | http://www-sequence.stanford.edu/group/yeast_deletion_project/deletions3.html |
| strain, strain background (*S. cerevisiae*) | ypl106cΔ | YSC1053 Yeast MATa deletion collection |  | http://www-sequence.stanford.edu/group/yeast_deletion_project/deletions3.html |
| strain, strain background (*S. cerevisiae*) | ypl113cΔ | YSC1053 Yeast MATa deletion collection |  | http://www-sequence.stanford.edu/group/yeast_deletion_project/deletions3.html |
| strain, strain background (*S. cerevisiae*) | ypl118wΔ | YSC1053 Yeast MATa deletion collection |  | http://www-sequence.stanford.edu/group/yeast_deletion_project/deletions3.html |
| strain, strain background (*S. cerevisiae*) | ypl119cΔ | YSC1053 Yeast MATa deletion collection |  | http://www-sequence.stanford.edu/group/yeast_deletion_project/deletions3.html |
| strain, strain background (*S. cerevisiae*) | ypl120wΔ | YSC1053 Yeast MATa deletion collection |  | http://www-sequence.stanford.edu/group/yeast_deletion_project/deletions3.html |
| strain, strain background (*S. cerevisiae*) | ypl121cΔ | YSC1053 Yeast MATa deletion collection |  | http://www-sequence.stanford.edu/group/yeast_deletion_project/deletions3.html |
| strain, strain background (*S. cerevisiae*) | ypl123cΔ | YSC1053 Yeast MATa deletion collection |  | http://www-sequence.stanford.edu/group/yeast_deletion_project/deletions3.html |
| strain, strain background (*S. cerevisiae*) | ypl129wΔ | YSC1053 Yeast MATa deletion collection |  | http://www-sequence.stanford.edu/group/yeast_deletion_project/deletions3.html |
| strain, strain background (*S. cerevisiae*) | ypl132wΔ | YSC1053 Yeast MATa deletion collection |  | http://www-sequence.stanford.edu/group/yeast_deletion_project/deletions3.html |
| strain, strain background (*S. cerevisiae*) | ypl135wΔ | YSC1053 Yeast MATa deletion collection |  | http://www-sequence.stanford.edu/group/yeast_deletion_project/deletions3.html |
| strain, strain background (*S. cerevisiae*) | ypl144wΔ | YSC1053 Yeast MATa deletion collection |  | http://www-sequence.stanford.edu/group/yeast_deletion_project/deletions3.html |
| strain, strain background (*S. cerevisiae*) | ypl148cΔ | YSC1053 Yeast MATa deletion collection |  | http://www-sequence.stanford.edu/group/yeast_deletion_project/deletions3.html |
| strain, strain background (*S. cerevisiae*) | ypl155cΔ | YSC1053 Yeast MATa deletion collection |  | http://www-sequence.stanford.edu/group/yeast_deletion_project/deletions3.html |
| strain, strain background (*S. cerevisiae*) | ypl157wΔ | YSC1053 Yeast MATa deletion collection |  | http://www-sequence.stanford.edu/group/yeast_deletion_project/deletions3.html |
| strain, strain background (*S. cerevisiae*) | ypl158cΔ | YSC1053 Yeast MATa deletion collection |  | http://www-sequence.stanford.edu/group/yeast_deletion_project/deletions3.html |
| strain, strain background (*S. cerevisiae*) | ypl161cΔ | YSC1053 Yeast MATa deletion collection |  | http://www-sequence.stanford.edu/group/yeast_deletion_project/deletions3.html |
| strain, strain background (*S. cerevisiae*) | ypl165cΔ | YSC1053 Yeast MATa deletion collection |  | http://www-sequence.stanford.edu/group/yeast_deletion_project/deletions3.html |
| strain, strain background (*S. cerevisiae*) | ypl167cΔ | YSC1053 Yeast MATa deletion collection |  | http://www-sequence.stanford.edu/group/yeast_deletion_project/deletions3.html |
| strain, strain background (*S. cerevisiae*) | ypl172cΔ | YSC1053 Yeast MATa deletion collection |  | http://www-sequence.stanford.edu/group/yeast_deletion_project/deletions3.html |
| strain, strain background (*S. cerevisiae*) | ypl173wΔ | YSC1053 Yeast MATa deletion collection |  | http://www-sequence.stanford.edu/group/yeast_deletion_project/deletions3.html |
| strain, strain background (*S. cerevisiae*) | ypl174cΔ | YSC1053 Yeast MATa deletion collection |  | http://www-sequence.stanford.edu/group/yeast_deletion_project/deletions3.html |
| strain, strain background (*S. cerevisiae*) | ypl178wΔ | YSC1053 Yeast MATa deletion collection |  | http://www-sequence.stanford.edu/group/yeast_deletion_project/deletions3.html |
| strain, strain background (*S. cerevisiae*) | ypl179wΔ | YSC1053 Yeast MATa deletion collection |  | http://www-sequence.stanford.edu/group/yeast_deletion_project/deletions3.html |
| strain, strain background (*S. cerevisiae*) | ypl180wΔ | YSC1053 Yeast MATa deletion collection |  | http://www-sequence.stanford.edu/group/yeast_deletion_project/deletions3.html |
| strain, strain background (*S. cerevisiae*) | ypl183w-aΔ | YSC1053 Yeast MATa deletion collection |  | http://www-sequence.stanford.edu/group/yeast_deletion_project/deletions3.html |
| strain, strain background (*S. cerevisiae*) | ypl184cΔ | YSC1053 Yeast MATa deletion collection |  | http://www-sequence.stanford.edu/group/yeast_deletion_project/deletions3.html |
| strain, strain background (*S. cerevisiae*) | ypl188wΔ | YSC1053 Yeast MATa deletion collection |  | http://www-sequence.stanford.edu/group/yeast_deletion_project/deletions3.html |
| strain, strain background (*S. cerevisiae*) | ypl189c-aΔ | YSC1053 Yeast MATa deletion collection |  | http://www-sequence.stanford.edu/group/yeast_deletion_project/deletions3.html |
| strain, strain background (*S. cerevisiae*) | ypl202cΔ | YSC1053 Yeast MATa deletion collection |  | http://www-sequence.stanford.edu/group/yeast_deletion_project/deletions3.html |
| strain, strain background (*S. cerevisiae*) | ypl203wΔ | YSC1053 Yeast MATa deletion collection |  | http://www-sequence.stanford.edu/group/yeast_deletion_project/deletions3.html |
| strain, strain background (*S. cerevisiae*) | ypl205cΔ | YSC1053 Yeast MATa deletion collection |  | http://www-sequence.stanford.edu/group/yeast_deletion_project/deletions3.html |
| strain, strain background (*S. cerevisiae*) | ypl206cΔ | YSC1053 Yeast MATa deletion collection |  | http://www-sequence.stanford.edu/group/yeast_deletion_project/deletions3.html |
| strain, strain background (*S. cerevisiae*) | ypl213wΔ | YSC1053 Yeast MATa deletion collection |  | http://www-sequence.stanford.edu/group/yeast_deletion_project/deletions3.html |
| strain, strain background (*S. cerevisiae*) | ypl215wΔ | YSC1053 Yeast MATa deletion collection |  | http://www-sequence.stanford.edu/group/yeast_deletion_project/deletions3.html |
| strain, strain background (*S. cerevisiae*) | ypl221wΔ | YSC1053 Yeast MATa deletion collection |  | http://www-sequence.stanford.edu/group/yeast_deletion_project/deletions3.html |
| strain, strain background (*S. cerevisiae*) | ypl226wΔ | YSC1053 Yeast MATa deletion collection |  | http://www-sequence.stanford.edu/group/yeast_deletion_project/deletions3.html |
| strain, strain background (*S. cerevisiae*) | ypl234cΔ | YSC1053 Yeast MATa deletion collection |  | http://www-sequence.stanford.edu/group/yeast_deletion_project/deletions3.html |
| strain, strain background (*S. cerevisiae*) | ypl239wΔ | YSC1053 Yeast MATa deletion collection |  | http://www-sequence.stanford.edu/group/yeast_deletion_project/deletions3.html |
| strain, strain background (*S. cerevisiae*) | ypl240cΔ | YSC1053 Yeast MATa deletion collection |  | http://www-sequence.stanford.edu/group/yeast_deletion_project/deletions3.html |
| strain, strain background (*S. cerevisiae*) | ypl247cΔ | YSC1053 Yeast MATa deletion collection |  | http://www-sequence.stanford.edu/group/yeast_deletion_project/deletions3.html |
| strain, strain background (*S. cerevisiae*) | ypl254wΔ | YSC1053 Yeast MATa deletion collection |  | http://www-sequence.stanford.edu/group/yeast_deletion_project/deletions3.html |
| strain, strain background (*S. cerevisiae*) | ypl259cΔ | YSC1053 Yeast MATa deletion collection |  | http://www-sequence.stanford.edu/group/yeast_deletion_project/deletions3.html |
| strain, strain background (*S. cerevisiae*) | ypl260wΔ | YSC1053 Yeast MATa deletion collection |  | http://www-sequence.stanford.edu/group/yeast_deletion_project/deletions3.html |
| strain, strain background (*S. cerevisiae*) | ypl261cΔ | YSC1053 Yeast MATa deletion collection |  | http://www-sequence.stanford.edu/group/yeast_deletion_project/deletions3.html |
| strain, strain background (*S. cerevisiae*) | ypl268wΔ | YSC1053 Yeast MATa deletion collection |  | http://www-sequence.stanford.edu/group/yeast_deletion_project/deletions3.html |
| strain, strain background (*S. cerevisiae*) | ypl270wΔ | YSC1053 Yeast MATa deletion collection |  | http://www-sequence.stanford.edu/group/yeast_deletion_project/deletions3.html |
| strain, strain background (*S. cerevisiae*) | ypl271wΔ | YSC1053 Yeast MATa deletion collection |  | http://www-sequence.stanford.edu/group/yeast_deletion_project/deletions3.html |
| strain, strain background (*S. cerevisiae*) | ypr008wΔ | YSC1053 Yeast MATa deletion collection |  | http://www-sequence.stanford.edu/group/yeast_deletion_project/deletions3.html |
| strain, strain background (*S. cerevisiae*) | ypr012wΔ | YSC1053 Yeast MATa deletion collection |  | http://www-sequence.stanford.edu/group/yeast_deletion_project/deletions3.html |
| strain, strain background (*S. cerevisiae*) | ypr018wΔ | YSC1053 Yeast MATa deletion collection |  | http://www-sequence.stanford.edu/group/yeast_deletion_project/deletions3.html |
| strain, strain background (*S. cerevisiae*) | ypr020wΔ | YSC1053 Yeast MATa deletion collection |  | http://www-sequence.stanford.edu/group/yeast_deletion_project/deletions3.html |
| strain, strain background (*S. cerevisiae*) | ypr024wΔ | YSC1053 Yeast MATa deletion collection |  | http://www-sequence.stanford.edu/group/yeast_deletion_project/deletions3.html |
| strain, strain background (*S. cerevisiae*) | ypr032wΔ | YSC1053 Yeast MATa deletion collection |  | http://www-sequence.stanford.edu/group/yeast_deletion_project/deletions3.html |
| strain, strain background (*S. cerevisiae*) | ypr036wΔ | YSC1053 Yeast MATa deletion collection |  | http://www-sequence.stanford.edu/group/yeast_deletion_project/deletions3.html |
| strain, strain background (*S. cerevisiae*) | ypr039wΔ | YSC1053 Yeast MATa deletion collection |  | http://www-sequence.stanford.edu/group/yeast_deletion_project/deletions3.html |
| strain, strain background (*S. cerevisiae*) | ypr043wΔ | YSC1053 Yeast MATa deletion collection |  | http://www-sequence.stanford.edu/group/yeast_deletion_project/deletions3.html |
| strain, strain background (*S. cerevisiae*) | ypr044cΔ | YSC1053 Yeast MATa deletion collection |  | http://www-sequence.stanford.edu/group/yeast_deletion_project/deletions3.html |
| strain, strain background (*S. cerevisiae*) | ypr045cΔ | YSC1053 Yeast MATa deletion collection |  | http://www-sequence.stanford.edu/group/yeast_deletion_project/deletions3.html |
| strain, strain background (*S. cerevisiae*) | ypr047wΔ | YSC1053 Yeast MATa deletion collection |  | http://www-sequence.stanford.edu/group/yeast_deletion_project/deletions3.html |
| strain, strain background (*S. cerevisiae*) | ypr057wΔ | YSC1053 Yeast MATa deletion collection |  | http://www-sequence.stanford.edu/group/yeast_deletion_project/deletions3.html |
| strain, strain background (*S. cerevisiae*) | ypr060cΔ | YSC1053 Yeast MATa deletion collection |  | http://www-sequence.stanford.edu/group/yeast_deletion_project/deletions3.html |
| strain, strain background (*S. cerevisiae*) | ypr066wΔ | YSC1053 Yeast MATa deletion collection |  | http://www-sequence.stanford.edu/group/yeast_deletion_project/deletions3.html |
| strain, strain background (*S. cerevisiae*) | ypr067wΔ | YSC1053 Yeast MATa deletion collection |  | http://www-sequence.stanford.edu/group/yeast_deletion_project/deletions3.html |
| strain, strain background (*S. cerevisiae*) | ypr069cΔ | YSC1053 Yeast MATa deletion collection |  | http://www-sequence.stanford.edu/group/yeast_deletion_project/deletions3.html |
| strain, strain background (*S. cerevisiae*) | ypr070wΔ | YSC1053 Yeast MATa deletion collection |  | http://www-sequence.stanford.edu/group/yeast_deletion_project/deletions3.html |
| strain, strain background (*S. cerevisiae*) | ypr074cΔ | YSC1053 Yeast MATa deletion collection |  | http://www-sequence.stanford.edu/group/yeast_deletion_project/deletions3.html |
| strain, strain background (*S. cerevisiae*) | ypr083wΔ | YSC1053 Yeast MATa deletion collection |  | http://www-sequence.stanford.edu/group/yeast_deletion_project/deletions3.html |
| strain, strain background (*S. cerevisiae*) | ypr091cΔ | YSC1053 Yeast MATa deletion collection |  | http://www-sequence.stanford.edu/group/yeast_deletion_project/deletions3.html |
| strain, strain background (*S. cerevisiae*) | ypr092wΔ | YSC1053 Yeast MATa deletion collection |  | http://www-sequence.stanford.edu/group/yeast_deletion_project/deletions3.html |
| strain, strain background (*S. cerevisiae*) | ypr100wΔ | YSC1053 Yeast MATa deletion collection |  | http://www-sequence.stanford.edu/group/yeast_deletion_project/deletions3.html |
| strain, strain background (*S. cerevisiae*) | ypr101wΔ | YSC1053 Yeast MATa deletion collection |  | http://www-sequence.stanford.edu/group/yeast_deletion_project/deletions3.html |
| strain, strain background (*S. cerevisiae*) | ypr111wΔ | YSC1053 Yeast MATa deletion collection |  | http://www-sequence.stanford.edu/group/yeast_deletion_project/deletions3.html |
| strain, strain background (*S. cerevisiae*) | ypr116wΔ | YSC1053 Yeast MATa deletion collection |  | http://www-sequence.stanford.edu/group/yeast_deletion_project/deletions3.html |
| strain, strain background (*S. cerevisiae*) | ypr117wΔ | YSC1053 Yeast MATa deletion collection |  | http://www-sequence.stanford.edu/group/yeast_deletion_project/deletions3.html |
| strain, strain background (*S. cerevisiae*) | ypr119wΔ | YSC1053 Yeast MATa deletion collection |  | http://www-sequence.stanford.edu/group/yeast_deletion_project/deletions3.html |
| strain, strain background (*S. cerevisiae*) | ypr124wΔ | YSC1053 Yeast MATa deletion collection |  | http://www-sequence.stanford.edu/group/yeast_deletion_project/deletions3.html |
| strain, strain background (*S. cerevisiae*) | ypr125wΔ | YSC1053 Yeast MATa deletion collection |  | http://www-sequence.stanford.edu/group/yeast_deletion_project/deletions3.html |
| strain, strain background (*S. cerevisiae*) | ypr131cΔ | YSC1053 Yeast MATa deletion collection |  | http://www-sequence.stanford.edu/group/yeast_deletion_project/deletions3.html |
| strain, strain background (*S. cerevisiae*) | ypr132wΔ | YSC1053 Yeast MATa deletion collection |  | http://www-sequence.stanford.edu/group/yeast_deletion_project/deletions3.html |
| strain, strain background (*S. cerevisiae*) | ypr135wΔ | YSC1053 Yeast MATa deletion collection |  | http://www-sequence.stanford.edu/group/yeast_deletion_project/deletions3.html |
| strain, strain background (*S. cerevisiae*) | ypr139cΔ | YSC1053 Yeast MATa deletion collection |  | http://www-sequence.stanford.edu/group/yeast_deletion_project/deletions3.html |
| strain, strain background (*S. cerevisiae*) | ypr141cΔ | YSC1053 Yeast MATa deletion collection |  | http://www-sequence.stanford.edu/group/yeast_deletion_project/deletions3.html |
| strain, strain background (*S. cerevisiae*) | ypr146cΔ | YSC1053 Yeast MATa deletion collection |  | http://www-sequence.stanford.edu/group/yeast_deletion_project/deletions3.html |
| strain, strain background (*S. cerevisiae*) | ypr153wΔ | YSC1053 Yeast MATa deletion collection |  | http://www-sequence.stanford.edu/group/yeast_deletion_project/deletions3.html |
| strain, strain background (*S. cerevisiae*) | ypr159wΔ | YSC1053 Yeast MATa deletion collection |  | http://www-sequence.stanford.edu/group/yeast_deletion_project/deletions3.html |
| strain, strain background (*S. cerevisiae*) | ypr163cΔ | YSC1053 Yeast MATa deletion collection |  | http://www-sequence.stanford.edu/group/yeast_deletion_project/deletions3.html |
| strain, strain background (*S. cerevisiae*) | ypr164wΔ | YSC1053 Yeast MATa deletion collection |  | http://www-sequence.stanford.edu/group/yeast_deletion_project/deletions3.html |
| strain, strain background (*S. cerevisiae*) | ypr166cΔ | YSC1053 Yeast MATa deletion collection |  | http://www-sequence.stanford.edu/group/yeast_deletion_project/deletions3.html |
| strain, strain background (*S. cerevisiae*) | ypr173cΔ | YSC1053 Yeast MATa deletion collection |  | http://www-sequence.stanford.edu/group/yeast_deletion_project/deletions3.html |
| strain, strain background (*S. cerevisiae*) | ypr179cΔ | YSC1053 Yeast MATa deletion collection |  | http://www-sequence.stanford.edu/group/yeast_deletion_project/deletions3.html |
| strain, strain background (*S. cerevisiae*) | ypr181cΔ | YSC1053 Yeast MATa deletion collection |  | http://www-sequence.stanford.edu/group/yeast_deletion_project/deletions3.html |
| strain, strain background (*S. cerevisiae*) | ypr185wΔ | YSC1053 Yeast MATa deletion collection |  | http://www-sequence.stanford.edu/group/yeast_deletion_project/deletions3.html |
| strain, strain background (*S. cerevisiae*) | ypr189wΔ | YSC1053 Yeast MATa deletion collection |  | http://www-sequence.stanford.edu/group/yeast_deletion_project/deletions3.html |
| strain, strain background (*S. cerevisiae*) | ypr191wΔ | YSC1053 Yeast MATa deletion collection |  | http://www-sequence.stanford.edu/group/yeast_deletion_project/deletions3.html |
| strain, strain background (*S. cerevisiae*) | ypr197cΔ | YSC1053 Yeast MATa deletion collection |  | http://www-sequence.stanford.edu/group/yeast_deletion_project/deletions3.html |
| strain, strain background (*S. cerevisiae*) | ypr199cΔ | YSC1053 Yeast MATa deletion collection |  | http://www-sequence.stanford.edu/group/yeast_deletion_project/deletions3.html |
| strain, strain background (*S. cerevisiae*) | UCC8240 | Gotschling lab - http://www.genetics.org/content/183/1/365.long | | S288C background |
| strain, strain background (*S. cerevisiae*) | UCC8241 | Gotschling lab - http://www.genetics.org/content/183/1/365.long | | S288C background |
| strain, strain background (*S. cerevisiae*) | UCC8335 | Gotschling lab - http://www.genetics.org/content/183/1/365.long | | S288C background |
| strain, strain background (*S. cerevisiae*) | UCC8338 | Gotschling lab - http://www.genetics.org/content/183/1/365.long | | S288C background |
| strain, strain background (*S. cerevisiae*) | UCC8356 | Gotschling lab - http://www.genetics.org/content/183/1/365.long | | S288C background |
| strain, strain background (*S. cerevisiae*) | UCC8357 | Gotschling lab - http://www.genetics.org/content/183/1/365.long | | S288C background |
| strain, strain background (*S. cerevisiae*) | UCC8358 | Gotschling lab - http://www.genetics.org/content/183/1/365.long | | S288C background |
| strain, strain background (*S. cerevisiae*) | UCC8359 | Gotschling lab - http://www.genetics.org/content/183/1/365.long | | S288C background |
| strain, strain background (*S. cerevisiae*) | UCC8360 | Gotschling lab - http://www.genetics.org/content/183/1/365.long | | S288C background |
| strain, strain background (*S. cerevisiae*) | UCC8361 | Gotschling lab - http://www.genetics.org/content/183/1/365.long | | S288C background |
| strain, strain background (*S. cerevisiae*) | UCC8362 | Gotschling lab - http://www.genetics.org/content/183/1/365.long | | S288C background |
| strain, strain background (*S. cerevisiae*) | UCC8363 | Gotschling lab - http://www.genetics.org/content/183/1/365.long | | S288C background |
| strain, strain background (*S. cerevisiae*) | PDR5-GFP | Yeast GFP clone collection - Thermo Fisher Scientific |  | https://www.thermofisher.com/in/en/home/references/protocols/proteins-expression-isolation-and-analysis/gfp-protocol/yeast-gfp-clone-collection.html |
| strain, strain background (*S. cerevisiae*) | PDR5-GFP pdr3Δ | This study |  |  |
| strain, strain background (*S. cerevisiae*) | DBVPG1788 | SGRP Set2 |  | https://catalogue.ncyc.co.uk/sgrp/set-2 |
| strain, strain background (*S. cerevisiae*) | DBVPG1853 | SGRP Set2 |  | https://catalogue.ncyc.co.uk/sgrp/set-2 |
| strain, strain background (*S. cerevisiae*) | DBVPG6040 | SGRP Set2 |  | https://catalogue.ncyc.co.uk/sgrp/set-2 |
| strain, strain background (*S. cerevisiae*) | DBVPG6044 | SGRP Set2 |  | https://catalogue.ncyc.co.uk/sgrp/set-2 |
| strain, strain background (*S. cerevisiae*) | DBVPG6765 | SGRP Set2 |  | https://catalogue.ncyc.co.uk/sgrp/set-2 |
| strain, strain background (*S. cerevisiae*) | K11 | SGRP Set2 |  | https://catalogue.ncyc.co.uk/sgrp/set-2 |
| strain, strain background (*S. cerevisiae*) | L1374 | SGRP Set2 |  | https://catalogue.ncyc.co.uk/sgrp/set-2 |
| strain, strain background (*S. cerevisiae*) | L1528 | SGRP Set2 |  | https://catalogue.ncyc.co.uk/sgrp/set-2 |
| strain, strain background (*S. cerevisiae*) | NCYC110 | SGRP Set2 |  | https://catalogue.ncyc.co.uk/sgrp/set-2 |
| strain, strain background (*S. cerevisiae*) | NCYC361 | SGRP Set2 |  | https://catalogue.ncyc.co.uk/sgrp/set-2 |
| strain, strain background (*S. cerevisiae*) | RM11_1A | SGRP Set2 |  | https://catalogue.ncyc.co.uk/sgrp/set-2 |
| strain, strain background (*S. cerevisiae*) | S288c | SGRP Set2 |  | https://catalogue.ncyc.co.uk/sgrp/set-2 |
| strain, strain background (*S. cerevisiae*) | SK1 | SGRP Set2 |  | https://catalogue.ncyc.co.uk/sgrp/set-2 |
| strain, strain background (*S. cerevisiae*) | UWOPS03-461.4 | SGRP Set2 |  | https://catalogue.ncyc.co.uk/sgrp/set-2 |
| strain, strain background (*S. cerevisiae*) | UWOPS05-217.3 | SGRP Set2 |  | https://catalogue.ncyc.co.uk/sgrp/set-2 |
| strain, strain background (*S. cerevisiae*) | UWOPS05-227.2 | SGRP Set2 |  | https://catalogue.ncyc.co.uk/sgrp/set-2 |
| strain, strain background (*S. cerevisiae*) | UWOPS83-787.3 | SGRP Set2 |  | https://catalogue.ncyc.co.uk/sgrp/set-2 |
| strain, strain background (*S. cerevisiae*) | UWOPS87-2421 | SGRP Set2 |  | https://catalogue.ncyc.co.uk/sgrp/set-2 |
| strain, strain background (*S. cerevisiae*) | W303 | SGRP Set2 |  | https://catalogue.ncyc.co.uk/sgrp/set-2 |
| strain, strain background (*S. cerevisiae*) | Y12 | SGRP Set2 |  | https://catalogue.ncyc.co.uk/sgrp/set-2 |
| strain, strain background (*S. cerevisiae*) | Y55 | SGRP Set2 |  | https://catalogue.ncyc.co.uk/sgrp/set-2 |
| strain, strain background (*S. cerevisiae*) | Y9 | SGRP Set2 |  | https://catalogue.ncyc.co.uk/sgrp/set-2 |
| strain, strain background (*S. cerevisiae*) | YIIc17_E5 | SGRP Set2 |  | https://catalogue.ncyc.co.uk/sgrp/set-2 |
| strain, strain background (*S. cerevisiae*) | YJM789 | SGRP Set2 |  | https://catalogue.ncyc.co.uk/sgrp/set-2 |
| strain, strain background (*S. cerevisiae*) | YJM975 | SGRP Set2 |  | https://catalogue.ncyc.co.uk/sgrp/set-2 |
| strain, strain background (*S. cerevisiae*) | YJM978 | SGRP Set2 |  | https://catalogue.ncyc.co.uk/sgrp/set-2 |
| strain, strain background (*S. cerevisiae*) | YJM981 | SGRP Set2 |  | https://catalogue.ncyc.co.uk/sgrp/set-2 |
| strain, strain background (*S. cerevisiae*) | YPS128 | SGRP Set2 |  | https://catalogue.ncyc.co.uk/sgrp/set-2 |
| strain, strain background (*S. cerevisiae*) | YPS606 | SGRP Set2 |  | https://catalogue.ncyc.co.uk/sgrp/set-2 |
| strain, strain background (*S. cerevisiae*) | YS2 | SGRP Set2 |  | https://catalogue.ncyc.co.uk/sgrp/set-2 |
| strain, strain background (*S. cerevisiae*) | YS4 | SGRP Set2 |  | https://catalogue.ncyc.co.uk/sgrp/set-2 |
| strain, strain background (*S. cerevisiae*) | YS9 | SGRP Set2 |  | https://catalogue.ncyc.co.uk/sgrp/set-2 |
| chemical compound, drug | TMRE | Thermo Fisher Scientific, Cat No. : T669 |  |  |
| chemical compound, drug | MitoTracker green | Thermo Fisher Scientific, Cat No. : M7514 |  |  |
| chemical compound, drug | Fluconazole | Sigma-Aldrich, Cat No. : F8929 |  |  |
